# Single-cell type analysis of wing premotor circuits in the ventral nerve cord of *Drosophila melanogaster*

**DOI:** 10.1101/2023.05.31.542897

**Published:** 2023-06-01

**Authors:** Erica Ehrhardt, Samuel C Whitehead, Shigehiro Namiki, Ryo Minegishi, Igor Siwanowicz, Kai Feng, Hideo Otsuna, FlyLight Project Team, Geoffrey W Meissner, David Stern, Jim Truman, David Shepherd, Michael H. Dickinson, Kei Ito, Barry J Dickson, Itai Cohen, Gwyneth M Card, Wyatt Korff

**Affiliations:** 1 Janelia Research Campus, Howard Hughes Medical Institute, 19700 Helix Dr, Ashburn, Virginia 20147, USA; 2 Institute of Zoology, University of Cologne, Zülpicher Str 47b, 50674 Cologne, Germany; 3 Physics Department, Cornell University, 271 Clark Hall, Ithaca, New York 14853, USA; 4 Queensland Brain Institute, University of Queensland, 79 Upland Rd, Brisbane, QLD, 4072, Australia; 5 California Institute of Technology, 1200 E California Blvd, Pasadena, California 91125, USA; 6 Department of Biology, University of Washington, Seattle, Washington 98195, USA; 7 School of Biological Sciences, Faculty of Environmental and Life Sciences, University of Southampton, Life Sciences Building, Southampton SO17 1BJ

**Keywords:** *Drosophila*, ventral nerve cord, motoneuron, wing, Split-GAL4, flight, courtship song

## Abstract

To perform most behaviors, animals must send commands from higher-order processing centers in the brain to premotor circuits that reside in ganglia distinct from the brain, such as the mammalian spinal cord or insect ventral nerve cord. How these circuits are functionally organized to generate the great diversity of animal behavior remains unclear. An important first step in unraveling the organization of premotor circuits is to identify their constituent cell types and create tools to monitor and manipulate these with high specificity to assess their function. This is possible in the tractable ventral nerve cord of the fly. To generate such a toolkit, we used a combinatorial genetic technique (split-GAL4) to create 195 sparse driver lines targeting 198 individual cell types in the ventral nerve cord. These included wing and haltere motoneurons, modulatory neurons, and interneurons. Using a combination of behavioral, developmental, and anatomical analyses, we systematically characterized the cell types targeted in our collection. Taken together, the resources and results presented here form a powerful toolkit for future investigations of neural circuits and connectivity of premotor circuits while linking them to behavioral outputs.

## Introduction

For animals to survive and reproduce, they must perform precisely controlled movements under the direction of the nervous system. Most of these motor behaviors in insects are facilitated by the vertebrate spinal cord analog, the ventral nerve cord (VNC). The VNC receives and processes sensory information and is involved in generating most of the locomotor actions that underlie fly behaviors such as walking (Bidaye et al., 2014; Tuthill and Wilson, 2016), grooming (Seeds et al., 2014), escape (Card and Dickinson, 2008), flight (Dickinson and Muijres, 2016, Namiki et al. 2022), courtship (Clyne and Miesenböck, 2008), and copulation (Crickmore and Vosshall, 2013; Pavlou et al., 2016).

In flying insects, such as the fruit fly, *Drosophila melanogaster*, subtle adjustments to wing motions can have large aerodynamic consequences that allow flies to rapidly evade predators (Muijres et al., 2014), effectively forage in complex environments (Van Breugel & Dickinson, 2014), and even traverse distances as large as ~15 km in a single flight bout (Coyne et al., 1982; Leitch et al., 2020). On the ground, male flies' tightly patterned and subtle small wing vibrations create a species-specific courtship song necessary to attract mates (Ewing, 1979). Control of behaviors for a given appendage, such as the wing, must use the same limited set of motoneurons and muscles (Dickinson & Tu, 1997; Lindsay et al., 2017; O’Sullivan et al., 2018), but may produce very different motions. For example, flight requires large, synchronized sweeping movements of both wings, whereas song is generated via small vibrations of a single wing. Moreover, flight and song occur in entirely different behavioral contexts. Just how premotor microcircuits in the VNC generate distinct, context-dependent patterns of motor activity using the same set of motoneurons is not well understood (O’Sullivan et al., 2018).

**Figure 1:**
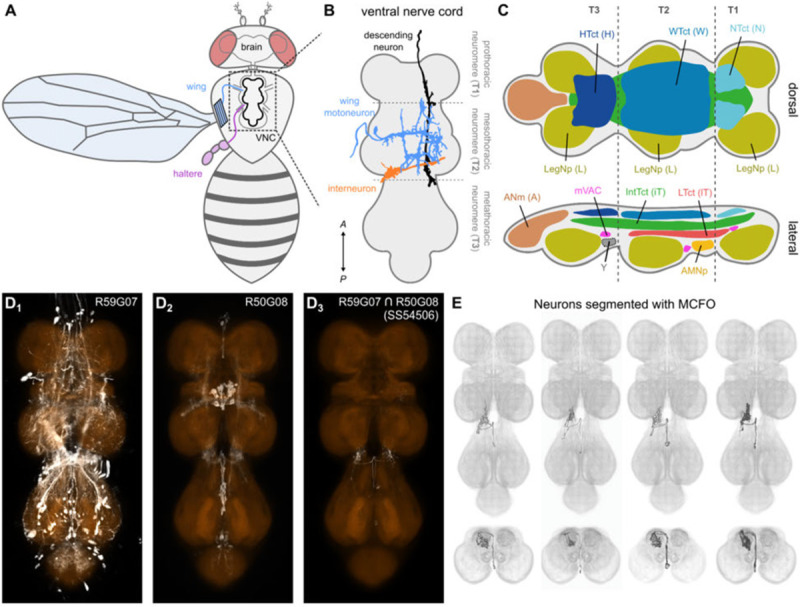
Isolating neurons in the ventral nerve cord (VNC). **(A)** The fly central nervous system (gray) with the ventral nerve cord (VNC) highlighted in black. Illustrations of wing motoneuron and haltere sensory afferent shown in blue and purple, respectively. (**B**) Top-down (dorsal) view of VNC showing example neuron types: wing motoneuron (blue), descending neuron (black), and haltere-to-wing neuropil interneuron (orange). Boundaries between the pro-, meso-, and metathoracic neuromeres—i.e. T1, T2, and T3—are also shown (gray dotted lines and labels). (**C**) Schematic of VNC neuropils. Abbreviations used: T1 (prothoracic segment), T2 (mesothoracic segment), T3 (metathoracic segment), VAC (ventral association center), mVAC (medial ventral association center), and AMNp (accessory mesothoracic neuropil). (**D**) Example usage of the split-GAL4 technique for narrowing driver line expression profile (white) in the VNC (orange). R59G07 (**D**_1_) and R50G08 (**D**_2_) are used to drive half of the GAL4 transcription factor in SS54506 (**D**_3_), resulting in a sparse expression pattern. (**E**) Multiple interneurons segmented from the expression pattern of SS54506 using multicolor flip-out (MCFO).

The *Drosophila* VNC (Figure 1A,B), a set of fused ganglia that contain motoneurons for muscles that move the wings, legs, and other thoracic appendages, is a promising model in which to investigate premotor circuits at the single neuron level because it is numerically tractable (on the order of 15,000 neurons estimated from light microscopy, D Shepherd, personal communication) and amenable to manipulation using genetic tools. Premotor circuits in the VNC receive sensory input from peripheral organs in the wings, legs, halteres, and abdomen (Tsubouchi et al., 2017) and descending commands from the brain (Namiki et al., 2018). Within the microcircuits of the VNC, these multimodal inputs are transformed into patterns of motoneuron activity that produce adaptive motor actions—including walking (Bidaye et al., 2014; Chen et al., 2018; Howard et al., 2019), flight (Schnell et al., 2017; O’Sullivan et al., 2018), grooming (Seeds et al., 2014), escape jumping (King & Wyman, 1980; J. R. Trimarchi & Schneiderman, 1995; von Reyn et al., 2014), and courtship (von Philipsborn et al., 2011; Shirangi et al., 2013). VNC neurons also transmit information to the brain via ascending pathways (Tsubouchi et al., 2017). Understanding the neural basis of motor behavior is thus contingent upon a detailed understanding of the neural circuitry that makes up the VNC. Despite recent advances in both functional recording (Chen et al., 2018) and detailed anatomical mapping of this region (Kuan et al., 2020), relatively little is known about the functional organization of VNC local circuitry (Venkatasubramanian & Mann, 2019).

To facilitate systematically identifying and visualizing the constituent neurons in the local premotor circuits of the VNC, we applied recent advances in combinatorial genetic techniques (Figure 1D; Luan et al., 2006; Jenett et al., 2012) to generate a large-scale collection of transgenic flies that can be used to target individual VNC neurons for both visualization and *in vivo* manipulation as has been done in other regions of the fly nervous system (e.g., Aso et al., 2014; Wolff et al., 2015; Wu et al., 2016; Namiki et al., 2018). The VNC can be broadly subdivided into dorsal and ventral regions: dorsal VNC neuropils control the halteres, neck, and wings in the execution of behaviors like flight and courtship song, while ventral VNC neuropils control the legs for behaviors like walking and grooming (Figure 1C; Court et al., 2020). Here we focus on the dorsal VNC neuropils associated with wing behaviors. We created 195 cell-type specific *Drosophila melanogaster* driver lines targeting motoneurons, the modulatory ventral unpaired median neurons (VUMs), and interneurons in this half of the VNC (Figure 1D,E; Namiki et al., 2018; Court et al., 2020). Ascending neurons that innervate the VNC are beyond the scope of this effort. This library should enable researchers to probe the premotor circuits controlling the rich set of behaviors requiring wing, neck, or haltere coordination, such as flight or courtship.

To help catalyze the use of this library, we performed a combination of behavioral assays and quantitative anatomical analyses to characterize the dorsal VNC organization functionally. Using reagents targeting wing motoneurons, we mapped individual motoneuron manipulations to specific behavioral phenotypes in both flight and courtship song behavioral assays. Using light microscopy and Multicolor Flip-Out (MCFO; Figure 1E), in tandem with reagents targeting dorsal VNC neurons, we segmented 198 cells and annotated both their developmental origins (Doe, 1992; Truman et al., 2010) and their sites of input and output. We then used this information to cluster dorsal VNC interneurons into putative functional groups based on their connectivity patterns and analyzed their volume overlap with descending neurons to elucidate possible roles in the execution of VNC behaviors that originate from top-down brain commands. Together, our results lay the groundwork for a basic functional architecture of the neural circuitry controlling wing movements and provide an important resource for future investigations of the neural substrates underlying motor behavior.

## Results

### Creation of a library of split-GAL4 lines

We used the split-GAL4 system to generate a collection of driver lines with sparse expression in dorsal VNC neurons (Figure 1D,E; Supplemental Figure 1). In the split-GAL4 system, two different enhancers each drive the expression of one domain of the GAL4 transcription factor (the activation domain, AD, or DNA binding domain, DBD), such that only cells expressing both domains produce a functional GAL4 protein and transcribe the gene of interest. This combinatorial approach can produce sparse, even cell-type specific, expression patterns. To produce driver lines with expression in only a single neuronal cell type, we screened the publicly available database of Janelia Gen1 GAL4 lines (Pfeiffer et al., 2008, 2010; Jenett et al., 2012) for pairs of lines that had expression in the same cell type using an automated protocol (Otsuna et al., 2018). From this search, we identified and performed 1,658 AD/DBD candidate intersections, which produced 195 combinations with sufficiently sparse expression to use to make homozygous transgenic lines for inclusion in our collection (Supplemental Figure 1).

We identified 198 unique dorsal VNC cell types targeted by these 195 stabilized driver lines. These included 12 wing power muscle motoneurons (in 16 driver lines), 16 wing control muscle motoneurons (in 34 driver lines), 5 haltere muscle motoneurons (in 15 driver lines), 5 ventral unpaired median (VUM) neurons (in 8 driver lines), 74 intrasegmental interneurons primarily innervating dorsal neuropil regions (in 63 driver lines) and 85 intersegmental interneurons primarily innervating the dorsal neuropils (in 80 driver lines). While generally sparse, many of our driver lines contained multiple cell types. We used the multi-color flip out (MCFO) stochastic labeling technique (Nern et al., 2015) to visualize different cell types expressed in a single driver in different colors, and we used this high-resolution, single-cell image data to create a database of their morphologies (Figure 1E).

Though we targeted dorsal VNC neurons, our screen serendipitously produced several lines sparsely targeting ventral VNC neurons, including at least one set of leg motoneurons in the mesothoracic segment (T2) of the VNC (SS34789), several primary leg sensory neurons (SS40865, SS41034, SS44081), abdominal motor neurons (SS41029, SS48240, SS48247), and a prosternal sensory neuron (SS47204, SS48287).

Split-GAL4 lines from this collection are available to the public and can be found at splitgal4.janelia.org and driver lines will be available at the Bloomington Stock Center in the fall of 2023.

### Wing motoneurons

Motoneurons receive input from premotor neurons in the nerve cord, extend axons through nerves that exit the central nervous system, and terminate in the periphery, where they innervate muscles. They thus comprise the final link between neural activity and motion, and any dissection of premotor circuits should be anchored by an analysis of motoneuron function. Motoneurons are also the easiest neuron type to identify within a given motor circuit, as they can be characterized by their muscle of termination. Yet for many common behaviors, exactly which motoneurons are activated and with what dynamic pattern is still unknown. This is especially true for behaviors involving appendages that must be moved through complex biomechanical articulations, such the fly wing hinge, and in very different kinematic patterns to produce different behaviors, such as flapping vs. singing.

**Figure 2:**
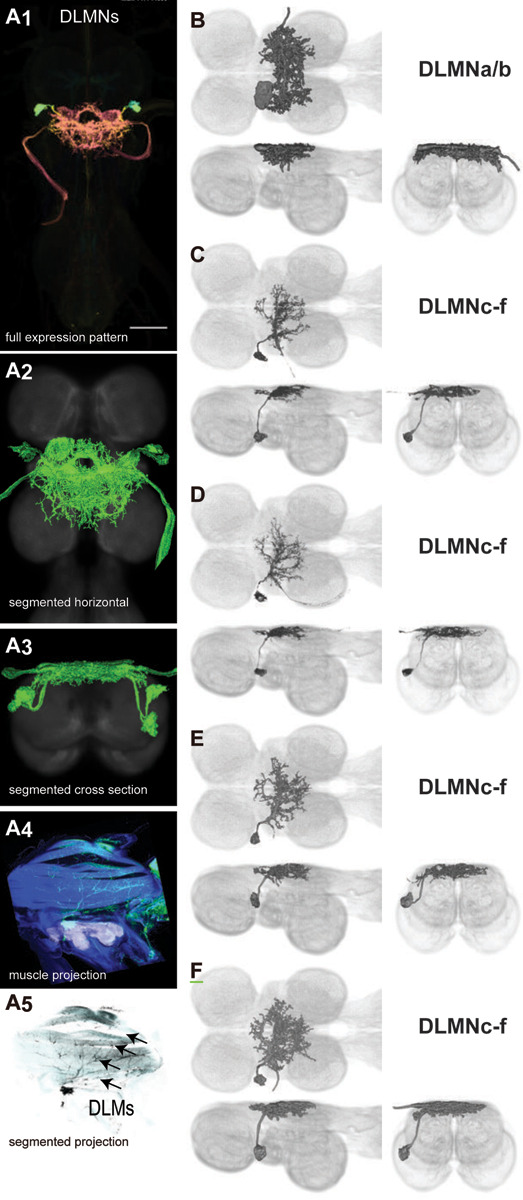
Morphology of DLM power muscle motoneurons. (**A**_1_) Color MIP of full expression pattern of a split line targeting DLMNs, SS44039, crossed with pJFRC51-3xUAS-Syt∷smGFP-HA in su(Hw)attP1; pJFRC225-5xUAS-IVS-myr∷smGFP-FLAG in VK00005, aligned to the JRC 2018 VNC Unisex template. Scale bar in A_1_ represents 50 μm. (**A**_2_) Segmented images of DLM wing motoneurons in upright VNCs. VNCs were aligned to the JRC 2018 Unisex template. (**A**_3_) Transverse views of the segmented neurons shown in A_2_. (**A**_4_) Images of the muscles and their motoneuron innervation in thoraxes stained with phalloidin conjugated with Alexa 633. Phalloidin staining is shown in blue, GFP in green, and nc82 (Bruchpilot, to label the nervous system) in gray. (**A**_5_) Segmented muscle images. (**B-F**) Multicolor flipout (MCFO) was used to separate the power motoneurons, isolating individual cells where possible. Males were used for all motoneuron images.

**Figure 3:**
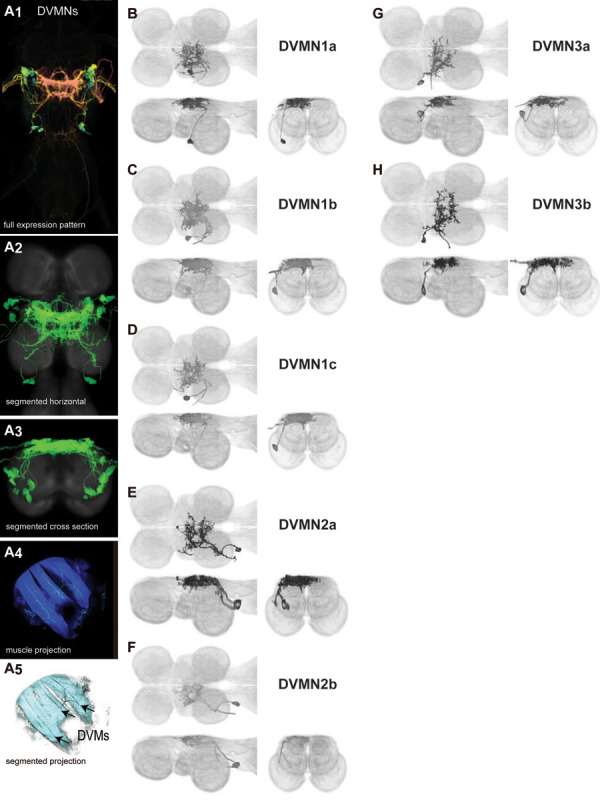
Morphology of DVM power muscle motoneurons. (**A**_1_) Color MIP of full expression pattern of a split line targeting DVMNs, SS31950, crossed with pJFRC51-3xUAS-Syt∷smGFP-HA in su(Hw)attP1; pJFRC225-5xUAS-IVS-myr∷smGFP-FLAG in VK00005, aligned to the JRC 2018 VNC Unisex template. Scale bar in A_1_ represents 50 μm. (**A**_2_) Segmented images of DVM wing motoneurons in upright VNCs. VNCs were aligned to the JFC 2018 Unisex template. (**A**_3_) Transverse views of the segmented neurons shown in A_2_. (**A**_4_) Images of the muscles and their motoneuron innervation in thoraxes stained with phalloidin conjugated with Alexa 633. Phalloidin staining is shown in blue, GFP in green, and nc82 (Bruchpilot, to label the nervous system) in gray. (**A**_5_) Segmented muscle images. (**B-H**) Multicolor flipout (MCFO) was used to separate the power motoneurons, isolating individual cells where possible. Males were used for all motoneuron images.

**Figure 4:**
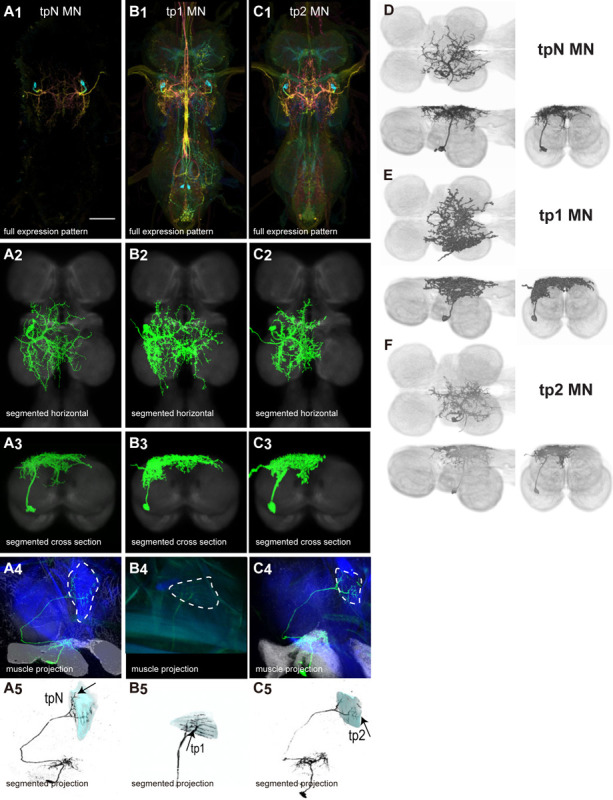
Morphology of tergopleural wing steering muscle motoneurons targeted by our sparse split lines. (**A**-**C**_1_) Color MIPs of full expression patterns of the split lines (respectively SS51528, SS41052, SS47120, SS47204, SS47125, SS40980, SS45772, SS45779, SS41039, SS45782, SS41027, SS45779, SS41027, SS32023,SS37253, SS49039), crossed with pJFRC51-3xUAS-Syt∷smGFP-HA in su(Hw)attP1; pJFRC225-5xUAS-IVS-myr∷smGFP-FLAG in VK00005, aligned to the JRC 2018 VNC Unisex template. Scale bar in A_1_ represents 50 μm. (**A**-**C**_2_) Segmented images of tergopleural wing motoneurons in upright VNCs. Multicolor flipout (MCFO) was used to separate left and right neurons. VNCs were aligned to the JFC 2018 Unisex template. (**A**-**C**_3_) Transverse views of the segmented neurons shown in A-C_2_. (**A**-**C**_4_) Images of the muscles and their motoneuron innervation in thoraxes stained with phalloidin conjugated with Alexa 633. Phalloidin staining is shown in blue, GFP in green, and nc82 (Bruchpilot, to label the nervous system) in gray. (**A**-**C**_5_) Segmented muscle images. (**D**-**F**) segmented images of steering motoneurons including side views. Males were used for all motoneuron images except tpN, as our tpN line lacked expression in males.

**Figure 5:**
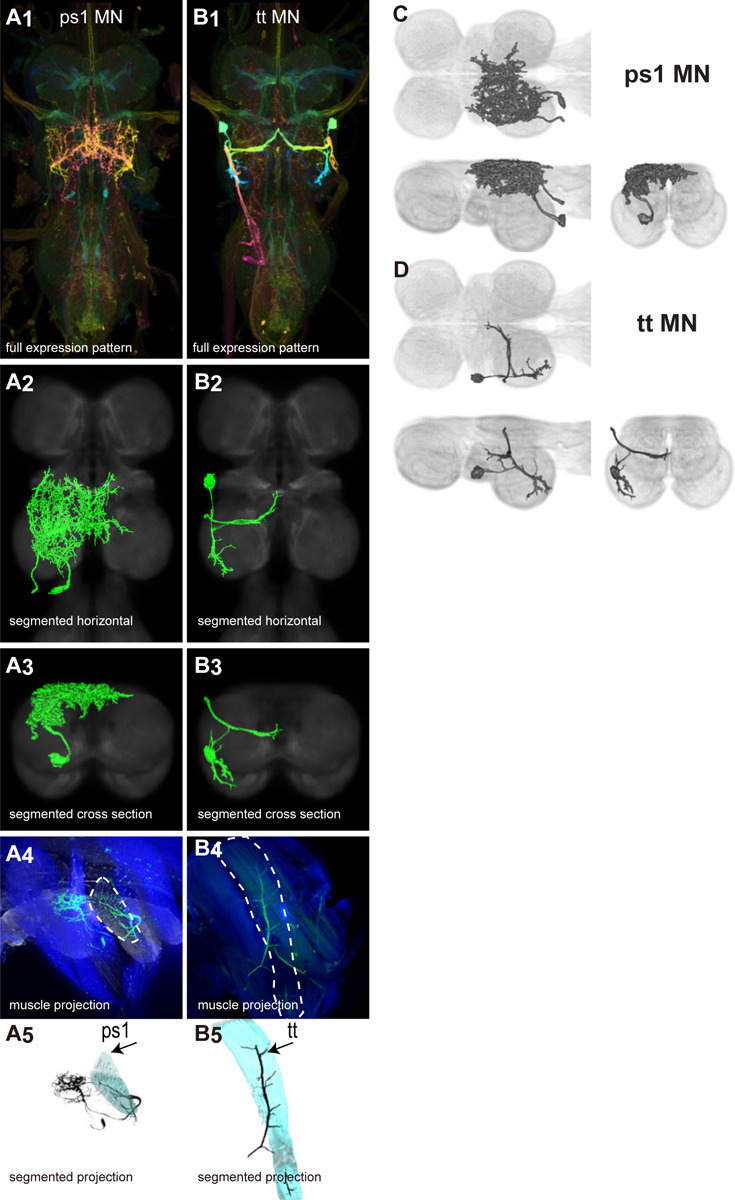
Morphology of other indirect wing steering muscle motoneurons targeted by our sparse split lines. (**A**-**B**_1_) Color MIPs of full expression patterns of the split lines, crossed with pJFRC51-3xUAS-Syt∷smGFP-HA in su(Hw)attP1; pJFRC225-5xUAS-IVS-myr∷smGFP-FLAG in VK00005, aligned to the JRC 2018 VNC Unisex template. Scale bar in A_1_ represents 50 μm. (**A**-**B**_2_) Segmented images of other indirect wing motoneurons in upright VNCs. Multicolor flipout (MCFO) was used to separate left and right neurons. VNCs were aligned to the JFC 2018 Unisex template. (**A**-**B**_3_) Transverse views of the segmented neurons shown in A-B_2_. (**A**-**B**_4_) Images of the muscles and their motoneuron innervation in thoraxes stained with phalloidin conjugated with Alexa 633. Phalloidin staining is shown in blue, GFP in green, and nc82 (Bruchpilot, to label the nervous system) in gray. (**A**-**B**_5_) Segmented muscle images. (**C**-**D**) segmented images of steering motoneurons including side views. Males were used for all motoneuron images.

**Figure 6:**
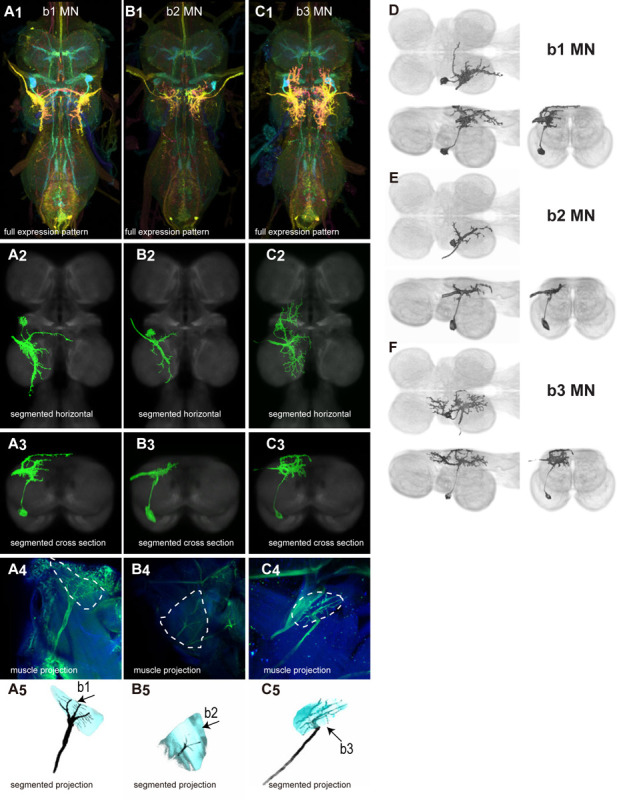
Morphology of basalar wing steering muscle motoneurons targeted by our sparse split lines. (**A**-**B**_1_) Color MIPs of full expression patterns of the split lines, crossed with pJFRC51-3xUAS-Syt∷smGFP-HA in su(Hw)attP1; pJFRC225-5xUAS-IVS-myr∷smGFP-FLAG in VK00005, aligned to the JRC 2018 VNC Unisex template. Scale bar in A_1_ represents 50 μm. (**A**-**B**_2_) Segmented images of basalar wing motoneurons in upright VNCs. Multicolor flipout (MCFO) was used to separate left and right neurons. VNCs were aligned to the JFC 2018 Unisex template. (**A**-**B**_3_) Transverse views of the segmented neurons shown in A-B_2_. (**A**-**B**_4_) Images of the muscles and their motoneuron innervation in thoraxes stained with phalloidin conjugated with Alexa 633. Phalloidin staining is shown in blue, GFP in green, and nc82 (Bruchpilot, to label the nervous system) in gray. (**A**-**B**_5_) Segmented muscle images. (**C**-**D**) segmented images of steering motoneurons including side views. Males were used for all motoneuron images.

**Figure 7:**
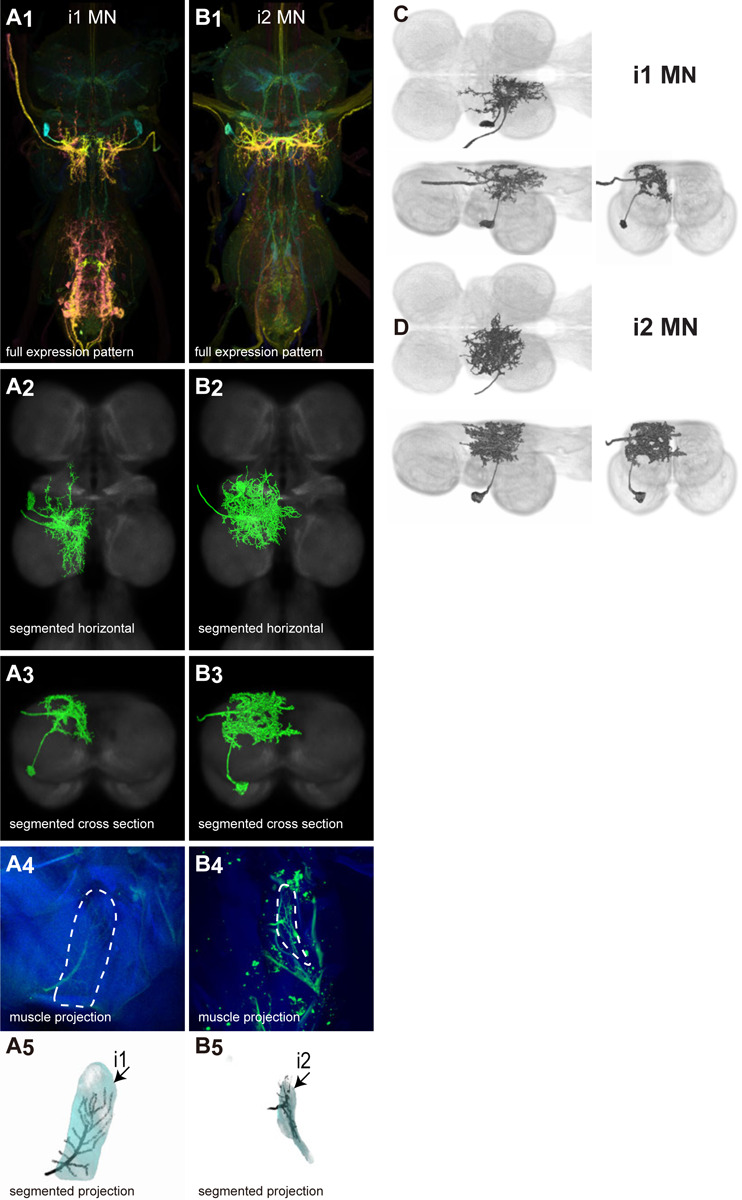
Morphology of first axillary wing steering muscle motoneurons targeted by our sparse split lines. (**A**-**B**_1_) Color MIPs of full expression patterns of the split lines, crossed with pJFRC51-3xUAS-Syt∷smGFP-HA in su(Hw)attP1; pJFRC225-5xUAS-IVS-myr∷smGFP-FLAG in VK00005, aligned to the JRC 2018 VNC Unisex template. Scale bar in A_1_ represents 50 μm. (**A**-**B**_2_) Segmented images of first axillary wing motoneurons in upright VNCs. Multicolor flipout (MCFO) was used to separate left and right neurons. VNCs were aligned to the JFC 2018 Unisex template. (**A**-**B**_3_) Transverse views of the segmented neurons shown in A-B_2_. (**A**-**B**_4_) Images of the muscles and their motoneuron innervation in thoraxes stained with phalloidin conjugated with Alexa 633. Phalloidin staining is shown in blue, GFP in green, and nc82 (Bruchpilot, to label the nervous system) in gray. (**A**-**B**_5_) Segmented muscle images. (**C**-**D**) segmented images of steering motoneurons including side views. Males were used for all motoneuron images.

**Figure 8:**
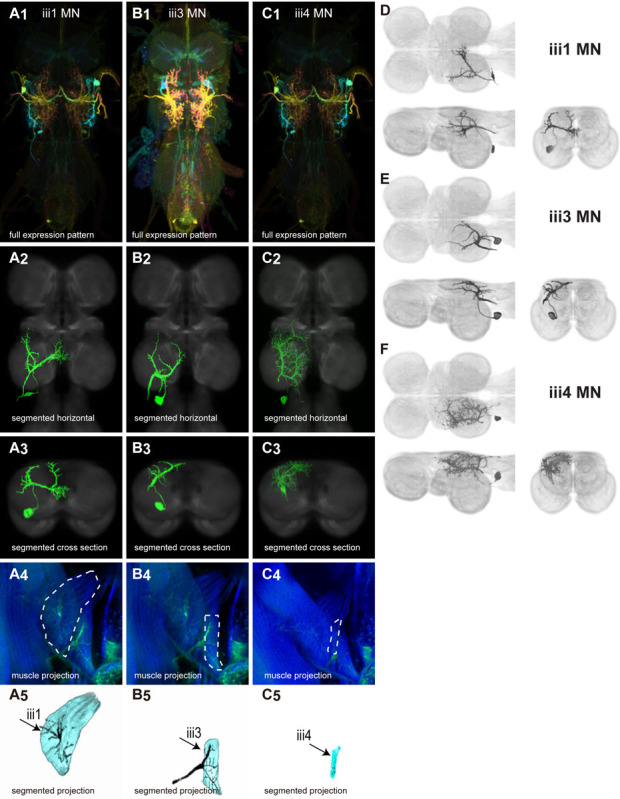
Morphology of third axillary wing steering muscle motoneurons targeted by our sparse split lines. (**A**-**B**_1_) Color MIPs of full expression patterns of the split lines, crossed with pJFRC51-3xUAS-Syt∷smGFP-HA in su(Hw)attP1; pJFRC225-5xUAS-IVS-myr∷smGFP-FLAG in VK00005, aligned to the JRC 2018 VNC Unisex template. Scale bar in A_1_ represents 50 μm. (**A**-**B**_2_) Segmented images of third axillary wing motoneurons in upright VNCs. Multicolor flipout (MCFO) was used to separate left and right neurons. VNCs were aligned to the JFC 2018 Unisex template. (**A**-**B**_3_) Transverse views of the segmented neurons shown in A-B_2_. (**A**-**B**_4_) Images of the muscles and their motoneuron innervation in thoraxes stained with phalloidin conjugated with Alexa 633. Phalloidin staining is shown in blue, GFP in green, and nc82 (Bruchpilot, to label the nervous system) in gray. (**A**-**B**_5_) Segmented muscle images. (**C**-**D**) segmented images of steering motoneurons including side views. Males were used for all motoneuron images.

**Figure 9:**
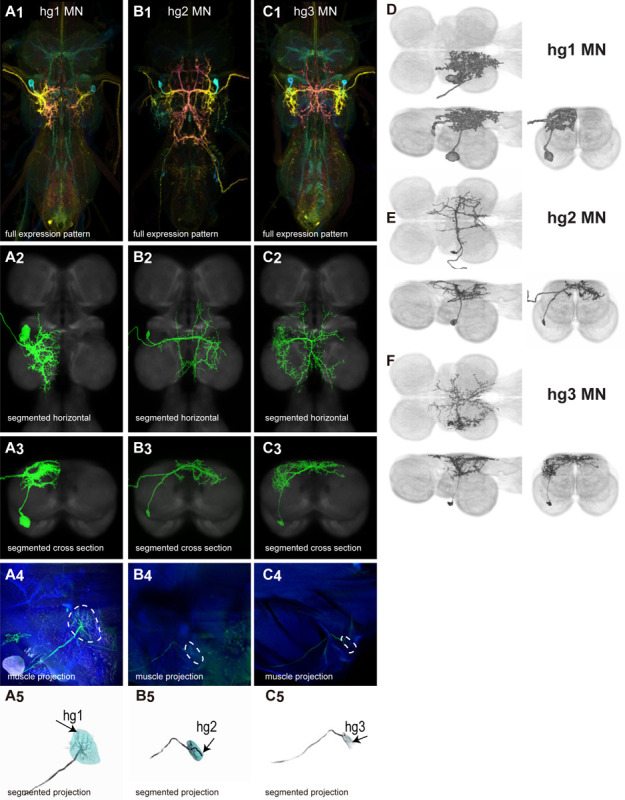
Morphology of fourth axillary wing steering muscle motoneurons targeted by our sparse split lines. (**A**-**C**_1_) Color MIPs of full expression patterns of the split lines, crossed with pJFRC51-3xUAS-Syt∷smGFP-HA in su(Hw)attP1; pJFRC225-5xUAS-IVS-myr∷smGFP-FLAG in VK00005, aligned to the JRC 2018 VNC Unisex template. Scale bar in A_1_ represents 50 μm. (**A**-**C**_2_) Segmented images of fourth axillary wing motoneurons in upright VNCs. Multicolor flipout (MCFO) was used to separate left and right neurons. VNCs were aligned to the JFC 2018 Unisex template. (**A**-**C**_3_) Transverse views of the segmented neurons shown in A-C_2_. (**A**-**C**_4_) Images of the muscles and their motoneuron innervation in thoraxes stained with phalloidin conjugated with Alexa 633. Phalloidin staining is shown in blue, GFP in green, and nc82 (Bruchpilot, to label the nervous system) in gray. (**A**-**C**_5_) Segmented muscle images. (**D**-**F**) segmented images of steering motoneurons including side views. Males were used for all motoneuron images except tpN, as our tpN line lacked expression in males.

To provide tools for analyzing motor circuits and probing motoneuron function, we sought to produce a comprehensive set of driver lines that targeted each of the motoneurons for the primary appendage driven by dorsal VNC microcircuits: the wing. The Dipteran wing motor system is highly specialized, and its constituent muscles can be broadly separated into two distinct categories: the large, asynchronous power muscles (Figures 2–3) and the small, synchronous control muscles (Figures 4–9). While they receive innervation from motoneurons, the power muscles contract at a frequency determined by the resonant properties of the insect’s thorax rather than the spike rate of motoneuron inputs (Pringle, 1949; Boettiger, 1960). In contrast, the 17 pairs of control muscles directly influence more subtle motions of the wings via attachments onto the wing hinge. We created 46 driver lines that covered 12/12 of the motoneurons innervating the power muscles and 16/18 of the motoneurons innervating the control muscles with varying levels of specificity.

#### Power muscle motoneurons

Four pairs of power muscles span the thoracic cavity. These are divided into two classes, orthogonally arranged, whose alternate contraction drives wing motion via deformations of the thorax that power the primary wing oscillations in flight and song (Pringle, 1949; Dickinson & Tu, 1997). The power muscles on each side of the body consist of one dorsal longitudinal muscle (DLM, divided into six large fibers), and three dorsal ventral muscles (DVMs, collectively divided into seven large fibers). Dorsal longitudinal motoneurons (DLMNs) innervate the DLMs: The two most dorsal DLM muscle fibers are innervated by DLMNa/b, while the four ventral DLM muscle fibers are each innervated by one of the other four DLMNs, DLMNc-f (Coggshall, 1978). In flies, the DVMs are innervated by seven motoneurons, with each motoneuron innervating a single DVM fiber (Trimarchi and Schneiderman, 1995; Schlurmann and Hausen, 2007).

We generated 16 lines targeting DLMNs and DVMNs. The morphology and innervation patterns of these power motoneurons are shown in Figures 2–3. These lines were categorized as being either 1) solely selective for DLMNs (no strong DVMN expression: SS31541, SS31561, SS44039, and SS44056), 2) largely selective for DVMNs motoneurons (expression in one or more DLMNs: SS41068 and SS49797), or 3) exhibiting mixed expression in both DLMNs and DVMNs (SS31543, SS31950, SS31997, SS37294, SS37295, SS40765, SS40772, SS40989, SS43980, SS44060). To confirm the identity of each set of motoneurons, we identified which muscles they innervated by co-staining the expression pattern of each split-GAL4 line with GFP and the muscles with phalloidin. In separate experiments, we used multi-color flip out (MCFO) to isolate individual power muscle motoneurons within our split-GAL4 lines and manually segment them where possible, creating digital meshes of individual neurons (or, where they could not be separated, bilateral pairs) to better characterize their morphology within the central nervous system (Figures 2B–F, 3B–H).

DLMNc-f are nearly identical and their somata form a closely apposed cluster (Figure 2C–F). The DLMNs could not be separated using split-GAL4 driver lines; every line with any DLMN expression had expression in all DLMns. Due to the nature of our technique, muscle images and high-quality VNC images could not be obtained from the same MCFO preparation. Thus we were unable to identify which DLMN neuron in our MCFO data corresponded to which specific DLM fiber and we refer to these as a group, DLMNc-f. In contrast, DLMNa/b, which innervates two DLM fibers, was definitively identified based on its morphology within the VNC, including its sizeable dorsal soma and contralateral axon (Figure 2B).

Among the DVMNs (Figure 3), soma positions are comparatively unique and their correspondence to DVM innervated has been established (Schlurmann and Hausen, 2007). The muscle DVM 1 is innervated by three motoneurons (DVMN1a-c, Figure 3B–D), whose somata are connected to their dendritic arbors by a neurite that projects posteriorly away from the soma before bending back to the anterior wing neuropil. The muscle DVM 2 is innervated by two motoneurons with posterior somata (DVMN2a-b, Figure 3E–F). In our MCFO preparations, DVMN2a never had expression on its own, so it could not be segmented and appears in the same image as DVMN1b (Figure 3E). The muscle DVM 3 is innervated by two motoneurons with anterior somata (DVMN3a-b, Figure 3G–H).

#### Control muscle motoneurons

There are 17 control muscles thought to modulate wing motion on rapid time scales (Dickinson & Tu, 1997). Twelve attach directly to hardened sclerites at the wing hinge and directly influence wing motion. These are named based on their points of insertion: the basalars (b1, b2, b3), first axillaries (i1, i2), third axillaries (iii1, iii3, iii4), and fourth axillaries (hg1, hg2, hg3, hg4) (Dickinson & Tu, 1997). The remaining control muscles do not attach to the wing hinge but could affect wing movements by changing the resonance frequency of the thorax. These include the tergopleurals (tp1, tp2), pleurosternals (ps1, ps2), and tergotrochanter (tt) muscles (Dickinson & Tu, 1997). These 17 control muscles are innervated by 18 known motoneurons—one motoneuron for each control muscle plus the tpN motoneuron, which innervates both the tp1 and tp2 muscles (Trimarchi & Schneiderman, 1994; O’Sullivan et al., 2018). Unlike the power muscles, each control muscle contracts synchronously with individual motoneuron spikes (Trimarchi & Schneiderman, 1994; Lindsay et al., 2017; O’Sullivan et al., 2018).

We generated 34 driver lines that targeted the wing control motoneurons. 18 of these driver lines are expressed in a single motoneuron cell type (Figures 4–9). These single neuron driver lines included both motoneurons to both direct steering muscles (i1, i2, hg1, hg2, hg3) and indirect control muscles (ps1, tp1, tp2, tpN). The remaining 16 driver lines expressed in more than one motoneuron type but collectively covered 16/18 wing control motoneurons. Some MNs in these multiple-MN driver lines could be identified and segmented (tt, b1, b2, iii1, iii3). The control motoneurons covered in our collection innervate a mix of both tonic (b1, b3, i2, iii3) and phasic (b2, i1, iii1, iii4, hg1, hg2, hg3) muscles, the two functionally distinct categories of direct steering muscles (Lindsay et al., 2017).

### Functional experiments using our wing motoneuron split lines in tethered flight and song

The articulation of the Dipteran wing is one of nature’s most complicated arrangements, including direct muscle attachment to move the wing hinge and indirect movement of the hinge via other muscles that span and deform the thorax. Thus, the contribution of any given muscle and its corresponding motoneuron, to wing motion cannot be inferred from anatomy alone. To demonstrate the utility of our driver lines and to provide a functional baseline for future analyses of premotor circuits, we used the motoneuron driver lines described above to probe the roles of both power and control wing muscles in the execution of two distinct wing behaviors: flight and courtship song. Previous work suggests these behaviors are coordinated by overlapping sets of muscles (Heide & Götz, 1996; Lindsay et al., 2017; O’Sullivan et al, 2018). Our collection of driver lines and quantitative behavioral assays allow us to confirm and expand upon these previous results to further understand how wing motoneurons participate in flight and song.

To complement existing data, we used our most sparse-expressing motoneuron lines to quantify the phenotypes of single muscle activation during flight or inactivation in courtship. Because the motoneuron-to-muscle relationship is one-to-one for the motoneurons we tested, we interpret our results as activating or silencing individual muscles. In flight we evaluated the roles of eight muscles, including the two different sets of power muscles (DLM, DVM) and six control muscles (tp2, ps1, i1, i2, hg1, hg2). In courtship we studied a subset of wing motoneurons (DVM, tp2, ps1, i2, hg1, hg2, hg3). As a genetic control, we tested flies using “empty” split-GAL4 lines with the same genetic background but no GAL4 expression (SS01062, for flight studies and SS01062 and SS01055 for courtship, Namiki et al., 2018).

**Figure 10:**
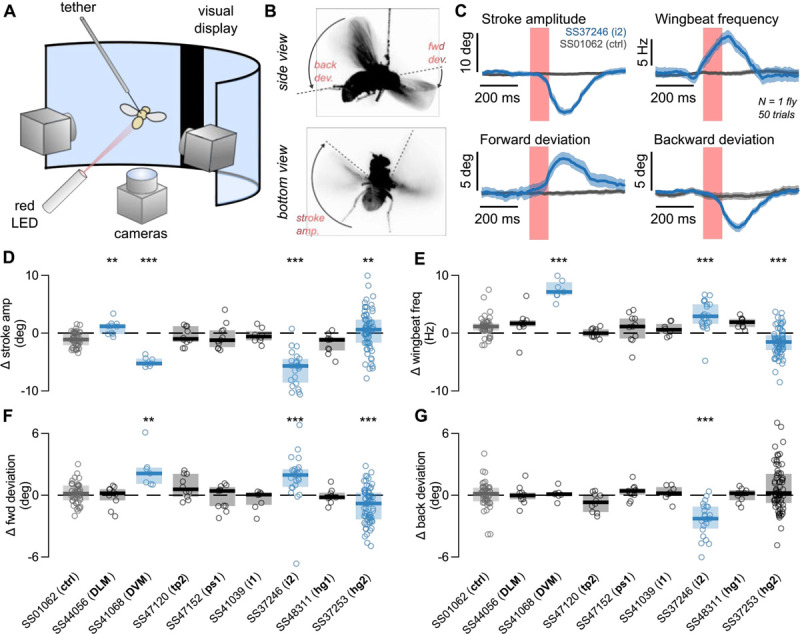
Optogenetic stimulation of wing motor neurons evokes changes to flight kinematics. (**A**) Schematic of tethered flight measurement apparatus. Tethered flies are positioned in front of a display screen that presents both open- and closed-loop visual stimuli. The fly is illuminated near-infrared LEDs and filmed by three cameras recording at 100 fps. An additional red LED (617 nm) provides optogenetic stimulation in 100 ms pulses. (**B**) Stills from two of the three cameras recording the tethered fly in flight. Top panel (side view) illustrates the forward (fwd) and backward (back) deviation angles; bottom panel (bottom view) illustrates the stroke amplitude. (**C**) Averaged wing kinematics for two flies undergoing 50 repetitions of a closed loop trial with 100 ms optogenetic pulse. Dark lines and envelopes show the mean and 95% confidence interval, respectively. Traces in blue correspond to a UAS-CsChrimson > i2-GAL4 fly; traces in gray show an example genetic control, UAS-CsChrimson > SS01062, where SS01062 is an empty split Gal4 line. (**D**-**G**) Statistics across flies for the four kinematic variables shown in (C). Open circles show per-fly measurements; bars and horizontal lines show interquartile range and population median, respectively. Significance is determined via Wilcoxon rank sum test with Bonferroni correction (***, p<0.001; **, p<0.01; *, p<0.05).

Previous studies of wing muscles during flight have either monitored activity in a subset of them while a tethered fly was flying or evaluated whether silencing specific motoneurons prevented flight at a general level. These studies indicated that activity in specific wing control muscles acts to make small changes in wing kinematics that have large aerodynamic consequences for controlling the fly’s flight trajectory. Here we were able to perform a new kind of experiment in which we used our lines to drive expression of a red-shifted channelrhodopsin (CsChrimson) in single motoneurons, allowing us to optogenetically activate specific motoneurons, and hence wing muscles, during tethered flight and quantify what effect this had on the details of wing kinematics. Using an automated video tracking algorithm (“Kinefly,” see Methods) we quantified the activation-induced changes in four wing kinematic parameters: wingbeat frequency, back and forward deviation of the wings, and stroke amplitude (Figure 10). We found that activation of one type of power muscle, the DLMs, increased stroke amplitude, whereas activation of the other power muscle type, the DVMs, decreased stroke amplitude, though forward deviation of the wing increased, and had a large effect on increasing wingbeat frequency (Figure 10D–G, p<0.05, Wilcoxon rank sum test compared to control genotype). The activation of i2 had the strongest effect in our screen, with a phenotype similar to DVM activation with the addition of a decrease in backward wing deviation. Activation of hg2 produced changes in three of our four wing kinematic parameters, but the mean value of the parameters did not change much. Rather, the variance we observed between flies increased substantially. Activation of the motoneurons for the remaining muscles (tp2, ps1, i1, hg1) did not lead to any observable phenotypes. This was a surprising result for tp2, which has previously been implicated as critical for flight and correct wing posture (O’Sullivan et al, 2018). One interpretation of our result is that, to engage the correct wing posture during flight, the tp2 muscle is maximally active and hence further motoneuron activation does not alter wing kinematics.

**Figure 11:**
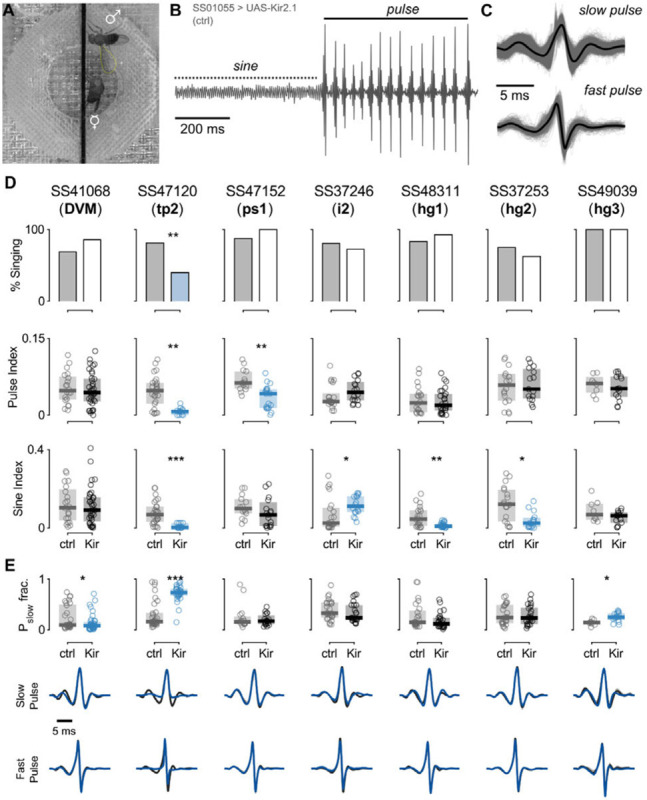
Chronic silencing of wing motor neurons results in courtship song deficits. (**A**) Image from a typical courtship assay, showing a male (above) extending its left wing to sing to a female (below). (**B**) Example trace of song recording from a control group fly. Bouts of sine and pulse song are labelled on the left and right of the trace, respectively. (**C**) Slow (top) and fast (bottom) pulse modes for a single fly. Thick black lines show the mean pulse shape; thin gray lines show individual pulses. (**D**) Statistics across genotypes for different song parameters: total fraction of tested flies that sing (top), fraction of song spent singing pulse mode song (middle), and fraction of song spent singing sine mode song (bottom). Open circles show per-fly measurements; bars and horizontal lines show interquartile range and population median, respectively. (**E**) Analysis of pulse type. Top row shows the fraction of pulses that are classified as slow for each genotype. Middle and bottom rows show waveforms for slow and fast pulse modes for each genotype (blue), overlaid onto control (dark gray). Thick line shows the grand mean across flies; envelope gives 95% confidence interval for mean from bootstrap. Significance assigned using the Wilcoxon rank sum test and Fisher’s exact test (***, p<0.001; **, p<0.01; *, p<0.05).

To investigate the role of wing muscles in courtship song, we silenced individual motoneurons in male flies using our motoneuron-specific lines to drive either the inwardly-rectifying potassium channel Kir2.1 (UAS-Kir2.1) to hyperpolarize the neurons and prevent spiking or tetanus neurotoxin light chain (UAS-TNTe), to block synaptic transmission (Sweeney et al., 1995; Baines et al., 2001). Motoneuron-silenced males were individually housed before introduction into a recording chamber with a 1-day old virgin female, where we recorded 30 minutes of courtship song (Figure 11A). Because experiments with both silencing reporters produced similar results, Figure 11 shows data from only Kir2.1 flies.

The song of *Drosophila melanogaster* contains two primary components: sine song and pulse song (Figure 11B). Following previous studies (Shirangi et al., 2013; O’Sullivan et al., 2018), we analyzed the fraction of flies within a given genotype that produced a song during the assay (Figure 11D, top), as well as the fraction of the song devoted to sine and pulse song, denoted sine index and pulse index, respectively (Figure 11D, middle and bottom). We found that silencing tp2 affected nearly all aspects of song production, silencing ps1 decreased production of pulse song, and silencing i2, hg1, and hg2 decreased production of sine song (*p*<0.05, Figure 11D).

To expand upon these and previous results, we further analyzed the details of pulse song production. Recent studies uncovered two distinct categories of pulses: fast and slow (Figure 11C; Clemens et al., 2018). Figure 11E shows averaged fast and slow pulse shapes for each genotype (blue, top and middle) in our experiments compared to control flies (gray, top and middle). We compared the relative proportion of fast and slow pulse types produced during singing, normalized to the total number of pulses produced, and found that DVM-silenced flies produced significantly fewer slow-mode pulses, while hg3-silenced flies produced significantly more slow-mode pulses (*p*<0.05; Figure 11E). Flies with the tp2 motoneuron silenced showed a significant increase in fast pulses produced, but this may be an epiphenomenon related to the low number of total pulses produced by these flies. These results were largely consistent with previous experiments of the same type (Shirangi et al., 2013; O’Sullivan et al., 2018), with the following differences: previously tp2 and i2 were labeled as pulse song specific muscles, whereas we see sine song changes upon their silencing.

The effect of DVM silencing on courtship song had not previously been reported, for lack of a DVMN-specific driver line. We used the DVMN line generated in this study and found that courtship song in DVMN silenced flies was largely unimpaired, with no change to time spent singing or amount of sine or pulse song. However, these flies did show a small but significant reduction in the fraction of pulse song using slow pulses, suggesting that the power muscle oscillation may be more critical for slow pulses than fast ones.

A critical question in the organization of premotor circuits is to what extent circuits coordinating different actions with the same appendage overlap. If there are muscles that are used exclusively in one behavior versus another, we might expect that these would have more distinct premotor circuits than muscles that are very active in multiple behaviors. Thus, understanding the role of the muscles for a given appendage across multiple behaviors is an important starting point for unraveling premotor organization. Previous studies, together with our results here, suggest that there is a large overlap in wing muscles involved in both flight and courtship, including both sets of power muscles (DLM and DVM) as well as control muscles tp1-2, tpN, i2, hg2, hg4, iii1-4 and tt. However, there may also be some separation in control. The b1 and b2 muscles do not seem to be used in courtship and are thus flight-specific. Likewise, hg1, hg3, and ps1 appear courtship-specific in the studies to date, and ps1 is critical to pulse song, whereas hg1 is critical to sine song. Our results here suggest additional specificity of function during courtship, with DVMs important for producing slow pulses and tp2 muscles important for producing fast pulses. These distinctions lay the groundwork for future studies to look at the overlap in premotor circuitry amongst these functional muscle groupings.

### Haltere motoneurons

**Figure 12:**
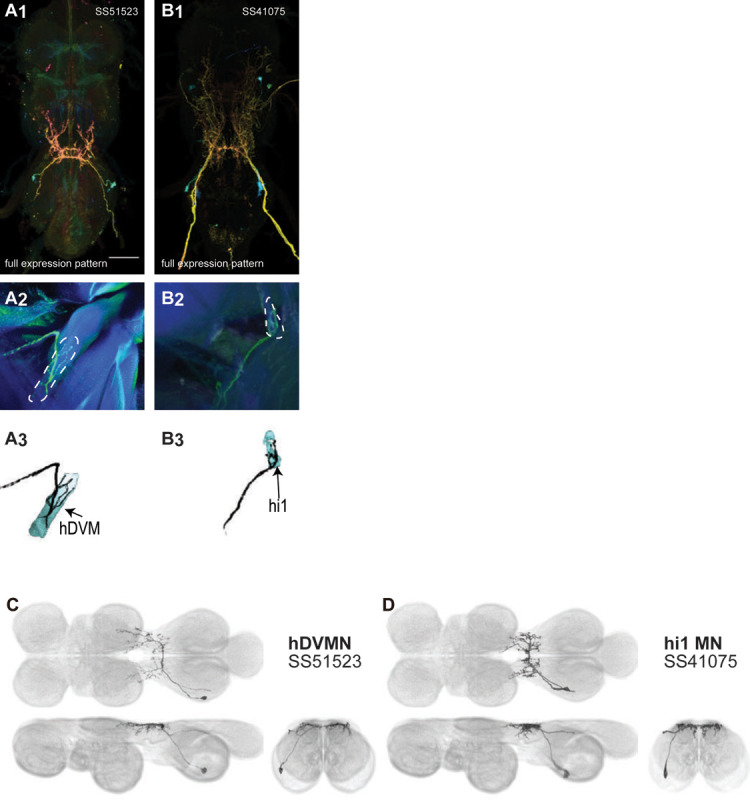
Morphology of haltere motoneurons targeted by our sparse split lines. (**A**-**B**_1_) Color MIPs of full expression patterns of the split lines, crossed with pJFRC51-3xUAS-Syt∷smGFP-HA in su(Hw)attP1; pJFRC225-5xUAS-IVS-myr∷smGFP-FLAG in VK00005, aligned to the JRC 2018 VNC Unisex template. Scale bar in A_1_ represents 50 μm. (**A**-**B**_2_) Images of the muscles and their motoneuron innervation in thoraxes stained with phalloidin conjugated with Alexa 633. Phalloidin staining is shown in blue, GFP in green, and nc82 (Bruchpilot, to label the nervous system) in gray. (**A**-**B**_3_) Segmented muscle images. (**C**-**D**) segmented images of haltere motoneurons.

**Figure 13:**
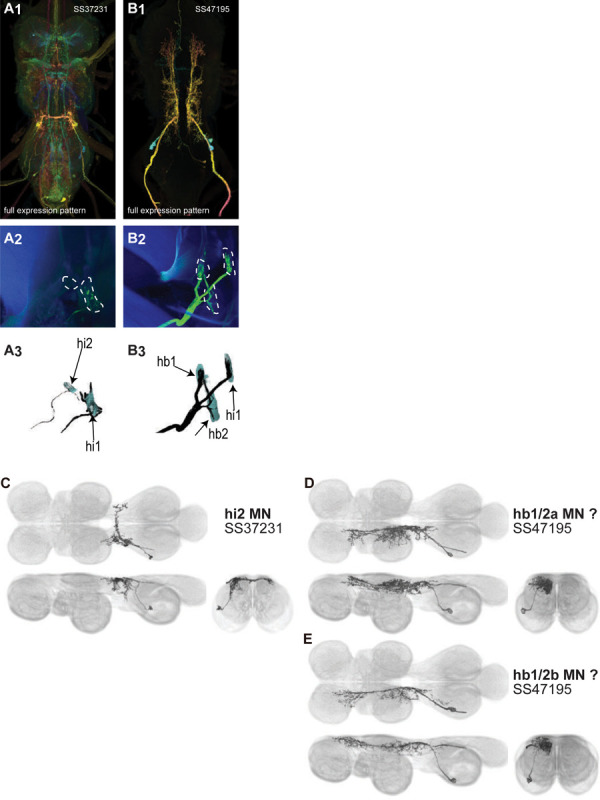
Morphology of haltere motoneurons targeted by our broad split lines. (**A**-**B**_1_) Color MIPs of full expression patterns of the split lines, crossed with pJFRC51-3xUAS-Syt∷smGFP-HA in su(Hw)attP1; pJFRC225-5xUAS-IVS-myr∷smGFP-FLAG in VK00005, aligned to the JRC 2018 VNC Unisex template. Scale bar in A_1_ represents 50 μm. (**A**-**B**_2_) Images of the muscles and their motoneuron innervation in thoraxes stained with phalloidin conjugated with Alexa 633. Phalloidin staining is shown in blue, GFP in green, and nc82 (Bruchpilot, to label the nervous system) in gray. (**A**-**B**_3_) Segmented muscle images. (**C**-**E**) segmented images of haltere motoneurons.

The haltere is associated with its own power muscle, hDVM, and six steering muscles. We generated a total of 15 driver lines that targeted the haltere motoneurons (Figures 12–13). Some of the haltere motoneurons could be identified and segmented (hDVM, hi1, hi2). The hb1 and hb2 motoneurons had expression in SS47195, along with hi1 MN (Figure 13B). Two neurons in SS47195 were segmented using MCFO (Figure 13D–E). However, we were unable to determine which of these cells innervated hb1 and which innervated hb2, so we named the cells hb1/2a and hb1/2b. SS36076 had expression in hiii3 MN, but we were unable to segment any image of this motoneuron.

In the VNC, the neurites of most haltere motoneurons mainly arborize in the haltere neuropil. However, hb1/2a and hb1/2b had major innervation not only in the haltere neuropil, but the wing and neck neuropils and intermediate tectulum as well. The intersegmental arborization of these haltere motoneurons may be involved in coordinating the neck, wing and haltere during steering or flight stabilization.

With the exception of tt MN, in the wing motoneuron neurites we only observed input sites in the VNC. However, the varicose morphology associated with output sites was visible in some of the neurites of hDVMN, hb1/2 MN, and hi2 MN in the VNC. We therefore examined the localization of synaptotagmin, a synaptic vesicle-specific protein, in our haltere motoneuron split lines. The split lines targeting hDVMN, hb1/2 MN and hi MN had a strong synaptotagmin signal in the haltere neuropil, supporting the observation of output site morphology. Weak synaptotagmin signal was associated with the neck and wing neuropil and T1 intermediate tectulum arborization of hb1/2 MN.

### VUM neurons

**Figure 14:**
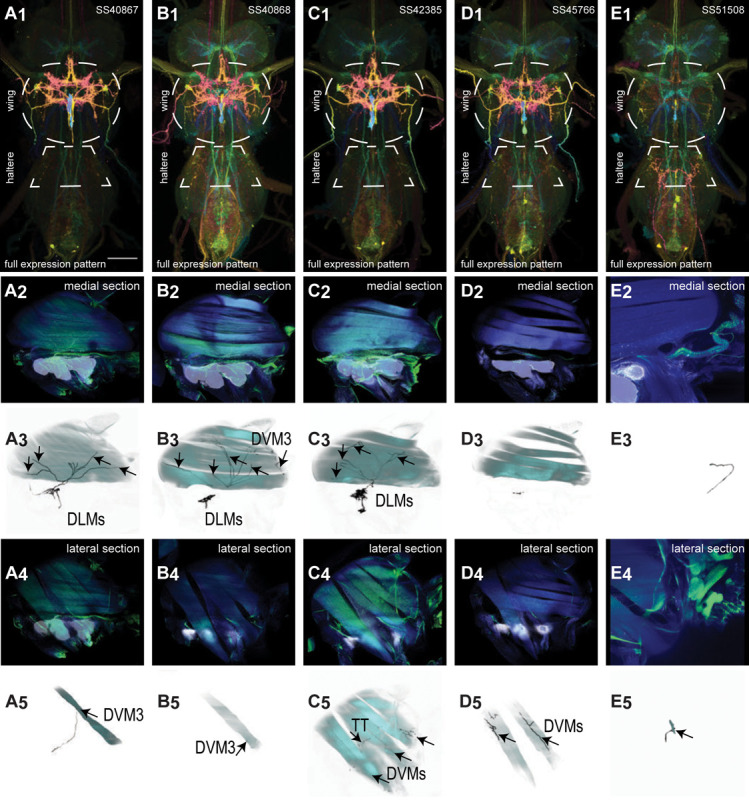
Morphology of VUMs in our split lines. (**A**_1_-**E**_1_) Color MIPs of the full expression pattern of each split in the VNC of males crossed with pJFRC51-3xUAS-Syt∷smGFP-HA in su(Hw)attP1; pJFRC225-5xUAS-IVS-myr∷smGFP-FLAG in VK00005, aligned to the JRC 2018 VNC Unisex template. Scale bar in A_1_ represents 50 μm. Wing and haltere neuropils are indicated by dashed white outlines. (**A**-**E**_2_) Medial views of the muscles and their motoneuron innervation in thoraxes stained with phalloidin conjugated with Alexa 633. Phalloidin staining is shown in blue, GFP in green, and nc82 (Bruchpilot, to label the nervous system) in gray. (**A**-**E**_3_) Segmented medial muscle images. (**A**-**E**_4_) Lateral views of the muscles and their motoneuron innervation. (**A**-**E**_5_) Segmented lateral muscle images. Males were used for all images.

In addition to excitatory motoneurons, Dipteran wing muscles are innervated by mesothoracic ventral unpaired median (T2VUM) neurons, neuromodulatory cells that play a crucial role in coordinated wing behaviors (Schlurmann & Hausen, 2003; Sadaf et al., 2015). These T2VUM cells are members of a broader category of largely efferent neurons called ventral unpaired median (VUM) neurons, so named for their somata, which cluster along the midline of the VNC in the ventral cortex, and their bilaterally symmetric neurite projections (Figure 14A–D; Schlurmann & Hausen, 2003). While a recently identified VUM cell in *Drosophila* has been shown to be dopaminergic (Sadaf et al., 2015), octopaminergic VUM neurons innervating wing muscles have been identified in several other insect species, including *Calliphora* (blowflies) and *Schistocerca* (desert locusts), and may be a general feature of the neural circuitry controlling insect wings (Schlurmann & Hausen, 2003; Stocker et al., 2018).

We created four driver lines targeting T2VUM neurons (Figure 14A–D). We identified the muscles innervated by these T2VUM cells as above by using our split lines to express the fluorescent reporter GFP staining hemithoraces of these flies with phalloidin to visualize the muscles (Figure 7A_3–6_–D_3–6_). Two of our T2VUM splits, SS40867 and SS40868, showed innervation of the DLMs and a single DVM (Figure 14A–B), a pattern matching the blowfly mesVUM-MJ in (Schlurmann & Hausen, 2003). This putative mesVUM-MJ homolog was the cell with the strongest expression in these two splits. SS42385 shows innervation of the DLMs, all DVMs, and the tergotrochanter (tt) muscle. This split may have the same mesVUM-MJ homolog that is present in SS40867 and SS40868, as well as homologs to mesVUM-TT (T2VUM targeting the TTM), mesVUM-PM (T2VUM targeting all power muscles), and a T2VUM which targets specifically DVMs. SS45766 shows innervation of only DVMs and not DLMs (Figure 14D). One line, SS51508, targeted a single VUM neuron whose soma was not in T2 but in the posterior VNC at the most anterior abdominal segment (Figure 14E) and whose dendrites mainly innervate the haltere neuropil. Phalloidin imaging revealed that the VUM targeted in SS51508 innervates a haltere muscle, which may be hDVM, the haltere’s serial homolog of DVM (Figure 14E_4–5_). Note, the SS51508 split line is not completely sparse — it also expresses in a pair of cell bodies in the posterior abdominal ganglion, and we observed a dangling axon in our phalloidin preparation, which could be a severed abdominal motoneuron.

**Figure 15:**
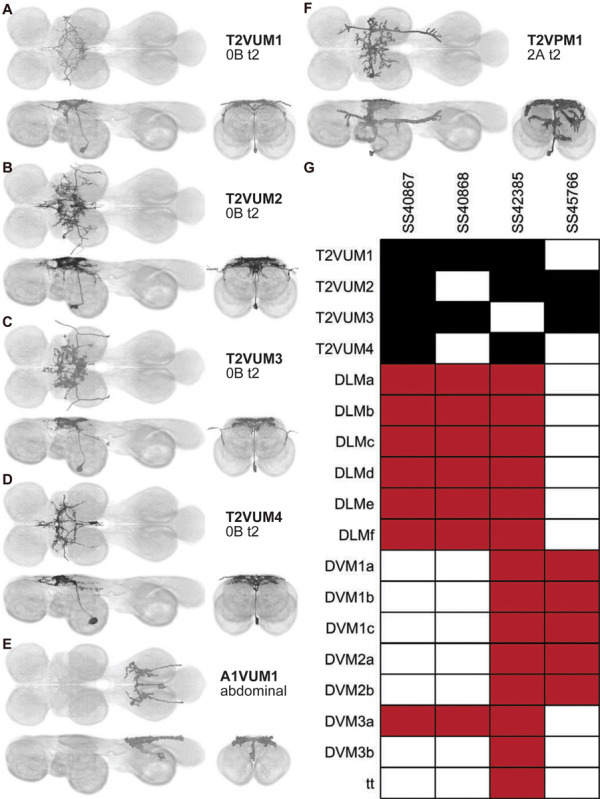
Morphology of individual VUMs and a VPM in our splits, and a table of expression in our T2VUM splits. Segmented multicolor flipout images aligned to the JRC 2018 VNC Unisex template.

We used MCFO to obtain images of individual neurons targeted by our VUM split lines, shown in Figure 15. MCFO often has stronger expression than the GFP reporter line that we used for phalloidin double-labeling experiments, so although the SS40867, SS40868 and SS45766 lines appear sparse in Figure 14A–E_2–5_, MCFO suggests that each of these splits actually has expression in multiple T2VUM neurons (Figure 15G). We did not create any split lines targeting VUMs in T1 or T3. Our splits do not target all T2VUMs; *Drosophila* has at least two more T2VUMs that did not appear in the MCFO of our splits. Figure 15G lists the T2VUMs that we can identify in the VNC MCFO images of each of our splits (black) and the muscle fiber innervation that was observed in phalloidin experiments with our splits (red). Oddly, SS42385 had fewer T2VUMs identified in MCFO but innervated more muscles than SS40867. This may be due to leaky expression in MCFO that causes labeling of cells that do not normally appear in the full expression pattern, or it could be due to weak GFP labeling resulting in DVM and tt innervation being too dim to see in phalloidin experiments.

In addition to the VUMs, we also observed a ventral paired median neuron (VPN) in T2 (Figure 15F, single cell segmented from this pair). The T2VPN1 has similar wing neuropil innervation to the VUMs, but also has additional terminals in T1 and T3.

A dopaminergic T2VUM is active during flight, and dopaminergic T2 neurons are reportedly required for wing coordination in flight (Sadaf et al, 2015). In addition to the classical motoneurons, arthropod muscles are innervated by octopaminergic VUMs. Octopamine increases the twitch tension and relaxation rate of muscle and promotes glycolysis (Evans and O’Shea, 1977; O’Shea and Evans, 1979; Mentel et al, 2003; Ormerod et al, 2013). In *Drosophila* flight, octopamine is critical for endurance (Stocker et al, 2018). In the abdominal ganglia of the locust, VUMs were reported to have only input (Pflüger and Watson, 1995). However, our synaptotagmin labeling of our T2VUM split lines revealed that T2VUMs have not only input but also output within the VNC itself. So VUMs could act on the muscle directly and on other neurons in the VNC by releasing neuromodulators. Because dopaminergic T2 neurons are linked to wing coordination while octopaminergic neurons are necessary for endurance in flight, identifying the neuromodulators released by VUMs could provide insight into their roles in behavior. Octopaminergic VUMs can be distinguished from dopaminergic VUMs based on their expression of tyrosine decarboxylase 2 (TDC2), the enzyme that synthesizes tyramine, the precursor of octopamine. However, tyramine is also a neuromodulator, so we cannot rule out that these VUMs may act by releasing tyramine. Two of our VUM splits, SS40867 and SS40868, used the TDC2-AD split half to restrict expression to octopaminergic neurons and then used different DBD split halves to further limit expression to only some T2 VUMs.

### Interneuron splits and MCFO images of individual interneurons

Dorsal VNC interneurons, which are upstream of efferent motoneurons and VUMs, play crucial roles in executing and coordinating wing behaviors, including take-off, grooming, flight, and courtship song. They remain, however, largely unexplored, in part because they are difficult to access and individually address (Venkatasubramanian & Mann, 2019). Thus, driver lines that target such VNC interneurons will be essential to unraveling the functional organization of the local VNC circuits that give rise to complex wing behaviors.

We generated 143 driver lines targeting a total of 159 dorsal VNC interneurons, opening the door to investigations of the local VNC circuits that control motor behaviors. Supplemental Figure 2 shows segmented images of intrasegmental interneurons which may be involved in processing sensory information or coordinating motor patterns within a neuromere; Supplemental Figure 3 shows segmented images of intersegmental interneurons which connect two or more neuromeres.

#### Naming convention

Our collection includes many interneurons not previously identified. Based on recent nomenclature standards (Court et al., 2020) and in coordination with contemporaneous studies on other interneuron groups in the VNC, we propose the following individual neuron naming scheme. To convey anatomical information in a compact form, our interneuron names contain 1) the primary VNC neuropil region in which the neurites arborize, 2) whether the neuron is unilateral (U) or bilateral (B), 3) whether the neuron is intrasegmental (abbreviated as L for Local, although some intrasegmental neurons may have wide-ranging arborization within a single neuromere and thus not be truly local) or intersegmental (I), and 4) a three-digit integer index. For example, “NUL01” in Supplemental Figure 1 (page 1 top left image triplet) is named for its arborization in the neck tectulum (N), its unilaterality (U), and its locality (L) in the prothoracic neuromere. Table 1 provides a full summary of the abbreviations used in this naming convention.

#### Hemilineage identification

Because the cells in our interneuron collection vary broadly in both their morphology and projection patterns (Supplemental Figures 2–3), we sought methods to categorize cells into groups that would facilitate inferences regarding cell function. A particularly useful method of categorizing neurons in the fly nervous system is to group them by their developmental origin, i.e. hemilineage. During development of the fly VNC, a segmentally repeated array of identifiable neuroblasts undergo division to generate ganglion mother cells, which in turn divide to produce A and B daughter cells (Truman et al., 2010). Thus, each neuroblast gives rise to two distinct lines of progeny—stemming from the A and B daughter cells—which are referred to as hemilineages. e The vast majority of neurons in the adult fly VNC are produced in this fashion, such that these neurons can be unequivocally identified as being a member of a specific hemilineage in the adult CNS.

Grouping VNC cells by hemilineage is a particularly useful method of categorization, as neurons from the same hemilineage share similar projection patterns (Lacin & Truman, 2016; Shepherd et al., 2019), neurotransmitter profiles (Lacin et al., 2019), and even functional significance (Harris et al., 2015; Shepherd et al., 2019)—akin to the functional populations arising from common stem cells in the vertebrate spinal cord (Grillner & Jessell, 2009). Moreover, hemilineage identity provides a compact framework for grouping neuron populations: there are, on average, 34 hemilineages found in each VNC hemineuromere, and the hemilineages found across different hemineuromeres are serially homologous.

Each interneuron in our collection was manually annotated for hemilineage type based on the morphology of hemilineage cell types in the adult fly VNC as described in Shepherd et al., 2019 (Supplemental Figures 2–3). For example, the NUL01 neuron (Supplemental Figure 1A) is a member of the 2A hemilineage—i.e. it arises from an A daughter cell of neuroblast 2—in the prothoracic neuromere of the VNC (t1), and thus was assigned the hemilineage classification “2A t1.”

#### Identifying interneuron input and output sites in VNC neuropil regions

**Figure 16:**
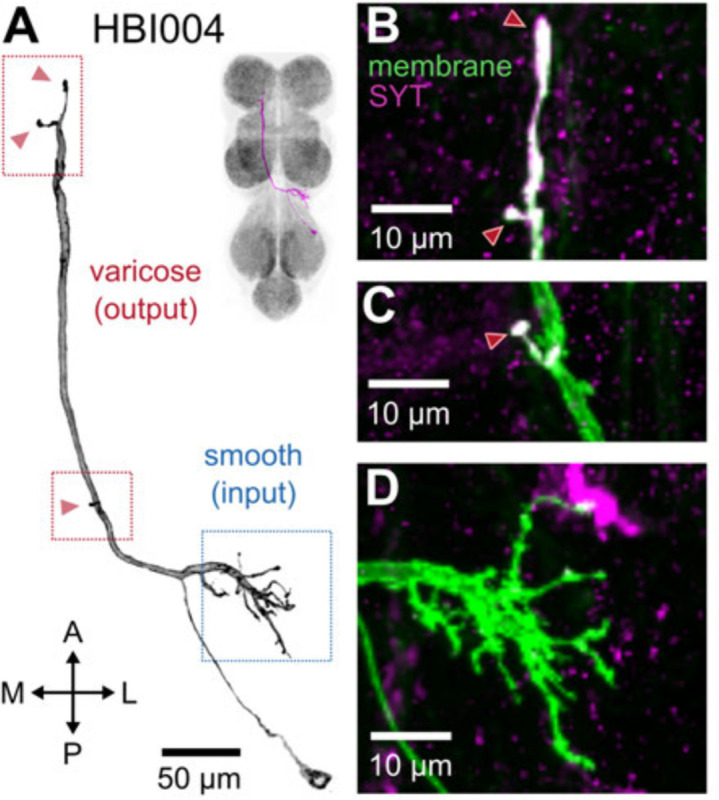
Identifying input and output sites of dorsal VNC interneurons in this study. (**A**) Morphology of example dorsal VNC interneuron HBI004. Neurites are varicose in the neck and wing neuropils (red) and smooth in the haltere neuropil (blue), indicating sites of output and input, respectively. Inset shows the HBI004 neuron overlaid on the structure of the VNC. (**B**-**D**) Close-up images of HBI04 neurites (same regions as highlighted in (**A**)). Staining with both GFP (green) and synaptotagmin (SYT, magenta) confirms varicose neurites as output sites and smooth neurites as input sites.

To characterize the broad connectivity patterns of the interneurons within our collection, we examined neurite projections within the neuropil regions of the VNC (Figure 16. Specifically, we identified the input and output neuropil regions of each interneuron and summarized their aggregate connectivity pattern.

We used two methods to distinguish presynaptic (input) and postsynaptic (output) terminals in each of our interneurons: manual annotation based on neurite morphology (Figure 16A) and imaging of terminals labeled with synaptotagmin, a synaptic vesicle-specific protein (Figure 16B–D). For the former, we distinguished between smooth or varicose neurite terminals and labeled these as input or output sites, respectively (Figure 16A, blue and red boxes; Namiki et al., 2018). For the latter, we labeled terminals that co-stained with synaptotagmin (Figure 16B–D, magenta) as presynaptic (output) and those without co-staining as postsynaptic (input). These two approaches produced consistent results, allowing us to determine the VNC neuropils in which each interneuron had input and output sites (Table 2).

### Inferring connectivity from volume overlap

**Figure 17:**
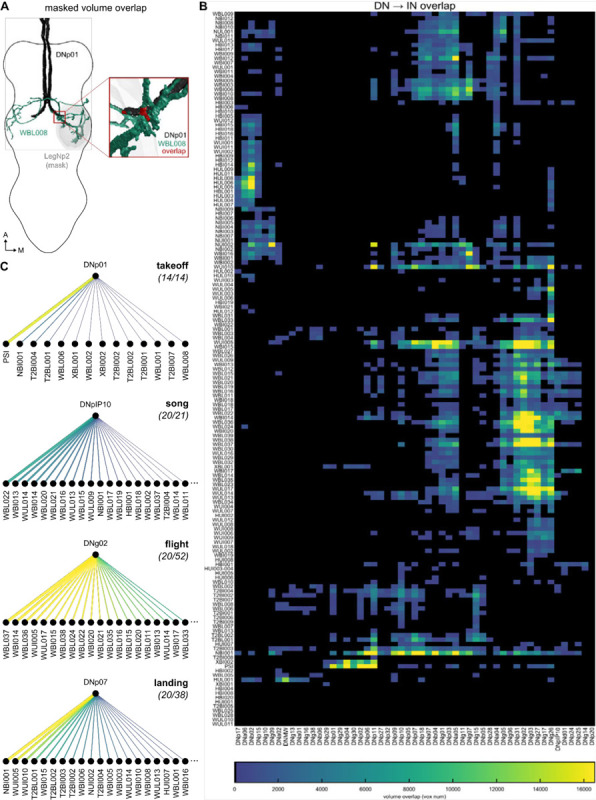
Masked volume overlap between descending neurons and interneurons. (**A**) Illustration of masked overlap calculation. For a given DN and IN pair (DNp01 and WBL008), neuropil regions wherein putative connections could occur are identified based on annotations of input/output neuropil regions. The intersection of neuropil regions wherein the DN has output sites and the IN has input sites constitutes the set of putative connection regions, and these regions are used as a mask to refine calculations of volume overlap between DN and IN images (zoomed inset, right). (**B**) Heatmap showing masked volume overlap between 53 descending neurons and the 163 dorsal VNC interneurons in this study. Color indicates raw overlap voxel count. (C) Tree plots showing putative downstream partners of DNp01, DNpIP10, DNg02, and DNp07 (from top to bottom). Top nodes in plots represent the selected DN; nodes below represent INs with putative anatomical connections based on masked volume overlap. Edge width and color correspond to the number of overlapping voxels between the DN and IN they connect. The number of putative IN connections is capped at 20 in each plot for clarity; text in upper right gives the fraction of overlapping INs plotted (e.g. 20/38 for DNp07, bottom).

To explore the possible functional roles of interneurons within our collection, we leveraged our MCFO images of segmented neurons (Supplemental Figures 2–3), along with findings from recent studies (Namiki et al., 2018), to infer potential anatomical connections between descending neurons (DNs) and our collection of interneurons. As a metric for potential anatomical connectivity, we used image volume overlap, i.e. for a given DN/IN 3D image pair—registered and aligned to the 2018 JFRC Unisex VNC template (Bogovic et al., 2020)—we calculated the number of voxels where the two neuron images overlap (Figure 17A; Stepanyants & Chklovskii, 2005). At the resolution of light microscopy, neuron images can overlap in regions that do not correspond to a synaptic junction; to account for this source of spurious signal, we refined our overlap estimates using the input/output annotations for both INs (Table 2; Figure 16) and DNs (Namiki et al., 2018). Figure 17A illustrates this process of volume overlap refinement, referred to here as masked volume overlap: when comparing a given DN/IN pair, we calculated volume overlap only in neuropil regions that are host to both outputs of the given DN and inputs of the given IN, i.e. neuropil regions that could contain forward synaptic connections between the two cells.

An estimated 350–500 pairs of descending neurons run from the brain to the VNC in *Drosophila* (Namiki et al., 2018), and many previous studies have identified several command-like DNs whose activity is tightly linked with specific behaviors, including walking (Bidaye et al., 2014, 2020), courtship song production (von Philipsborn et al., 2011), takeoff (von Reyn et al., 2014), landing (Ache, et al., 2019), in-flight maneuvers (Suver et al., 2016; Schnell et al., 2017; Namiki et al., 2021), and other behaviors (Cande et al., 2018). We calculated the masked volume overlap between the 159 interneurons and 53 DNs in this study, identifying potential anatomical connectivity between pairs of cells from these two sets (Figure 17B).

Using these masked volume overlap calculations, we identified 1,701 sites of overlap between the DN and IN images in our collection greater than 300 voxels, the approximate size of a synapse in our images (see Methods). To begin associating INs in our collection with potential microcircuits for specific behaviors, we selected example DNs with well-studied behavioral correlates and plotted the top 10–20 INs with the greatest overlap with these DNs (Figure 17C). These example DNs include ones associated with takeoff (DNp01; King and Wyman, 1980; von Reyn et al., 2014), courtship song production (DNpIP10; von Philipsborn et al., 2011), changes in wingbeat amplitude during flight (DNg02; Namiki et al., 2021), and landing (DNp07; Ache, et al., 2019). For each of these example DNs, we identified between 14 and 52 INs with putative synaptic connections, suggesting a role for these INs in the production of behavior.

**Figure 18:**
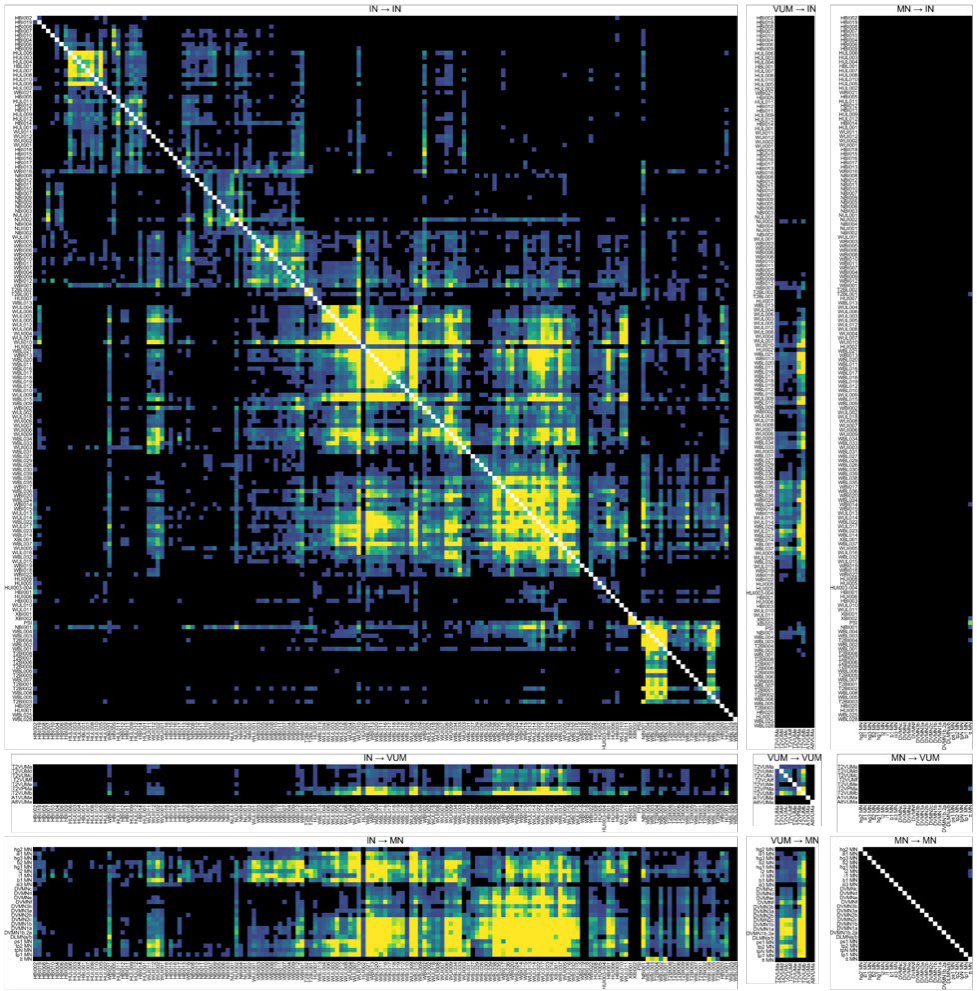
Masked volume overlap between INs, VUMs, and MNs. Block matrices showing masked volume overlap between the interneurons, ventral unpaired median neurons, and wing motoneurons in our cell collection.

**Figure 19:**
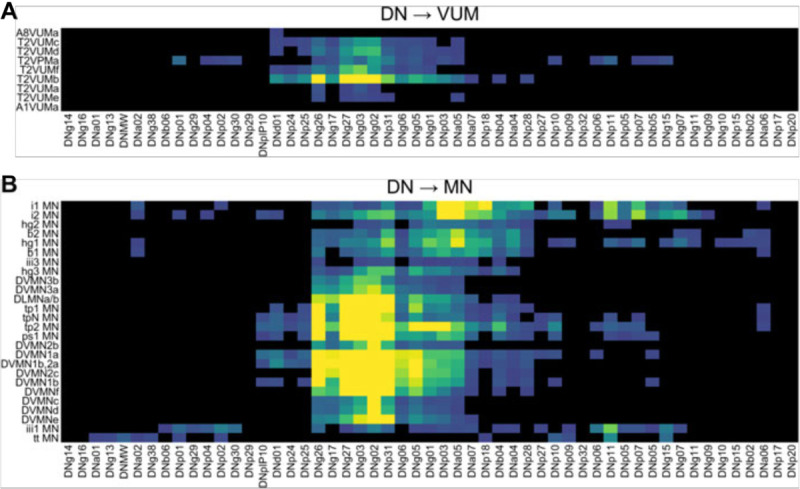
Masked volume overlap between DNs, VUMs, and MNs. (**A**-**B**) Heatmap showing masked volume overlap between DNs and VUMs (**A**) and between DNs and wing MNs (**B**).

To aid future users of these driver lines, we also applied this masked overlap analysis to the INs, MNs, and VUMs targeted by our driver line collection (Figure 18), as well as DN/VUM and DN/MN overlap (Figure 19).

### Clustering interneurons by input/output pattern

**Figure 20:**
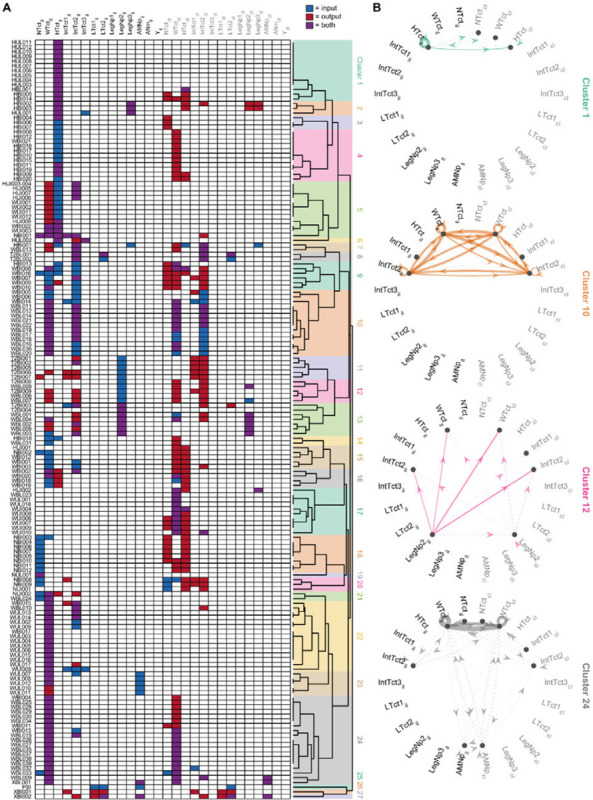
Clustering interneurons based on input/output patterns. (**A**) Matrix plot showing the VNC neuropil regions (columns) that are input and/or output sites for individual interneurons (rows). Filled pixels indicate that the neuron from the corresponding row had an input (blue), output (red), or combined input and output (purple) site in the VNC neuropil region from the corresponding column. Ipsilateral and contralateral neuropil regions are labeled in black and gray, respectively. (**B**) Dendrogram plot shows hierarchical clustering of the neurons based on the Pearson’s correlation applied as a metric to the pattern of input/output sites. Cluster divisions are demarcated by solid color bars and labels, with optimal cluster number determined using a gap statistic (see Methods).

**Figure 21:**
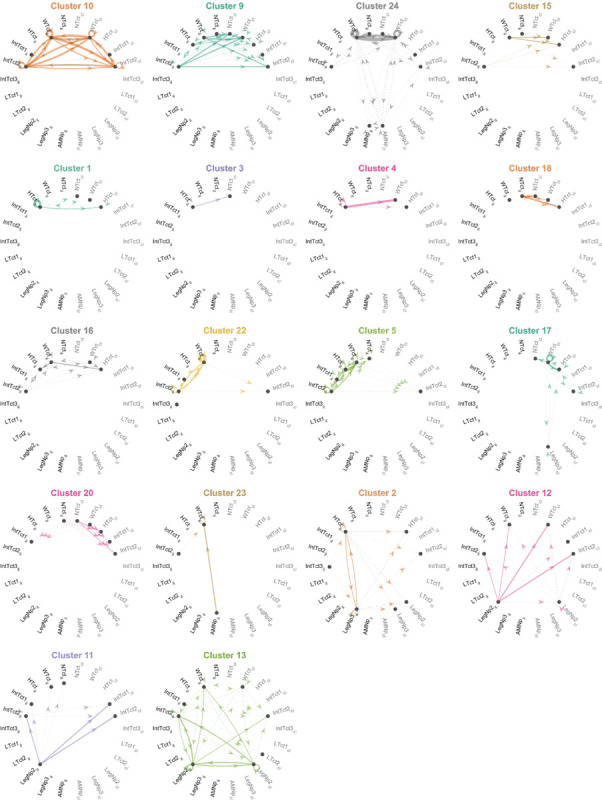
Directed graph plots for interneuron clusters. Directed graph plots as in Figure 20B for all interneuron clusters containing more than two cells.

The overlap analyses in Figure 17B revealed clusters of DN-IN connectivity. This suggests that many of the interneurons in our collection might be organized as microcircuits or clusters of highly interconnected INs. To look for such connectivity motifs, we performed hierarchical clustering of the interneurons using their annotated input/output sites (Figure 20; Methods). These connectivity-based clusters can serve as a first step towards identifying putative functional units within our interneuron collection. Our analysis produced 27 clusters (Figure 20), with each interneuron’s input/output pattern shown as a row in the matrix plot (left) and the similarity between neurons shown with a dendrogram plot (right). We generated directed graph plots to visualize these clusters and illustrate the putative information flow between VNC neuropil regions represented by the interneurons in each cluster (Figure 21).

Across these interneuron clusters, we found three general motifs: 1) broader “local” interneurons that bilaterally linked dorsal and intermediate tectulum in T2 (e.g. cluster 10; Figure 21), 2) feed-forward connections linking dorsal neuropils across the midline (e.g. cluster 4 that takes input from the ipsilateral haltere neuropil and sends output to the contralateral wing tectulum; Figure 21), and 3) projections from the leg neuropil to the dorsal neuropils (e.g. cluster 12; Figure 21).

**Figure 22:**
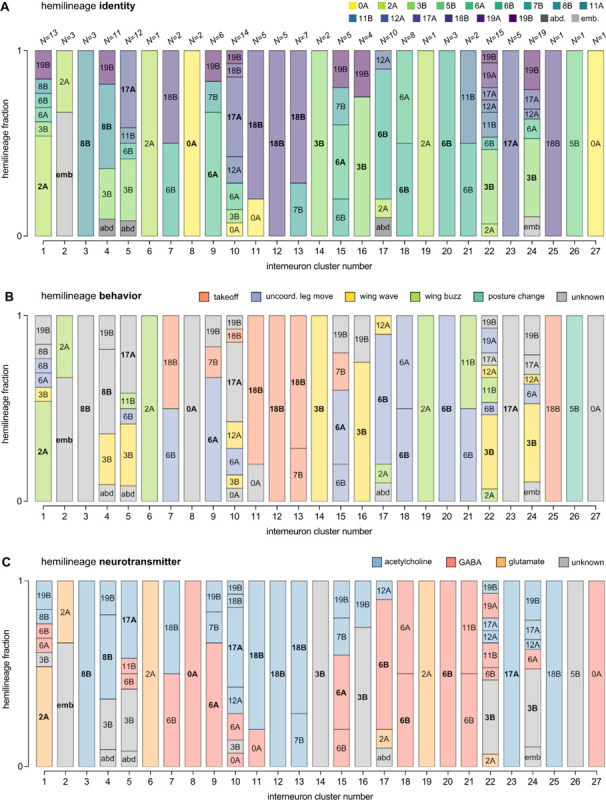
Hemilineage identity of clustered interneurons. (**A**) Bar plots showing fractional hemilineage makeup of each interneuron cluster from Figure 20. Each vertical bar represents one of the 27 interneuron clusters, with total cluster size indicated by numbers at the top. Hemilineages are color-coded according to key (top right) and indicated by labels within bars. Hemilineages that represent the plurality in a given cluster are indicated by bolded labels. The abbreviations “emb.” and “abd.” are used to denote embryonic and abdominal cells, respectively, and are shown in gray. For clarity, data presented here omits distinctions between hemilineages from different hemineuromeres. (**B**) Same data as in **A**, but with hemilineages color-coded by their associated behavior from (Harris et al., 2015). Gray regions indicate hemilineages for which a behavior has not yet been associated. (**C**) Same data as in **A**, but with hemilineages color-coded by their associated neurotransmitter profile from (Lacin et al., 2019). Gray regions indicated hemilineages for which neurotransmitter profiles have not yet been identified.

Hemilineage identity is linked to both anatomical similarity and functional significance (Harris et al., 2015; Lacin & Truman, 2016; Shepherd et al., 2019). To examine the correspondence between this hemilineage identification and our putative connectivity-based clusters, we analyzed the hemilineage makeup of our interneurons clusters obtained from input/output motifs. These two independent methods of grouping interneurons—input/output clustering and hemilineage—provide complementary approaches for meaningfully categorizing the interneurons in our collection, as well as forming hypotheses for the functional roles of subsets of interneurons. Figure 22A shows the hemilineage makeup of each of the clusters defined in Figure 21. While our clustering method did not explicitly contain information about hemilineage identity, we found that several interneuron clusters, e.g. clusters 11–13 (Figure 22A), are primarily composed of cells from one hemilineage, 18B, whose activation has been shown to drive a takeoff (Harris et al., 2015). Other clusters contained cells from a wide range of different hemilineages—for example cluster 1 contains cells from the 2A, 3B, 6A, 6B, 8B, and 19B hemilineages (Figure 22A).

Figure 22B,C show the same intra-cluster hemilineage makeup color-coded by previously reported behavioral correlates (Figure 22B; from Harris et al., 2015) and neurotransmitter profiles (Figure 22C; from Lacin et al., 2019) associated with each hemilineage. As shown in Figure 22B,C, the interneurons comprising clusters 11–13 are all associated with takeoff and are cholinergic, with the exception of one GABAergic cell from hemilineage 0A in cluster 11. Clusters 18 and 20, composed of cells from the 6A and 6B hemilineages, are GABAergic, and associated with uncoordinated leg movements. We interpret from this that some of our connectivity-based clusters may represent functional microcircuits, as aligned with the behavioral predictions from hemilineage data. However, others will need to be investigated to determine their functional significance.

**Figure 23:**
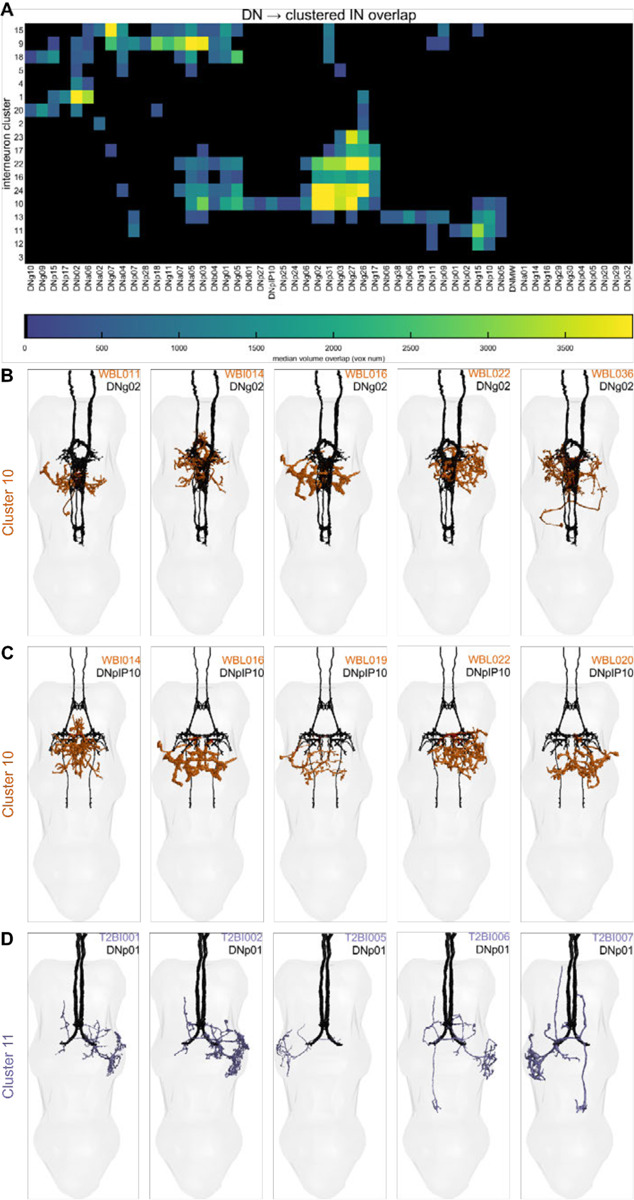
Masked volume overlap between descending neurons and clustered interneurons. (**A**) Heatmap showing masked volume overlap between descending neurons (DNs) and interneurons (INs), with the latter grouped according to the connectivity clusters defined in Figure 20. Color corresponds to median overlap voxel count between a given DN (black) and the set of INs within each cluster. Clusters with 2 or fewer cells are excluded. (**B**-**D**) 5 example INs from a given cluster drawn with selected DNs that show high median overlap with the given IN cluster. Example pairings show cluster 10 interneurons (orange) with DNg02 (**B**), cluster 10 interneurons (orange) with DNpIP10 (**C**), and cluster 11 interneurons (purple) with DNp01 (**D**).

To further generate hypotheses for the function of the connectivity-based clusters, we looked at the putative connectivity of DNs onto these clusters. The heatmap in Figure 23A shows the median volume overlap between DNs and the INs within each cluster. From these, we were able to associate several DNs of interest with IN clusters. This revealed a few clusters of clusters, or superclusters. One particular supercluster stood out, involving IN clusters 23, 17, 22, 16, 24, and 10 and several DNs. DNg02, involved in flight control, was a prominent member of this cluster. This poses the strong hypothesis that the other DNs in this supercluster, which potentially synapse onto similar IN clusters may serve associated roles in flight control. Future work should use the driver lines for INs in these clusters to perform functional experiments in flying flies to test this hypothesis.

### Sexual dimorphism in neuron morphology

**Figure 24:**
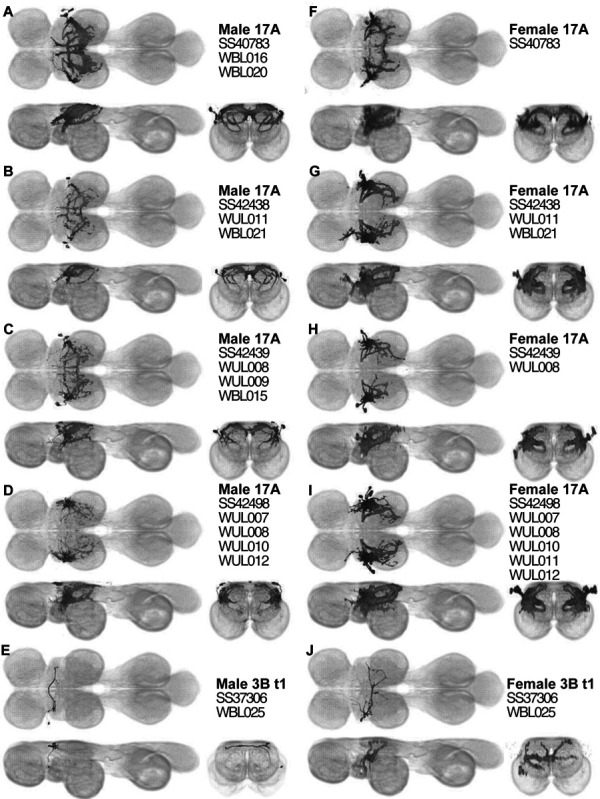
Sexually dimorphic anatomy in split lines targeting two hemilineages. **A** shows the male expression pattern of a split targeting 3B t1, crossed with pJFRC51-3xUAS-Syt∷smGFP-HA in su(Hw)attP1; pJFRC225-5xUAS-IVS-myr∷smGFP-FLAG in VK00005, not aligned **B**-**E** show the male expression patterns of 17A t2 in four different split lines, crossed with pJFRC51-3xUAS-Syt∷smGFP-HA in su(Hw)attP1; pJFRC225-5xUAS-IVS-myr∷smGFP-FLAG in VK00005. **F**-**J** show the female expression patterns of these same lines, crossed with UAS-CsChrimson. All images are aligned to the JRC 2018 VNC template.

Most of the interneurons that we observed were the same in males and females, but some interneurons are sexually dimorphic (Figure 24). We created four splits targeting hemilineage 17A with somata in T2 (SS40783, SS42438, SS42439 and SS42498). The cells labeled in these splits appear to be subsets of a fruitless-expressing cell type called dMS2 (von Philipsborn et al, 2011). We used MCFO to dissect the separate dMS2 / 17A t2 cells in the male (Supplemental Figures 2–3). The male dMS2 cells often have medial neurites that are not present in the female. Another other split line that displayed sexual dimorphism was SS37306, a line targeting a single cell in T2 in the hemilineage 3B t1. In the female, this cell has extensive neurites in T2 that innervate not only dorsal T2 but have more ventral branches as well. In contrast, in the male this cell’s neurites are greatly reduced and are only present in the dorsal-most layer VNC. The neurites of the SS37306 cell in the male also are more anterior, and in the neck neuropil, while the neurites of the female cell are more posterior, and in the wing neuropil.

The driver line VT43702 had previously been reported to drive expression in a cluster of fruitless-expressing interneurons in dorsal T2 called dMS2, which play a role in the production of pulse song in males (von Philipsborn et al., 2011). dMS2 is a cluster of cells, and VT43702 also drives expression in other cell types, so we aimed to produce sparser driver lines targeting only one or a few cells in the dMS2 cluster. However, because our split-screening process used only female images, we could not target the male morphology of dMS2, but we targeted the morphology of the dorsal T2 interneurons seen in female VT43702 flies. We created four splits targeting hemilineage 17A with somata in T2 (SS40783, SS42438, SS42439 and SS42498) whose female morphology was extremely similar to VT43702. After stabilizing the splits, we found that in males most of these splits appear to label subsets of dMS2.

We compared the cells labeled in these four Split-Gal4 lines. The dMS2 cluster contains several neurons. Among the cell types we have identified, seven cell types (WBL015-021) correspond to the dMS2 cluster cells. The Split-Gal4 line SS40783 visualized six pairs of cells in males but four pairs in females. Males labeled two of the dMS2 cells (WBL016 and WBL020) as well as four other cells that we could not precisely identify because of the overlapping arborizations. Whereas the male samples visualize a thick commissural neurites in the wing neuropil and an extended neurites towards AMN (arrows in Figure 24B). The commissural neurites belong to WBL016, while the neurites extending towards the AMN belong to both WBL016 and WBL020. These neurites are apparently missing in females. Given the reduced number of cells labeled in females, a likely explanation is that WBL016 and WBL020 are not labeled in females. An alternative explanation is that these cells may be present in both sexes but with very different morphology.

The line SS42438 labels two pairs of cells in males but three in females. One of the labeled cells is WUL011, which is not in the dMS2 cluster. This cell appears to have relatively similar morphology between sexes; it is a unilateral local wing neuron in both males and females. However, WUL011 appears to have sparser neurites in females and denser, more extensive neurites in males. WBL021 in the dMS2 cluster showed strong dimorphism. In males, the cell is bilateral, but it females, it is transformed into a unilateral neuron (arrows in Figure 24C,D). One additional cell is labeled only in males, but we could not precisely identify its cell type because of the overlapping arborizations.

The line SS42439 labels three pairs of cells in males but two in females. One of the dMS2 cluster cells (WBL015) is labeled in males, but a corresponding cell is not visualized in females. The male samples visualize two more non-dMS2 17A t2 cells (WUL008 and WUL009). Among them WUL008 shows consistent morphology in both sexes. The other female WUL has sparse neurites. The male WUL009 has several dense arborizations approaching the midline, particularly in posterior T2, but also in anterior and central T2. In the female, there are no neurites near the midline in anterior or central T2, and the posterior medial neurites are reduced compared to in the male (Arrows in Figure 24E,F).

Finally, the line SS42498 labels four pairs of cells in males but five in females. Unlike the other four lines, this line only labels unilateral neurons. WUL007, WUL008, WUL010 and WUL012 are labeled in both sexes, whereas WUL011 appears to be labeled only in females. None of them are determined as dMS2 cells, but they showed dimorphism. In females, the neurites of the WULs stay in a very lateral area in central T2, while in males, these WULS have neurites that extend more medially, both in the dorsal wing neuropil and near the AMN.

In addition to these four lines, we identified sexual dimorphism in the Split-Gal4 line SS37306, which labels a single cell in the wing neuropil (WBL025) that belongs to the hemilineage 3B t1. In males, the neurites of WBL025 are rather sparse arborizes in a dorsal layer of the VNC, right at the boundary of the posterior neck neuropil with the wing neuropil. On the contrary, the cell has much more extensive neurites in females, with many ventral branches in the AMN and intermediate tectulum. Whereas the male WBL025 crosses the midline in the neck neuropil, the female counterpart crosses the midline in the intermediate tectulum. The female neurites also lie more posteriorly in females and project more to the wing neuropil instead of neck neuropil.

## Discussion

*Drosophila melanogaster* is a powerful model organism for investigating neural circuits. While detailed studies of the fly brain have yielded deep insight into the neural computations performed therein, studies of the ventral nerve cord have been limited by its relative inaccessibility. However, understanding the anatomy and function of VNC circuitry, as well as how these circuits interact with the brain, are crucial to investigations attempting to link neural computation to behavioral output. To assist in future efforts exploring the function of VNC circuits, we systematically generated and characterized 195 sparse split-GAL4 driver lines (Table 3) that drive expression in a wide range of dorsal VNC cell types, including wing motoneurons, ventral unpaired median neurons, and local VNC interneurons. To our knowledge, this driver line toolkit is the largest collection of genetic reagents sparsely targeting VNC neurons generated to date. These driver lines will allow future studies to image, silence, activate, genetically manipulate, and/or ablate individual neurons in the VNC, providing a powerful probe for investigating the physiology and function of VNC circuits.

Using multicolor flip-out (MCFO), we obtained single-cell images from the expression patterns of these driver lines, thereby isolating and visualizing 198 unique neuron types in the VNC, many of which have not been previously targeted by other split driver lines. Using these single-cell images, we took steps to categorize the broad range of neurons by annotating their patterns of input and output sites, hemilineage identity, and potential anatomical connections with cells from previously described neuronal classes. We envision the characterization of dorsal VNC neurons provided in this study serving as a guide for future investigations that seek to elucidate the detailed function of VNC circuitry.

### Homologous neurons in other insects

Motoneurons directly produce behavioral readouts which can be quantified in order to investigate motor control. Fly wing motoneurons are particularly interesting because they are required for several very different behaviors, such as flight and courtship song; because the role of activity in the MNs innervating the stretch-activated power muscles is unclear; and because the manner in which steering muscles act on the wing hinge or thorax in order to precisely alter the movements of the wing remains mysterious. We created driver lines targeting most wing MNs and visualized the morphology of individual MNs using MCFO.

The morphology of the DLMNs of *Drosophila* has previously been investigated using horseradish peroxidase injection (Coggshall, 1978; Sun & Wyman, 1997). However, little was known about the DVMNs of *Drosophila*; their morphology had never been described. We created sparse driver lines targeting the power MNs and used MCFO to visualize these neurons. We found that the power MNs, including DVMNs, of *Drosophila* are nearly identical to those reported in *Calliphora* (Schlurmann & Hausen, 2007). The most visible difference in DLMN morphology between *Drosophila* and *Calliphora* is that the medial dendrites of DLMNa/b extend further both anteriorly and posteriorly in *Calliphora*, while the medial dendrites in *Drosophila* are restricted to a narrower area on the anterior-posterior axis. Thus the power MNs of *Calliphora* are scaled up but have extremely similar features to *Drosophila* power MNs. This suggests that the anatomical features of power MNs are conserved in Diptera.

Power muscles and motoneurons associated with the front wing have been described in more distantly related insects, particularly in Lepidoptera, making some comparisons across orders possible. In moths, as in *Drosophila*, the dendritic arbors of power MNs are largely located in the dorsal nerve cord (Kondoh & Obara, 1982; Agee & Orona, 1988; Duch et al., 2000; Ando et al., 2011). The dorsal area where the dendrites of power MNs are located receives input from behaviorally-significant sensory systems, including from the wing campaniform sensilla in both flies and moths (Ando et al., 2011), from haltere campaniform sensilla in flies (Bartussek & Lehmann, 2018), from the tympanic nerves in moths (Agee & Orona, 1988). Direct mechanosensory inputs to the wing MNs contribute to precise flight control, while acoustic input enables moths to reflexively perform evasive maneuvers in response to the ultrasonic vocalizations of insectivorous bats (Roeder, 1964).

In *Drosophila*, the single DLM muscle runs the entire length of the thorax (Figure 2A_4–5_). In moths, a DLM muscle extends throughout the thorax (Hanegan & Heath, 1970; Kondoh & Obara, 1982; Komai, 1998; Duch et al., 2000). The DLMNs innervating this muscle are similar in moths and *Drosophila*. In both the moth and in *Drosophila*, the somata of the MNs that innervate the four ventral fibers of this DLM form a closely apposed cluster on the ventral side of the nerve cord, ipsilateral to their axons (Kondoh & Obara, 1982; Agee & Orona, 1988; Duch et al., 2000; Ando et al., 2011; Figure 2C–F). The dorsal fiber of the moth DLM is innervated by the only DLMN whose soma is contralateral to its axon, and more dorsal than the somata of the ventral fiber DLMNs (Kondoh & Obara, 1982; Agee & Orona, 1988; Duch et al., 2000; Duch & Levine, 2000; Ando et al., 2011). Likewise, the two most dorsal fibers of the Diptera DLM are innervated by the sole DLMN with a contralateral, dorsal soma (Figure 2B).

There are major differences between fly and moth DLMNs. Most of the dendrites of moth DLMN1e are ipsilateral to its axon and contralateral to its soma (Kondoh & Obara, 1982; Duch & Levine, 2000; Ando et al., 2011). In contrast, the equivalent DLMNa/b in *Drosophila* has a symmetric arbor, with major innervation on each side. A functional difference between moth and fly power MNs is that moth power MNs receive direct acoustic input enabling moths to turn away from bats reflexively, so perhaps the asymmetry of the moth DLMN1e aids in this steering response. Moth DLMNe dendrites are located far posterior to DLMNa-d (Kondoh & Obara, 1982; Ando et al., 2011). The corresponding *Drosophila* DLMNc-f arbor largely overlaps with that of DLMNa/b. The silkmoth has two additional DLM muscles, unlike any fly muscles, which do not act as wing depressors and whose function is not known (Kondoh & Obara, 1982). Likewise, the silkmoth has three more DLMNs than flies.

In *Drosophila*, wing elevation is powered by three DVM muscles; the haltere is also associated with an hDVM muscle. Saturniid moths have two sets of DVM muscles: one in the anterior thorax and one in the posterior thorax (Hanegan & Heath, 1970). However, the anatomy of DVMNs of saturniid moths has not been described. The anatomy of silkmoth DVMNs has been described, but only for the anterior thorax DVMNs, which are homologous to the DVMNs of *Drosophila*. Moths have more DVM muscles but fewer DVM fibers than *Drosophila*. *Drosophila* has three DVM muscles, with three, two and two fibers. Moths have five DVM muscles per wing; the anterior-most muscle has two fibers and the rest have one fiber. The DVMs of the silkmoth anterior thorax have nine DVMNs, while *Drosophila*’s wing DVMs have seven DVMNs.

Moth DVMNs tend to have very different morphology from fly DVMNs. The general shape of the arborization of silkmoth anterior DVMN1 and the location of their somata somewhat resemble fly DVMN1a-c. Both of these sets of MNs have three medial arbors: anterior, middle and posterior. However, the moth DVMN1 arborization extends laterally almost to the edge of the nerve cord, while fly DVMN1a-c arborization is much more medial. They both have somata that are located anteriorly.

DVMN2 of the silkmoth anterior thorax also somewhat resembles fly DVMN1a-c; they both have a cell body fiber that projects posterior dorsally to the arborization. However, the arborization is shaped differently in other ways; for example, the arborization of fly DVMN1a-c crosses the midline while the arborization of moth DVMN2 does not.

DVM3 of the silkmoth anterior thorax is innervated by three MNs that do not resemble any fly power MN. The silkmoth DVMN3 have small dorsal somata ipsilateral to their axons, while all fly power MNs have ventral somata except DLMNa/b, which has a giant contralateral dorsal soma. The silkmoth DVMN3 have arborization extending throughout a broad region in the middle of the nerve cord, reaching much more ventrally than other silkmoth power MN dendrites, while all fly wing MNs have largely dorsal arborization.

DVMN4,5 of the silkmoth anterior thorax resemble DVM2a,b: they both have their somata posterior to their dendrites, unlike other power MNs. Again there are also differences, for example that the fly MN arborization crosses the midline but the moth MN arborization does not.

Thus, DVMN anatomy of different orders of insects tends to be very different. This suggests that the morphology of the DVMNs has changed dramatically over the course of evolution, in contrast to the DLMNs, which appear to have somewhat conserved morphology even between different orders.

The morphology of many *Drosophila* steering MNs has been reported in previous studies (Trimarchi & Schneiderman, 1994; O’Sullivan et al., 2018). We are the first to describe the anatomy of the hg3 MN in the VNC and to produce sparse driver lines targeting this MN. We used one of these sparse driver lines to investigate the function of hg3 MN in courtship song, a behavior in which two other hg steering muscles, hg1 and hg2, are required for sine song. We found that in contrast to hg1 and hg2, hg3 plays no role in sine song, put rather may be involved in fast pulses. The morphology of i1 MN has previously been described (Trimarchi & Schneiderman, 1994), but we created the first sparse driver line targeting this MN, and used it to investigate the role of i1 MN in several parameters of tethered flight. We did not find any effect of activating i1 MN on the flight parameters that we examined, but this driver line will likely be useful for exploring the function of i1 MN in other behaviors or in other parameters of flight.

The morphology of steering MNs may vary dramatically between different Dipteran genera. For example, in *Drosophila*, b1 MN has a relatively simple morphology, with two long major fibers: one that projects across the midline to the contralateral side and one that projects posteriorly (Figure 6A, C). In contrast, b1 MN in *Calliphora* has a completely different morphology: the posterior main branch found in *Drosophila* appears to be absent in *Calliphora*, and instead, the *Calliphora* b1 MN has numerous relatively short branches projecting both anteriorly and posteriorly (Fayyazuddin & Dickinson, 1996). Thus, the b1 MN of *Calliphora* more closely resembles i2 MN in *Drosophila* than b1 MN in *Drosophila*.

Such differences in morphology raise the question of whether homologous steering MNs in different Dipteran species receive different input from interneurons or sensory neurons in the VNC, which could lead to differences in the motor systems between species. High resolution electron microscopy data of the *Drosophila* VNC may shed further light on this subject. However, some haltere sensory input to b1 MN has been shown to be similar in *Drosophila* and *Calliphora* (Trimarchi & Murphey, 1997).

Many steering muscles, including b1, show similar patterns of activation across different Dipteran genera. In both *Calliphora* and *Drosophila*, b1 MN fires a spike in phase with each wing beat, and the phase of b1 MN’s firing advances on the outer side when the fly turns (Dickinson & Tu, 1997). However there are differences in flight between different genera. In *Drosophila*, the degree of pronation of the wing does not vary and may not be an important flight control parameter, while in calypterate flies, the degree of pronation is an independent parameter that could be used for longitudinal control and may increase on the inside wing when the fly turns (Taylor, 2001). The typical wingbeat frequency of *Calliphora* may be half the rate of *Drosophila* (Bartussek & Lehmann, 2018). In some behavioral contexts, haltere sensory input appears to be much more important for *Calliphora* than *Drosophila*. *Calliphora* oscillates its halteres when walking, and uses its halteres to stabilize takeoff (Yarger et al., 2020), while *Drosophila* does not. Possibly the differences in steering MN morphology between species are related to their differences in behavior.

The extent of the differences in steering MNs between *Calliphora* and *Drosophila* may also vary from one steering MN to the next. The difference in morphology in b1 MN between the two genera is striking. In contrast, the overall morphology of tt MN is similar between the two genera; tt MN has the same major branches in both. A possible difference between the two genera is that tt MN in *Calliphora* has many very thin, complex sub-branches splitting off from its major branches (Strausfeld et al., 1984). These fine sub-branches are not visible in *Drosophila*.

In Diptera, the hindwings have evolved into club-shaped halteres, which have their own reduced set of muscles. The morphology of the haltere MNs of *Drosophila* have not been described in detail before. We created driver lines targeting haltere MNs and used MCFO to visualize six haltere MNs.

It may be informative to compare the haltere MNs with the MNs of the muscles associated with the hindwings in other insects, such as Lepidoptera. Moths have two pairs of wings, but the hindwings are smaller than the forewings and are mechanically linked to them, in contrast to more distantly related insects like locusts and dragonflies, which have well-developed hindwings that flap out of phase with the forewings. Moths have power muscles that are similar to fly power muscles in many ways. The projection patterns of sensory neurons from the fore- and hindwings of the moth are similar to those of campaniform sensilla from the wings and halteres of flies (Ando et al., 2011). Given all these similarities in the flight system, moth and fly MNs may be expected to have some similarities as well.

MNs of the hawkmoth hindwing DLMNs have their dendrites in a dorsal layer of the nerve cord, with their cell bodies more ventral, the same as the haltere MNs of *Drosophila* (Ando et al., 2011). Like hDVMN, hi1 MN, and hi2 MN in *Drosophila*, the dendrites of the hindwing MNs in the hawkmoth appear to be close to where the axon exits the nerve cord. On the other hand, the somata of the hawkmoth hindwing MNs also appear to be close to the dendrites and axon on the anterior-posterior axis (Ando et al., 2011), while in *Drosophila*, the somata of haltere MNs are usually far posterior to their dendrites and axon. So *Drosophila*’s haltere power muscle MN, hDVMN, has some anatomical similarities but also differences with hawkmoth hindwing power muscle MNs.

One of the interneurons targeted by our splits is the PSI. The PSI is important for escape, along with the tt MN that triggers jumping and the DLMNs that drive wing depression. The PSI is unique in that its axon terminals are not within the central nervous system, but rather its synaptic output is in the peripheral nerve (King & Wyman, 1980). Signals from the PSI need not travel through the dendritic arbors of the DLMNs but can immediately activate the fibers of DLMNs within the peripheral nerve, thus producing a much more rapid effect, in a behavior, escape, in which reactions must be as fast as possible.

The PSI has also been described in *Calliphora* (Strausfeld *et al.*, 1984). However, the anatomy of the PSI is very different between the two genera. The PSI of *Drosophila* has only medial dendrites within the VNC, while the PSI of *Calliphora* also has lateral dendrites. The dendrites of the *Drosophila* PSI project fairly far anteriorly into T1 and posteriorly. In contrast, the dendrites of *Calliphora* PSI are restricted to a narrow area on the anterior-posterior axis. This is surprising, considering that other parts of the escape circuit, the DLMNs and giant fiber have extremely similar morphology in *Drosophila* and *Calliphora*, and that tt MN also has more similar anatomy in *Drosophila* and *Calliphora*.

*Calliphora* has eight to ten contralaterally projecting haltere interneurons (cHINs) that receive input in the haltere campaniform sensilla and are coupled to neck motoneurons (Strausfeld & Seyan, 1985). We visualized four types of similar cHINs. Because these neurons mainly innervate the haltere neuropil and are bilateral and intersegmental, we named them HBI004-007. It is difficult to determine how many cells of each type exist, whether each type represents a single cell on each side or a couple of cells.

All of these *Drosophila* cHINs have different morphology than *Calliphora* cHINs. Different haltere interneurons such as cHIN types may receive input from different populations of sensilla and thus respond to different types of haltere motion (Yarger & Fox, 2018). However, the dendrites where cHINs receive input in the haltere neuropil have extremely similar morphology in *Calliphora* and *Drosophila*. On the other hand, in the neck neuropil, *Calliphora* cHINs have many fine, thin branches. In contrast, each *Drosophila* cHIN innervates the neck neuropil with just one or two varicose terminals. This may suggest that the *Drosophila* cHINs have different connections in the neck neuropil than *Calliphora* cHINs.

cHINs can be distinguished as n-cHINs that innervate the neck neuropil (Strausfeld & Seyan, 1985) versus w-cHINs that innervate the wing neuropil (Trimarchi & Murphey, 1997). In our interneuron collection, HBI004-007 are n-cHINs, while HBI008-HBI012 and HBI017-HBI020 are w-cHINs. In *Drosophila*, some w-cHINs, possibly HBI008-HBI010, are coupled to b1 MN (Trimarchi & Murphey, 1997). *Calliphora* also has w-cHINs (Hengstenberg, 1991).

### Quantification of behavior using driver lines

A benefit of studying the neural circuitry of the VNC is that its outputs largely take the form of behaviors that are amenable to quantification. Using a subset of our driver lines—those targeting wing motoneurons—we established mappings between the manipulation of individual wing muscle activity and specific changes in the performance of both tethered flight (Figure 10) and courtship song (Figure 11), similar to findings reported in previous studies (Lindsay et al., 2017; O’Sullivan et al., 2018). More broadly, our collection of sparse driver lines, combined with the sophisticated genetic toolkit for *Drosophila* (Venken et al., 2011; Simpson & Looger, 2018), offers the opportunity to investigate the consequences of targeted neural manipulations on the broad range of behaviors controlled through the VNC. The confluence of recent advances in both experimental and computational techniques allows large-scale behavioral screens wherein complex behavioral outputs are both automatically and robustly quantified (Cande et al., 2018; Williamson et al., 2018; Seong et al., 2020). In combination with the driver lines presented in this study, such advances in large-scale behavioral quantification could facilitate rapid testing for the involvement of VNC neurons in the execution of an investigator’s behavior of choice.

Because our driver line collection largely focuses on regions of the VNC involved in wing behaviors, the utility of this collection for investigating the neural substrates of behavior is particularly apparent in the realm of *Drosophila* flight. Tethered flight experiments—like the ones performed in this study—are an invaluable tool for linking neuronal circuitry to flight phenotypes (Suver et al., 2016; Lindsay et al., 2017; Schnell et al., 2017); however, a comprehensive understanding of flight’s underpinnings will require more naturalistic assays, in which flies are allowed to freely traverse 3D space. A sophisticated set of tools already exists for quantifying subtle, complex flight behaviors (Ristroph et al., 2010; Beatus & Cohen, 2015; Dickinson & Muijres, 2016); the addition of genetic reagents that will allow targeted manipulation of small populations of neurons in untethered flight assays will open the door to a new level of detail with which the neural substrates of flight can be investigated.

### T2VUM morphology

Two of our T2VUM splits, SS40867 and SS40868, showed innervation of the DLMs and a single DVM (Figure 14A–B), a pattern matching the blowfly mesVUM-MJ (Schlurmann & Hausen, 2003). The morphology of mesVUM-MJ in Calliphora resembles T2VUM1 in Drosophila: MesVUM-MJ has a curved main fiber similar to T2VUM1 and both neurons have extensive dense, fine arborization like a cloud around the main fiber. T2VUM1 is labeled in both SS40867 and SS40868. T2VUM1 in Drosophila may correspond to mesVUM-MJ in Calliphora.

SS42385 shows innervation of the DLMs, all DVMs, and the tergotrochanter (tt) muscle. The innervation pattern suggests that SS42385 may label homologs to blowfly mesVUM-TT (T2VUM targeting the tt muscle) and mesVUM-PM (T2VUM targeting all power muscles). SS42385 labels T2VUM2, which has a horizontal main fiber similar to mesVUM-TT. SS42385 labels T2VUM4, which has a curved main fiber similar to mesVUM-PM. The rest of the neurites of T2VUM4 and mesVUM-PM also match well: they both have fibers that project straight anteriorly and posteriorly along the midline, more laterally they both have arborizations projecting outwards from the midline like the points of a star, and neither of them have much arborization immediately around the main fiber.

The morphology of T2VUM1 matches mesVUM-MJ well and the morphology of T2VUM4 matches mesVUM-PM well, but mesVUM-TT does not match T2VUM2’s morphology as well. mesVUM-TT has similar medial arborization to T2VUM2- straight anterior and posterior fibers along the midline and a nest of dense fibers all around the central wing neuropil- but mesVUM-TT also has extensive lateral arborization that projects into T1 and T3, while the lateral arbors of T2VUM2 are greatly reduced. Perhaps the morphology of mesVUM-TT is not as well conserved between species. This is surprising because the tt muscle is important for escape, a behavior often necessary for survival, and because the tt MN morphology is largely conserved.

SS45766 shows innervation of only DVMs and not DLMs (Figure 14D). This does not correspond to any known Calliphora VUM. In the VNC, SS45766 labels T2VUM2 and T2VUM3, two cells whose morphology resembles each other extremely strongly. One SS45776 MCFO preparation also showed expression in T2VUM1.

One line, SS51508, targeted a single VUM neuron whose soma was not in T2 but in the posterior VNC at the most anterior abdominal segment (Figure 14E) and whose dendrites mainly innervate the haltere neuropil.

### Developmental origins of VNC neurons

As the insect analog of the vertebrate spinal cord, the VNC offers the potential for investigating the principles governing development-function links with wide applicability. The motor control systems of the spinal cord include highly diverse populations of interneurons which are organized into cardinal classes based on their common ontogeny and function (Grillner & Jessell, 2009; Arber, 2012). Similarly, the majority of VNC cells can be assigned to a specific hemilineage, with cells in a given hemilineage sharing similar functional and anatomical characteristics (Harris et al., 2015; Lacin et al., 2019; Shepherd et al., 2019).

In this study, we annotated the hemilineage identities of all ventral unpaired median neurons and interneurons targeted in our driver line collection using single-cell MCFO images (Figure 15, Supplemental Figures 2–3). In addition to providing insights into the putative functions of these neurons, this hemilineage labeling confers two significant benefits for the translational applicability of VNC research. First, hemilineage organization is shared across insect species (Shepherd et al., 2019), allowing results obtained from the study of our driver lines to inform the study of other insect nervous systems. Second, the homology to the vertebrate spinal cord will facilitate comparisons between the development-function links in the tractable *Drosophila* CNS and a broader range of vertebrate model systems. Such cross-taxa comparisons have the potential to elucidate general principles of motor system organization, and our driver line collection offers a unique toolkit to pursue such questions.

In aggregate, our interneuron collection contains cells from 14 VNC hemilineages: 0A, 2A, 3B, 5B, 6A, 6B, 7B, 8B, 11B, 12A, 17A, 18B, 19A, 19B. Consistent with our goal of producing driver lines targeting wing control circuitry, the hemilineages represented in our interneuron collection primarily arborize either solely in dorsal VNC neuropil regions (2A, 6A, 6B, 11B) or bridge connections between ventral and dorsal VNC neuropils (0A, 3B, 5B, 7B, 8B, 12A, 17A, 19A, 19B) as might be involved in take-off behaviors (Shepherd et al., 2019). By analyzing single-cell images, we found that many hemilineages which innervate both dorsal and ventral regions of the VNC include neurons which arborize only in the dorsal VNC. The hemilineages which have been reported to connect the dorsal and ventral VNC do contain interneurons which connect different layers of the VNC, but they also include solely dorsal interneurons, and presumably solely ventral interneurons which are not included in this study. Thus our sparse driver lines and single-cell analysis revealed diversity within hemilineages which had not previously been reported. In addition, activation experiments from previous studies have linked the hemilineages in our interneuron collection to several behaviors (Harris, et al., 2015), including wing buzzing (2A, 7B, 11B, 12A, 18B), wing waving (3B, 12A), takeoff (7B, 11B, 18B), postural changes (3B, 5B, 8B), and uncoordinated leg movements (6A, 6B). The confluence of anatomical and behavioral evidence suggests that the hemilineages represented in our interneuron collection are likely to represent discrete populations involved in the control of wing behaviors; future investigations involving these interneurons can leverage this hemilineage identification to probe their functional significance.

### Hypothesis generation for VNC circuit investigation

A goal of this study was to generate functional hypotheses about the roles of previously unstudied neurons targeted in our driver line collection. While cells like the wing motoneurons allow a natural link to behavioral function, many of the interneurons targeted by our driver lines have not been previously described, and their functional roles are more difficult to ascertain based solely on anatomy. Such hypotheses will necessarily be limited in scope, due to both resolution limits of our light-level connectivity annotations and the lack of functional recording data; however, cluster-based hypotheses may nevertheless be useful as a guide in future studies of this largely unexplored region of the fly nervous system.

To assist in generating hypotheses regarding the less thoroughly understood interneurons, we clustered these cells based on their patterns of inputs and outputs within the VNC (Figure 17). This clustering method provides a tractable description for the 160 interneurons targeted in our driver line collection, as well as a means to infer functional roles for interneuron groups. To further inform functional hypotheses arising from this clustering, we leveraged knowledge from previous studies regarding the links between hemilineage identity and function (Figure 22), and inferred potential anatomical connectivity between interneurons and a set of descending neurons relaying signals from the brain to the VNC (Figure 23).

Collectively, these analyses create a framework for informing future studies of the VNC circuitry controlling complex behaviors like takeoff, courtship, and flight control. For instance, we postulated above that two clusters of interneurons—clusters 1 and 2 (Figures 20, 21)—were likely involved in the execution of takeoff. Our reasoning for this hypothesis was that both clusters 1) contained interneurons connecting mesothoracic leg neuropils to both the intermediate and wing tectula, 2) were largely comprised of cells from hemilineage 18B (Figure 20), which is known to be involved in takeoff (Harris et al., 2015; Lacin et al., 2020), and 3) exhibited potential anatomical connections with the giant fiber, DNp01 (Figure 22B), which is known to drive fast takeoff escape responses (von Reyn et al., 2014). Researchers hoping to leverage our driver line collection to study the neural substrates of takeoff can use this reasoning to investigate the interneurons in clusters 1 and 2 as a starting point; alternatively, the same deductive approach could be used to identify other putative takeoff-related interneurons. Importantly, while the circuits governing behaviors like takeoff are expected to include neurons in both the dorsal and ventral VNC—the latter of which was not the focus of this study—due to the need for leg-wing coordination to properly execute this motor sequence, the driver lines in this study, along with the methodology for forming early hypotheses, can still be used as an entry point for examining the interneuron circuitry controlling such behaviors. Moreover, the methodology described here can be used with forthcoming driver line collections that target ventral VNC neurons, providing a richer set of preliminary hypotheses to guide future investigations.

A similar line of reasoning can be employed to generate hypotheses regarding interneurons involved in courtship song or flight control. In the case of courtship song, interneurons in our cluster 4 (Figure 21) exhibit connections between wing and intermediate tectula, are partially composed of cells from the courtship-related hemilineage 12A (Shirangi et al., 2016), and have potential anatomical connections with the song command neuron DNpIP10 (Figure 23A). In the case of flight control, where less is known about the relationship between hemilineage and behavior, and fewer descending neurons have been definitively linked to flight maneuvers, additional lines of evidence may be necessary to identify promising interneuron groups for investigation. However, interneurons clusters with strong connections among the neck, haltere, and wing tectula, and that have potential anatomical connectivity with dorsally projecting DNs—e.g. interneuron clusters 8, 10, and 11—still provide a strong starting point for investigating the neural substrates of flight control.

The above constitute three examples of hypotheses that can be generated using the driver lines and analyses presented in this study. Future researchers can make use of these guides, as well as use the tools presented here to formulate hypotheses regarding putative involvement of interneurons in any behavior likely controlled by circuits in the dorsal VNC. Importantly, while hypotheses generated in this manner can serve as useful guide in the initial stages of future research, additional lines of evidence—as can be obtained through behavioral testing, functional recording (Chen et al., 2018), or high-resolution electron microscopy data (Phelps et al., 2021)—will be necessary to rigorously test any hypotheses generated in this study.

### Compatibility with the *Drosophila* neurobiology toolkit

The resources and analytic approaches presented in the study will be greatly complemented by the burgeoning set of genetic tools, experimental techniques, and novel data sets available for the investigation of the *Drosophila* central nervous system. As alluded to above, the use of driver lines in combination with tools that allow transient or chronic manipulation of genetically targeted neurons will be an invaluable resource for linking neural activity in the VNC to behavior (Venken et al., 2011; Simpson & Looger, 2018). In cases where our driver lines target multiple cell types, techniques for systematically reducing driver line expression patterns will help hone the results of such efforts (Isaacman-Beck et al., 2020). Similarly, expressing new, advanced calcium indicators in the cells targeted by our driver lines (Zhang et al., 2020) and utilizing recently developed techniques for monitoring neural activity in the VNC (Chen et al., 2018), will facilitate a greater understanding of the temporal dynamics of VNC circuitry. Genetic techniques for synaptic tracing in combination with our driver lines (Talay et al., 2017; Cachero et al., 2020) or matching our segmented light microscopy images to electron microscopy reconstructions of the fly VNC (Phelps et al., 2021) will allow more rigorous tests of connectivity and assist in circuit-level analysis. Finally, just as a collection of descending neuron reagents (Namiki et al., 2018) was used in this study as means to better understand putative roles of VNC interneurons, the increasing number of such driver line sets can be used in combination with the one presented here to gain a richer understanding of how neural circuits function at the scale of the full CNS.

The above represent just a few examples of how we envision the resources and results presented here may aid in the study of *Drosophila* neurobiology as a whole. With the continuing advent of new tools and techniques, we are poised to investigate the fly central nervous system in unprecedented detail, an effort that has already borne impressive results. It is our hope that, in sharing these resources and hypothesis-generating tools with the community, we can contribute to this grander endeavor.

## Materials and Methods

### Fly Stocks

Split-GAL4 driver lines stocks were generated as described below and maintained as homozygous stocks. For experiments, the driver lines were crossed to one of the following effector lines: 20XUAS-CsChrimson-mVenus trafficked in attP18 for activation experiments and screening VNC expression; w;UAS-Kir2.1-GFP(III) or w+ DL; DL; pJFRC49-10XUAS-IVS-eGFPKir2.1 in attP2 (DL) for courtship song silencing experiments with Kir; w+; UAS-TNTe for courtship song silencing experiments with tetanus; or pJFRC7-20xUAS-IVS-mCD8∷GFP in attP2 for imaging innervation of muscles with phalloidin staining. The progeny from these crosses were reared on cornmeal-based food in a 25°C incubator with a 16:8 light cycle.

### Generation of split Gal4 lines targeting VNC neurons

14,284 confocal stacks of the VNCs of female Janelia Generation1 GAL4 flies (Jenett et al. 2012) were registered to a common template. For alignment, confocal stacks of the VNC were converted to an 8-bit nrrd file format. The reference channel, which used the nc82 antibody against Bruchpilot to label neuropils, was used to normalize contrast across samples. Each stack was rotated so that the anterior-poster axis was vertical, and the reference channel was aligned to a template by nonrigid warping using the computational morphometry toolkit. VNC images were aligned to the JRC2018_VNC_UNISEX template. The signal channel was then transformed using the warped mesh.

Each of these aligned stacks was then used to generate a maximum intensity projection (MIP), in which the color of the signal encoded its depth in the dorsal-ventral axis, called a Color MIP. Masks were drawn around neurites of targeted wing motoneurons in these Color MIPs and used to search the entire collection for a match using a custom-written FIJI plugin (Namiki et al., 2018; Otsuna et al., 2018). This process generally narrowed down the number of possible matching expression patterns to a few hundred.

Over the course of this project, we selected 1,658 AD/DBD split GAL4 intersections to screen. Split intersections were screened by crossing them with 20XUAS-CsChrimson-mVenus trafficked in attP18, dissecting out the central nervous system of 3–10-day old female offspring, immunolabeling against mVenus and nc82, embedding the preparations in DPX, viewing them with a Zeiss fluorescent microscope and then imaging selected preparations with a confocal microscope with the 20x objective. Most of the split intersections either had broad expression or dim expression. Of the splits, we selected 193 to be stabilized by making them homozygous for the AD and DBD transgenes. For stabilized splits, we used multi-color flip out to stochastically label individual neurons. Images of CsChrimson-mVenus expression patterns and of multi-color flip out were aligned and used to produce more color MIPs which could be used to search for sparser splits. Images were segmented using Amira or VVDViewer (https://github.com/takashi310/VVD_Viewer).

### Polarity labeling to visualize output sites

After split intersections were stabilized, they were crossed with pJFRC51-3xUAS-Syt∷smGFP-HA in su(Hw)attP1; pJFRC225-5xUAS-IVS-myr∷smGFP-FLAG in VK00005. The central nervous system of 3–10 day old male offspring was dissected and immunolabeled against nc82, HA and FLAG. nc82 was deposited to the DSHB by Buchner, E. (DSHB Hybridoma Product nc82). Labeling protocol and additional details are available at https://www.janelia.org/project-team/flylight/protocols.

### Phalloidin Immunohistochemistry and Confocal Imaging

In order to visualize which muscles were innervated by motoneurons with split GAL4 expression, we labeled the muscles with phalloidin, a mushroom toxin which binds to f-actin. Splits were crossed with pJFRC7-20xUAS-IVS-mCD8∷GFP in attP2 on cornmeal-based food and kept at 25°C. When the offspring were 3–4 days old, males were chilled on ice to anesthetize them, dunked in ethanol to remove the wax on the cuticle, and dipped in phosphate-buffered saline (PBS) twice to remove any traces of ethanol. The head, abdomen, wings and legs of each fly were removed using forceps and scissors. Then the fly thoraxes were fixed in 1% paraformaldehyde (Fisher Scientific) in PBS overnight at 4°C while nutating. The thoraxes were washed in 0.5% PBS with Triton-X (PBT) four times for 10–15 minutes each while nutating. Then the thoraxes were embedded in 7% SeaKem LE agarose (Fisher Scientific) which was hardened at 4°C for 60 minutes. The thoraxes were each cut in half along the midline by hand with a stiff single-edged razor blade (Personna). The preparations were blocked in PBS with 1% Triton-X, 0.5% DMSO, 3% normal goat serum, with a pinch of NaN_3_, and a pinch of escin. 1:200 bovine hyaluronidase type IV-S (Sigma Aldrich) was also added to break down connective tissue and thus allow better penetration of the antibody. The preparations were blocked for 60 minutes before being incubated in 1:100 phalloidin conjugated with Alexa Fluor 633 (Life Tech) along with 1:50 anti-nc82 in mouse (Univ of Iowa) and 1:1000 anti-GFP in rabbit diluted in the blocking solution. The preparations were left in the primary antibody and phalloidin solution for 5 days at 4°C.

After incubation in the primary antibody, preparations were washed four times for 10–15 minutes in 0.1% PBT. The preparations were blocked again in the same blocking solution but without hyaluronidase for 30 minutes before being incubated with 1:500 goat anti-mouse Cy3 and 1:500 goat anti-rabbit Alexa 488 for 2–3 days at 4°C. Preparations were washed overnight in 0.1% PBT, then fixed in 2% paraformaldehyde in PBS at 4°C, then washed four times 10–15 minutes. The preparations were cleared in a graded series of glycerol for 30 minutes each: 5% glycerol, 10% glycerol, 20% glycerol, 30% glycerol, 50% glycerol, 65% glycerol. Then the preparations were left in 80% glycerol overnight at 4°C. Preparations were dehydrated in a graded series of ethanol for 30 minutes each: 30% ethanol, 60% ethanol, 90% ethanol, then 100% ethanol three times for 15 minutes. The preparations were put into 50% methyl salicylate (Sigma Aldrich) for 30 minutes, then 100% methyl salicylate. To remove any remaining glycerol, the preparations were rinsed in ethanol and then returned to 100% methyl salicylate. The preparations were left in 100% methyl salicylate overnight at 4°C. The preparations were mounted on cover slips in methyl salicylate. Preparations were imaged with a 10x air objective using the Zeiss 880 NLO upright confocal microscope or the Zeiss 710 confocal microscope. Images were processed using Fiji and VVDViewer.

### Courtship song silencing experiments

Courtship behavior assays and analysis were performed as described in Arthur et al., 2013. Motoneuron splits were crossed with w+; UAS-TNTe or w;UAS-Kir2.1-GFP(III) or w+ DL; DL; pJFRC49-10XUAS-IVS-eGFPKir2.1 in attP2 (DL) (ID# 1117481) on semi-defined food (food described in Backhaus et al., 1984). As a control, “blank” split-GAL4 lines created from the same Gen1 GAL4 collection but with no central nervous system expression (SS01055 or SS01062) were also crossed with the same reporter lines on power food. Virgin male offspring were collected and socially isolated in small glass vials of power food. To entrain the circadian rhythm of the flies, for at least 48 hours before each experiment the males were kept in an incubator in which the lights turned on daily within two hours before the time of the experiment (i.e. if the incubator’s lights turned on at 9 am every day, the experiment would be done before 11 am). Each male was placed in his own courtship arena, along with a w1118 virgin female who had been collected the day before. An aspirator was used to insert the flies into the arena; they were not anesthetized. The arenas were within an acrylic box on air table to reduce outside noise and vibration. An acrylic platform held each chamber over a pressure gradient microphone. The temperature and humidity were recorded simultaneously using a SHT75 humidity sensor (Sensirion). Song was recorded for 30 minutes. Control and experimental flies were always recorded simultaneously.

### Flight activation experiments

Flight activation experiments were performed as in Suver et al., 2016. Motoneuron splits, as well as the SS01062 blank split negative control, were crossed with 20XUAS-CsChrimson-mVenus trafficked in attP18 on food with 1:250 retinal and kept in the dark. Female offspring were tested when 3–8 days old. Flies were cooled and tethered by gluing each fly to a wire with Loctite^®^ 3972^™^ UV-activated cement (Henkel). The tether was positioned on the anterior dorsal thorax on each fly. An arena of 470 nm blue LEDs was used to display closed-loop visual stimuli in the form of vertical stripe patterns, as described in in Kim et al., 2017. Fly behavior during these experiments was measured using three IR-sensitive cameras recording at 100 frames per second, as well as a Wingbeat Tachometer (IO Rodeo) for measuring wingbeat frequency.

A 617 nm red fiber-coupled LED (Thorlabs) suitable for optogenetic activation of the red-shifted channelrhodopsin, CsChrimson, was positioned below the fly, aimed at the ventral thorax and turned on in 0.1 s pulses with 6 s between each pulse. Each closed loop experiment comprised either 20 or 50 consecutive trials. In open loop experiments, the LED arena displayed closed loop visual stimuli for 20 trials, followed by open loop stimuli in random order, repeated 5 times, followed by closed loop stimuli for 10 trials. The open loop stimuli were horizontal expansion, clockwise rotation, counterclockwise rotation, and contraction of the stripes. The intensity of the red light was set to 1.9 milliW/mm^2^ for each experiment.

Kinefly, a machine vision system for quantifying flight behavior in real time (Suver et al., 2016), was used to extract the following wing kinematic parameters from the camera views and Wingbeat Tachometer: stroke amplitude, forward deviation angle, backward deviation angle, and wingbeat frequency.

### Behavioral Data Analysis

Analysis of behavioral data from tethered flight and courtship experiments was performed in MATLAB. For tethered flight experiments, noise in the single-trial wing kinematics was removed using a median-filter with a 15 ms window. To detect changes resulting from the optogenetic perturbation, the mean values of each kinematic variable in the 1 second window prior to an LED pulse were subtracted from individual traces. The filtered, mean-subtracted data was then averaged across trials for each fly/condition, as well as across the left and right wing, since the genetic lines used target the motor neurons bilaterally. The maximum change in the fly-averaged kinematics during the optogenetic stimulus period was then compared to control using the Wilcoxon rank sum test, as in Figure 10D–G. The 95% confidence intervals for per-fly mean wing kinematics, as in Figure 10C, were calculated by a 500-sample bootstrap procedure.

For courtship experiments, the FlySongSegmenter program (Arthur et al., 2013) was used to automatically segment raw sound recordings into periods of pulse song, sine song, or noise (non-singing). The per-fly fraction spent singing each song type (song index or pulse index) was compared to controls using a Wilcoxon rank sum test (Figure 11C, bottom and middle rows), while the total number of flies in a genotype that sung at all (percent time singing > 0.5%) was compared to control using Fisher’s exact test (Figure 11C, top row). Additionally, the identified song pulses were grouped into ‘fast’ or ‘slow’ mode pulses using the classifier described in (Clemens, et al., 2018). The per-fly fraction of pulses that were classified as ‘slow’ was compared between each genotype and its control using a Wilcoxon rank sum test. The per-genotype traces for pulse modes, as in Figure 11E, were calculated as the grand mean across flies, with 95% confidence intervals determined using a 500-sample bootstrap.

### Input/Output Identification

VNC interneurons were clustered by their pattern of inputs and outputs in previously defined neuropil regions (Namiki et al., 2018; Court et al., 2020). Post-synaptic dendrites (input sites) and pre-synaptic axonal terminals (output sites) were identified by their respective morphological differences using 63X magnification microscopy images. Post-synaptic dendrites have smooth endings, whereas axon terminals have a varicose shape (Namiki et al., 2018). For further confirmation of neurite type, split driver lines were crossed with pJFRC51-3xUAS-Syt∷smGFP-HA in su(Hw)attPa in order to visualize the localization of synaptotagmin. Using this identification strategy, each individual neuron from the driver line collection was scored for the presence or absence of inputs and outputs in each of the 28 identified VNC neuropils (14 neuropil regions, each with an ipsilateral and contralateral half; see Figure 1C and Figure 20). For a given cell and neuropil region combination, the possible annotation values were: “D” (“Dendrite,” signifying input), “A” (“Axon,” signifying output), “M” (“Mixed,” signifying mixed inputs and outputs), and “P” (“Partitioned,” signifying spatially segregated inputs and outputs in the same neuropil region). Moreover, we distinguished between major and minor innervation of a neuropil region using upper and lower case letters, respectively, i.e. “A” would represent major output, while “a” would represent minor output.

### Interneuron clustering

To identify connectivity motifs within the interneuron collection, we converted the categorical input/output annotations described above into 56-dimensional binary vectors for each cell, and performed a hierarchical clustering analysis on these resultant vectors. The conversion was accomplished by only taking into account major sites of innervation, and assigning these a value of “1” in the input/output vector; all other entries were set to “0.” To account for the difference between input and output, we separately generated two 28-dimensional binary vectors for each neuron (28 being the number of neuropil regions targeted for annotation), representing the major inputs and outputs respectfully, and concatenated them to arrive at the 56-dimensional vector which accounted for the full input/output profile of the cell, per our annotations. These combined input/output vectors were then hierarchically clustered using the Pearson correlation as a distance metric. Optimal cluster number was determined by maximizing the gap statistic (MATLAB, evalclusters.m), yielding 27 distinct VNC interneuron clusters (see Figure 20).

### Masked volume overlap

Potential anatomical connectivity was quantified using the 3D volume overlap of neuron pairs. For these calculations, individual neurons were segmented from 63X MCFO images aligned to a common template VNC (JRC2018_VNC_UNISEX). These individual neuron images were then binarized using a multi-level Otsu method thresholding.

Masked volume overlap was calculated using the input/output annotations described above, along with a set of segmented, binarized images of VNC neuropil volumes aligned to the common VNC template. For each binary neuron image, two masks were generated from the binarized VNC neuropil region images: a mask corresponding to input sites, and one corresponding to output sites. Input (output) site masks were obtained by taking the union of all neuropil region images in which the given neuron had input (output) sites, according to our annotations. For two neurons, indexed *j* and *k*, we calculated the masked overlap, $O_{jk}$, as:

$$O_{jk}=\left(M_{j}^{in}\circ N_{j}\right)\cdot \left(M_{k}^{out}\circ N_{k}\right)$$

Where *N*_*j*_ and *N*_*k*_ are the binarized images of neurons *j* and *k*; ∘ indicates the element-wise product between two arrays; · denotes the dot product; and $M_{j}^{in}$ and $M_{k}^{out}$ are the input and output masks for neurons *j* and *k*, respectively. Note that this calculation is not commutative, i.e. $O_{jk}\neq O_{kj}$, due to the inclusion of the input and output masks, which make the calculations directed. The quantity $O_{jk}$ gives the number of overlapping voxels that correspond to putative connections in which neuron *j* is postsynaptic and neuron *k* is presynaptic (vice-versa for $O_{kj}$).

## Supplementary Material

1

## Figures and Tables

**Table 1:** Interneuron naming convention See the main text for detailed definitions. Neuropil region names used for the first letter come from (Court et al., 2020). Definitions with asterisks are defined but not used in the current study.

Letter position	Significance	Possible entries
1st letter	primary arborizing neuropil	N (Neck tectulum, NTct)
		W (Wing tectulum, WTct)
		H (Haltere tectulum, HTct)
		C1 (prothoracic intermediate/lower Tectulum)
		C2 (mesothoracic intermediate/lower Tectulum)
		C3 (metathoracic intermediate/lower Tectulum)
		X (accessory mesothoracic neuropil, AMN)
		Y (accessory metathoracic neuropil)*
		L (Leg neuropils)*
		A (Abdominal neuromeres)*
2nd letter	Laterality	B (Bilateral)
		U (Unilateral)
3rd letter	Type of projection	L (Local segmental interneuron)
		I (Intersegmental interneuron)
		A (Ascending interneuron)*
		M (Motor neuron for non-wing muscles)*
ID number	3-digit code	From ventral to dorsal layers of arborizations

**Table 2:** List of neurons identified in the study The number of cell bodies, cell body locations and numbers, hemilineages of origin, areas of arborizations, existence of axon-like projections, the driver lines that label the cells and alternative names previously used in the literature are listed for all the neuron types. Loc.: location of the cell bodies: 1, 2, 3, A, associated with T1, T2, T3 or Abdominal ganglia; a, anterior; p, posterior, v, ventral; d, dorsal, m, medial; l, lateral;. No.: most plausible number of the cells of that type based on the comparison of the driver line labeling samples; actual numbers in the VNC may differ. For unpaired cells, the total number of cells is given in italics; for paired cells, the number of cells per side is given in plain text. Lineage: most plausible cell lineage based on the comparison of the neuroblast labeling: emb, embryonic; abd, abdominal. Areas of arborizations: first character represents arborization ipsilateral to the soma, second character represents arborization contralateral to the soma. A, axon terminals, D, dendritic arbors, M, intermixed input and output sites, P, input and output sites partitioned in separate areas within the same hemi-neuropil. Lower case letters, only small or sparse arborizations. Dash, no arborizations on that side of the VNC. N, neck neuropil; W, wing neuropil; X, accessory metathoracic neuropil (AMN); H, haltere neuropil; Y, accessory mesothoracic neuropil; iT1, iT2, iT3, intermediate tectulum in the T1, T2 and T3 neuromeres; lT1, lT2, lT3, lower tectulum in the T1, T2 and T3 neuromeres; L1, L2, L3; leg neuropils in the T1, T2 and T3 neuromeres; A, abdominal neuromeres; Ext; external projection towards outside of the VNC. Seg: +, existence of a section of elongated fiber(s) that connect different segregated masses of arborizations. Specific drivers: the lines that label only one or a few types of neurons that are clearly segregated. Other drivers: the lines that also label various other cells.

type	Somata Loc.	No.	hemi lineage	N	iT1	lT1	L1	X	W	iT2	lT2	L2	Y	H	iT3	L3	A	Ext	Seg	Specific lines	Other lines	other names in *Drosophila*	orthologous cells with visualized morphology
				86	42	5	9	28	174	131	34	30	0	113	10	25	1						
DLMNa/b	2adl	1	emb						DD									DLMa, DLMb		SS44039, SS44056	SS31541, SS31561, SS43980, SS44060	MN5 (1), MN5/6 (2), 45a/b (3)	DLMNa/b (Calliphora 4), MN5 (Manduca 5,6), DLMN1e (Bombyx 7)
DLMNc	2avl	1	emb						DD									DLMc		SS44039, SS44056	SS31541, SS31561, SS43980, SS44060	MN4 (1), 45c (3)	DLMNc (Calliphora 4), MN4 (Manduca 5,6), DLMN1d (Bombyx 7)
DLMNd	2avl	1	emb						DD									DLMd		SS44039, SS44056	SS31541, SS31561, SS43980, SS44060	MN3 (1), 45d (3)	DLMNd (Calliphora 4), MN3 (Manduca 5,6), DLMN1c (Bombyx 7)
DLMNe	2avl	1	emb						DD									DLMe		SS44039, SS44056	SS31541, SS31561, SS43980, SS44060	MN2 (1), 45e (3)	DLMNe (Calliphora 4), MN2 (Manduca 5,6), DLMN1b (Bombyx 7)
DLMNf	2avl	1	emb						DD									DLMf		SS44039, SS44056	SS31541, SS31561, SS43980, SS44060	MN1 (1), 45f (3)	DLMNf (Calliphora 4), MN1 (Manduca 5,6), DLMN1a (Bombyx 7)
DVMN1a	2avl	1	emb						DD	d–								DVM1a		SS41068, SS49797	SS31950, SS40989, SS43980, SS44060	DVMNIa (8)	DVMN1a (Calliphora 4)
DVMN1b	2avl	1	emb						DD	d–								DVM1b		SS41068, SS49797	SS31950, SS40989, SS43980, SS44060	DVMNIb (8)	DVMN1b (Calliphora 4)
DVMN1c	2avl	1	emb						DD									DVM1c		SS41068, SS49797	SS31950, SS40989, SS43980, SS44060	DVMNIc (8)	DVMN1c (Calliphora 4)
DVMN2a	2pvl	1	emb						DD									DVM2a		SS41068, SS49797	SS31950, SS40989, SS43980, SS44060	DVMNIIa (8)	DVMN2a (Calliphora 4)
DVMN2b	2pvl	1	emb						DD	d–								DVM2b		SS41068, SS49797	SS31950, SS40989, SS43980, SS44060	DVMNIIb (8)	DVMN2b (Calliphora 4)
DVMN3a	2avl	1	emb						DD									DVM3a		SS41068, SS49797	SS31950, SS40989, SS43980, SS44060	DVMNIIIa (8)	DVMN3a (Calliphora 4)
DVMN3b	2avl	1	emb						DD	d–								DVM3b		SS41068, SS49797	SS31950, SS40989, SS43980, SS44060	DVMNIIIb (8)	DVMN3b (Calliphora 4)

tpN MN	2avl	1	emb	Dd					DD	D–				d–				tp1, tp2		SS51528			
tp1 MN	2avl	1	emb	Dd					DD	d–				dd				tp1			SS41052, SS36076	dtp MN (9)	
tp2 MN	2avl	1	emb	Dd					DD	D–				d–				tp2		SS47120	SS42464	vtp MN (9)	
ps1 MN	2pvl	1	emb	Dd					DD	d–	d–			d–				ps1		SS47152			
ps2 MN	2avl	1	emb															ps2					
tt MN	2avl	1	emb							a–	a–	D–						tt	+	SS47125	SS40765, SS40772, SS41027, SS45772		tt MN (Calliphora 10)
b1 MN	2avl	1	emb						Dd	d–				D–				b1	+		SS40980		b1 MN (Calliphora 11)
b2 MN	2avl	1	emb	D–					D–	D–	D–			D–				b2		SS45772	SS40772, SS41027, SS47160		
b3 MN	2avl	1	emb	D–					D–	D–				d–				b3	+	SS45779, SS52405	SS47160, SS48311, SS49806		
i1 MN	2avl	1	emb	D–					D–	D–	D–			d–				i1			SS40772, SS41027, SS41039		
i2 MN	2avl	1	emb	D–					Dd	Dd	DD							i2		SS37246, SS45782	SS34778, SS34781, SS45778, SS53438		
iii1 MN	2pvl	1	emb						D–	Dd	DD							iii1	+		SS40980		
iii3 MN	2pvl	1	emb						D–					d–				iii3	+	SS45779	SS40980		
iii4 MN	2pvl	1	emb	d–					D–	D–				D–				iii4		SS45779	SS41027, SS47160		
hg1 MN	2avl	1	emb	D–					D–	D–				D–				hg1		SS32023, SS47161	SS40864, SS45779, SS49809	vMS2 (12)	
hg2 MN	2avl	1	emb	dD					DD	DD				dd				hg2	+	SS37253, SS52395	SS37252, SS52404		
hg3 MN	2avl	1	emb	DD				D–	DD	dd				DD				hg3	+	SS49039, SS52405	SS37252, SS40864, SS47160, SS52404		
hg4 MN	2avl	1	emb															hg4					

hDVMN	3pvl	1	emb						–d					DM				hDVM	+	SS51523, SS51524	SS25532, SS37253, SS40989, SS53378		
hiii2 MN	3adm	1	emb															hiii2					
hiii3 MN	3avl	1	emb															hiii3			SS36076		
hb1/2a MN ?	3avl	1	emb	M–	m–				M–	D–				M–				+		SS47195	SS40851		
hb1/2b MN ?	3avl	1	emb	M–	m–				M–	D–				M–		d–		+	+	SS47195	SS40851		
hi1 MN	3avl	1	emb											DD	dd			hi1		SS25532, SS37231	SS37253, SS37295, SS41075, SS47195		
hi2 MN	3avl	1	emb											MM		d–		hi2		SS25532, SS37231	SS36076, SS40989		

T2VUM1	2avm	*1*	0B t2	dd	dd				MM	dd								+		SS40868	SS40867, SS45766		
T2VUM2	2avm	*1*	0B t2	dd	dd				MM	DD								+		SS42385, SS46645	SS40867, SS45766		mesVUM-TT? (Calliphora 13)
T2VUM3	2avm	*1*	0B t2	dd	dd				MM	dd								+		SS40868, SS42385	SS40867, SS45766, SS37253		
T2VUM4	2pvm	*1*	0B t2	dd	dd				MM	dd								+		SS40868, SS42385	SS40867		mesVUM-MJ? (Calliphora 13)
T2VPM1	2avm	2	2A t2	aa	–d	–D		–D	MM	mm	MM			–a	–A				+	SS48268			
A1VUM1	Aavm	*3*	abd											mm			DD	+	+	SS51508			

NUL001	1avm	5	2A t1	P–																	SS44314		

XBL001	2avm	1	emb					MM	AA	–a	aa	d–							+	SS43546			
C2BL001	2vma	1	0A t2					mm	mm	MM	MM									SS36094			
C2BL002	2vma	1	0A t2					mm	mm	MM	MM									SS36094			

WUL001	2avm	5	2A t2						–M	–m											SS44314		
WUL002	2avm	1	2A t2	a–			a–	d–	M–	m–										SS31472	SS44314		
WUL003	2adl	1	11B t2						M–											SS33409, SS49042			
WUL004	2adl	1	11B t2						M–	m–										SS33409, SS49041			
WUL005	2pdl	1	19A t2						M–											SS33409, SS49042	SS49800		
WUL006	2pdm	1	19A t2						M–											SS25511, SS33409			
WUL007	2adl	1	17A t2					D–	M–	a–		m–		a–							SS42498	dMS2 (12)	
WUL008	2adl	1	17A t2	m–				D–	M–	a–		m–		a–						SS42442	SS42439, SS42498	dMS2 (12)	
WUL009	2adl	1	17A t2	m–			m–	D–	M–	a–		m–		a–						SS42442	SS42439	dMS2 (12)	
WUL010	2adl	1	17A t2				m–	D–	M–	a–		m–		a–							SS42498	dMS2 (12)	
WUL011	2adl	1	17A t2				d–	D–	M–	a–		m–		a–							SS42438	dMS2 (12)	
WUL012	2adl	1	17A t2		m–				M–	M–	m–	m–		a–							SS42438, SS42439, SS47161, SS49766	dMS2 (12)	
WUL013	2pdl	1	3B t2	m–				m–	M–	P–	d–									SS48215			
WUL014	2pdm	1	3B t2	m–	m–			m–	M–	P–	d–									SS48215			
WUL015	1pvl	1	3B t1						M–	m–											SS48268		
WUL016	1pvl	1	3B t1						M–												SS47192, SS48268		
WUL017	2pdm	1	19B t2	m–	m–			m–	M–	P–	d–									SS48215			
WUL018	2pvm	2	6B t2	–a				–m	–M					–a						SS53421			

WBL001	2adl	1	18B t2						Aa	a–	p–	PA							+	SS45607, SS45830	SS40782		
WBL002	2adl	1	18B t2						A–	a–	p–	PA							+	SS45830, SS45843			
WBL003	2adl	1	18B t2						A–	a–	p–	PA							+	SS45843, SS42465			
WBL004	2adl	1	18B t2		d–				A–	a–	p–	PA							+	SS45830			
WBL005	2adl	1	18B t2						–A	–A	Da	DA							+	SS42465, SS45843	SS45607, SS45830		
WBL006	2adl	1	18B t2						AA	AA	d–	D–							+	SS25482, SS31309	SS31263		
WBL007	2adl	1	18B t2						aA	aa	d–	DA							+	SS31309, SS45830	SS31263, SS45607		
WBL008	2adl	1	18B t2						AA	aa		DA							+	SS31263			
WBL009	2pvm	3	emb					–M	MM	–M	aa								+	SS46725			
WBL010	2pvm	1	12A t2						M–	MA									+	SS48272			
WBL011	2pvm	1	12A t2						MM	MM										SS48272, SS42465			
WBL012	2pvm	1	12A t2						MM	MM	d–									SS48272, SS42465			
WBL013	2avm	10	6B t2						AA	MM	mm									SS47200, SS49799	SS54445		
WBL014	2adl	1	18B t1					d–	MM	m–										SS40783	SS49766		
WBL015	2adl	1	17A t2					d–	MM	M–	d–									SS42498	SS42438, SS42439	dMS2 (12)	
WBL016	2adl	1	17A t2					d–	MM	DD										SS40783, SS46675		dMS2 (12)	
WBL017	2adl	1	17A t2						PD	PD										SS40783, SS42442	SS42438	dMS2 (12)	
WBL018	2adl	1	17A t2						PD	PD										SS46675		dMS2 (12)	
WBL019	2adl	1	17A t2						PP	PD										SS46675	SS42438	dMS2 (12)	
WBL020	2adl	1	17A t2						PD	DD										SS40783		dMS2 (12)	
WBL021	2adl	1	17A t2						PP	PP										SS40783	SS42438	dMS2 (12)	
WBL022	2avm	4	6B t2					–a	aM	–a										SS42447			
WBL023	1pvl	4	6B t1	D–					Ma	d–										SS49779			
WBL024	1pvl	3	3B t1					d–	MM	MM										SS51830			
WBL025	1pvl	1	3B t1						MA										+		SS37306		
WBL026	1pvl	1	3B t1	dd					MM										+	SS52389			
WBL027	1pvl	1	3B t1	dd					MM										+	SS52389			
WBL028	1pvl	1	3B t1	dd					MA										+	SS52389			
WBL029	1pvl	1	3B t1	dd					MA										+	SS52389			
WBL030	1pvl	1	3B t1	dd					MA										+	SS52389			
WBL031	1pdl	1	3B t1	dd	–D				MM										+	SS31259, SS44002	SS44003, SS47219, SS47222, SS48268		
WBL032	1pvl	2	3B t1	DD	aa				MM										+	SS44002, SS44003			
WBL033	1pvl	1	3B t1						DA										+	SS44003	SS48268		
WBL034	2pvm	1	12A t2	aa					MA										+		SS47192		
WBL035	2pdm	1	19B t2						MM										+		SS37233, SS49041		
WBL036	2pdm	1	19B t2	a–					MM	DD									+	SS20796	SS40778, SS37233		
WBL037	2pdl	1	19B t2						MM	dd									+	SS20796	SS37233		
WBL038	2pdl	1	19B t2	mm	d–				MM										+	SS49041			
WBL039	2pdm	1	19B t2	aa					MM										+		SS37233, SS40778		

HUL001	3avm	1	2A t3											P–	D–	M–				SS54506	SS41041		
HUL002	3avm	2	2A t3							A–				P–	P–	p–				SS25502	SS44314		
HUL003	3avm	1	2A t3							a–				M–						SS54506			
HUL004	3avm	1	2A t3							a–				M–						SS54506			
HUL005	3avm	1	2A t3							m–				M–						SS25502, SS54506	SS31472		
HUL006	3avm	1	2A t3											M–						SS25502			
HUL007	3avm	1	2A t3							m–				M–						SS54506			
HUL008	3avm	1	2A t3							a–				M–						SS54506			
HUL009	3avm	1	2A t3							a–				M–						SS54506			
HUL010	3pdl	1	3B t3											M–						SS49125			
HUL011	3avm	?	19B t3							a–				M–						SS25500, SS31274			
HUL012	3pvl	3	19B t3							a–				M–						SS25500, SS25478	SS31274		

HBL001	3pvm	4	6B t3											MM					+	SS45611			

NUI001	1pvm	5	6B t1	–D	–a				–m	–A				–A					+	SS54480, SS54495			
NUI002	1pdl	5	11B t1	D–	m–				P–	A–				A–					+	SS48619			

NBI001	2avm	?	6B t2	M–	Mm			d–	M–	MA	dd			M–					+	SS60603			
NBI002	2avm	2	7B t2	Da					DA	d–				–A					+	SS31899			
NBI003	1pvm	1	6B t1	DA					D–	–a				–A					+	SS30330, SS32400	SS54480		
NBI004	1pvm	1	6A t1	DA	–a				–m	–a				–A					+	SS30330, SS54480	SS32400, SS54474		
NBI005	1pvm	1	6A t1	DA	–a				–m	–a				–A					+	SS49807			
NBI006	1pvm	1	6A t1	DA										–A					+	SS54495	SS54474		
NBI007	1pvl	1	6A t1	DA					–m	–a				–A					+	SS54480	SS31309, SS54474, SS54495		
NBI008	1pvm	1	6B t1	DD	AA				dD	–A				–A					+		SS25553, SS54495		
NBI009	1pvm	1	6B t1	DD	–A				dp	–A				–A					+		SS25553, SS54495		
NBI010	1pvm	1	6B t1	DA					–A					–A					+	SS42079			
NBI011	1pvm	1	6B t1	D–					–A					–A					+	SS42079			
NBI012	1pvl	1	6B t1	D–					–A					–A					+	SS42079			

PSI	2adl	1	18B t2			D–	d–	D–	–d	dd	DD	d–						DLMn		SS49776, SS49809	SS49777, SS49810, SS49812		PSI (*Z210* 10)
XBI001	1pvm	2	5B t1		AA	AA	mm	dd			AA	mm							+		SS33489, SS60603		
XBI002	2avm	?	0A t2			AA	mm	PP		AA	PP	aa								SS42475, SS47200	SS48311		

C2BI001	2avm	1	18B t2	–a	–A				a–	AA	aa	D–							+	SS52392	SS49784		
C2BI002	2avm	1	18B t2	–a	–A				a–	MA		D–							+		SS49784		
C2BI003	2avm	1	7Bα t2		–a				mm	ma	m–	M–							+	SS52392	SS41039, SS49784		
C2BI004	2avm	2	7B t2	DA					DA	DA				DA					+	SS28361			
C2BI005	2avm	1	7B t1	d–	Da	–a			p–	MA	–A	M–								SS46735	SS42475, SS42499, SS60603		
C2BI006	2avm	1	0A t2	–a	–A				aa	aA	dd	D–		–a		–a			+	SS42464, SS52392	SS31290		
C2BI007	2avm	1	18B t2	–a	–a				–A	–A		D–		–a		–a			+	SS42464			
C2BI008	2avm	1	18B t2	–a	AA				Aa	AA		D–		–a		–a			+	SS31289, SS31290			
C2BI009	2avm	1	18B t2	–a	–A				AA	AA		D–		–a		–a			+	SS31289, SS42464	SS31290, SS46735, SS49784		
C2BI010	2avm	1	18B t2	A–	AA				a–	AA		D–		–a		–a			+	SS31290, SS52392			

WUI001	2pvl	1	3B t2						A–					D–	a–						SS49800, SS49778		
WUI002	2pvl	1	3B t2						A–					D–						SS49778, SS49807	SS49800		
WUI003	2pvl	3	11B t2						M–					M–						SS49807	SS29871		
WUI004	3pvm	2	abd					–d	–M					–M		–m			+		SS29871		
WUI005	1pvl	3	3B t1	m–	D–				M–	D–				a–	D–					SS49779			
WUI006	2avm	1	6B t2	–A					–M	–d				–A						SS40456, SS53421	SS42447		
WUI007	2pvm	1	6B t2						–M	–d				–A						SS53421			
WUI008	2avm	1	6B t2	–A					–M	–d				–A						SS40456, SS53421	SS42447		
WUI009	2avm	1	6B t2	–A					–M	–d				–A		–a				SS40456, SS53421	SS42447		
WUI010	2avm	10	12A t2					–d	–M	–M				–A					+	SS49799			
WUI011	3avm	1	3B t2						A–					D–							SS49800		
WUI012	2pvm	1	3B t2						A–					D–							SS49800		

WBI001	2pdm	1	19B t2	m–	m–				DA	Da				mA		–a			+	SS25521, SS37984	SS33437, SS40764		
WBI002	2pdm	1	19B t2	a–					MA	Ma				AA		–a			+	SS25521, SS33437	SS40764, SS40782		
WBI003	2pvm	1	6A t2						MA	mm									+		SS40980, SS45385		
WBI004	2avm	1	6A t2	–A					MA	m–				d–					+		SS45385		
WBI005	2avm	1	6A t2	–a	–A				Dm	DD									+	SS42446	SS48204		
WBI006	2avm	1	6A t2	–a	–a				Da	DD									+	SS42446	SS48204		
WBI007	2pvm	1	6A t2	–A	–a				DA	DA				A–					+	SS42446	SS45385, SS48204		
WBI008	2pvm	1	6A t2	–A	–a				MA	DA				d–					+		SS48204		
WBI009	2avm	1	6A t2	–a	–a				DA	DA				–A					+	SS42446	SS48311		
WBI010	2pvl	1	6A t2	–A	–A				DA	MA				d–					+		SS45385, SS48204		
WBI011	2pvl	1	6A t2	–A					MP	DA				DM					+		SS25553, SS45385, SS48204		
WBI012	2avm	1	6A t2						DA					–A					+		SS40969, SS42499		
WBI013	2adl	1	17A t2				a–	d–	MM	Dd											SS42438	dMS2 (12)	
WBI014	2avm	?	0A t2		DD				MM	DD										SS46295			
WBI015	1pvl	3	6B t1	d–	A–				Mm	Mm				a–						SS49779			
WBI016	1pvl	2	3B t1	mm	dd		dd		Pp	d–				aa						SS49779, SS51830			
WBI017	1pdl	1	3B t1	aa					MM	dd	dd			AA					+	SS49802	SS40969		
WBI018	2adl	2	3B t2						D–					AA					+	SS44005, SS44051	SS31259, SS44003, SS47219, SS47222		
WBI019	2pdm	2	3B t2						DD					AA					+	SS31259, SS44051	SS40969, SS44002, SS47222, SS48221		
WBI020	3adl	?	19B t3						–A					D–					+		SS40969		
WBI021	Aadl	3	abd						Mm					M–					+	SS29600, SS29871			

HUI001	3adm	1	17A t3						A–	M–				D–		d–			+	SS51531			
HUI002	3adm	1	17A t3						Aa	M–				D–		d–			+	SS48709	SS46735		
HUI003	3adm	2	17A t3						A–	P–				D–		d–			+	SS51531	SS46260, SS46735		
HUI004	3adm	1	17A t3						A–	M–				D–		d–			+	SS46260, SS48709			
HUI005	3adm	1	17A t3						A–	m–				P–		d–			+	SS46735, SS48709	SS46260		

HBI001	3adl	2	18B t3	–a					AA	MM				DD	–d	DD			+	SS49777			
HBI002	3adl	1	emb						–a	–A		–A		MA	–m	MA			+	SS49777			
HBI003	3adm	1	emb						–m	–a		–A		MA	–d	MA			+		SS42499, SS43979		
HBI004	3avl	?	8B t3	–A					–a					Ma		d–			+	SS47214		n-cHIN (14)	n-cHIN (Calliphora 15)
HBI005	3avl	?	8B t3	–A					–a					MA		d–			+	SS47215		n-cHIN (14)	n-cHIN (Calliphora 15)
HBI006	3avl	?	8B t3	–A										D–					+	SS44002, SS47214	SS47215	n-cHIN (14)	n-cHIN (Calliphora 15)
HBI007	3avm	?	8B t3	–A										D–					+	SS47214, SS47215	SS47204	n-cHIN (14)	n-cHIN (Calliphora 15)
HBI008	3avl	1	8B t3						–A					D–					+	SS49853		w-cHIN (14)	w-cHIN (Calliphora 16)
HBI009	3avl	1	8B t3						–A					D–					+	SS47215		w-cHIN (14)	w-cHIN (Calliphora 16)
HBI010	3avl	1	8B t3						–A					MA					+	SS49853		w-cHIN (14)	w-cHIN (Calliphora 16)
HBI011	3avm	2	8B t3						–A					M–					+		SS29535	w-cHIN (14)	w-cHIN (Calliphora 16)
HBI012	3avm	2	8B t3						–A					D–					+		SS29535	w-cHIN (14)	w-cHIN (Calliphora 16)
HBI013	3pvm	3	6A t3	–A					dA					MA					+	SS44276			
HBI014	3adl	3	19B t3	–A					–M	Da				Da					+	SS31246			
HBI015	3adm	2	19B t3						–A					M–	d–	da			+	SS30816, SS32377	SS37274, SS42050, SS42493		
HBI016	3adm	4	abd						–A					DA		da			+	SS42050			
HBI017	3pvm	1	3B t2						–A					D–					+	SS49806		w-cHIN (14)	
HBI018	3pvm	1	3B t2						–A					D–					+	SS29602		w-cHIN (14)	
HBI019	3pdl	1	3B t2						DA					D–					+	SS29602, SS47215	SS48221	w-cHIN (14)	
HBI020	3pdl	1	3B t2						aA	d–				D–					+	SS29602	SS44054	w-cHIN (14)	

**Table 3:** List of driver lines in the study For each driver line, the cell categories, cell types, VNC expression quality rating and brain expression quality rating are given. Cell categories provide an overview of which cells are present: Bi, bilateral; Uni, unilateral; MN, motoneuron; LN, local interneuron; IN, intersegmental interneuron; VUM, ventral unpaired median neuron; VPM, ventral paired median neuron. Cell types list the names of cells identified in each driver line. VNC and Brain ratings award a grade of A to driver lines with no off-target expression, A/B for driver lines with off-target expression in one or two cells, B for up to five off-target cell types, C for more than five off-target cell types.

Driver line	Cell categories	Cell types	VNC rating	Brain rating
SS20796	Bi-LN	WBL036, WBL037	B	A/B
SS25478	Uni-LN	HUL012	B	B
SS25482	Bi-LN	WBL006	A	B
SS25500	Uni-LN	HUL011, HUL012	A	B
SS25502	Uni-LN	HUL002, HUL005, HUL006	A	C
SS25511	Uni-LN	WUL006	B	B
SS25521	Bi-IN	WBI001, WBI002	A	B
SS25532	haltere MN	hDVMN, hi1 MN, hi2 MN	A	A
SS25553	Bi-IN	NBI008, NBI009, WBI011	C	B
SS28361	Bi-IN	C2BI004	A	A
SS29535	Bi-IN	HBI011, HBI012	C	C
SS29600	Bi-IN	WBI021	A	B
SS29602	Bi-IN	HBI018, HBI019, HBI020	A	A
SS29871	Uni-IN, Bi-IN	WUI004, WBI021	C	B
SS30330	Bi-IN	NBI003, NBI004	A	B
SS30816	Bi-IN	HBI015	A	A
SS31246	Bi-IN	HBI014	A	B
SS31259	Bi-LN, Bi-IN	WBL031, WBI018, WBI019	B	A
SS31263	Bi-LN	WBL006, WBL007, WBL008	B	A
SS31274	Uni-LN	HUL011, HUL012	B	B
SS31289	Bi-IN	C2BI008, C2BI009	A	B
SS31290	Bi-IN	C2BI006, C2BI008, C2BI009, C2BI010	A	B
SS31309	Bi-LN, Bi-IN	WBL006, WBL007, NBI007	B	B
SS31472	Uni-LN	WUL002, HUL005	B	B
SS31541	power MN	DLMNs	B	B
SS31543	power MN, haltere MN	DLMNs, DVMNs, hDVMN	C	A/B
SS31561	power MN	DLMNs	C	A
SS31899	Bi-IN	NBI002	B	B
SS31950	power MN	DLMNs, DVMNs	B	B
SS32023	wing control MN	hg1 MN	B	A/B
SS32377	Bi-IN	HBI015	A	B
SS32400	Bi-IN	NBI003, NBI004	B	B
SS33409	Uni-LN	WUL003, WUL004, WUL005, WUL006	A	A
SS33437	Bi-IN	WBI001, WBI002	B	B
SS33489	Bi-IN	XBI001	C	B
SS34778	wing control MN	i2 MN	C	B
SS34781	wing control MN	i2 MN	B	B
SS34789	leg MN	leg MN	C	B
SS36076	wing control MN, haltere MN	tp1 MN, hi2 MN	C	C
SS36094	Bi-LN	C2BL001, C2BL002	B	C
SS37231	haltere MN	hi1 MN, hi2 MN	B	B
SS37233	VUM, Bi-LN	WBL035, WBL036, WBL039	C	B
SS37246	wing control MN	i2 MN	B	B
SS37252	wing control MN	hg2 MN, hg3 MN	A	B
SS37253	wing control MN, haltere MN, VUM	hg2 MN, hg3 MN, hDVMN, hi1 MN, T2VUM3	B	B
SS37262	Uni-LN	leg local interneuron	B	B
SS37274	Bi-IN	HBI015	C	B
SS37294	power MN, wing control MN,	DLMNs, DVMNs, tt MN	C	B
SS37295	power MN, haltere MN	DLMNs, DVMNs, hi1 MN	C	A
SS37296	Bi-AN	leg ascending neuron	C	B
SS37306	Bi-LN	WBL025	C	A
SS37984	Bi-LN	WBI001	A	B
SS40449	Uni-LN	leg local interneuron	A/B	A
SS40456	Uni-IN	WUI006. WUI008, WUI009	A	A
SS40764	Bi-IN	WBI001, WBI002	B	A
SS40765	power MN, wing control MN	DLMNs, DVMNs, tt MN, i2 MN	C	C
SS40772	power MN, wing control MN	DLMNs, tt MN, b2 MN, i1 MN	C	B
SS40778	Bi-LN	WBL036, WBL039	C	A
SS40782	Bi-LN, Bi-IN	WBL001, WBI002	C	B
SS40783	Bi-LN	WBL014, WBL016, WBL017, WBL020, WBL021	A	A
SS40851	haltere MN	hb1 MN, hb2 MN	B	A
SS40864	wing control MN	hg1 MN, hg3 MN	C	B
SS40865	Bi-SN	leg sensory	C	B
SS40867	VUM	T2VUM1, T2VUM2, T2VUM3, T2VUM4	B	A
SS40868	VUM	T2VUM1, T2VUM3	B	A
SS40969	haltere MN, Bi-IN	hDVMN, WBI012, WBI017, WBI019, WBI020	C	A
SS40980	wing control MN	b1 MN, iii1 MN, iii3 MN	C	A
SS40989	power MN, wing control MN, haltere MN	DLMNs, DVMNs, tt MN, hDVMN, hi1 MN, hi2 MN	C	A
SS41027	wing control MN	tt MN, b2 MN, i1 MN, iii4 MN	C	A
SS41029	abdominal MN	adominal MN	C	A
SS41034	Uni-SN	leg sensory	B	B
SS41039	wing control MN, Bi-IN	i1 MN, C2BI003	C	C
SS41041	Uni-LN	HUL001	C	B
SS41052	wing control MN	tp1 MN	C	C
SS41068	power MN	DLMNs, DVMNs	B	A
SS41075	power MN, haltere MN	DVMNs, hi1 MN	C	A
SS42050	Bi-IN	HBI015, HBI016	A	B
SS42079	Bi-IN	NBI010, NBI011, NBI012	A	A
SS42385	VUM	T2VUM2, T2VUM3, T2VUM4	B	A
SS42438	Uni-LN, Bi-LN, Bi-IN	WUL011, WUL012, WBL015, WBL017, WBL019, WBL021, WBI013	B	A
SS42439	Uni-LN, Bi-LN	WUL008, WUL009, WBL015	B	A
SS42442	Uni-LN, Bi-LN	WUL008, WUL009, WBL017	B	B
SS42446	Bi-IN	WBI005, WBI006, WBI007, WBI009	B	A
SS42447	Bi-LN, Uni-IN	WBL022, WUI006, WUI008, WUI009	B	A
SS42464	wing control MN, Bi-IN	tp2 MN, C2BI006, C2BI007, C2BI009	B	A
SS42465	Bi-LN	WBL001, WBL003, WBL005, WBL011, WBL012	B	A
SS42475	Bi-IN	XBI002, C2BI005	B	A
SS42493	Bi-IN	HBI015	B	B
SS42498	Uni-LN	WUL007, WUL008, WUL010, WUL012, WBL015	B	B
SS42499	Bi-IN	C2BI005, WBI012, HBI003	C	A
SS43546	Bi-LN	XBL001	B	A
SS43972	Bi-LN	leg bilateral interneuron	B	A
SS43979	Bi-IN	HBI003	B	A
SS43980	power MN	DLMNs, DVMNs	B	A
SS43995	wing control MN, Uni- LN	b2 MN, leg interneuron	B	B
SS44002	Bi-LN, Bi-IN	WBL031, WBL032, HBI006	B	A
SS44003	Bi-LN, Bi-IN	WBL031, WBL032, WBL033, WBI018, WBI019	B	A
SS44005	Bi-IN	WBI018	B	A
SS44009	Uni-AN	leg ascending neuron	C	B
SS44028	Bi-AN	leg ascending neuron	C	B
SS44034	Bi-IN	leg intersegmental neuron	A	A
SS44039	power MN	DLMNs	A	A
SS44046	Uni-LN	leg local interneuron	A	A
SS44051	Bi-IN	WBI018, WBI019	B	A
SS44054	Bi-IN	HBI020	C	A
SS44056	power MN	DLMNs	A	A
SS44057	Uni-LN	leg local interneuron	C	A
SS44060	power MN, haltere MN	DLMNs, DVMNs	B	A
SS44081	Uni-SN	leg sensory	A	A
SS44276	Bi-IN	HBI013	B	B
SS44314	Uni-LN	NUL001, WUL001, WUL002, HUL002	C	B
SS45385	Bi-IN	WBI003, WBI004, WBI007, WBI010, WBI011	C	B
SS45607	Bi-LN	WBL001, WBL005, WBL007	B	A
SS45611	Bi-LN	HBL001	A	B
SS45736	Uni-IN	HUI004, leg interneuron	C	A
SS45766	VUM	T2VUM1, T2VUM2, T2VUM3	B	A
SS45772	wing control MN	tt MN, b2 MN, i2 MN	B	A
SS45778	wing control MN	i2 MN	C	A
SS45779	wing control MN	b3 MN, iii3 MN, hg1 MN	B	A
SS45782	wing control MN	i2 MN	B	B
SS45830	Bi-LN	WBL001, WBL002, WBL004, WBL005, WBL007	A	A
SS45843	Bi-LN	WBL002, WBL003, WBL005	A	A
SS46260	Uni-IN	HUI003, HUI004, HUI005	A	B
SS46295	Bi-IN	WBI014	A	A
SS46645	VUM	T2VUM2	A	B
SS46675	Bi-LN	WBL016, WBL018, WBL019	A	A
SS46725	Bi-LN	WBL009	B	B
SS46735	Uni-IN, Bi-IN	C2BI005, C2BI009, HUI002, HUI003, HUI005	B	A
SS47120	wing control MN	tp2 MN	A/B	B
SS47125	wing control MN	tt MN	A	A
SS47152	wing control MN	ps1 MN	A	B
SS47160	wing control MN	b2 MN, b3 MN, iii4 MN, hg1 MN, hg3 MN	B	A
SS47161	wing control MN, Uni- LN	hg1 MN, WUL012	B	A
SS47192	Uni-LN, Bi-LN	WUL016, WBL034	B	A/B
SS47195	haltere MN	hi1 MN, hb1 MN, hb2 MN	B	B
SS47200	Bi-LN	WBL013, XBI002	A	B
SS47204	Uni-SN, Bi-IN	prosternal sensory, HBI007	B	B
SS47214	Bi-IN	HBI004, HBI006, HBI007	B	A
SS47215	Bi-IN	HBI005, HBI006, HBI007, HBI009, HBI019	B	B
SS47219	Bi-LN, Bi-IN	WBL031, WBI018, WBI019	C	A
SS47222	Bi-LN, Bi-IN	WBL031, WBI018, WBI019	B	A
SS48204	Bi-IN	WBI005, WBI006, WBI007, WBI008, WBI010, WBI011	C	A
SS48215	Uni-LN	WUL013, WUL014, WUL017	A	A
SS48221	Bi-IN	HBI019	C	B
SS48240	abdominal MN	abdominal MN	B	A
SS48247	abdominal MN	abdominal MN	B	A
SS48268	VPM, Uni-LN, Bi-LN	T2VPM1, WUL015, WUL016, WBL031, WBL033	C	A
SS48272	Bi-LN	WBL010, WBL011, WBL012	C	A
SS48287	Uni-SN	prosternal sensory	C	A
SS48305	Bi-IN	leg bilateral interneuron	B	B
SS48311	wing control MN, Bi-IN	b3 MN, hg1 MN, XBI002, WBI009	B	B
SS48619	Uni-IN	NUI002	B	B
SS48709	Uni-IN	HUI002, HUI004, HUI005	B	A
SS49039	wing control MN	hg3 MN	B	A
SS49041	Bi-LN	WUL004, WUL005, WBL035, WBL038	A	A
SS49042	Uni-LN	WUL003, WUL005	B	A
SS49125	Uni-LN	HUL010	A	A
SS49766	Bi-LN	WUL012, WBL014	C	A
SS49776	Bi-IN	PSI	A	A
SS49777	Bi-IN	PSI, HBI001, HBI002	A/B	B
SS49778	Uni-IN	WUI001, WUI002	B	A
SS49779	Bi-LN, Uni-IN, Bi-IN	WBL023, WUI005, WBI015, WBI016	B	A
SS49784	Bi-LN, Bi-IN	C2BI001, C2BI002, C2BI003, C2BI009	C	A
SS49853	Bi-IN	HBI008, HBI010	A/B	B
SS49797	power MN	DLMNs, DVMNs	B	A
SS49799	Bi-LN, Uni-IN	WBL013, WUI010	B	B
SS49800	Uni-IN	WUL005, WUI001, WUI002, WUI011, WUI012	B	A
SS49802	Bi-IN	WBI017, WBI019	A	A
SS49806	wing control MN, Bi-IN	b3 MN, hg1 MN, HBI017	A/B	B
SS49807	Bi-IN, Uni-IN	NBI005, WUI002, WUI003	B	A/B
SS49809	Bi-IN	PSI	A	A/B
SS49810	Bi-IN	PSI	B	B
SS49812	Bi-IN	PSI	A/B	A
SS49861	Uni-LN	leg local interneuron	B	A
SS51508	VUM	A1VUM1	B	A
SS51523	haltere MN	hDVMN	A/B	B
SS51524	haltere MN	hDVMN	A	A
SS51528	wing control MN	tpN MN	A	B
SS51531	Uni-IN	HUI001, HUI003	B	B
SS51830	Bi-LN, Bi-IN	WBL024, WBI016	B	A
SS52389	Bi-LN	WBL026, WBL027, WBL028, WBL029, WBL030	B	B
SS52392	Bi-IN	C2BI001, C2BI003, C2BI006, C2BI007, C2BI010	A	B
SS52395	wing control MN	hg2 MN	A	A/B
SS52404	wing control MN	hg1 MN	A/B	A
SS52405	wing control MN	b3 MN, i2 MN, hg3 MN	A/B	A
SS53378	haltere MN	hDVMN	A	A
SS53421	Uni-LN, Uni-IN	WUL018, WUI006, WUI007, WUI008, WUI009	A	B
SS53435	Bi-LN	leg bilateral interneuron	A	A
SS53438	wing control MN	i2 MN	B	A
SS54445	Bi-LN, Uni-IN	WBL013	B	B
SS54474	Bi-IN	NBI004, NBI006, NBI007	B	A
SS54480	Uni-IN, Bi-IN	NUI001, NBI003, NBI004, NBI007	B	A
SS54495	Uni-IN, Bi-IN	NUI001, NBI006, NBI007, NBI008, NBI009	B	A
SS54506	Uni-LN	HUL001, HUL002, HUL003, HUL004, HUL005, HUL007, HUL008, HUL009	B	A
SS60603	Bi-IN	NBI001, XBI001, C2BI005	B	A

**Table 4:** Previous power muscle and motoneuron terminology used in the literature The terminology used to name dorsolongitudinal (DLM) and dorsoventral (DVM) power muscles, their muscle fibers or their motoneurons in 105 previous articles from 1970 to present is summarized. The fly DLM comprises five muscle fibers, which have been assigned been named by some studies in order from ventral to dorsal (V to D) and by others from dorsal to ventral (D to V). The terminology of this study is described in the top row.

year of publication	animals	DLM fiber names	DLM fiber order	dorsal fiber neuron names	ventral fiber neuron names	DVM muscle names	DVM fiber names	DVM fiber neuron names	Reference	citation
2023	*Drosophila melanogaster*	DLMa-f	D to V	DLMNa/b	DLMNc-f	DVM1-3	DVM1a-c,2a-b,3a-b	DVMN1a-c,2a-b,3a-b		This study
1973	*Drosophila melanogaster*	DLM-I,-II	V to D			DVM-I,-II,-III			1	Levine, J.D., & Wyman, R.J. (1973). Neurophysiology of flight in wild-type and a mutant *Drosophila*. *Proc. Nat. Acad. Sci. USA, 70*(4), 1050–1054
1977	*Drosophila melanogaster*	DLM I,II	V to D			DVM I,II,III			2	Ewing, A.W. (1977) The neuromuscular basis of courtship song in *Drosophila*: The role of the indirect flight muscles. *J. comp. Physiol. 119*, 249–265.
1996	*Drosophila melanogaster*	DLM				DVM I,II,III			3	Fernandes, J.J., Celniker, S.E., & VijayRaghavan, K. (1996). Development of the indirect muscle attachment sites in *Drosophila:* Role of the PS integrins and the *stripe* gene. *Developmental Biology*
1979	*Drosophila melanogaster*	DLM				DVM I,II,III			4–7	Ewing, A.W. (1979). The neuromuscular basis of courtship song in *Drosophila*: The role of the direct and axillary wing muscles. *J. Comp. Physiol. 130*, 87–93.
1993	*Drosophila melanogaster*	DLM				DVM I,II,III				Edgecomb, R.S., Ghetti, C., & Schneiderman, A.M. (1993). *Bendless* alters thoracic musculature in *Drosophila*. *Journal of Neurogenetics 8*(4), 209–219. DOI: 10.3109/01677069309083449
1997	*Drosophila melanogaster*	DLM				DVM I,II,III				Sandstrom, D.J., Bayer, C.A., Fristrom, J.W., & Resifo, L.L. (1997). *Broad-Complex* transcription factors regulate thoracic muscle attachment in *Drosophila. Developmental Biology, 181*, 168–185.
1999	*Drosophila melanogaster*	DLM				DVM I,II,III				Sandstrom, D.J., & Restifo, L.L. (1999). Epidermal tendon cells require *Broad Complex* function for correct attachment of the indirect flight muscles in *Drosophila melanogaster*. *Journal of Cell Science*
2001	*Drosophila melanogaster*	DLM				DVM-I,-II,-III			8	Rivlin, P.K., Gong, A., Schneiderman, A.M., & Booker, R. (2001). The role of *Ultrabithorax* in the patterning of adult thoracic muscles in *Drosophila melanogaster. Dev Genes Evol 211*, 55–66. DOI
2006	*Drosophila melanogaster*	DLM		mn	mn	DVM-I,-II,-III		mn	9	Gordon, S., & Dickinson, M.H. (2006). Role of calcium in the regulation of mechanical power in insect flight. *PNAS 103*(11), 4311–4315. DOI 10.1073/pnas.0510109103
1980	*Drosophila melanogaster*	DLM		DLM mn	DLM mn	DVM I,II,III			10	Tanouye, M.A., & Wyman, R.J. (1980). Motor outputs of the giant fiber in *Drosophila. Journal of Neurophysiology, 44*(2), 405–421.
1983	*Drosophila melanogaster*	DLM		DLM mn	DLM mn				11	Thomas, J.B., & Wyman, R.J. (1983). Normal and mutant connectivity between identified neurons in *Drosophila. TINS* 214–219.
1994	*Drosophila melanogaster*	DLM		DLMmn	DLMmn	DVM I,II,III			12	Euk Oh, C., McMahon, R., Benzer, S., & Tanouye, M.A. (1994). *bendless*, a *Drosophila* gene affecting neuronal connectivity, encodes a Ubiquitin-conjugating enzyme homolog. *The Journal of*
2015	*Drosophila melanogaster*	DLM		DLMmn	DLMmn				13	Kroll, J.R., Wong, K.G., Siddiqui, F.M., & Tanouye, M.A. (2015). Disruption of endocytosis with the dynamin mutant *shibirets1* suppresses seizures in *Drosophila. Genetics, 201*(3), 1087–1102. doi:
2018	*Drosophila melanogaster*	DLM		DLMn, DLMmn	DLMn, DLMmn				14	Augustin, H., McGourty, K., Allen, M.J., Adcott, J., Wong, C.T., Boucrot, E., & Partridge, L. (2018). Impact of insulin signaling and proteasomal activity on physiological output of a neuronal circuit in aging *Drosophila melanogaster. Neurobiology of Aging, 66*, 149–157. doi:
1990	*Drosophila melanogaster*	DLM		DLMN	DLMN				15	Baird, D.H., Schalet, A.P., & Wyman, R.J. (1990). The *Passover* locus in *Drosophila melanogaster*: Complex complementation and different effects on the giant fiber neural pathway. *Genetics, 126*,
1999	*Drosophila melanogaster*	DLM		DLMn	DLMn				16–29	Allen, M.J., Shan, X., Caruccio, P., Froggett, S.J., Moffat, K.G., & Murphey, R.K. (1999). Targeted expression of truncated *Glued* disrupts giant fiber synapse formation in *Drosophila. The Journal of*
2000	*Drosophila melanogaster*	DLM		DLMn	DLMn					Allen, M.J., Shan, X., & Murphey, R.K. (2000). A role for *Drosophila* Drac1 in neurite outgrowth and synaptogenesis in the giant fiber system. *Molecular and Cellular Neuroscience, 16*, 754–765. doi:
2002	*Drosophila melanogaster*	DLM		DLMn	DLMn					Godenschwege, T.A., Hu, H., Shan-Crofts, X., Goodman, C.S., & Murphey, R.K. (2002). Bi-directional signaling by Semaphorin 1a during central synapse formation in *Drosophila. Nature*
2003	*Drosophila melanogaster*	DLM		DLMn	DLMn					Murphey, R.K., Froggett, S.J., Caruccio, P., Shan-Crofts, X., Kitamoto, T., & Godenschwege, T.A. (2003). Targeted expression of *shibirets* and *semaphorin 1a* reveals critical periods for synapse formation in the giant fiber of *Drosophila. Development, 130*(16), 3671–3682. doi:
2007	*Drosophila melanogaster*	DLM		DLMn	DLMn					Allen, M.J., & Murphey, R.K. (2007). The chemical component of the mixed GF-TTMn synapse in *Drosophila melanogaster* uses acetylcholine as its neurotransmitter. *European Journal of*
2008	*Drosophila melanogaster*	DLM		DLMn	DLMn					Uthaman, S.B., Godenschwege, T.A., & Murphey, R.K. (2008). A mechanism distinct from Highwire for the *Drosophila* ubiquitin conjugase Bendless in synaptic growth and maturation. *The Journal of*
2009	*Drosophila melanogaster*	DLM		DLMn	DLMn					Godenschwege, T.A., & Murphey, R.K. (2009). Genetic interaction of Neuroglian and Semaphorin1a during guidance and synapse formation. *Journal of Neurogenetics, 23*(1–2), 147–155. DOI:
2010	*Drosophila melanogaster*	DLM		DLMn	DLMn					Zhao, X.-L., Wang, W.-A., Tan, J.-X., Huang, J.-K., Zhang, X., Zhang, B.-Z., Wang, Y.-H., YangCheng, H.-Y., Zhu, H.-L., Sun, X.-J., & Huang, F.-D. (2010). Expression of ß-Amyloid induced age-dependent presynaptic and axonal changes in *Drosophila. The Journal of Neuroscience*,
2010	*Drosophila melanogaster*	DLM		DLMn	DLMn					Meija, M., Heghinian, M.D., Busch, A., Armishaw, C.J., Mari, F., & Godenschwege, T.A. (2010). A novel approach for *in vivo* screening of toxins using the *Drosophila* Giant Fiber circuit. *Toxicon, 56*(8), 1398–1407. doi: 10.1016/toxicon.2010.08.005
2012	*Drosophila melanogaster*	DLM		DLMn	DLMn					Mejia, M., Heghinian, M.D., Busch, A., Mari, F., & Godenschwege, T.A. (2012). Paired nanoinjection and electrophysiology assay to screen for bioactivity of compounds using the *Drosophila melanogaster* Giant Fiber System. *Journal of Visualized Experiments, (62)*, 3597. doi: 10.3791/3597
2014	*Drosophila melanogaster*	DLM		DLMn	DLMn					Lin, J.-Y., Wang, W.-A., Zhang, X., Liu, H.-Y., Zhao, X.-L., & Huang, F.-D. (2014). Intraneuronal accumulation of Aß42 induces age-dependent slowing of neuronal transmission in *Drosophila*. *Neurosci Bull 30*(2), 185–190. DOI: 10.1007/s12264-013-1409-9
2016	*Drosophila melanogaster*	DLM		DLMn	DLMn					Pezier, A.P., Jezzini, S.H., Bacon, J.P., & Blagburn, J.M. (2016). Shaking B mediates synaptic coupling between auditory sensory neurons and the giant fiber of *Drosophila melanogaster. PLoS ONE, 11*(4), e0152211. doi: 10.1371/journal.pone.0152211
2017	*Drosophila melanogaster*	DLM		DLMn	DLMn					Borgen, M., Rowland, K., Boerner, J., Lloyd, B., Khan, A., & Murphey, R. (2017). Axon termination, pruning, and synaptogenesis in the giant fiber system of *Drosophila melanogaster* is promoted by Highwire. *Genetics, 205*(3), 1229–1245. doi: 10.1534/genetics.116.197343
2019	*Drosophila melanogaster*	DLM		DLMn	DLMn					Lee, J., Iyengar, A., & Wu, C.-F. (2019). Distinctions among electroconvulsion- and proconvulsant-induced seizure discharges and native motor patterns during flight and grooming: quantitative spike pattern analysis in *Drosophila* flight muscles. *Journal of Neurogenetics, 33*(2). doi:
2013	*Drosophila melanogaster*	DLM		DLMn	DLMn c-f				30	Huang, J.-K., Ma, P.-L., Ji, S.-Y., Zhao, X.-L., Tan, J.-X., Sun, X.-J., & Huang, F.-D. (2013). Age-dependent alterations in the presynaptic active zone in a *Drosophila* model of Alzheimer's disease. *Neurobiology of Disease, 51*, 161–167. doi: 10.1016/j.nbd.2021.11.06
1988	*Drosophila melanogaster*	a-f	D to V	DLMn a/b	DLMn c-f	DVM I,II,III			31	Hummon, M.R., & Costello, W.J. (1988). Induced neuroma formation and target muscle perturbation in the giant fiber pathway of the *Drosophila* temperature-sensitive mutant *shibire*. *Roux's Arch Dev*
1989	*Drosophila melanogaster*	DLM fiber a-f	D to V	DLMn a/b	DLMn c-f	DVM I,II,III			32	De la Pompa, J.L., Garcia, J.R., & Ferrus, A. (1989). Genetic analysis of muscle development in *Drosophila melanogaster*. *Developmental Biology 131*, 439–454.
1993	*Drosophila melanogaster*	DLM fibre a-f	D to V	DLM motoneuron a/b		DVM I,II,III			33	Hummon, M.R., & Costello, W.J. (1993). Flight muscle formation in *Drosophila* mosaics: Requirement for normal *shibire* function of endocytosis. *Roux's Arch Dev Biol 202*, 95–102.
1978	*Drosophila melanogaster*	DLM a-f	D to V	DLMa,b motoneuron	DLMc-f motoneuron				34	Coggshall, J.C. (1978). Neurons associated with the dorsal longitudinal flight muscles of *Drosophila melanogaster*. *J. Comp. Neur. 177*, 707–720.
1997	*Drosophila melanogaster*	DLMa-f	D to V	DLMn a/b	DLMn c-f	DVM I,II,III			35	Sun, Y.-A., & Wyman, R.J. (1997). Neurons of the *Drosophila* Giant Fiber System: I. Dorsal Longitudinal Motor Neurons. *The Journal of Comparative Neurology, 387*, 157–166.
1992	*Drosophila melanogaster*	DLMa-f	D to V	DLMn a/b	DLMn c-f				36, 37	Engel, J.E., & Wu, C.-F. (1992). Interactions of membrane excitability mutations affecting potassium and sodium currents in the flight and giant fiber escape systems of *Drosophila. J Comp Physiol A*,
2002	*Drosophila melanogaster*	DLMa-f	D to V	DLMn a/b	DLMn c-f					Lee, J., & Wu, C.-F. (2002). Electroconvulsive seizure behavior in *Drosophila*: Analysis of the physiological repertoire underlying a stereotyped action pattern in bang-sensitive mutants. *The*
1981	*Drosophila melanogaster*	DLMa-f	D to V	a/b					38	Tanouye, M.A., & Wyman, R.J. (1981). Inhibition between flight motoneuron in *Drosophila. J Comp Physiol, 144*, 345–355.
1992	*Drosophila melanogaster*	DLM a-f	D to V			DVM I,II,III			39	Hummon, M.R., & Costello, W.J. (1992). Cell lineage of flight muscle fibers in *Drosophila*: A fate map of induced *shibire* phenotype in mosaics. *Roux's Arch Dev Biol 201*, 88–94.
1986	*Drosophila melanogaster*	a-f	D to V			DVM I,II,III			40	Costello, W.J., & Wyman, R.J. (1986). Development of an indirect flight muscle in a muscle-specific mutant of *Drosophila melanogaster. Developmental Biology, 118*(1), 247–258. DOI: 10.1016/0012-
2008	*Drosophila melanogaster*	a-f	D to V			I,II,III			41	Atreya, K.B., & Fernandes, J.J. (2008). Founder cells regulate fiber number but not fiber formation during adult myogenesis in *Drosophila*. *Developmental Biology, 321*(1), 123–140. doi:
1996	*Drosophila melanogaster*	a-f	D to V						42–45	Farrell, E.R., Fernandes, J., & Keshishian, H. (1996). Muscle organizers in *Drosophila*: The role of persistent larval fibers in adult flight muscle development. *Developmental Biology 176*, 220–229.
2004	*Drosophila melanogaster*	a-f	D to V							Hebbar, S., & Fernandes, J.J. (2004). Pruning of motor neuron branches establishes the DLM innervation pattern in *Drosophila. Journal of Neurobiology, 60*(4), 499–516.
2004	*Drosophila melanogaster*	a-f	D to V							Banerjee, S., Lee, J., Wu, C.-F., & Hasan, G. (2004). Loss of flight and associated neuronal rhythmicity in inositol 1,4,5-triphosphate receptor mutants of *Drosophila. The Journal of Neuroscience, 24*(36), 7869–7878. DOI: 10.1523/JNEUROSCI.0656-04.2004
2019	*Drosophila melanogaster*	a-f	D to V							Chaturvedi, D., Prabhakar, S., Aggarwal, A., Atreya, K.B., & VijayRaghavan, K. (2019). Adult *Drosophila* muscle morphometry through microCT reveals dynamics during ageing. *Open Biol. 9: 190087* http://dx.doi.org/10.1098/rsob.190087
1991	*Drosophila melanogaster*	DLM a-f	V to D			DVM I,II,III			46	Fernandes, J., Bate, M., & VijayRaghavan, K. (1991). Development of the indirect flight muscles of *Drosophila. Development 113*, 67–77.
1995	*Drosophila melanogaster*	a-f	V to D			I,II,III			47	Lee, J.C., VijayRaghavan, K., Celniker, S.E., & Tanouye, M.A. (1995). Identification of a *Drosophila* muscle development gene with structural homology to mammalian early growth response transcription factors. *Proc. Natl. Acad. Sci. USA 92*, 10344–10348.
1996	*Drosophila melanogaster*	a-f	unclear			DVM I,II,III			48	Fernandes, J.J., & Keshishian, H. (1996). Patterning the dorsal longitudinal flight muscles (DLM) of *Drosophila*: Insights from the ablation of larval scaffolds. *Development 122*, 3755–3763.
1998	*Drosophila melanogaster*	DLM a-f	D to V	MN 5	MN 1–4	DVM I,II,III			49	Fernandes, J.J., & Keshishian, H. (1998). Nerve-muscle interactions during flight muscle development in *Drosophila*. *Development 125*, 1769–1779.
1999	*Drosophila melanogaster*	a-f	D to V	MN5					50	Schmid, A., Chiba, A., & Doe, C.Q. (1999). Clonal analysis of *Drosophila* embryonic neuroblasts: neural cell types, axon projections and muscle targets. *Development 126*, 4653–4689
2005	*Drosophila melanogaster*	DLM a-f	D to V	MN 5					51, 52	Hebbar, S., & Fernandes, J.J. (2005). A role for Fas II in the stabilization of motor neuron branches during pruning in *Drosophila. Developmental Biolog, 285*(1), 185–199. doi:
2010	*Drosophila melanogaster*	DLM a-f	D to V	MN 5						Hebbar, S., & Fernandes, J.J. (2010). Glial remodeling during metamorphosis influences the stabilization of motor neuron branches in *Drosophila. Developmental Biology, 340*(2), 344–354. doi:
2006	*Drosophila melanogaster*	DLMa-f	unclear	DLMn	DLMn				53, 54	Lee, J., & Wu, C.-F. (2006). Genetic modifications of seizure susceptibility and expression by altered excitability in *Drosophila* Na+ and K+ channel mutants. *J Neurophysiol 96*, 2465–2478.
2013	*Drosophila melanogaster*	DLMa-f	unclear	DLMn	DLMn					Zhang, T., Wang, Z., Wang, L., Luo, N., Jiang, L., Liu, Z., Wu, C.-F., & Dong, K. (2013). Role of the DSC1 channel in regulating neuronal excitability in *Drosophila melanogaster*: Extending nervous system stability under stress. *PLOS Genetics*. doi: 10.1371/journal.pgen.1003327
1996	*Drosophila melanogaster*	DLMa-f	unclear						55	Engel, J.E., & Wu, C.-F. (1996). Altered habituation of an identified escape circuit in *Drosophila* memory mutants. *The Journal of Neuroscience, 16*(10), 3486–3499.
2013	*Drosophila melanogaster*	DLM45a-f	D to V			DVM47,48,46	DVM47a-c,48a-b,46ab		56	Lehmann, F.-O., Skandalis, D.A., & Berthe. (2013). Calcium signalling indicates bilateral power balancing in the *Drosophila* flight muscle during manoeuvring flight. *J R Soc Interface 10*, 20121050.
1992	*Drosophila melanogaster*	DLM45a-f	D to V			DVM I,II,III	47a-c,48a-b,46a-b		57	Restifo, L.L., & White, K. (1992) Mutations in a steroid hormone-regulated gene disrupt the metamorphosis of internal tissues in *Drosophila*: Salivary glands, muscle, and gut. *Roux's Arch Dev*
1984	*Drosophila melanogaster*	DLM45a-f	D to V	DLMn 45a/b	DLMn 45c-f	DVM I,II,III	47a-c,48a-b,46a-b		58	Gorczyca, M., & Hall, J.C. (1984). Identification of a cholinergic synapse in the giant fiber pathway of *Drosophila* using conditional mutations of acetylcholine synthesis. *Journal of Neurogenetics, 1*(4),
1977	*Drosophila melanogaster*	45a-f	D to V	45a/b	45c-f	47,48,46	47a-c,48a-b,46a-b		59, 60	Harcombe, E.S., & Wyman, R.J. (1977). Output pattern generation by *Drosophila* flight motoneurons. *Journal of Neurophysiology, 40*(5), 1066–1077.
1978	*Drosophila melanogaster*	45a-f	D to V	45a/b	45c-f	47,48,46	47a-c,48a-b,46a-b			Harcombe, E.S., & Wyman, R.J. (1978). The cyclically repetitive firing sequences of identified *Drosophila* flight motoneurons. *J. comp. Physiol., 123*, 271–279.
1986	*Drosophila melanogaster*	45a-f	D to V						61, 62	Elkins, T., Ganetzky, B., & Wu, C.-F. (1986). A *Drosophila* mutation that eliminates a calcium-dependent potassium current. *Proc. Natl. Acad. Sci. USA, 83*, 8415–8419.
2017	*Drosophila melanogaster*	45a-f	D to V							De Rose, F., Marotta, R., Talani, G., Catelani, T., Solari, P., Poddighe, S., Borghero, G., Marrosu, F., Sanna, E., Kasture, S., Acquas, E., & Liscia, A. (2017). Differential effects of phytotherapic preparations in the hSOD1 *Drosophila melanogaster* model of ALS. *Scientific Reports, 7*, 41059.
2010	*Drosophila melanogaster*	DLM 45a-f	D to V	DLMn	DLMn				63	Allen, M.A., & Godenschwege, T.A. (2010). Electrophysiological recordings from the *Drosophila* giant fiber system (GFS). *Cold Spring Harb Protoc, 7*. doi:10.1101/pdb.prot5453.
2012	*Drosophila melanogaster*	1–6	V to D	MN5	DLMn1–4				64	Kadas, D., Tzortzopoulos, A., Skoulakis, E.M.C., & Consoulas, C. (2012). Constitutive activation of Ca2+/Calmodulin-Dependent Protein Kinase II during development impairs central cholinergic transmission in a circuit underlying escape behavior in *Drosophila. The Journal of Neuroscience*,
1983	*Drosophila melanogaster*	1–6	V to D	MN5	MN1–4				65, 66	Koenig, J.H., & Ikeda, K. (1983). Reciprocal excitation between identified flight motor neurons in *Drosophila* and its effect on pattern generation. *J Comp Physiol, 150*, 305–317.
2014	*Drosophila melanogaster*	1–6	V to D	MN5	MN1–4					Hutchinson, K.M., Vonhoff, F., & Duch, C. (2014). Dscam1 is required for normal dendrite growth and branching but not for dendritic spacing in *Drosophila* motoneurons. *The Journal of Neuroscience*
1980	*Drosophila melanogaster*	1–6	V to D	MN5/6	MN1–4				67	Koenig, J.H., & Ikeda, K. (1980). Neural interactions controlling timing of flight muscle activity in *Drosophila*. *J. exp. Biol. 87*, 121–136.
1983	*Drosophila melanogaster*	1–6	V to D		DLM motor neuron 14				68	Koenig, J.H., & Ikeda, K. (1983). Characterization of the intracellularly recorded response of identified flight motor neurons in *Drosophila. J Comp Physiol, 150*, 295–303.
1973	*Drosophila melanogaster*	DLM-I(1),(2),,,(5), V to D DLM-II(6)	5,6	1–4	DVM-I,-II,-III	T,A,S,R,C,L,O			69	Levine, J., & Tracey, D. (1973). Structure and function of the giant motorneuron of *Drosophila melanogaster. J. comp. Physiol. 87*, 213–235.
1973	*Drosophila melanogaster*	1–6	V to D			DVM-I,-II,-III	T,A,S,R,C,L,O		70	Levine, J.D., & Hughes, M. (1973). Stereotaxic map of the muscle fibers in the indirect flight muscles of *Drosophila melanogaster. J. Morph., 140*, 153–158.
1981	*Drosophila melanogaster*	1–6	V to D				T,A,S,R,C,L,O		71, 72	Benshalom, G., & Dagan, D. (1981). Electrophysiological analysis of the temperature-sensitive paralytic *Drosophila* mutant, *para ts. J Comp Physiol, 144*, 409–417.
1985	*Drosophila melanogaster*	1–6	V to D				T,A,S,R,C,L,O			Benshalom, G., & Dagan, D. (1985). *Drosophila* neural pathways: Genetic and electrophysiological analysis. *J Comp Physiol, 156*, 13–23.
1980	*Drosophila melanogaster*	1–6	V to D						73, 74	Ikeda, K., Koenig, J.H., & Tsuruhara, T. (1980). Organization of identified flight muscle of *Drosophila melanogaster*. *Journal of Neurocytology, 9*, 799–823
1989	*Drosophila melanogaster*	1–6	V to D							Wang, D., Keng, Z.C., Hsu, K., & Tan, C.C. (1989) *Drosophila* mutants with progressive atrophy in dorsal longitudinal muscles. *Journal of Neurogenetics, 6*(1), 27–39. DOI:
2015	*Drosophila melanogaster*	1–6	V to D		MN1–4				75	Koenig, J.H., Goto, J.J., & Ikeda, K. (2015). Novel NMDA receptor-specific desensitization/inactivation produced by ingestion of the neurotoxins, ß-N-methylamine-L-alanine (BMAA) or ß-N-oxalylamino-L-alanine (BOAA/ß-ODAP). *Comparative Biochemistry and Physiology Part C: Toxicology & Pharmacology, 167*, 43–50. doi: 10.1016/j.cbpc.2014.08.006
2016	*Drosophila melanogaster*	1–6	V to D			I,II,III			76	Rai, M., Katti, P., & Nongthomba, U. (2016). Spatio-temporal coordination of cell cycle exit, fusion and differentiation of adult muscle precursors by *Drosophila* Erect wing (Ewg). *Mechanisms of Development, 141*, 109–118. doi: 10.1016/j.mod.2016.03.004
1995	*Drosophila melanogaster*	DLM1–6	V to D	DLMn5	DLMn1–4	I,II,III	Ia-c,IIa-b,IIIa-b	DVMn Ia-c,IIa-b,IIIa-b	77	Trimarchi, J.R., & Schneiderman, A.M. (1995). Flight initiations in *Drosophila melanogaster* are mediated by several distinct motor patterns. *J Comp Physiol A 176*, 355–364.
2005	*Drosophila melanogaster*	DLM1–6	V to D	DLMn5	DLMn1–4				78	Glasscock, E., & Tanouye, M.A. (2005). *Drosophila* couch potato mutants exhibit complex neurological abnormalities including epilepsy phenotypes. *Genetics, 169*(4), 2137–2149. doi:
1995	*Drosophila melanogaster*	DLM1–6	V to D	DLMmn5	DLMmn1–4				79	Pavlidis, P., & Tanouye, M.A. (1995). Seizures and failures in the giant fiber pathway of *Drosophila* bang-sensitive paralytic mutants. *The Journal of Neuroscience, 15*(8), 5810–5819.
1988	*Drosophila melanogaster*	DLM1–6	V to D	MN5	MN1–4				80–83	Ikeda, K., & Koenig, J.H. (1988). Morphological identification of the motor neurons innervating the dorsal longitudinal flight muscle of *Drosophila melanogaster. The Journal of Comparative Neurology*,
2002	*Drosophila melanogaster*	DLM1–6	V to D	MN5	MN1–4					Consoulas, C., Restifo, L.L., & Levine, R.B. (2002). Dendritic remodeling and growth of motoneurons during metamorphosis of *Drosophila melanogaster. The Journal of Neuroscience 22*(12), 4906–4917.
2014	*Drosophila melanogaster*	DLM1–6	V to D	MN5	MN1–4					Ryglewski, S., Kadas, D., Hutchinson, K., Schuetzler, N., Vonhoff, F., & Duch, C. (2014). Dendrites are dispensible for basic motoneuron function but essential for fine tuning of behavior. *PNAS 111*(50), 18049–18054. doi/10.1073/pnas.1416247111
2019	*Drosophila melanogaster*	DLM1–6	V to D	MN5	MN1–4					Kadas, D., Duch, C., & Consoulas, C. (2019). Postnatal increase in axonal conduction velocity of an identified *Drosophila* interneuron require fast sodium, L-type calcium and Shaker potassium channels. *eNeuro, 6*(4), doi: 10.1523/ENEURO.0181-19.2019
2013	*Drosophila melanogaster*	DLM1–6	D to V						84	Rai, M., & Nongthomba, U. (2013). Effect of myonuclear number and mitochondrial fusion on *Drosophila* indirect flight muscle organization and size. *Experimental Cell Research, 319*(17), 2566-
2005	*Drosophila melanogaster*	DLM fiber 1–6	unclear						85	Koenig, J.H., & Ikeda, K. (2005). Relationship of the reserve vesicle population to synaptic depression in the tergotrochanteral and dorsal longitudinal muscles of *Drosophila. Journal of Neurophysiology, 94*(3), 2111–2119. doi: 10.1152/jn.00323.2005
1993	*Drosophila melanogaster*	DLM		MN 5		DVM I,II,III			86	Fernandes, J., & VijayRaghavan, K. (1993). The development of indirect flight muscle innervation in *Drosophila melanogaster*. *Development 118*, 215–227.
2009	*Drosophila melanogaster*	DLM		MN5	MN1				87	Ryglewski, S., & Duch, C. (2009). *Shaker* and *Shal* mediate transient calcium-independent potassium current in a *Drosophila* flight motoneuron. *Journal of Neurophysiology, 102*(6), 3673–3688. doi: 10.1152/jn.00693.2009.
	*Drosophila, Calliphora, Muscina*,	DL I-VI	D to V			DV I, II, III			88	Nachtigall, W., & Wilson, D.M. (1967). Neuro-muscular control of Dipteran flight. *J. Exp. Biol. 47*, 77–97.
1977	*Drosophila, Calliphora, Musca*,	DLM 1–6	V to D	MN 5	MN 1–4				89	Ikeda, K. (1977) Flight motor innervation of a flesh fly. In: Hoyle G. editor. *Identified Neurons and Behavior of Arthropods*. Springer. pp. 357–358.
1985	19 different Diptera fly species, including *Drosophila, Calliphora, Musca*,	d.l.m. I-VI	V to D			d.v.m. I,II,III			90	Miyan, J.A., & Ewing, A.W. (1985). How Diptera move their wings: A re-examination of the wing base articulation and muscle systems concerned with flight. *Philosophical Transactions of the Royal Society of London. Series B, Biological Sciences, 311*(1150), 271–302.
1979	*Tipula paludosa* crane fly	dlm				dvm1			91	Heide, G. (1979). Proprioceptive feedback dominates the central oscillator in the patterning of the flight motoneuron output in *Tipula* (Diptera). *J. Comp. Physiol.*, *134*, 177–189.
2007	*Calliphora erythrocephala* bl	DLM a-f	D to V	Mn-DLM a/b	Mn-DLM c-f	DVM 1,2,3	DVM 1a-c,2a-b,3a-b	Mn-DVM 1a-c,2a-b,3a-b	92	Schlurmann, M., & Hausen, K. (2007). Motoneurons of the flight power muscles of the blowfly *Calliphora erythrocephala*: Structures and mutual dye coupling. *The Journal of Comparative* Neurology, 500, 448–464.
1980	*Musca domestica* house	DLM 1–6	V to D						93	Adams, M.E., & Miller, T.A. (1980). Neural and behavioral correlates of pyrethroid and DDT-type poisoning in the house fly, *Musca domestica* L. *Pesticide Biochemistry and Physiology, 13*(2), 137–147. doi: 10.1016/0048-3575(80)90065-6.
2001	*Manduca sexta* hawkmoth	DLM1–5	V to D	MN5	MN1–4				94	Bayline, R.J., Duch, C. & Levine, R.B. (2001). Nerve-muscle interactions regulation motor terminal growth and myoblast distribution during muscle development. *Developmental Biology 231*, 348–363. doi:10.1006/dbio.2001.0158.
2000	*Manduca sexta* hawkmoth	1–5	V to D	MN5					95, 96	Duch, C., Bayline, R.J., & Levine, R.B. (2000). Postembryonic development of the dorsal longitudinal flight muscle and its innervation in *Manduca sexta. The Journal of Comparative Neurology, 422*, 1–17.
2000	*Manduca sexta* hawkmoth	1–5	V to D	MN5						Duch, C., & Levine, R.B. (2000). Remodeling of membrane properties and dendritic architecture accompanies the postembryonic conversion of a slow into a fast motoneuron. *The Journal of* Neuroscience, 20(18), 6950–6961.
2003	*Manduca sexta* hawkmoth	DLM		MN5	MN1–4				97	Duch, C., & Mentel, T. (2003). Stage-specific activity patterns affect motoneuron axonal retraction and outgrowth during the metamorphosis of *Manduca sexta. European Journal of Neuroscience, 17*, 945–962.
2004	*Manduca sexta* hawkmoth	dl1a-e	V to D						98	Tu, M.S., & Daniel, T.L. (2004). Submaximal power output from the dorsolongitudinal flight muscles of the hawkmoth *Manduca sexta. J Exp Biol, 207*(26), 4651–4662. doi: 10.1242/jeb.01321.
2011	*Manduca sexta* hawkmoth	DLM1a-e	V to D						99, 100	George, N.T., & Daniel, T.L. (2011). Temperature gradients in the flight muscles of *Manduca sexta* imply a spatial gradient in muscle force and energy output. *J Exp Biol, 214*(6), 894–900. doi: 10.1242/jeb.047969.
2012	*Manduca sexta* hawkmoth	DLM1a-e	V to D							George, N.T., Sponberg, S., & Daniel, T.L. (2012). Temperature gradients drive mechanical energy gradients in the flight muscle of *Manduca sexta. J Exp Biol, 215*(3), 571–579. doi: 10.1242/jeb.062901.
1998	*Agrius convolvuli*	DLM(i) - (iv)	D to V						101	Komai, Y. (1998). Augmented respiration in a flying insect. *The Journal of Experimental Biology 201*, 2359–2366.
2004	*Agrius convolvuli*	DL1a-e	V to D			DV1	DV1a-c		102	Ando, N., & Kanzaki, R. (2004). Changing motor patterns of the 3rd axillary muscle activities associated with longitudinal control in freely flying hawkmoths. *Zoological Science, 21*, 123–130.
	*Agrius convolvuli*	DLM1		DL1-MNs		DVM1, DVM2		DV-MNs	103	Ando, N., Wang, H., Shirai, K., & Kanzaki, R. (2011). Central projections of the wing afferents in the hawkmoth, *Agrius convolvuli. Journal of Insect Physiology, 51*(11), 1518–1536. doi: 10.1016/j.jinsphys.2011.08.002.
1982	*Bombyx mori* silkmoth	DLM1a-e,2,3	V to D	DLM1e motoneurone	DLM1a-d motoneurone	DVM1,2,3,4,5	DVM1a,1b,2,3,4,5	DVM1a,1b,2,3,4,5 motoneurone	104	Kondoh, Y., & Obara, Y. (1982). Anatomy of motoneurones innervating mesothoracic indirect flight muscles in the silkmoth *Bombyx mori. J. exp. Biol.*, 98, 23–37.
1970	*Hyalaphora cecropia*	dl1a-e	V to D			dv1,2,3,5	dv1a,1b,2,3,5		105	Hanegan, J.L., & Heath, J.E. (1970). Temperature dependence of the neural control of the moth flight system. *J. Exp. Biol., 53*, 629–639.

**Table:** STAR Methods

Reagent type (species) or resource	Designation	Source or reference	Identifiers	Additional information
Genetic reagent (Drosophila melanogaster)	79C09-x-119C05	this paper	split_gal4.janelia.org:SS20796	split-GAL4 driver line targeting WBL036, WBL037
Genetic reagent (Drosophila melanogaster)	VT025966-x-VT013121	this paper	split_gal4.janelia.org:SS25478	split-GAL4 driver line targeting HUL012
Genetic reagent (Drosophila melanogaster)	VT044959-x-VT049281	this paper	split_gal4.janelia.org:SS25482	split-GAL4 driver line targeting WBL006
Genetic reagent (Drosophila melanogaster)	VT058688-x-VT018834	this paper	split_gal4.janelia.org:SS25500	split-GAL4 driver line targeting HUL011, HUL012
Genetic reagent (Drosophila melanogaster)	VT026761-x-VT026338	this paper	split_gal4.janelia.org:SS25502	split-GAL4 driver line targeting HUL002, HUL005, HUL006
Genetic reagent (Drosophila melanogaster)	VT032906-x-VT026663	this paper	split_gal4.janelia.org:SS25511	split-GAL4 driver line targeting WUL006
Genetic reagent (Drosophila melanogaster)	VT060731-x-VT033912	this paper	split_gal4.janelia.org:SS25521	split-GAL4 driver line targeting WBI001, WBI002
Genetic reagent (Drosophila melanogaster)	VT025783-x-VT041692	this paper	split_gal4.janelia.org:SS25532	split-GAL4 driver line targeting hDVMN, hi1 MN, hi2 MN
Genetic reagent (Drosophila melanogaster)	VT008133-x-VT044655	this paper	split_gal4.janelia.org:SS25553	split-GAL4 driver line targeting NBI008, NBI009, WBI011
Genetic reagent (Drosophila melanogaster)	VT037863-x-GMR_17A10	this paper	split_gal4.janelia.org:SS28361	split-GAL4 driver line targeting C2BI004
Genetic reagent (Drosophila melanogaster)	VT044650-x-GMR_83A11	this paper	split_gal4.janelia.org:SS29535	split-GAL4 driver line targeting HBI011, HBI012
Genetic reagent (Drosophila melanogaster)	VT007746-x-VT024620	this paper	split_gal4.janelia.org:SS29600	split-GAL4 driver line targeting WBI021
Genetic reagent (Drosophila melanogaster)	VT014014-x-VT037825	this paper	split_gal4.janelia.org:SS29602	split-GAL4 driver line targeting HBI018, HBI019, HBI020
Genetic reagent (Drosophila melanogaster)	GMR_24F06-x-GMR_22B12	this paper	split_gal4.janelia.org:SS29871	split-GAL4 driver line targeting WUI004, WBI021
Genetic reagent (Drosophila melanogaster)	VT014604-x-GMR_37H01	this paper	split_gal4.janelia.org:SS30330	split-GAL4 driver line targeting NBI003, NBI004
Genetic reagent (Drosophila melanogaster)	VT031084-x-VT040003	this paper	split_gal4.janelia.org:SS30816	split-GAL4 driver line targeting HBI015
Genetic reagent (Drosophila melanogaster)	VT038171-x-VT021780	this paper	split_gal4.janelia.org:SS31246	split-GAL4 driver line targeting HBI014
Genetic reagent (Drosophila melanogaster)	VT008182-x-VT022091	this paper	split_gal4.janelia.org:SS31259	split-GAL4 driver line targeting WBL031, WBI018, WBI019
Genetic reagent (Drosophila melanogaster)	VT026387-x-VT030598	this paper	split_gal4.janelia.org:SS31263	split-GAL4 driver line targeting WBL006, WBL007, WBL008
Genetic reagent (Drosophila melanogaster)	VT025966-x-VT043137	this paper	split_gal4.janelia.org:SS31274	split-GAL4 driver line targeting HUL011, HUL012
Genetic reagent (Drosophila melanogaster)	GMR_34H12-x-VT016461	this paper	split_gal4.janelia.org:SS31289	split-GAL4 driver line targeting C2BI008, C2BI009
Genetic reagent (Drosophila melanogaster)	GMR_34H12-x-GMR_77H08	this paper	split_gal4.janelia.org:SS31290	split-GAL4 driver line targeting C2BI006, C2BI008, C2BI009, C2BI010
Genetic reagent (Drosophila melanogaster)	GMR_72G07-x-VT044959	this paper	split_gal4.janelia.org:SS31309	split-GAL4 driver line targeting WBL006, WBL007, NBI007
Genetic reagent (Drosophila melanogaster)	GMR_50G08-x-GMR_41H07	this paper	split_gal4.janelia.org:SS31472	split-GAL4 driver line targeting WUL002, HUL005
Genetic reagent (Drosophila melanogaster)	104C08-x-117C08	this paper	split_gal4.janelia.org:SS31541	split-GAL4 driver line targeting DLMNs, DVMNs
Genetic reagent (Drosophila melanogaster)	104C08-x-11D09	this paper	split_gal4.janelia.org:SS31543	split-GAL4 driver line targeting DLMNs, DVMNs, hDVMN
Genetic reagent (Drosophila melanogaster)	105G03-x-31F09	this paper	split_gal4.janelia.org:SS31561	split-GAL4 driver line targeting DLMNs
Genetic reagent (Drosophila melanogaster)	GMR_26H04-x-GMR_46A10	this paper	split_gal4.janelia.org:SS31899	split-GAL4 driver line targeting NBI002
Genetic reagent (Drosophila melanogaster)	120A11-x-11D09	this paper	split_gal4.janelia.org:SS31950	split-GAL4 driver line targeting DLMNs, DVMNs
Genetic reagent (Drosophila melanogaster)	91C05-x-75F02	this paper	split_gal4.janelia.org:SS32023	split-GAL4 driver line targeting hg1 MN
Genetic reagent (Drosophila melanogaster)	VT040003-x-VT019902	this paper	split_gal4.janelia.org:SS32377	split-GAL4 driver line targeting HBI015
Genetic reagent (Drosophila melanogaster)	VT014604-x-VT034622	this paper	split_gal4.janelia.org:SS32400	split-GAL4 driver line targeting NBI003, NBI004
Genetic reagent (Drosophila melanogaster)	VT050238-x-VT043015	this paper	split_gal4.janelia.org:SS33409	split-GAL4 driver line targeting WUL003, WUL004, WUL005, WUL006
Genetic reagent (Drosophila melanogaster)	VT060731-x-VT019730	this paper	split_gal4.janelia.org:SS33437	split-GAL4 driver line targeting WBI001, WBI002
Genetic reagent (Drosophila melanogaster)	GMR_22B12-x-GMR_26C06	this paper	split_gal4.janelia.org:SS33489	split-GAL4 driver line targeting XBI001
Genetic reagent (Drosophila melanogaster)	110H04-x-42H07	this paper	split_gal4.janelia.org:SS34778	split-GAL4 driver line targeting i2 MN
Genetic reagent (Drosophila melanogaster)	74F04-x-42H07	this paper	split_gal4.janelia.org:SS34781	split-GAL4 driver line targeting i2 MN
Genetic reagent (Drosophila melanogaster)	129A02-x-73C03	this paper	split_gal4.janelia.org:SS34789	split-GAL4 driver line targeting leg MN
Genetic reagent (Drosophila melanogaster)	VT008277-x-VT005002	this paper	split_gal4.janelia.org:SS36076	split-GAL4 driver line targeting tp1 MN, hi2 MN
Genetic reagent (Drosophila melanogaster)	GMR_48D11-x-VT019771	this paper	split_gal4.janelia.org:SS36094	split-GAL4 driver line targeting C2BL001, C2BL002
Genetic reagent (Drosophila melanogaster)	11C05-x-58H05	this paper	split_gal4.janelia.org:SS37231	split-GAL4 driver line targeting hi1 MN, hi2 MN
Genetic reagent (Drosophila melanogaster)	12E04-x-128B04	this paper	split_gal4.janelia.org:SS37233	split-GAL4 driver line targeting WBL035, WBL036, WBL039
Genetic reagent (Drosophila melanogaster)	23C02-x-42H07	this paper	split_gal4.janelia.org:SS37246	split-GAL4 driver line targeting i2 MN
Genetic reagent (Drosophila melanogaster)	25C08-x-102B02	this paper	split_gal4.janelia.org:SS37252	split-GAL4 driver line targeting hg2 MN, hg3 MN
Genetic reagent (Drosophila melanogaster)	25C08-x-113C11	this paper	split_gal4.janelia.org:SS37253	split-GAL4 driver line targeting hg2 MN, hg3 MN, hDVMN, hi1 MN, T2VUM3
Genetic reagent (Drosophila melanogaster)	33H04-x-111H07	this paper	split_gal4.janelia.org:SS37262	split-GAL4 driver line targeting leg interneuron
Genetic reagent (Drosophila melanogaster)	38A10-x-39G05	this paper	split_gal4.janelia.org:SS37274	split-GAL4 driver line targeting HBI015
Genetic reagent (Drosophila melanogaster)	42B09-x-118H02	this paper	split_gal4.janelia.org:SS37294	split-GAL4 driver line targeting DLMNs, DVMNs, tt MN
Genetic reagent (Drosophila melanogaster)	42B09-x-11D09	this paper	split_gal4.janelia.org:SS37295	split-GAL4 driver line targeting DLMNs, DVMNs, hi1 MN
Genetic reagent (Drosophila melanogaster)	42B09-x-18H03	this paper	split_gal4.janelia.org:SS37296	split-GAL4 driver line targeting leg interneuron
Genetic reagent (Drosophila melanogaster)	64C05-x-12E04	this paper	split_gal4.janelia.org:SS37306	split-GAL4 driver line targeting WBL025
Genetic reagent (Drosophila melanogaster)	VT037805-x-37E07	this paper	split_gal4.janelia.org:SS37984	split-GAL4 driver line targeting WBI001
Genetic reagent (Drosophila melanogaster)	120H03-x-VT016254	this paper	split_gal4.janelia.org:SS40449	split-GAL4 driver line targeting leg interneuron
Genetic reagent (Drosophila melanogaster)	GMR_22E12-x-VT063559	this paper	split_gal4.janelia.org:SS40456	split-GAL4 driver line targeting WUI006. WUI008, WUI009
Genetic reagent (Drosophila melanogaster)	104A07-x-101A05	this paper	split_gal4.janelia.org:SS40764	split-GAL4 driver line targeting WBI001, WBI002
Genetic reagent (Drosophila melanogaster)	104B07-x-105G06	this paper	split_gal4.janelia.org:SS40765	split-GAL4 driver line targeting DLMNs, DVMNs, i2 MN, tt MN
Genetic reagent (Drosophila melanogaster)	105G06-x-114A07	this paper	split_gal4.janelia.org:SS40772	split-GAL4 driver line targeting tt MN, b2 MN, i1 MN, hg1 MN
Genetic reagent (Drosophila melanogaster)	112A07-x-127G08	this paper	split_gal4.janelia.org:SS40778	split-GAL4 driver line targeting WBL036, WBL039
Genetic reagent (Drosophila melanogaster)	112A07-x-73C07	this paper	split_gal4.janelia.org:SS40782	split-GAL4 driver line targeting WBL001, WBI002
Genetic reagent (Drosophila melanogaster)	112E12-x-107A11	this paper	split_gal4.janelia.org:SS40783	split-GAL4 driver line targeting WBL014, WBL016, WBL017, WBL020, WBL021
Genetic reagent (Drosophila melanogaster)	126A10-x-56B12	this paper	split_gal4.janelia.org:SS40851	split-GAL4 driver line targeting hb1 MN, hb2 MN
Genetic reagent (Drosophila melanogaster)	91C05-x-105G06	this paper	split_gal4.janelia.org:SS40864	split-GAL4 driver line targeting hg1 MN, hg3 MN
Genetic reagent (Drosophila melanogaster)	91C05-x-114A07	this paper	split_gal4.janelia.org:SS40865	split-GAL4 driver line targeting leg sensory
Genetic reagent (Drosophila melanogaster)	Tdc2-x-VT027316	this paper	split_gal4.janelia.org:SS40867	split-GAL4 driver line targeting T2VUM1, T2VUM2, T2VUM3, T2VUM4
Genetic reagent (Drosophila melanogaster)	Tdc2-x-25C01	this paper	split_gal4.janelia.org:SS40868	split-GAL4 driver line targeting T2VUM1, T2VUM3
Genetic reagent (Drosophila melanogaster)	109F10-x-28E12	this paper	split_gal4.janelia.org:SS40969	split-GAL4 driver line targeting hDVMN, WBI012, WBI017, WBI019, WBI020
Genetic reagent (Drosophila melanogaster)	118H02-x-47F01	this paper	split_gal4.janelia.org:SS40980	split-GAL4 driver line targeting b1 MN, iii1 MN, iii3 MN
Genetic reagent (Drosophila melanogaster)	126C10-x-28E12	this paper	split_gal4.janelia.org:SS40989	split-GAL4 driver line targeting DLMNs, DVMNs, tt MN, hDVMN, hi1 MN, hi2 MN
Genetic reagent (Drosophila melanogaster)	33F10-x-75F02	this paper	split_gal4.janelia.org:SS41027	split-GAL4 driver line targeting tt MN, b2 MN, i1 MN, iii4 MN
Genetic reagent (Drosophila melanogaster)	33H04-x-28E12	this paper	split_gal4.janelia.org:SS41029	split-GAL4 driver line targeting adominal MN
Genetic reagent (Drosophila melanogaster)	38A10-x-12G01	this paper	split_gal4.janelia.org:SS41034	split-GAL4 driver line targeting leg sensory
Genetic reagent (Drosophila melanogaster)	38D03-x-93E02	this paper	split_gal4.janelia.org:SS41039	split-GAL4 driver line targeting i1 MN, C2BI003
Genetic reagent (Drosophila melanogaster)	41A07-x-12G01	this paper	split_gal4.janelia.org:SS41041	split-GAL4 driver line targeting HUL001
Genetic reagent (Drosophila melanogaster)	52E06-x-39G05	this paper	split_gal4.janelia.org:SS41052	split-GAL4 driver line targeting tp1 MN
Genetic reagent (Drosophila melanogaster)	70B07-x-74D06	this paper	split_gal4.janelia.org:SS41068	split-GAL4 driver line targeting DLMNs, DVMNs
Genetic reagent (Drosophila melanogaster)	93E02-x-22A12	this paper	split_gal4.janelia.org:SS41075	split-GAL4 driver line targeting DVMNs, hi1 MN
Genetic reagent (Drosophila melanogaster)	VT040003-x-VT048635	this paper	split_gal4.janelia.org:SS42050	split-GAL4 driver line targeting HBI015, HBI016
Genetic reagent (Drosophila melanogaster)	GMR_96A08-x-GMR_79E01	this paper	split_gal4.janelia.org:SS42079	split-GAL4 driver line targeting NBI010, NBI011, NBI012
Genetic reagent (Drosophila melanogaster)	109G12-x-11C01	this paper	split_gal4.janelia.org:SS42385	split-GAL4 driver line targeting T2VUM2, T2VUM3, T2VUM4
Genetic reagent (Drosophila melanogaster)	22H01-x-21B02	this paper	split_gal4.janelia.org:SS42438	split-GAL4 driver line targeting WUL011, WUL012, WBL015, WBL017, WBL019, WBL021, WBI013
Genetic reagent (Drosophila melanogaster)	22H01-x-95F12	this paper	split_gal4.janelia.org:SS42439	split-GAL4 driver line targeting WUL007, WUL012, WBL016, WBL020
Genetic reagent (Drosophila melanogaster)	GMR_23B09-x-GMR_21B02	this paper	split_gal4.janelia.org:SS42442	split-GAL4 driver line targeting WUL008, WUL009, WBL015
Genetic reagent (Drosophila melanogaster)	26B04-x-39H12	this paper	split_gal4.janelia.org:SS42446	split-GAL4 driver line targeting WBI005, WBI006, WBI007, WBI009
Genetic reagent (Drosophila melanogaster)	26B04-x-76H04	this paper	split_gal4.janelia.org:SS42447	split-GAL4 driver line targeting WBL022, WUI006, WUI008, WUI009
Genetic reagent (Drosophila melanogaster)	41G04-x-109H02	this paper	split_gal4.janelia.org:SS42464	split-GAL4 driver line targeting tp2 MN, C2BI006, C2BI007, C2BI009
Genetic reagent (Drosophila melanogaster)	41G04-x-122A10	this paper	split_gal4.janelia.org:SS42465	split-GAL4 driver line targeting WBL001, WBL003, WBL005, WBL011, WBL012
Genetic reagent (Drosophila melanogaster)	64E06-x-109H02	this paper	split_gal4.janelia.org:SS42475	split-GAL4 driver line targeting XBI002, C2BI005
Genetic reagent (Drosophila melanogaster)	92A09-x-115F05	this paper	split_gal4.janelia.org:SS42493	split-GAL4 driver line targeting HBI015
Genetic reagent (Drosophila melanogaster)	95F12-x-21B02	this paper	split_gal4.janelia.org:SS42498	split-GAL4 driver line targeting WUL007, WUL008, WUL010, WUL012, WBL015
Genetic reagent (Drosophila melanogaster)	09F12-x-87G07	this paper	split_gal4.janelia.org:SS42499	split-GAL4 driver line targeting C2BI005, WBI012, HBI003
Genetic reagent (Drosophila melanogaster)	GMR_48A03-x-VT063560	this paper	split_gal4.janelia.org:SS43546	split-GAL4 driver line targeting XBL001
Genetic reagent (Drosophila melanogaster)	102A09-x-104A05	this paper	split_gal4.janelia.org:SS43972	split-GAL4 driver line targeting leg interneuron
Genetic reagent (Drosophila melanogaster)	104C08-x-33E06	this paper	split_gal4.janelia.org:SS43979	split-GAL4 driver line targeting HBI003
Genetic reagent (Drosophila melanogaster)	104C08-x-74D06	this paper	split_gal4.janelia.org:SS43980	split-GAL4 driver line targeting DLMNs, DVMNs
Genetic reagent (Drosophila melanogaster)	112C03-x-77H03	this paper	split_gal4.janelia.org:SS43995	split-GAL4 driver line targeting b2 MN, leg interneuron
Genetic reagent (Drosophila melanogaster)	119G05-x-31E10	this paper	split_gal4.janelia.org:SS44002	split-GAL4 driver line targeting WBL031, WBL032, HBI006
Genetic reagent (Drosophila melanogaster)	119G05-x-38A07	this paper	split_gal4.janelia.org:SS44003	split-GAL4 driver line targeting WBL031, WBL032, WBL033, WBI018, WBI019
Genetic reagent (Drosophila melanogaster)	119G05-x-94H03	this paper	split_gal4.janelia.org:SS44005	split-GAL4 driver line targeting WBI018
Genetic reagent (Drosophila melanogaster)	VT026034-x-VT033050	this paper	split_gal4.janelia.org:SS44009	split-GAL4 driver line targeting leg interneuron
Genetic reagent (Drosophila melanogaster)	127E05-x-26B07	this paper	split_gal4.janelia.org:SS44028	split-GAL4 driver line targeting leg interneuron
Genetic reagent (Drosophila melanogaster)	13E04-x-110D12	this paper	split_gal4.janelia.org:SS44034	split-GAL4 driver line targeting leg interneuron
Genetic reagent (Drosophila melanogaster)	14E09-x-117C08	this paper	split_gal4.janelia.org:SS44039	split-GAL4 driver line targeting DLMNs
Genetic reagent (Drosophila melanogaster)	18H03-x-52E12	this paper	split_gal4.janelia.org:SS44046	split-GAL4 driver line targeting leg interneuron
Genetic reagent (Drosophila melanogaster)	20C03-x-28H03	this paper	split_gal4.janelia.org:SS44051	split-GAL4 driver line targeting WBI018, WBI019
Genetic reagent (Drosophila melanogaster)	23B05-x-111D01	this paper	split_gal4.janelia.org:SS44054	split-GAL4 driver line targeting HBI020
Genetic reagent (Drosophila melanogaster)	23B05-x-117C08	this paper	split_gal4.janelia.org:SS44056	split-GAL4 driver line targeting DLMNs
Genetic reagent (Drosophila melanogaster)	23B05-x-127G08	this paper	split_gal4.janelia.org:SS44057	split-GAL4 driver line targeting leg interneuron
Genetic reagent (Drosophila melanogaster)	23B05-x-74D06	this paper	split_gal4.janelia.org:SS44060	split-GAL4 driver line targeting DLMNs, DVMNs
Genetic reagent (Drosophila melanogaster)	84H05-x-13E04	this paper	split_gal4.janelia.org:SS44081	split-GAL4 driver line targeting Lco leg sensory
Genetic reagent (Drosophila melanogaster)	VT050112-x-GMR_85H06	this paper	split_gal4.janelia.org:SS44276	split-GAL4 driver line targeting HBI013
Genetic reagent (Drosophila melanogaster)	GMR_50G08-x-VT039458	this paper	split_gal4.janelia.org:SS44314	split-GAL4 driver line targeting NUL001, WUL001, WUL002, HUL002
Genetic reagent (Drosophila melanogaster)	VT004417-x-VT014709	this paper	split_gal4.janelia.org:SS45385	split-GAL4 driver line targeting WBI003, WBI004, WBI007, WBI010, WBI011
Genetic reagent (Drosophila melanogaster)	GMR_23E04-x-GMR_83A12	this paper	split_gal4.janelia.org:SS45607	split-GAL4 driver line targeting WBL001, WBL005, WBL007
Genetic reagent (Drosophila melanogaster)	GMR_72E10-x-VT000629	this paper	split_gal4.janelia.org:SS45611	split-GAL4 driver line targeting HBL001
Genetic reagent (Drosophila melanogaster)	VT016254-x-120H03	this paper	split_gal4.janelia.org:SS45736	split-GAL4 driver line targeting HUI004, leg interneuron
Genetic reagent (Drosophila melanogaster)	122B04-x-11C01	this paper	split_gal4.janelia.org:SS45766	split-GAL4 driver line targetingT2VUM1, T2VUM2, T2VUM3
Genetic reagent (Drosophila melanogaster)	125A04-x-75F02	this paper	split_gal4.janelia.org:SS45772	split-GAL4 driver line targeting tt MN, b2 MN, I1 MN
Genetic reagent (Drosophila melanogaster)	127F10-x-128A07	this paper	split_gal4.janelia.org:SS45778	split-GAL4 driver line targeting i2 MN
Genetic reagent (Drosophila melanogaster)	127F10-x-75F02	this paper	split_gal4.janelia.org:SS45779	split-GAL4 driver line targeting b3 MN, iii3 MN, iii4 MN, hg1 MN
Genetic reagent (Drosophila melanogaster)	128A07-x-125C10	this paper	split_gal4.janelia.org:SS45782	split-GAL4 driver line targeting i2 MN
Genetic reagent (Drosophila melanogaster)	78B06-x-67E03	this paper	split_gal4.janelia.org:SS45830	split-GAL4 driver line targeting WBL001, WBL002, WBL004, WBL005, WBL007
Genetic reagent (Drosophila melanogaster)	95F12-x-59F02	this paper	split_gal4.janelia.org:SS45843	split-GAL4 driver line targeting WBL002, WBL003, WBL005
Genetic reagent (Drosophila melanogaster)	GMR_81C11-x-GMR_54H10	this paper	split_gal4.janelia.org:SS46260	split-GAL4 driver line targeting HUI003, HUI004, HUI005
Genetic reagent (Drosophila melanogaster)	VT046018-x-GMR_76G11	this paper	split_gal4.janelia.org:SS46295	split-GAL4 driver line targeting WBI014
Genetic reagent (Drosophila melanogaster)	VT064569-x-VT056770	this paper	split_gal4.janelia.org:SS46645	split-GAL4 driver line targeting T2VUM2
Genetic reagent (Drosophila melanogaster)	GMR_22H01-x-VT008681	this paper	split_gal4.janelia.org:SS46675	split-GAL4 driver line targeting WBL016, WBL018, WBL019
Genetic reagent (Drosophila melanogaster)	VT004417-x-VT028869	this paper	split_gal4.janelia.org:SS46725	split-GAL4 driver line targeting WBL009
Genetic reagent (Drosophila melanogaster)	GMR_12A11-x-VT009094	this paper	split_gal4.janelia.org:SS46735	split-GAL4 driver line targeting C2BI005, C2BI009, HUI002, HUI003, HUI005
Genetic reagent (Drosophila melanogaster)	107C08-x-114A09	this paper	split_gal4.janelia.org:SS47120	split-GAL4 driver line targeting tp2 MN
Genetic reagent (Drosophila melanogaster)	110D01-x-75F02	this paper	split_gal4.janelia.org:SS47125	split-GAL4 driver line targeting tt MN
Genetic reagent (Drosophila melanogaster)	123C07-x-115B11	this paper	split_gal4.janelia.org:SS47152	split-GAL4 driver line targeting ps1 MN
Genetic reagent (Drosophila melanogaster)	127F10-x-10A12	this paper	split_gal4.janelia.org:SS47160	split-GAL4 driver line targeting b2 MN, b3 MN, iii4 MN, hg1 MN, hg3 MN
Genetic reagent (Drosophila melanogaster)	127F10-x-31D08	this paper	split_gal4.janelia.org:SS47161	split-GAL4 driver line targeting hg1 MN, WUL012
Genetic reagent (Drosophila melanogaster)	44D02-x-23B05	this paper	split_gal4.janelia.org:SS47192	split-GAL4 driver line targeting WUL016, WBL034
Genetic reagent (Drosophila melanogaster)	44F09-x-121H06	this paper	split_gal4.janelia.org:SS47195	split-GAL4 driver line targeting hi1 MN, hb1 MN, hb2 MN
Genetic reagent (Drosophila melanogaster)	50G08-x-23B05	this paper	split_gal4.janelia.org:SS47200	split-GAL4 driver line targeting WBL013, XBI002
Genetic reagent (Drosophila melanogaster)	67B01-x-116H07	this paper	split_gal4.janelia.org:SS47204	split-GAL4 driver line targeting HBI007, prosternal sensory
Genetic reagent (Drosophila melanogaster)	75D06-x-67B01	this paper	split_gal4.janelia.org:SS47214	split-GAL4 driver line targeting HBI004, HBI006, HBI007
Genetic reagent (Drosophila melanogaster)	75D06-x-75F06	this paper	split_gal4.janelia.org:SS47215	split-GAL4 driver line targeting HBI005, HBI006, HBI007, HBI009, HBI019
Genetic reagent (Drosophila melanogaster)	92B11-x-112C01	this paper	split_gal4.janelia.org:SS47219	split-GAL4 driver line targeting WBL031, WBI018, WBI019
Genetic reagent (Drosophila melanogaster)	92B11-x-38A07	this paper	split_gal4.janelia.org:SS47222	split-GAL4 driver line targeting WBL031, WBI018, WBI019
Genetic reagent (Drosophila melanogaster)	105D02-x-85F12	this paper	split_gal4.janelia.org:SS48204	split-GAL4 driver line targeting WBI005, WBI006, WBI007, WBI008, WBI010, WBI011
Genetic reagent (Drosophila melanogaster)	115B11-x-23B05	this paper	split_gal4.janelia.org:SS48215	split-GAL4 driver line targeting WUL013, WUL014, WUL017
Genetic reagent (Drosophila melanogaster)	119F12-x-55H04	this paper	split_gal4.janelia.org:SS48221	split-GAL4 driver line targeting HBI019
Genetic reagent (Drosophila melanogaster)	125G04-x-103B07	this paper	split_gal4.janelia.org:SS48240	split-GAL4 driver line targeting abdominal MN
Genetic reagent (Drosophila melanogaster)	125G04-x-120A08	this paper	split_gal4.janelia.org:SS48247	split-GAL4 driver line targeting abdominal MN
Genetic reagent (Drosophila melanogaster)	23B05-x-123G02	this paper	split_gal4.janelia.org:SS48268	split-GAL4 driver line targeting T2VPM1, WUL015, WUL016, WBL031, WBL033
Genetic reagent (Drosophila melanogaster)	26A08-x-123G02	this paper	split_gal4.janelia.org:SS48272	split-GAL4 driver line targeting WBL010, WBL011, WBL012
Genetic reagent (Drosophila melanogaster)	50G08-x-114A07	this paper	split_gal4.janelia.org:SS48287	split-GAL4 driver line targeting prosternal sensory
Genetic reagent (Drosophila melanogaster)	92H07-x-52B02	this paper	split_gal4.janelia.org:SS48305	split-GAL4 driver line targeting leg interneuron
Genetic reagent (Drosophila melanogaster)	127F10-x-115A02	this paper	split_gal4.janelia.org:SS48311	split-GAL4 driver line targeting b3 MN, hg1 MN, XBI002, WBI009
Genetic reagent (Drosophila melanogaster)	VT045281-x-VT030541	this paper	split_gal4.janelia.org:SS48619	split-GAL4 driver line targeting NUI002
Genetic reagent (Drosophila melanogaster)	GMR_65D05-x-GMR_44H01	this paper	split_gal4.janelia.org:SS48709	split-GAL4 driver line targeting HUI002, HUI004, HUI005
Genetic reagent (Drosophila melanogaster)	127F10-x-25C08	this paper	split_gal4.janelia.org:SS49039	split-GAL4 driver line targeting hg3 MN
Genetic reagent (Drosophila melanogaster)	127G08-x-116H08	this paper	split_gal4.janelia.org:SS49041	split-GAL4 driver line targeting WUL004, WUL005, WBL035, WBL038
Genetic reagent (Drosophila melanogaster)	127G08-x-118C10	this paper	split_gal4.janelia.org:SS49042	split-GAL4 driver line targeting WUL003, WUL005
Genetic reagent (Drosophila melanogaster)	GMR_48A03-x-VT021731	this paper	split_gal4.janelia.org:SS49125	split-GAL4 driver line targeting HUL010
Genetic reagent (Drosophila melanogaster)	108H11-x-107A11	this paper	split_gal4.janelia.org:SS49766	split-GAL4 driver line targeting WUL012, WBL014
Genetic reagent (Drosophila melanogaster)	VT012768-x-20A03	this paper	split_gal4.janelia.org:SS49776	split-GAL4 driver line targeting PSI
Genetic reagent (Drosophila melanogaster)	VT012768-x-75F06	this paper	split_gal4.janelia.org:SS49777	split-GAL4 driver line targeting PSI, HBI001, HBI002
Genetic reagent (Drosophila melanogaster)	113F07-x-127G08	this paper	split_gal4.janelia.org:SS49778	split-GAL4 driver line targeting WUI001, WUI002
Genetic reagent (Drosophila melanogaster)	113F07-x-27A05	this paper	split_gal4.janelia.org:SS49779	split-GAL4 driver line targeting WBL023, WUI005, WBI015, WBI016
Genetic reagent (Drosophila melanogaster)	116H08-x-112B09	this paper	split_gal4.janelia.org:SS49784	split-GAL4 driver line targeting C2BI001, C2BI002, C2BI003, C2BI009
Genetic reagent (Drosophila melanogaster)	123C01-x-74D06	this paper	split_gal4.janelia.org:SS49797	split-GAL4 driver line targeting DLMNs, DVMNs
Genetic reagent (Drosophila melanogaster)	125E09-x-37E06	this paper	split_gal4.janelia.org:SS49799	split-GAL4 driver line targeting WBL013, WUI010
Genetic reagent (Drosophila melanogaster)	VT043294-x-113F07	this paper	split_gal4.janelia.org:SS49800	split-GAL4 driver line targeting WUL005, WUI001, WUI002, WUI011, WUI012
Genetic reagent (Drosophila melanogaster)	VT043294-x-24F10	this paper	split_gal4.janelia.org:SS49802	split-GAL4 driver line targeting WBI017, WBI019
Genetic reagent (Drosophila melanogaster)	137F12-x-14A02	this paper	split_gal4.janelia.org:SS49806	split-GAL4 driver line targeting b3 MN, hg1 MN, HBI017
Genetic reagent (Drosophila melanogaster)	137G02-x-127G08	this paper	split_gal4.janelia.org:SS49807	split-GAL4 driver line targeting NBI005, WUI002, WUI003
Genetic reagent (Drosophila melanogaster)	10B11-x-VT012768	this paper	split_gal4.janelia.org:SS49809	split-GAL4 driver line targeting PSI
Genetic reagent (Drosophila melanogaster)	10B11-x-118F02	this paper	split_gal4.janelia.org:SS49810	split-GAL4 driver line targeting PSI
Genetic reagent (Drosophila melanogaster)	10B11-x-13C08	this paper	split_gal4.janelia.org:SS49812	split-GAL4 driver line targeting PSI
Genetic reagent (Drosophila melanogaster)	72B02-x-59H02	this paper	split_gal4.janelia.org:SS49853	split-GAL4 driver line targeting HBI008, HBI010
Genetic reagent (Drosophila melanogaster)	93E02-x-VT026839	this paper	split_gal4.janelia.org:SS49861	split-GAL4 driver line targeting leg interneuron
Genetic reagent (Drosophila melanogaster)	125G04-x-24E06	this paper	split_gal4.janelia.org:SS51508	split-GAL4 driver line targeting A1VUM1
Genetic reagent (Drosophila melanogaster)	45G01-x-121D11	this paper	split_gal4.janelia.org:SS51523	split-GAL4 driver line targeting hDVMN
Genetic reagent (Drosophila melanogaster)	45G01-x-122H10	this paper	split_gal4.janelia.org:SS51524	split-GAL4 driver line targeting hDVMN
Genetic reagent (Drosophila melanogaster)	73C04-x-60A06	this paper	split_gal4.janelia.org:SS51528	split-GAL4 driver line targeting tpN MN
Genetic reagent (Drosophila melanogaster)	74H07-x-117C08	this paper	split_gal4.janelia.org:SS51531	split-GAL4 driver line targeting HUI001, HUI003
Genetic reagent (Drosophila melanogaster)	VT019307-x-VT029814	this paper	split_gal4.janelia.org:SS51830	split-GAL4 driver line targeting WBL024, WBI016
Genetic reagent (Drosophila melanogaster)	120H05-x-76H04	this paper	split_gal4.janelia.org:SS52389	split-GAL4 driver line targeting WBL026, WBL027, WBL028, WBL029, WBL030
Genetic reagent (Drosophila melanogaster)	124C12-x-102C03	this paper	split_gal4.janelia.org:SS52392	split-GAL4 driver line targeting C2BI001, C2BI003, C2BI006, C2BI007, C2BI010
Genetic reagent (Drosophila melanogaster)	125A04-x-102C03	this paper	split_gal4.janelia.org:SS52395	split-GAL4 driver line targeting hg2 MN
Genetic reagent (Drosophila melanogaster)	127F10-x-102B02	this paper	split_gal4.janelia.org:SS52404	split-GAL4 driver line targeting hg1 MN
Genetic reagent (Drosophila melanogaster)	127F10-x-125C10	this paper	split_gal4.janelia.org:SS52405	split-GAL4 driver line targeting b3 MN, i2 MN, hg3 MN
Genetic reagent (Drosophila melanogaster)	112B01-x-128A07	this paper	split_gal4.janelia.org:SS53378	split-GAL4 driver line targeting hDVMN
Genetic reagent (Drosophila melanogaster)	22E12-x-58F02	this paper	split_gal4.janelia.org:SS53421	split-GAL4 driver line targeting WUL018, WUI006, WUI007, WUI008, WUI009
Genetic reagent (Drosophila melanogaster)	69H11-x-85G10	this paper	split_gal4.janelia.org:SS53435	split-GAL4 driver line targeting leg interneuron
Genetic reagent (Drosophila melanogaster)	75C10-x-107G12	this paper	split_gal4.janelia.org:SS53438	split-GAL4 driver line targeting i2 MN
Genetic reagent (Drosophila melanogaster)	85G10-x-117H12	this paper	split_gal4.janelia.org:SS54445	split-GAL4 driver line targeting WBL013
Genetic reagent (Drosophila melanogaster)	125H05-x-46C02	this paper	split_gal4.janelia.org:SS54474	split-GAL4 driver line targeting NBI004, NBI006, NBI007
Genetic reagent (Drosophila melanogaster)	125H05-x-93B07	this paper	split_gal4.janelia.org:SS54480	split-GAL4 driver line targeting NUI001, NBI003, NBI004, NBI007
Genetic reagent (Drosophila melanogaster)	46C02-x-125D05	this paper	split_gal4.janelia.org:SS54495	split-GAL4 driver line targeting NUI001, NBI006, NBI007, NBI008, NBI009
Genetic reagent (Drosophila melanogaster)	50G08-x-59D07	this paper	split_gal4.janelia.org:SS54506	split-GAL4 driver line targeting HUL001, HUL002, HUL003, HUL004, HUL005, HUL007, HUL008, HUL009
Genetic reagent (Drosophila melanogaster)	VT026771-x-VT019749	this paper	split_gal4.janelia.org:SS60603	split-GAL4 driver line targeting NBI001, XBI001, C2BI005
Antibody	nc82 supernatent	mouse α-bruchpilot	Developmental Studies Hybridoma Bank # nc	https://www.janelia.org/sites/default/files/Project%20Teams/Fly%20Light/FL%20Protocol%20-%20Adult%20IHC%20%20Split%20Screen_1.pdf
Antibody	rabbit polyclonal anti-GFP	Thermo Fischer Scientific	Cat #: A-11122; RRID: AB_221569	
Antibody	Alexa Fluor 488 goat anti-rabbit	Thermo Fischer Scientific	Cat #: A-11034; RRID: AB_2576217	
Antibody	Alexa Fluor 568 goat anti-mouse	Thermo Fischer Scientific	Cat #: A-11031; RRID: AB_144696	
Chemical compound, drug	Phalloidin Alexa Fluor 633	Life Tech		
Chemical compound, drug	paraformaldehyde	Electron Miscroscopy Services	15713-S	https://www.janelia.org/sites/default/files/Project%20Teams/Fly%20Light/FL%20Recipe%20-%20PFA_2.pdf
Chemical compound, drug	Triton X-100	Sigma Aldrich	X100	https://www.janelia.org/sites/default/files/Project%20Teams/Fly%20Light/FL%20Protocol%20-%20Adult%20IHC%20%20Split%20Screen_1.pdf
				
Chemical compound, drug	SeaKem LE agarose	Fisher	Cat #: BMA50001	
Chemical compound, drug	DMSO			
Chemical compound, drug	Normal goat serum			
Chemical compound, drug	NaN3			
Chemical compound, drug	escin			
Chemical compound, drug	bovine hyaluronidase type IV-S	Sigma Aldrich	Cat #: H3884	
Chemical compound, drug	glycerol			
Chemical compound, drug	ethanol			
Chemical compound, drug	methyl salicylate	Sigma Aldrich	Cat #: M6752	
Chemical compound, drug	DPX Mountant	Electron Miscroscopy Services	#13512	https://www.janelia.org/sites/default/files/Project%20Teams/Fly%20Light/FL%20Protocol%20-%20DPX%20Mounting_0.pdf
Chemical compound, drug	xylene	Fisher	x5-500	https://www.janelia.org/sites/default/files/Project%20Teams/Fly%20Light/FL%20Protocol%20-%20DPX%20Mounting_0.pdf
Software, algorithm	FlySongSegmenter	Arthur et al, 2013		https://github.com/FlyCourtship/FlySongSegmenter
Software, algorithm	BatchSongAnalysis	Ben Arthur, David Stern		https://github.com/dstern/BatchSongAnalysis
Software, algorithm	Kinefly	Suver at al, 2016		https://github.com/ssafarik/Kinefly

## Data Availability

All code needed to reproduce the analyses in this paper can be found at: https://github.com/samcwhitehead/dorsal_vnc_analysis
