# Supplementary figures and images for: Single-cell type analysis of wing premotor circuits in the ventral nerve cord of Drosophila melanogaster
Source: bioRxiv. 2025 Feb 20:2023.05.31.542897. Preprint. [Version 3] doi: 10.1101/2023.05.31.542897 (PMC10312520; doi:10.1101/2023.05.31.542897)

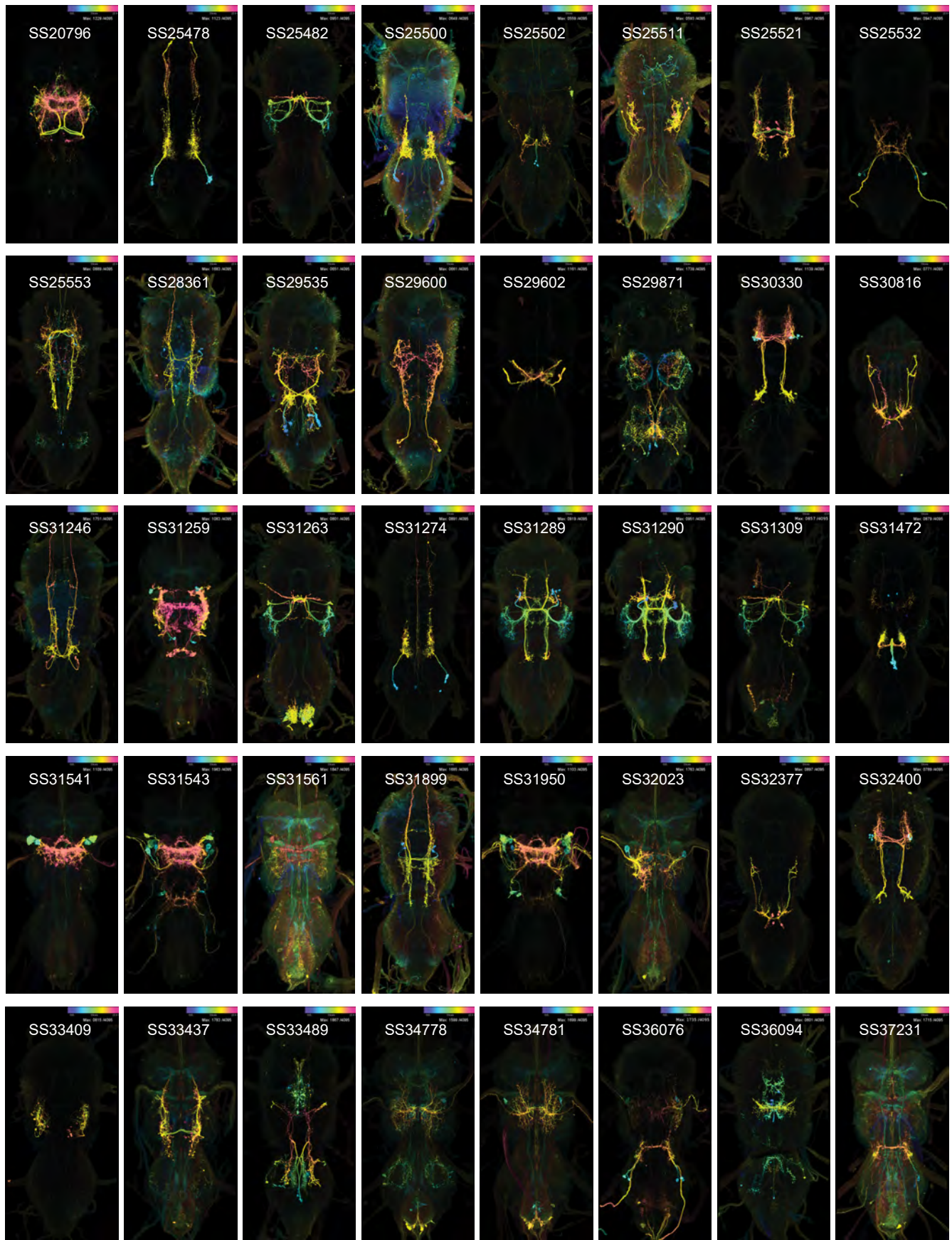

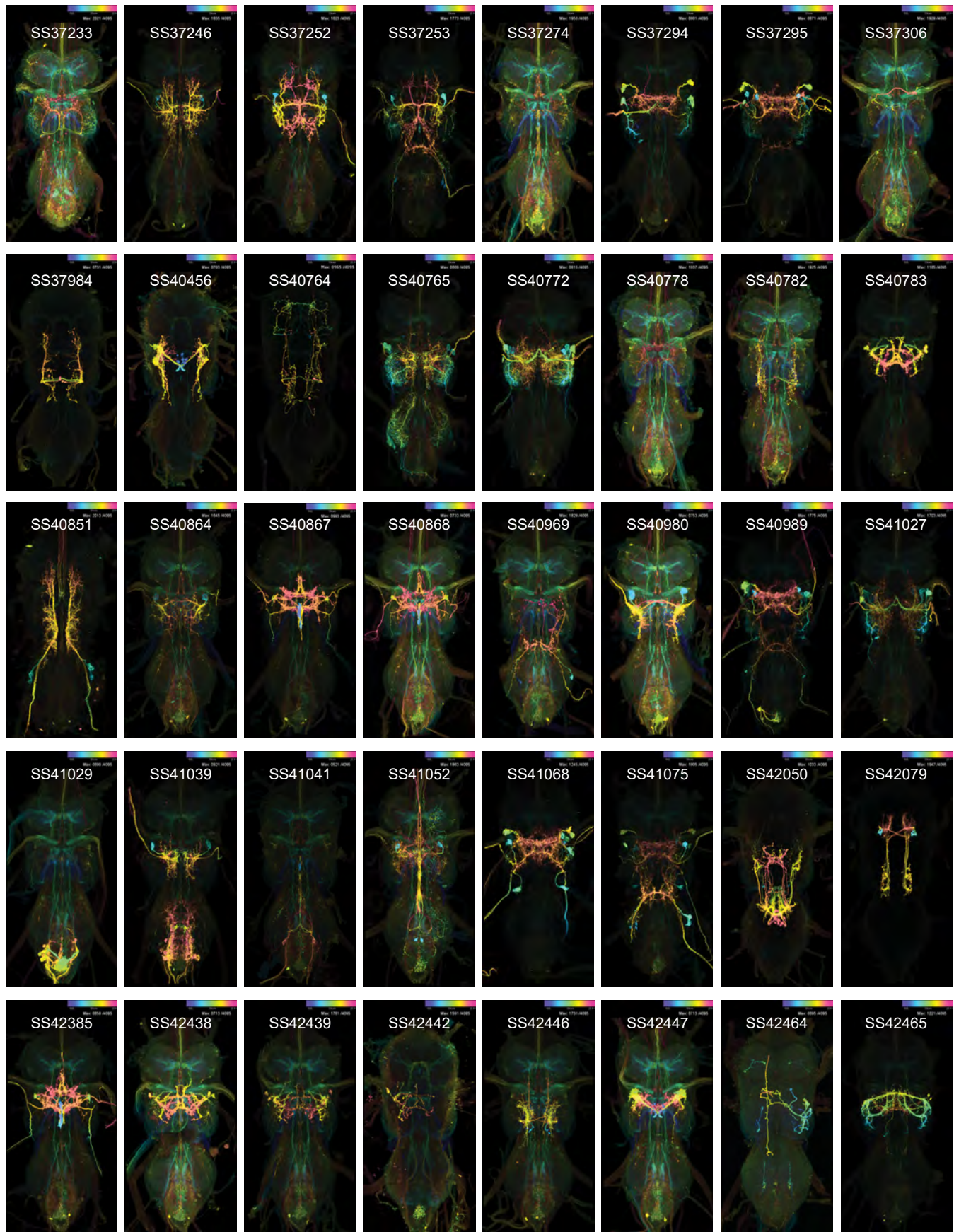

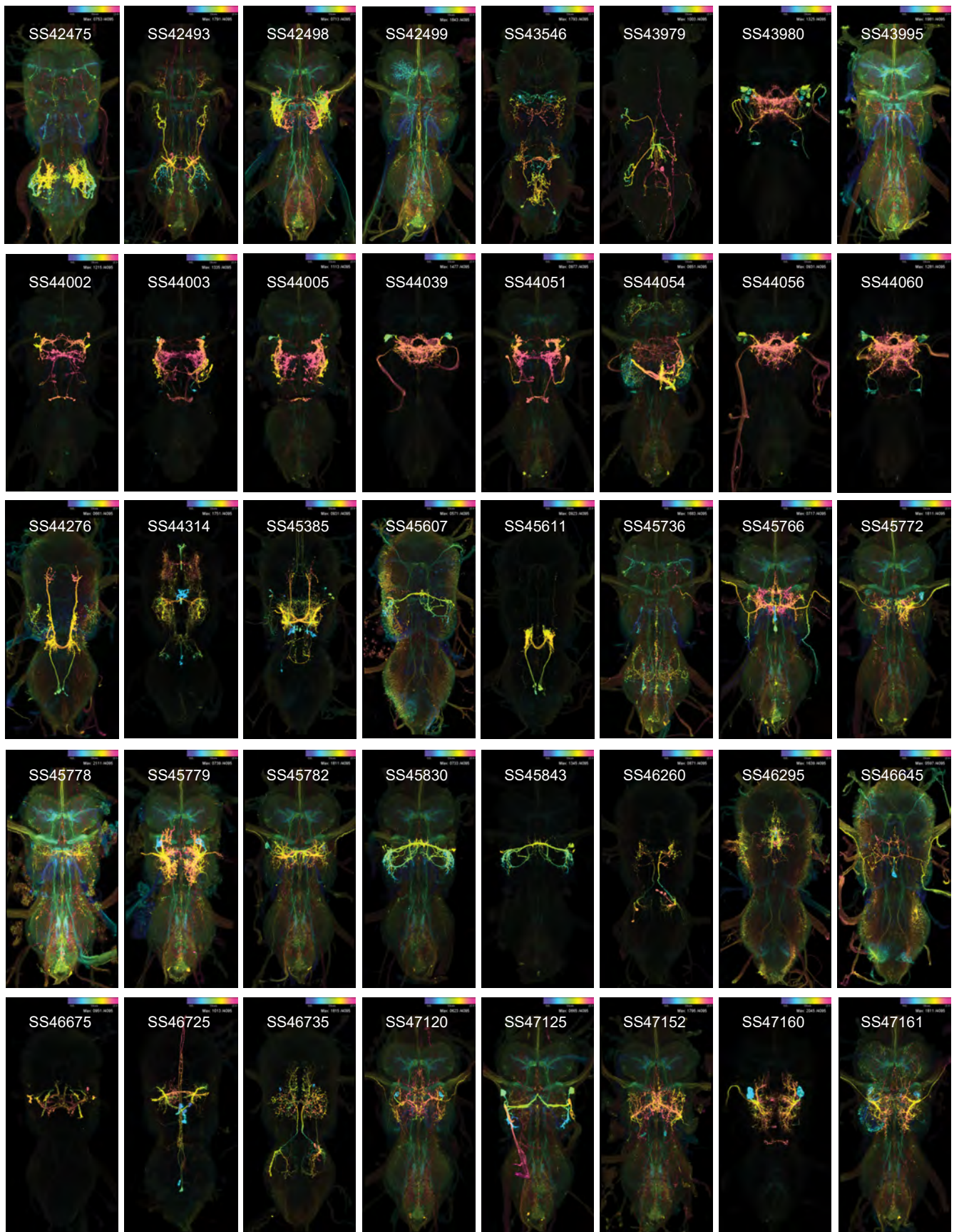

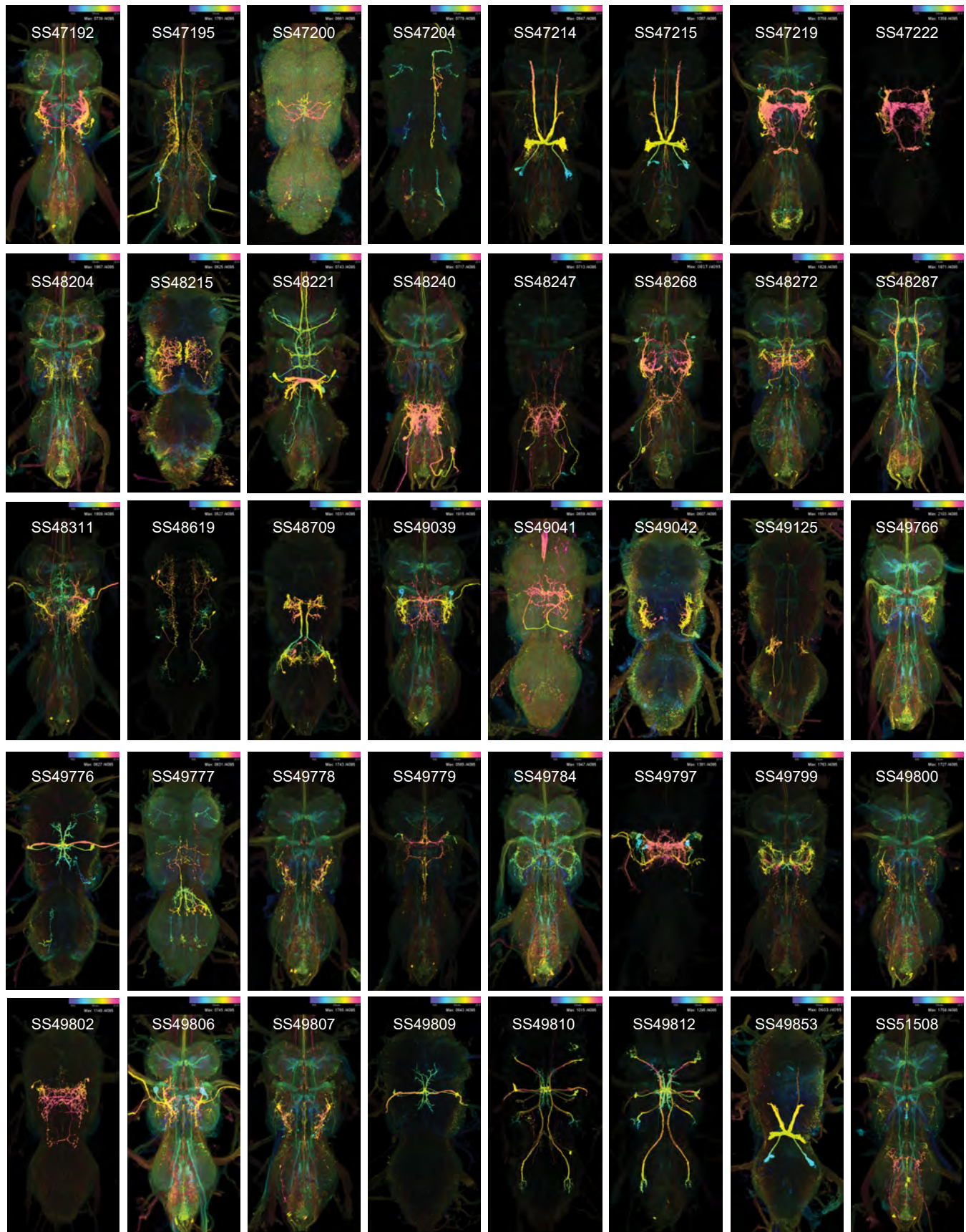

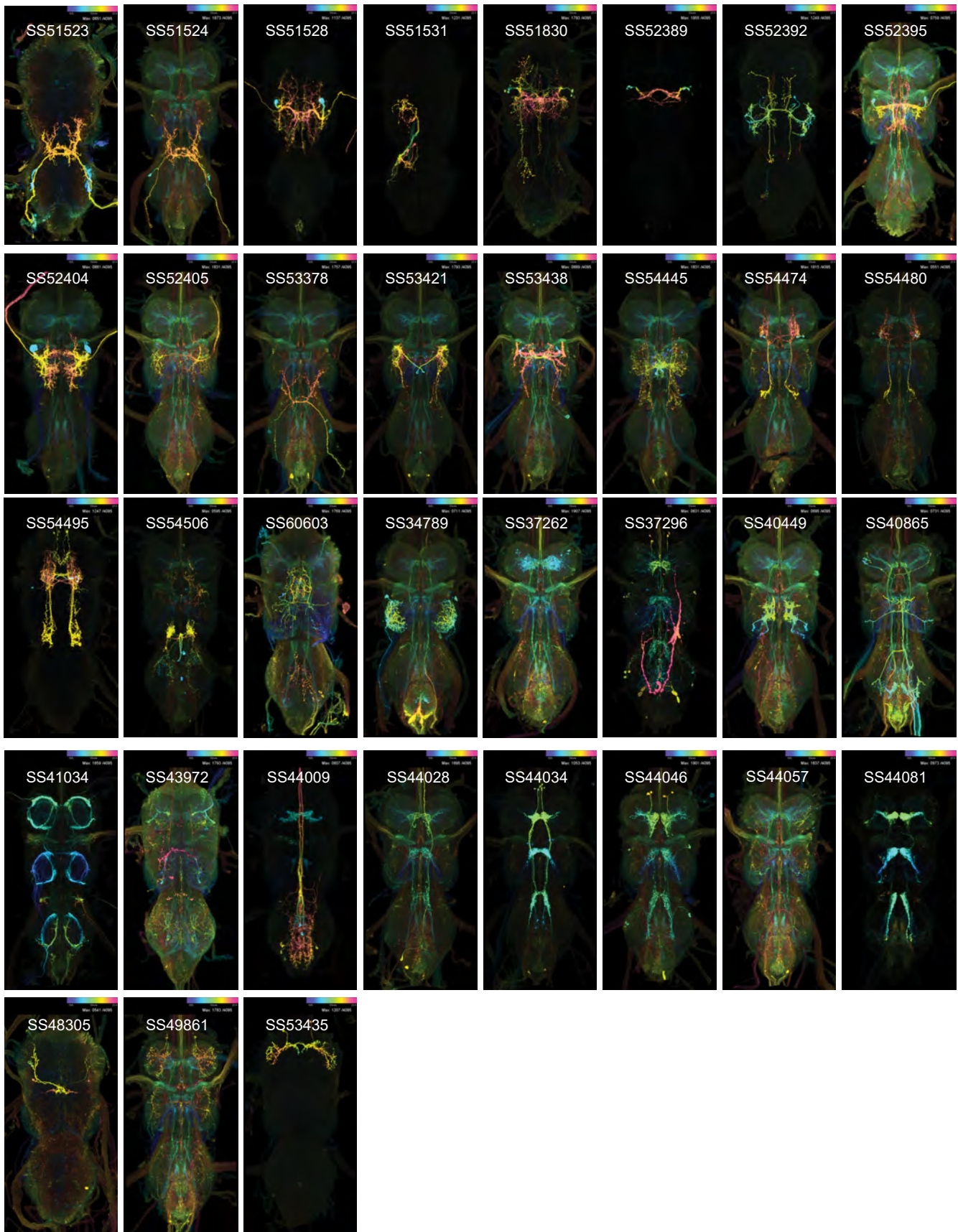

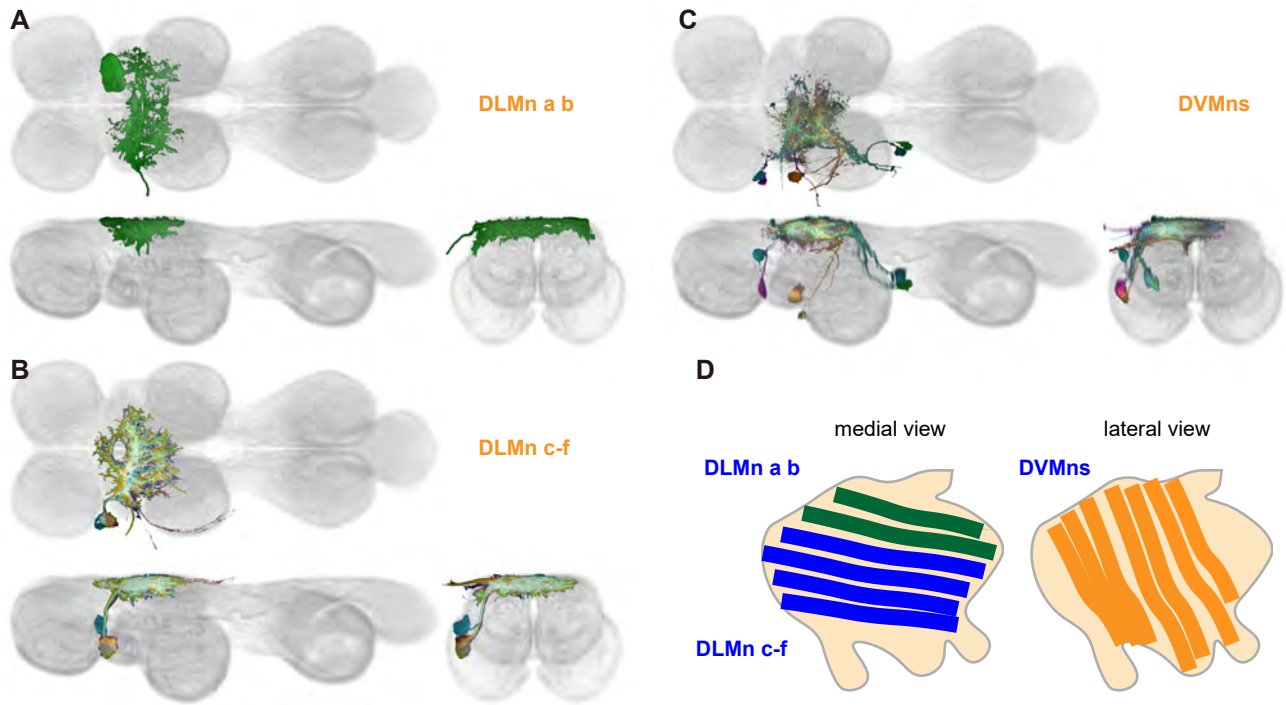

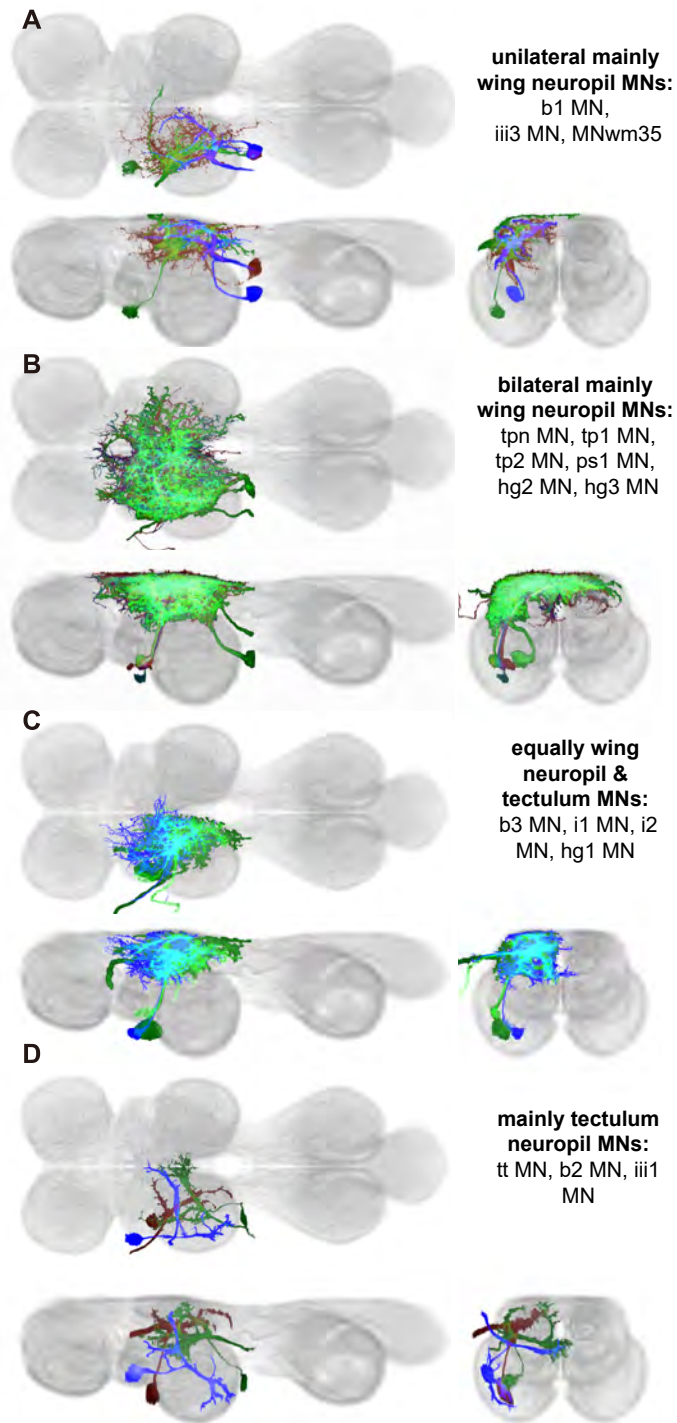

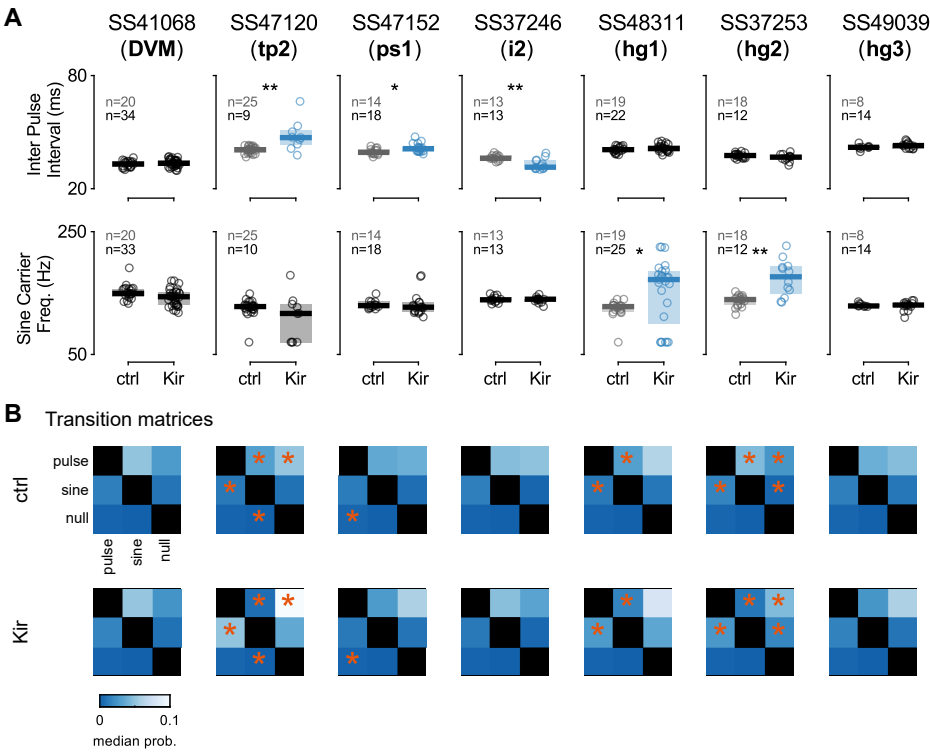

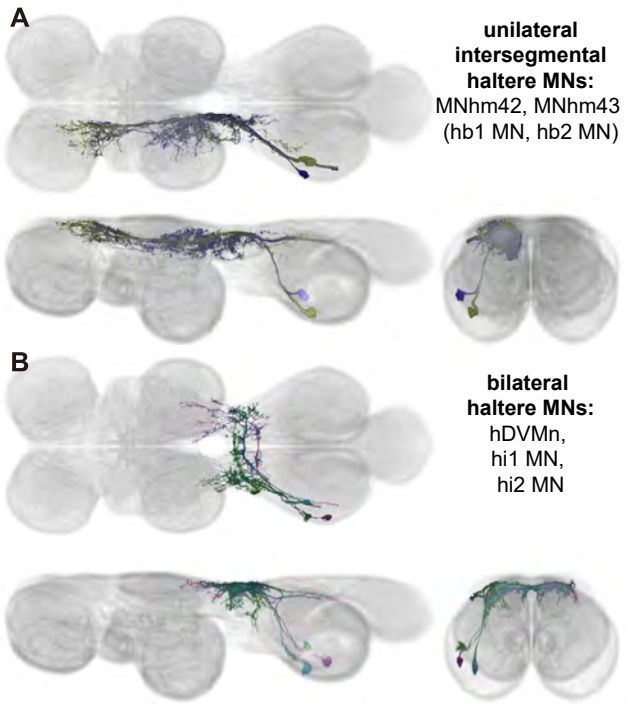

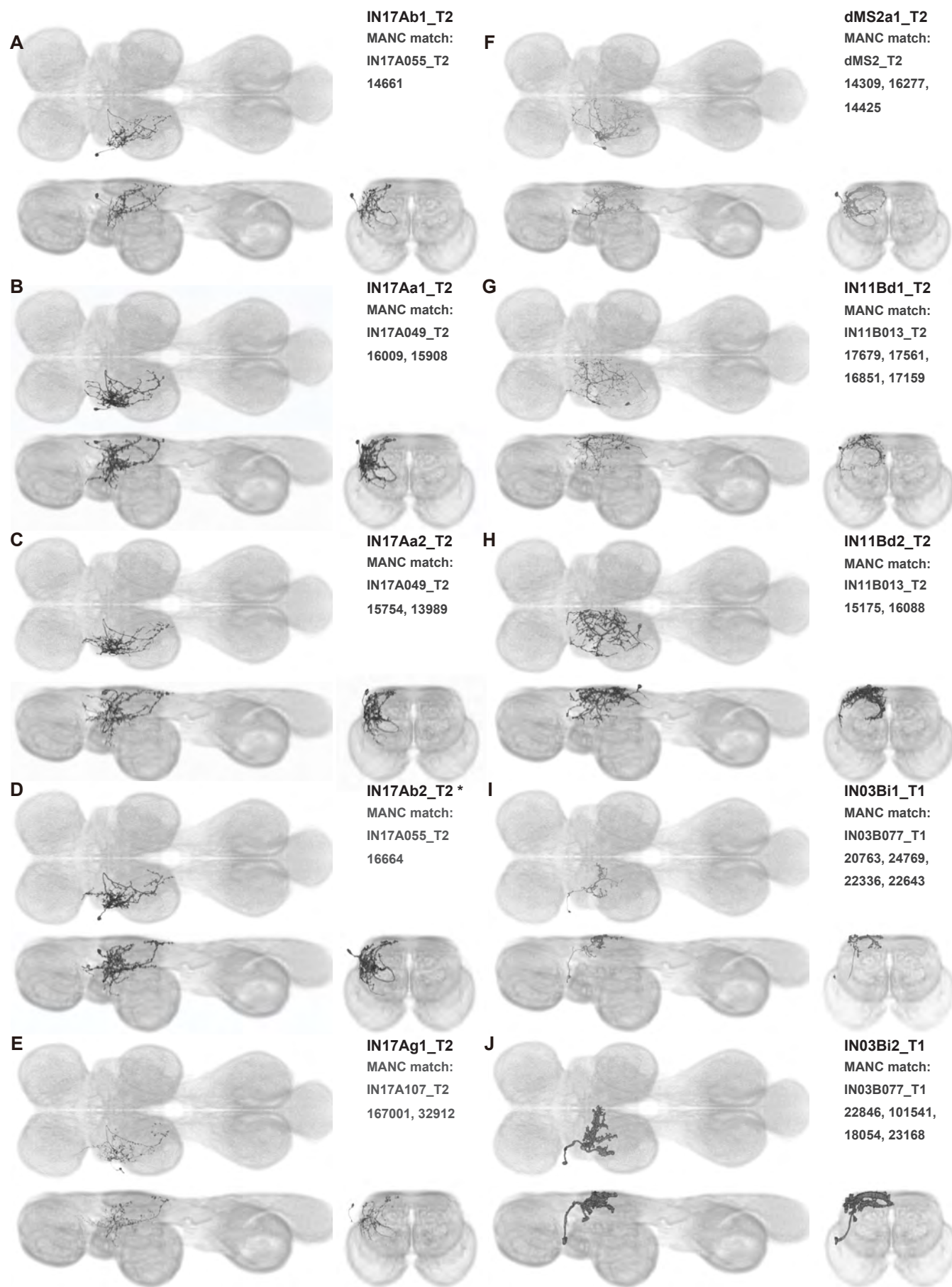

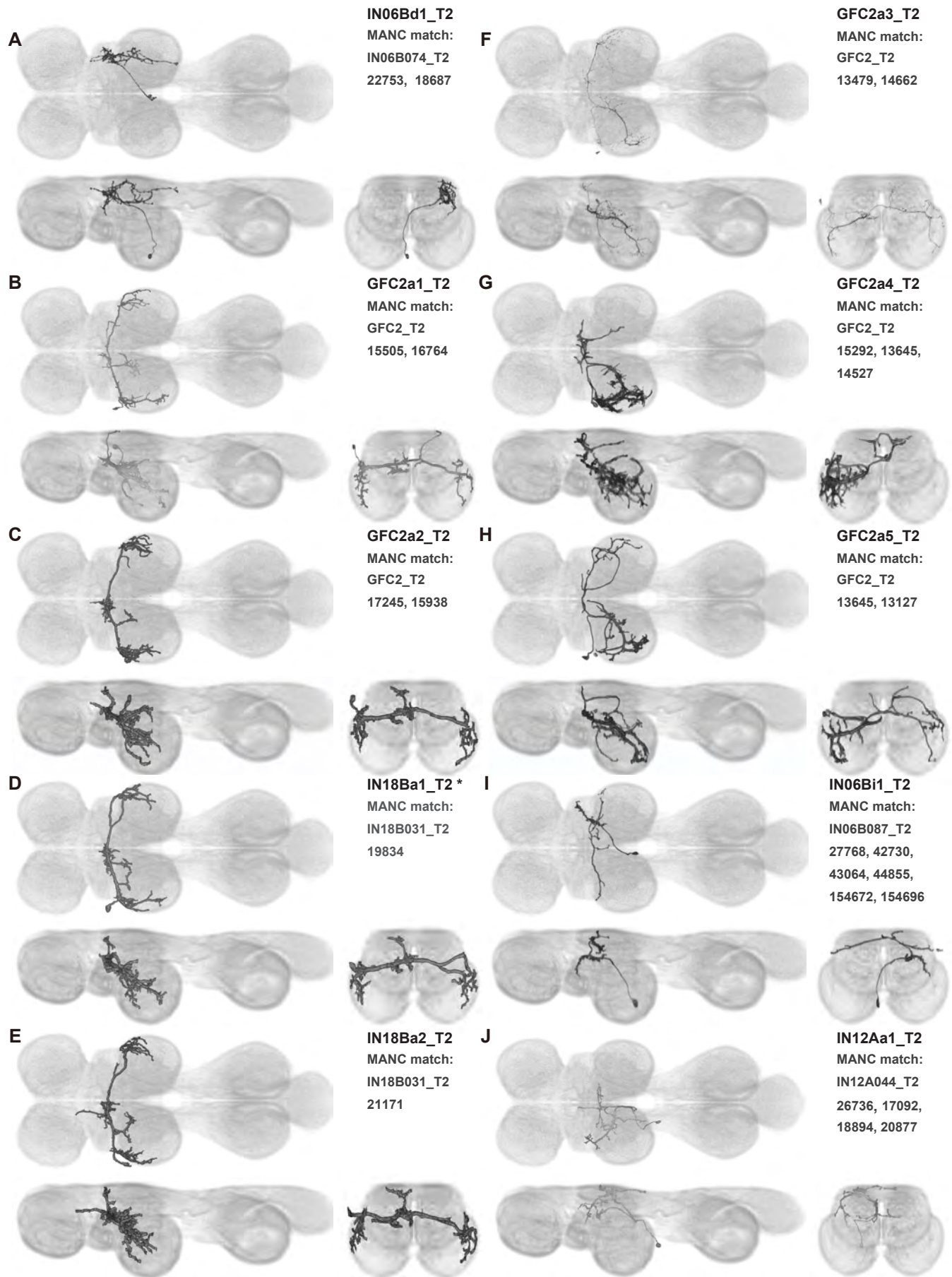

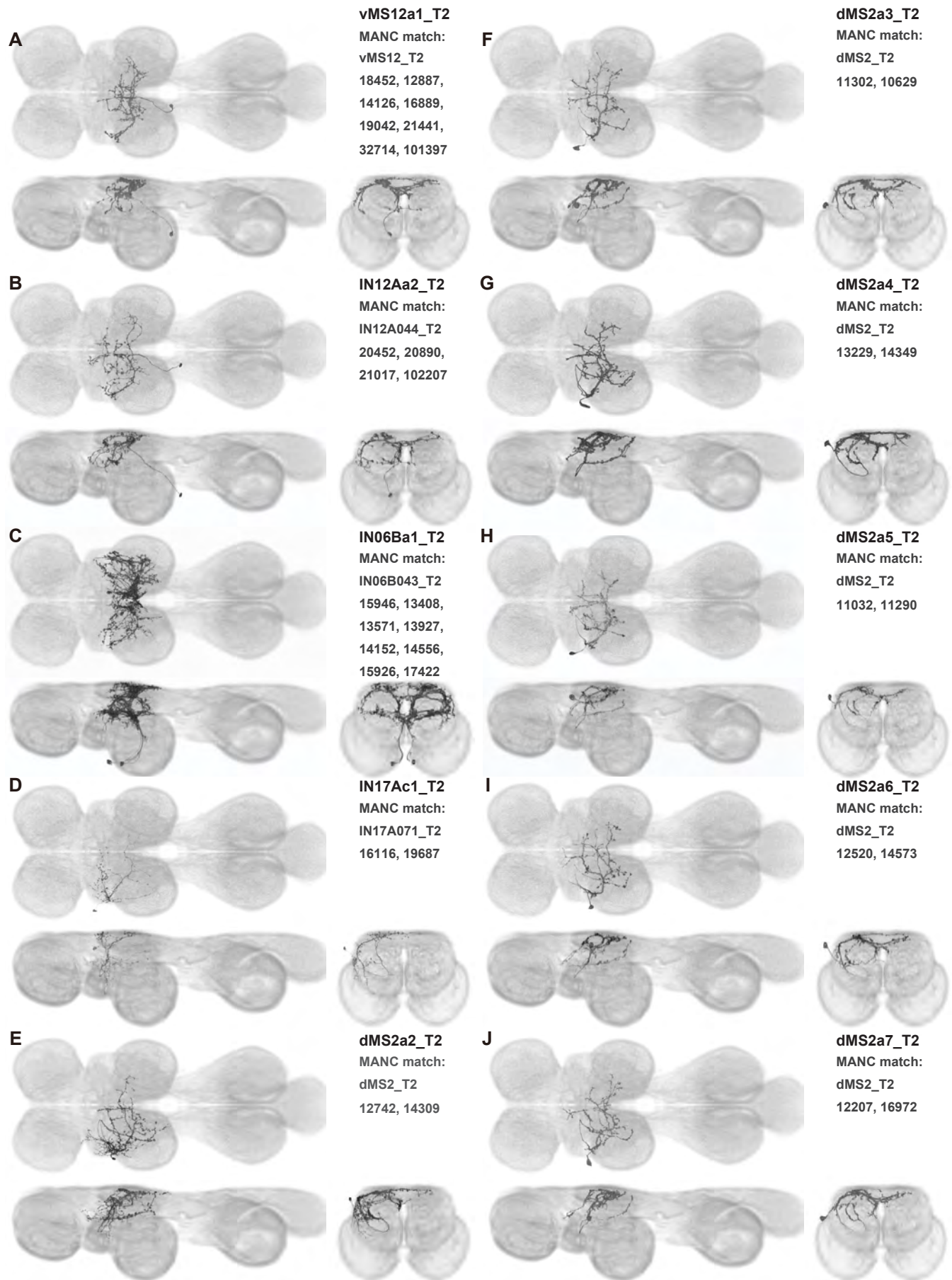

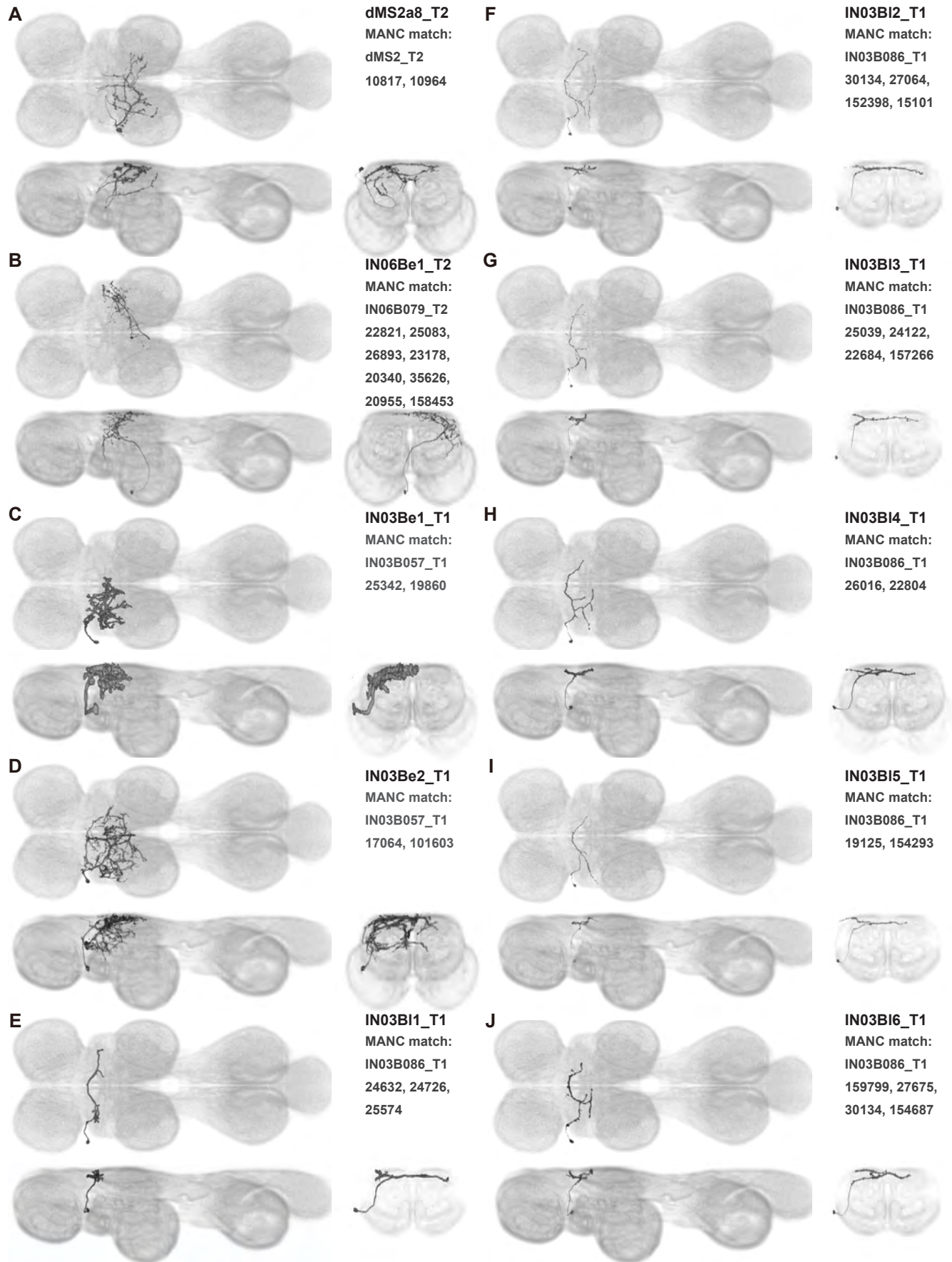

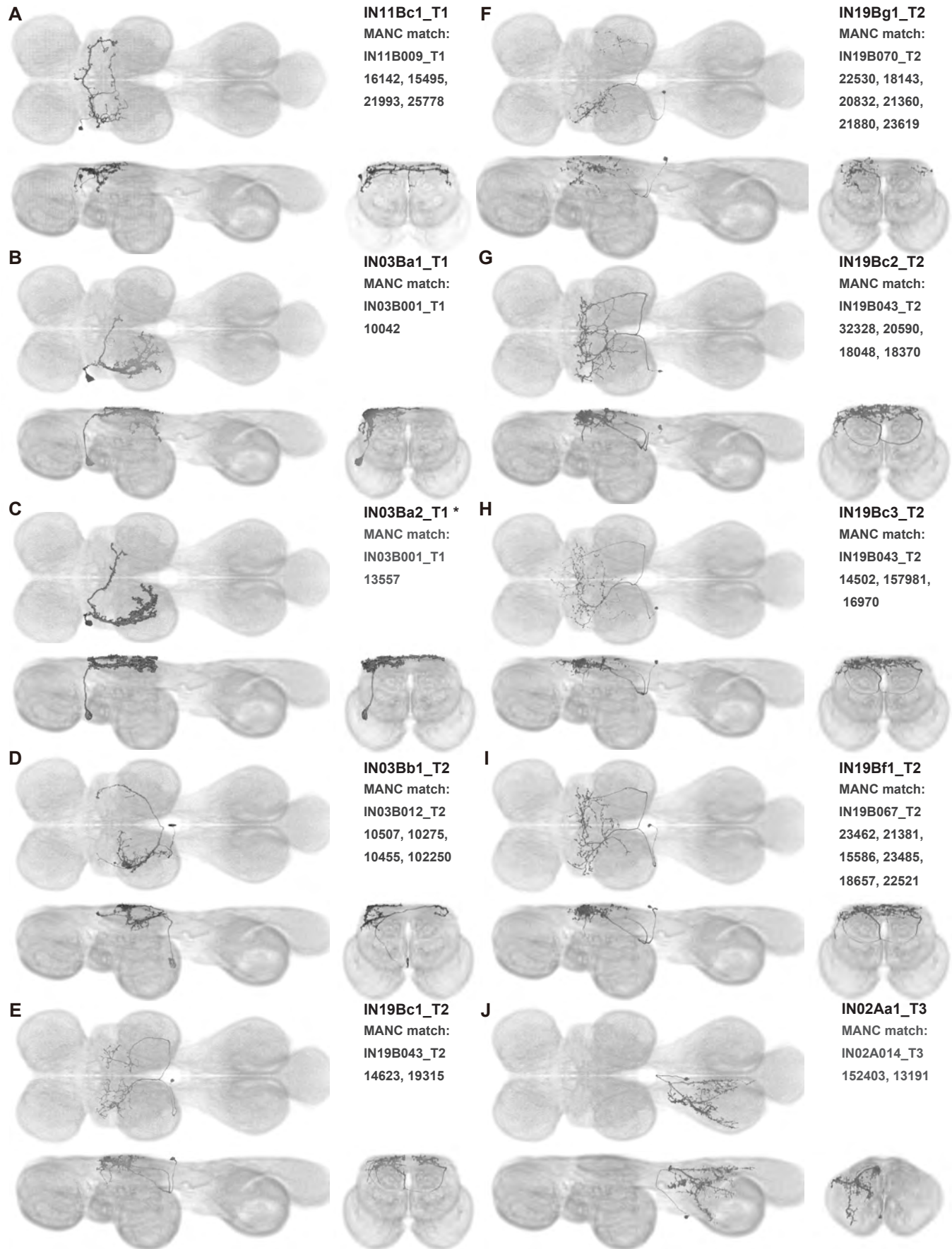

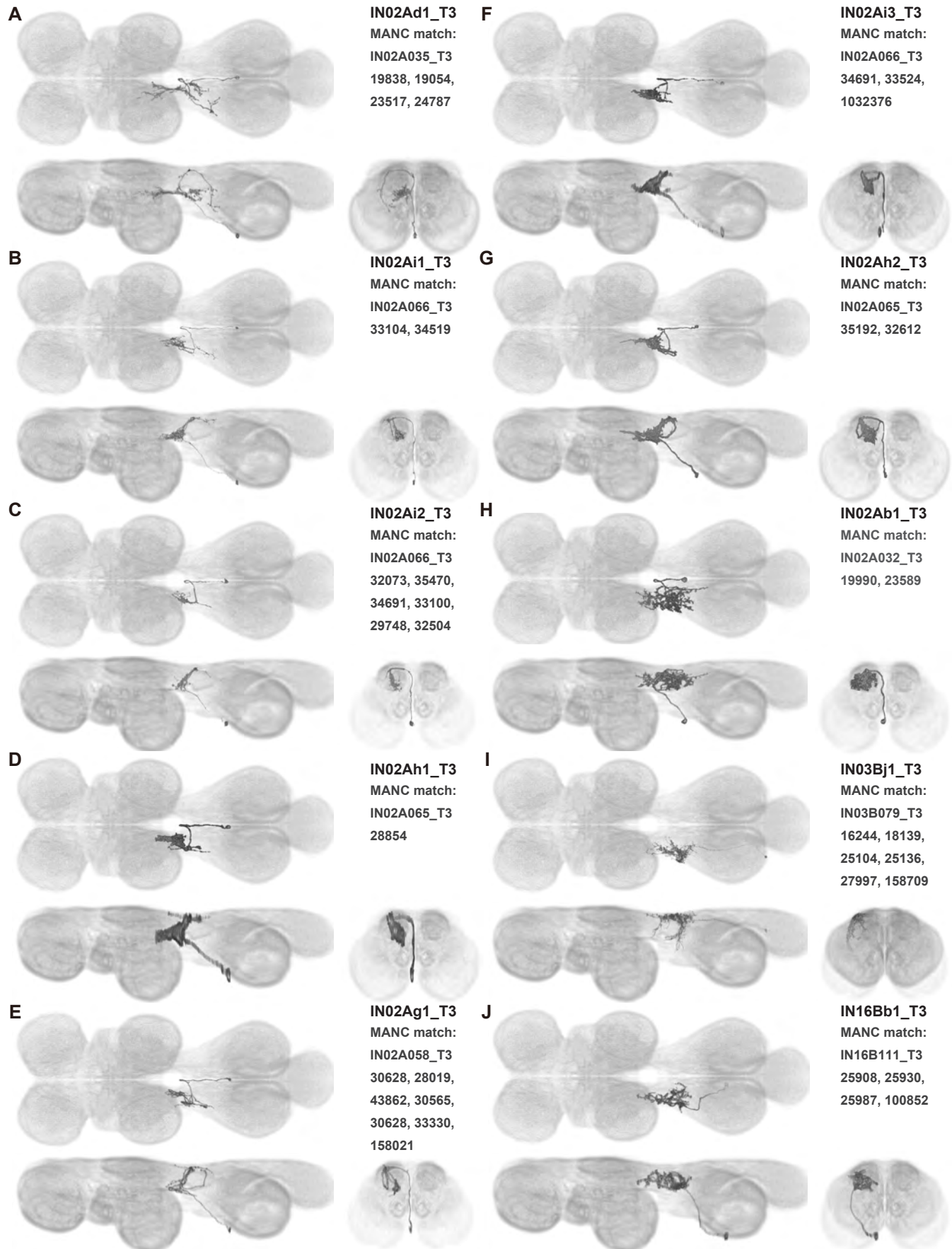

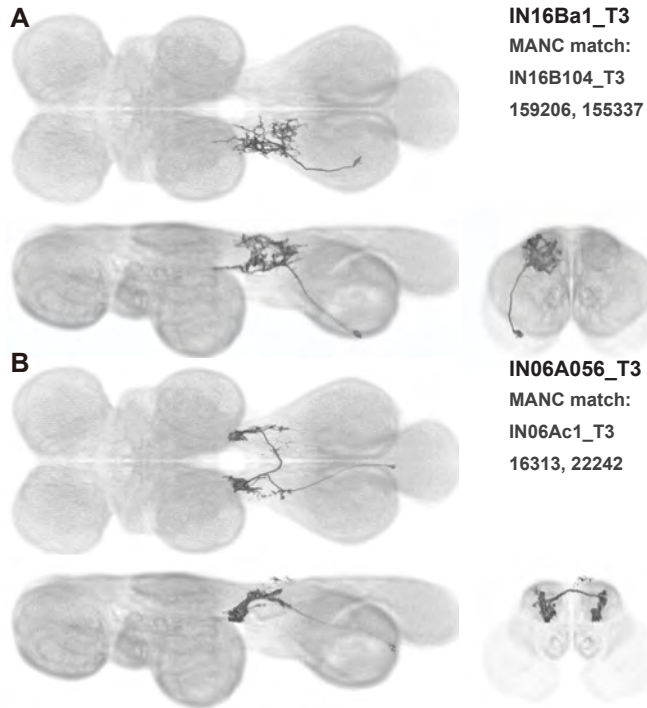

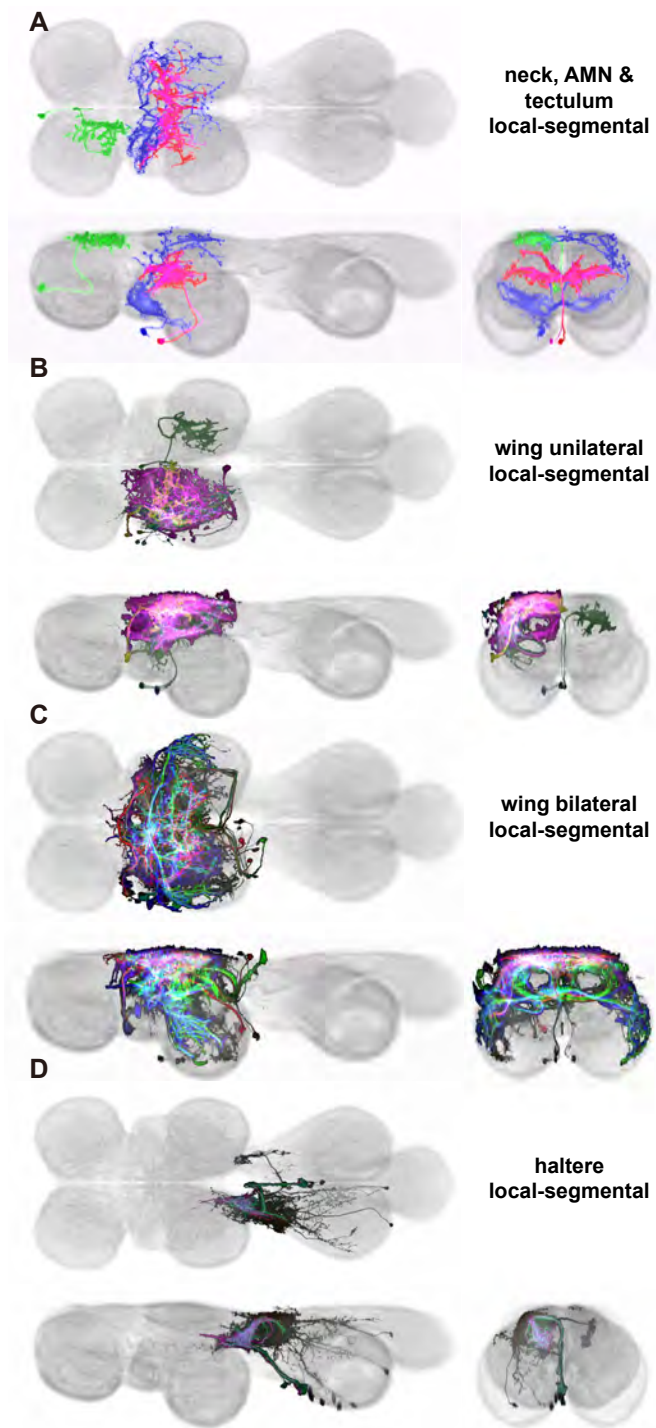

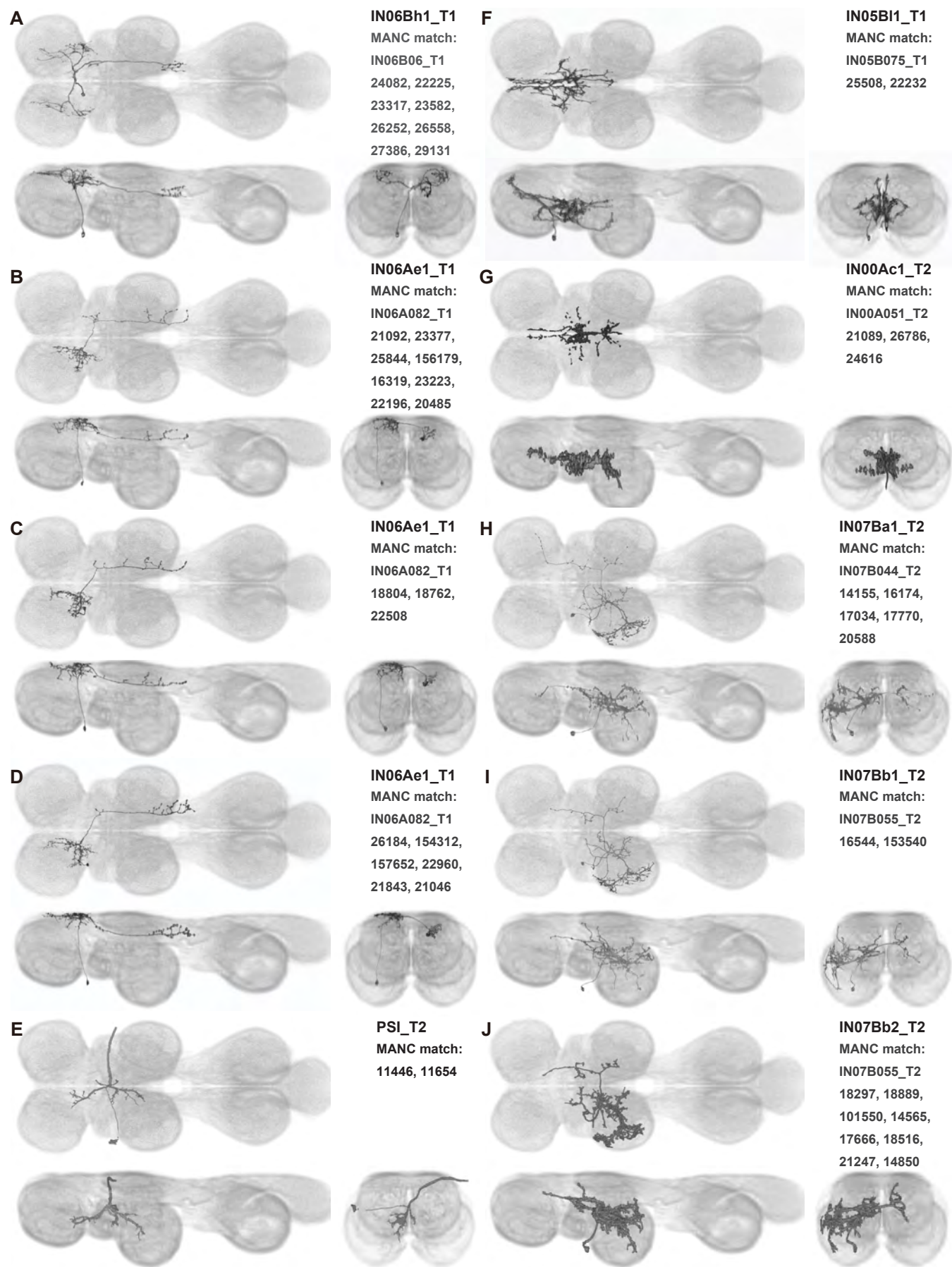

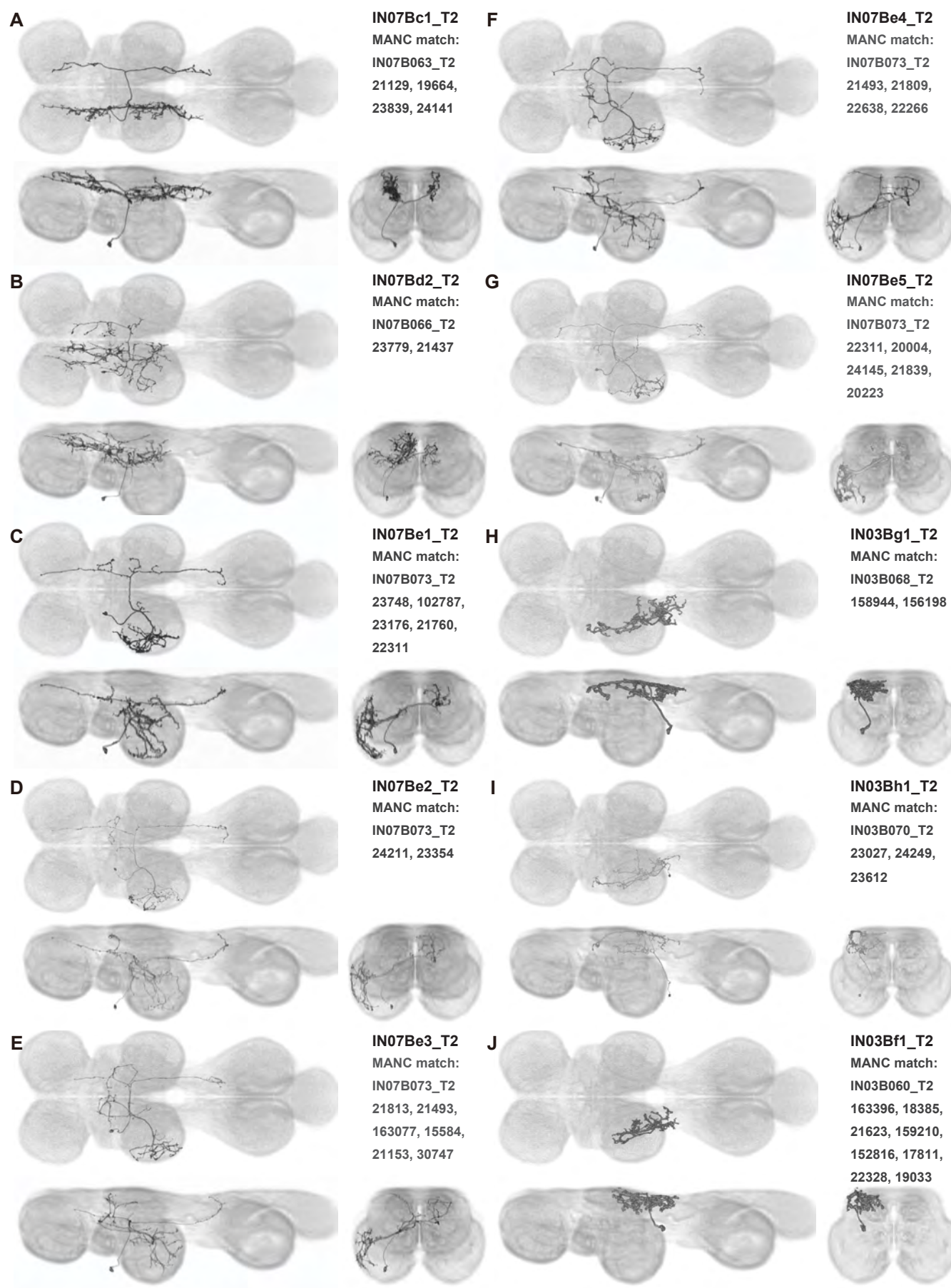

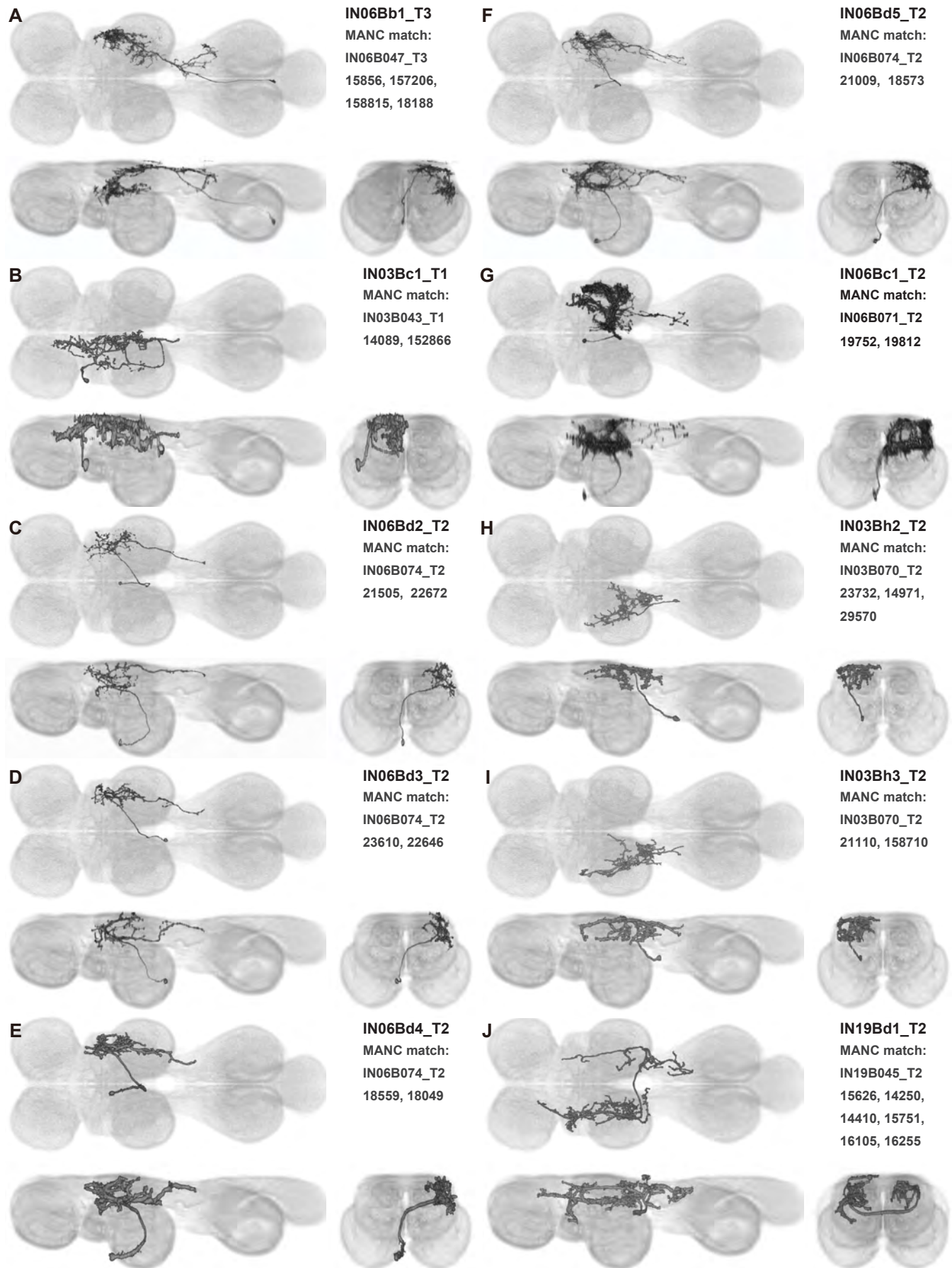

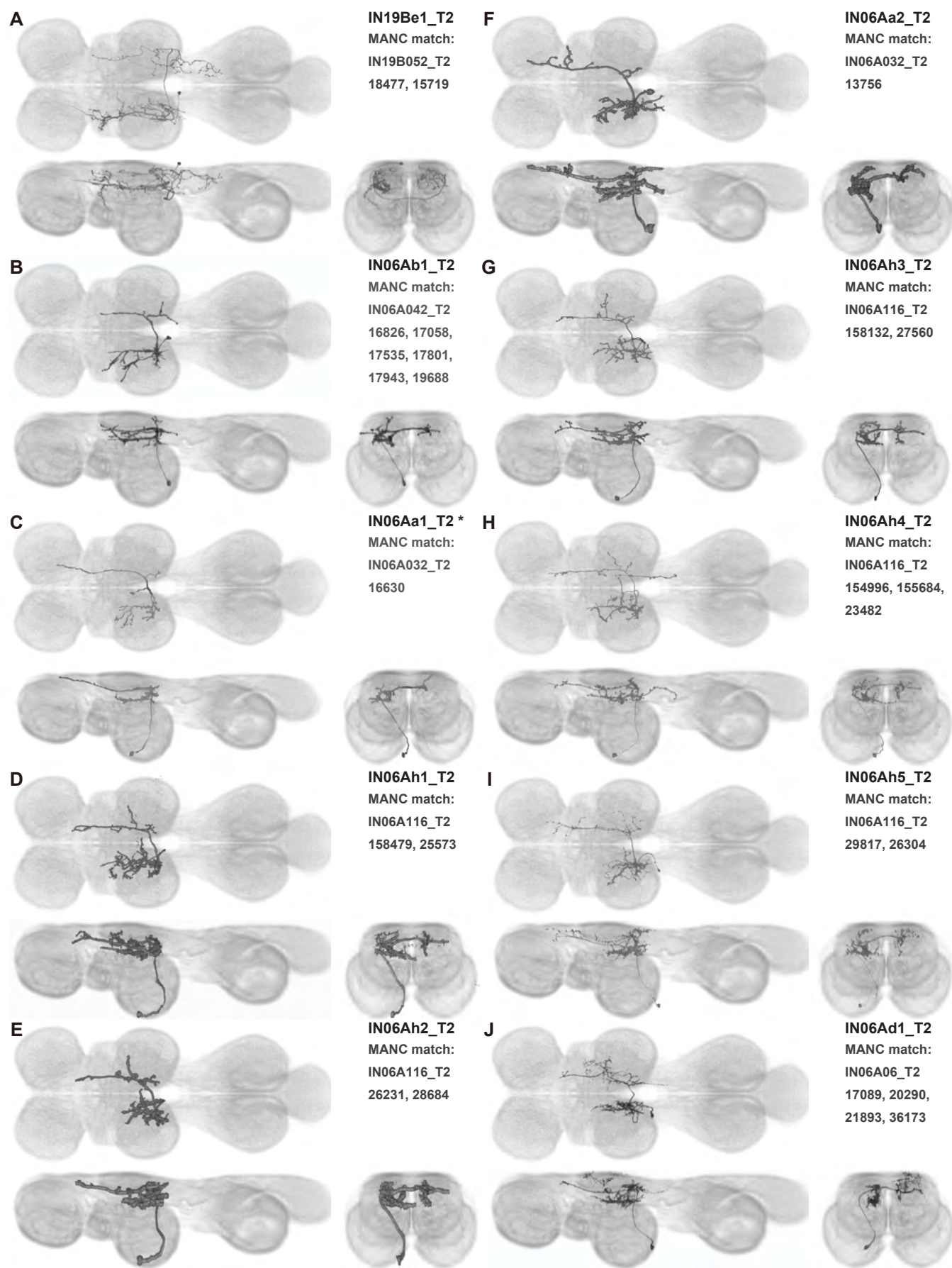

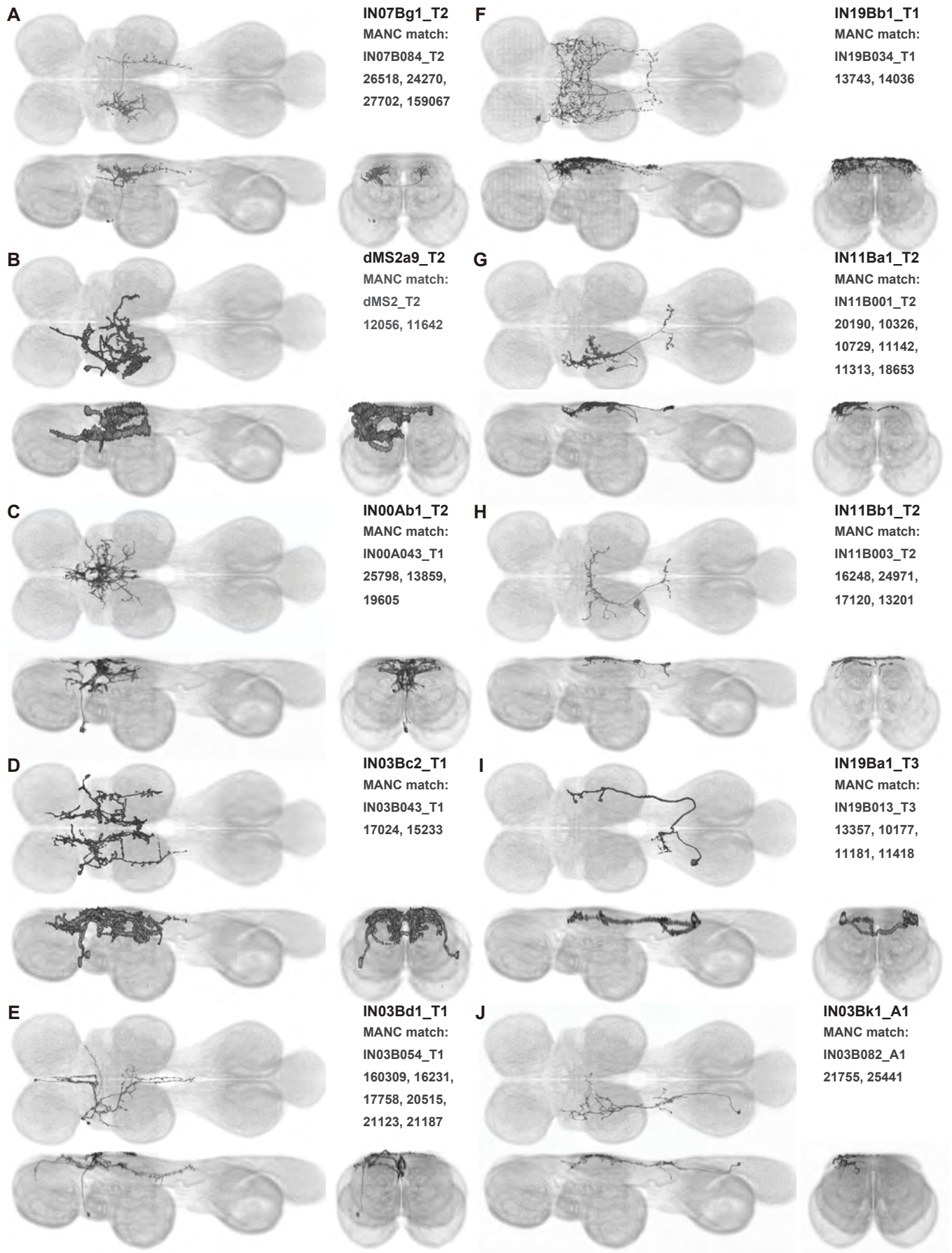

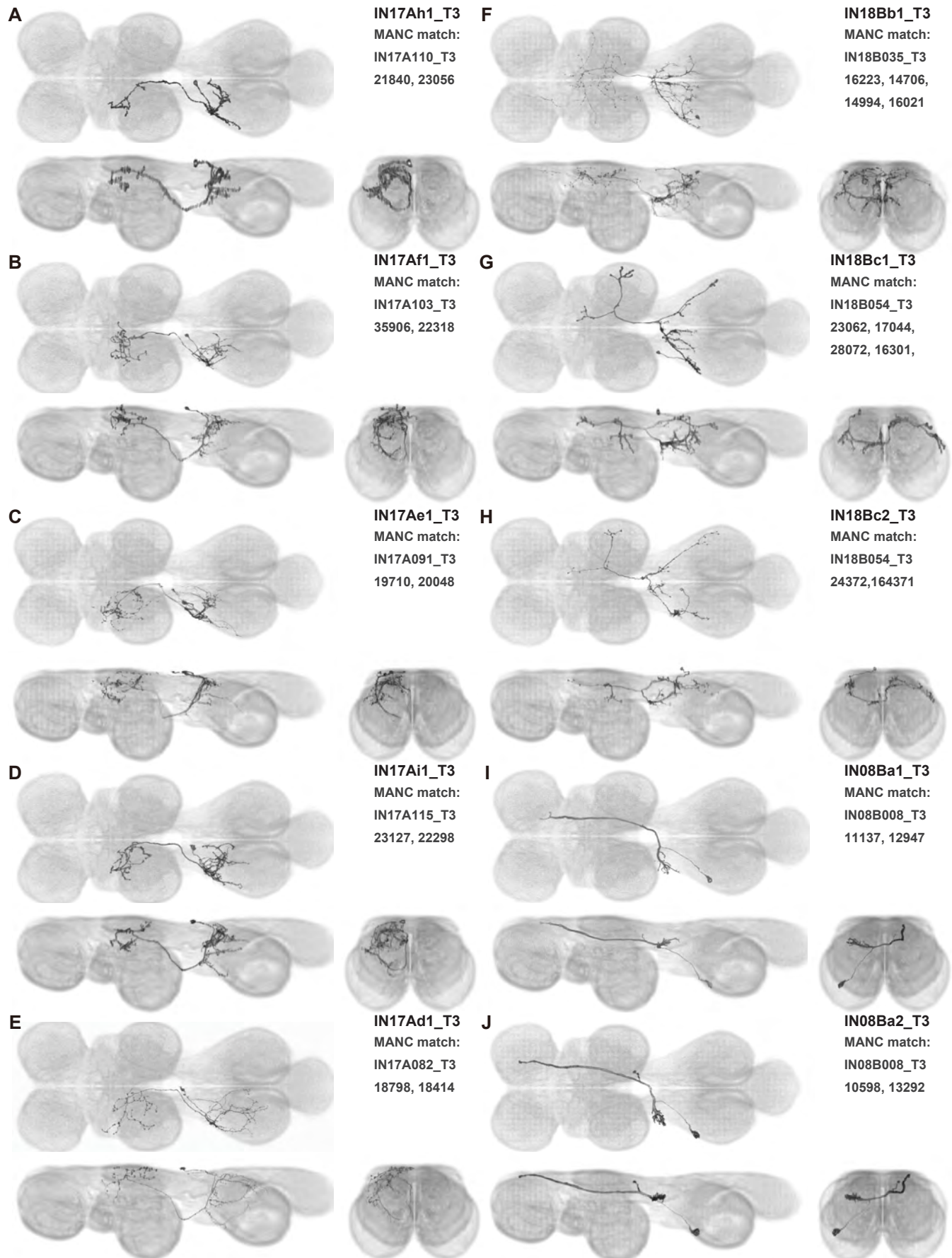

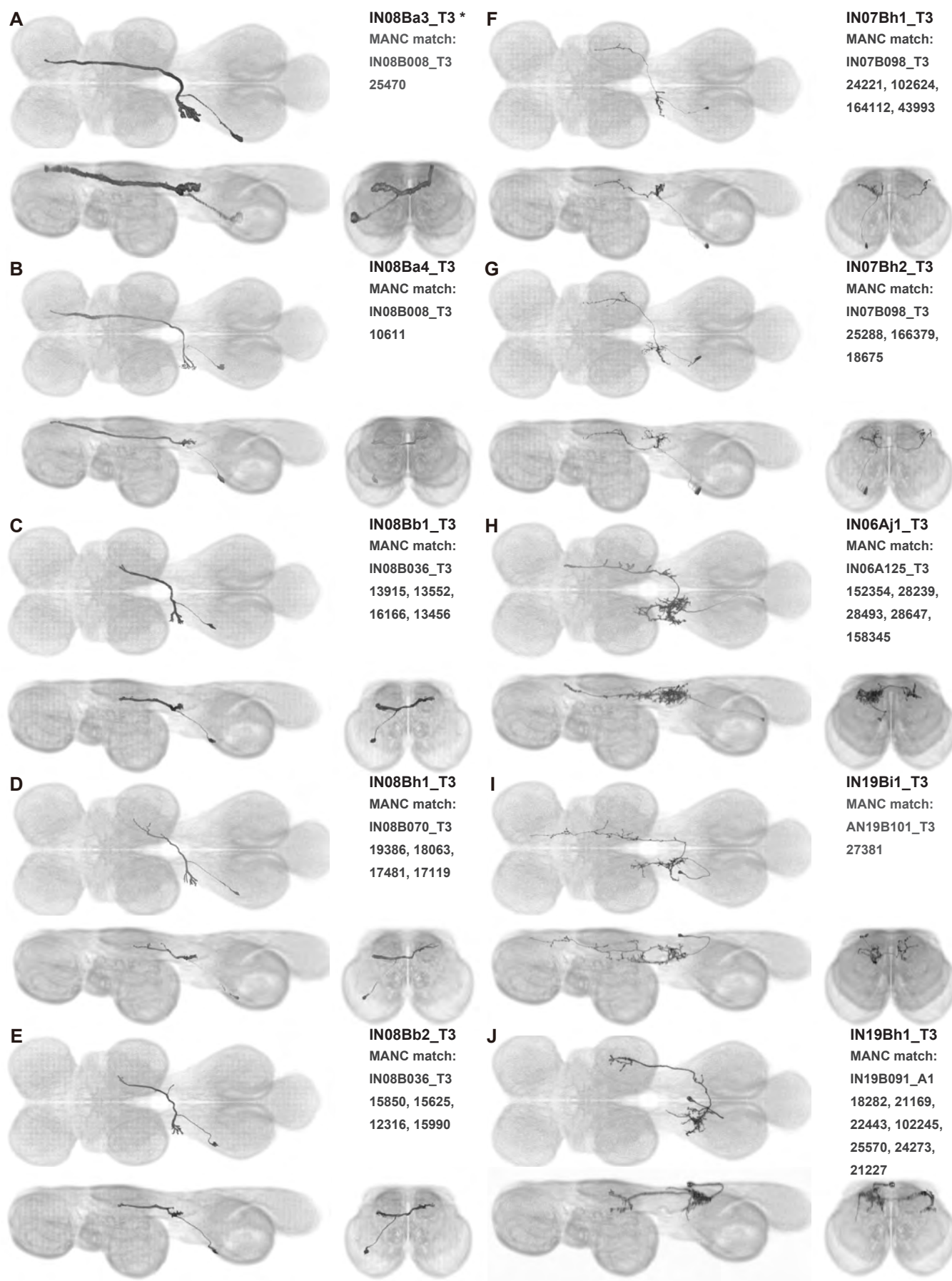

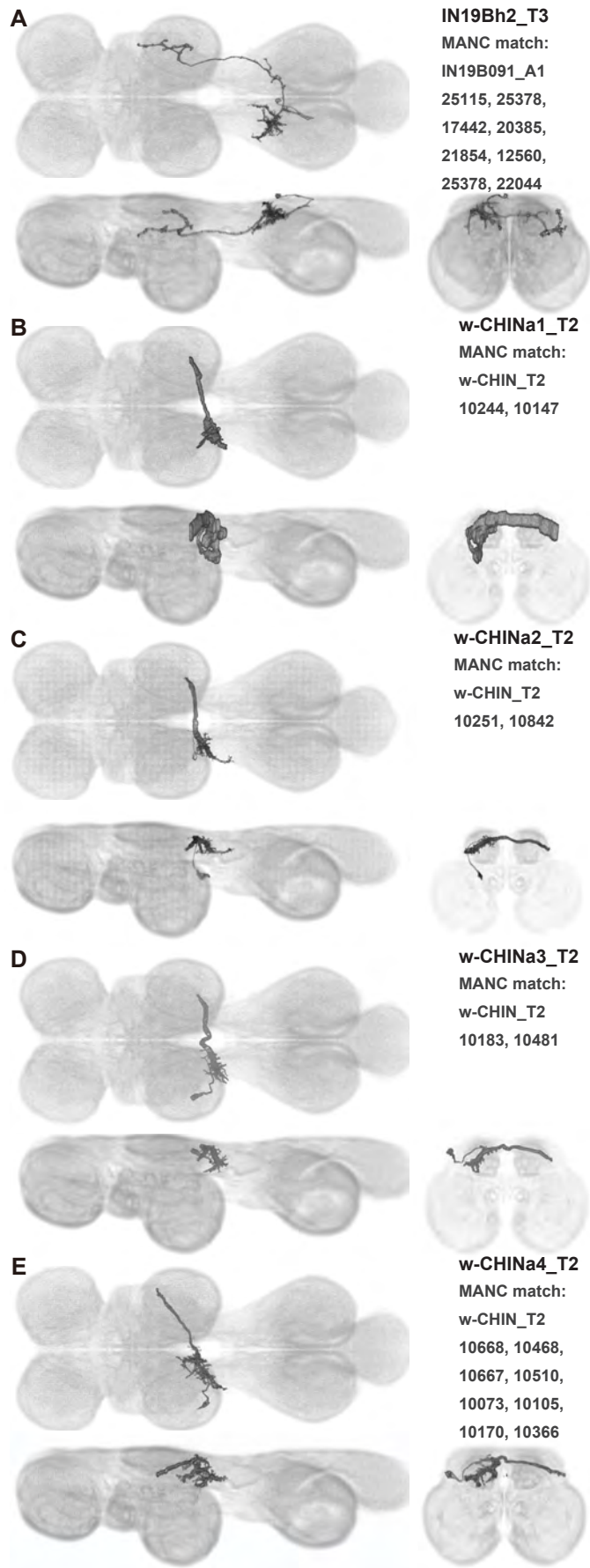

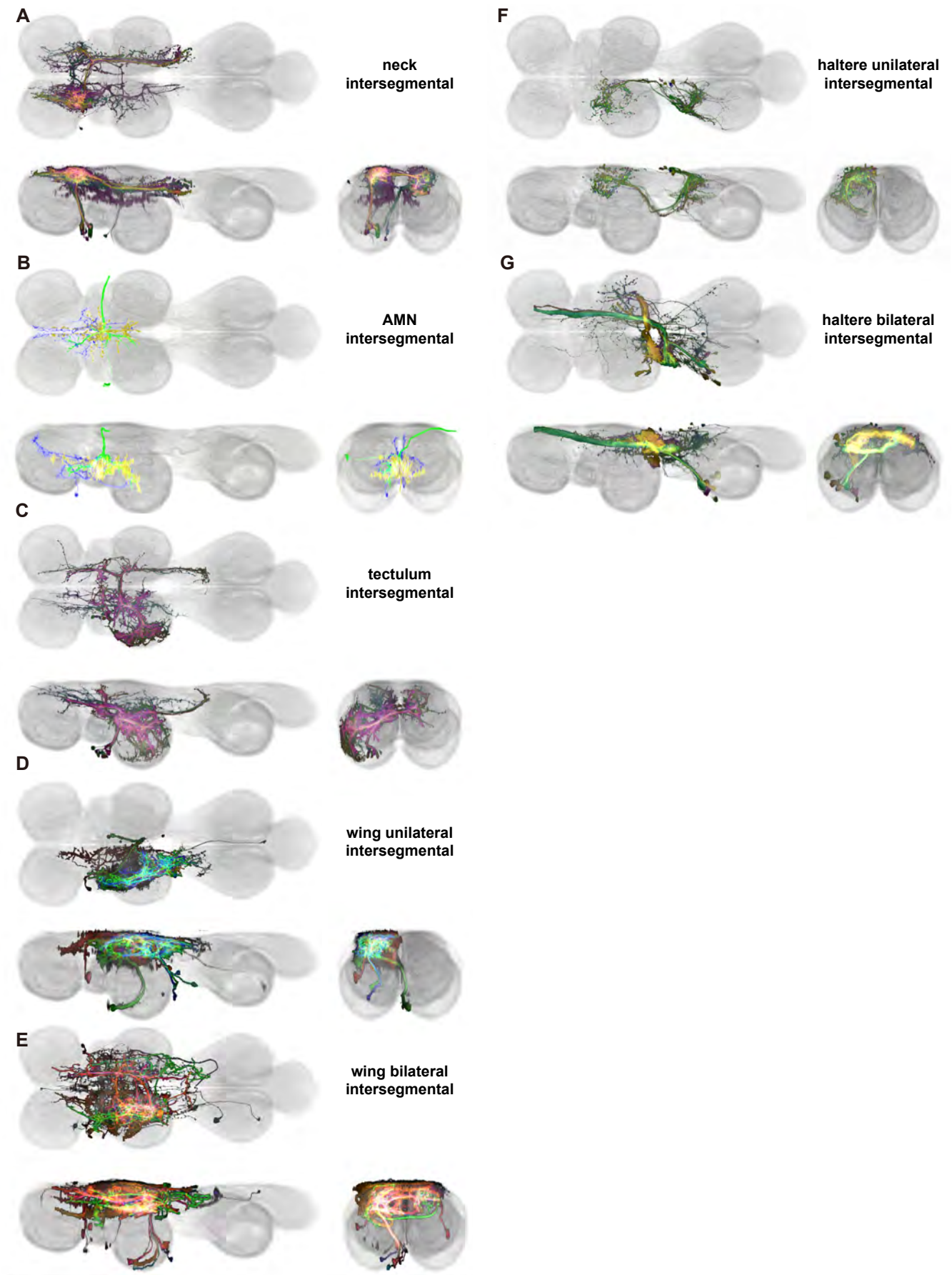

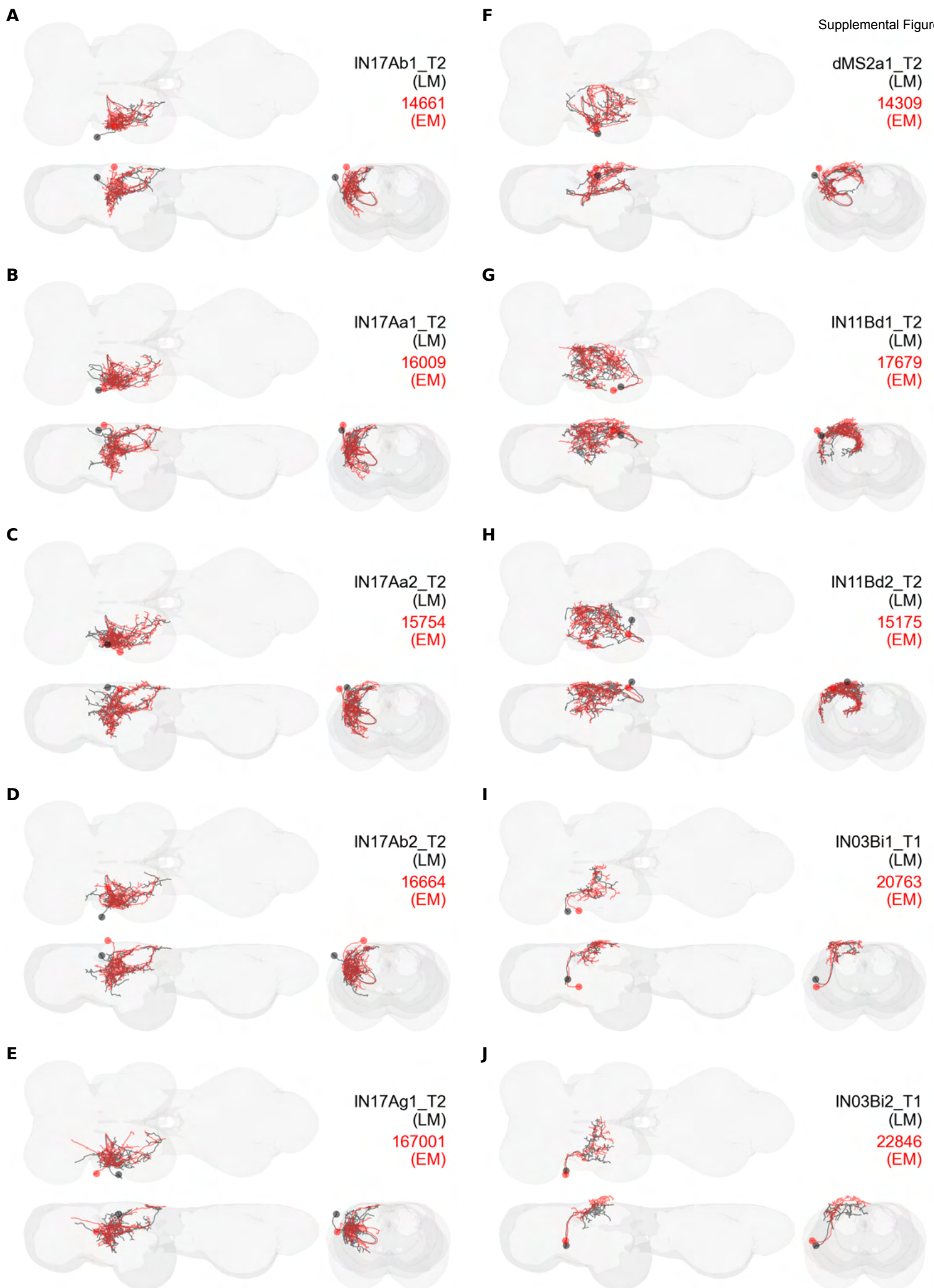

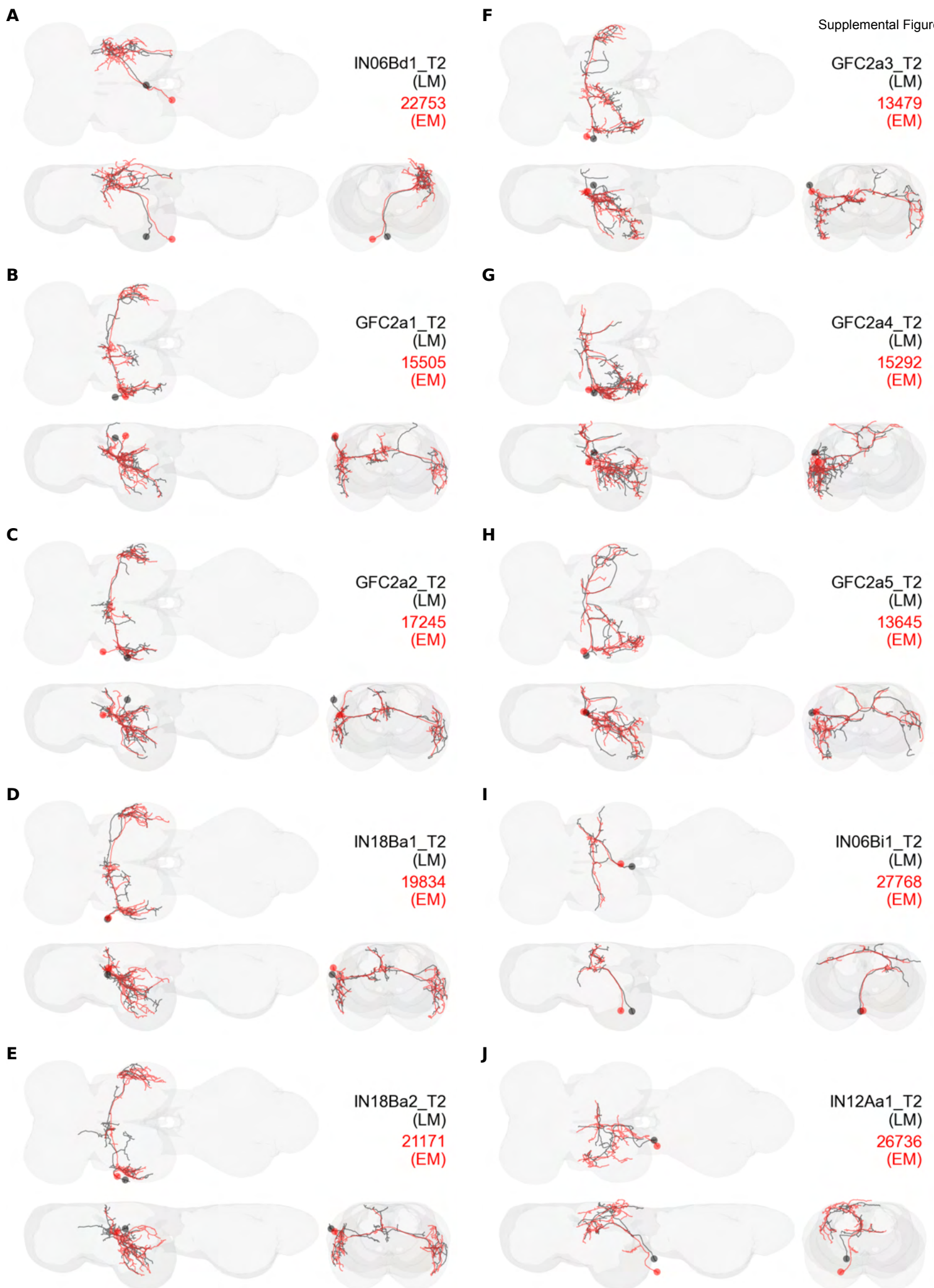

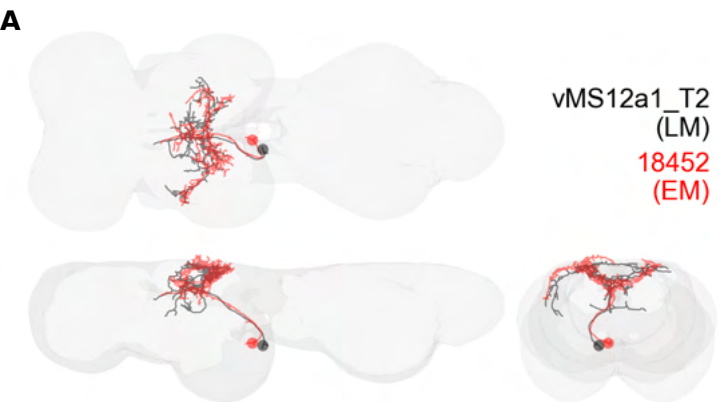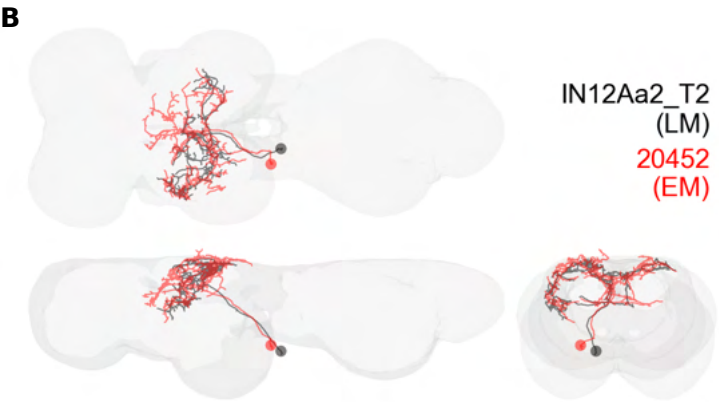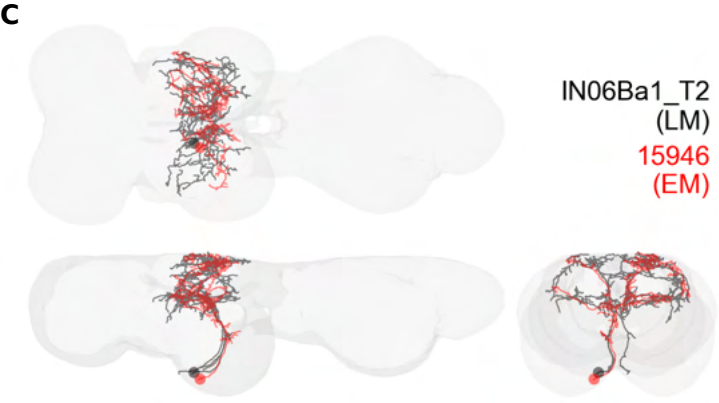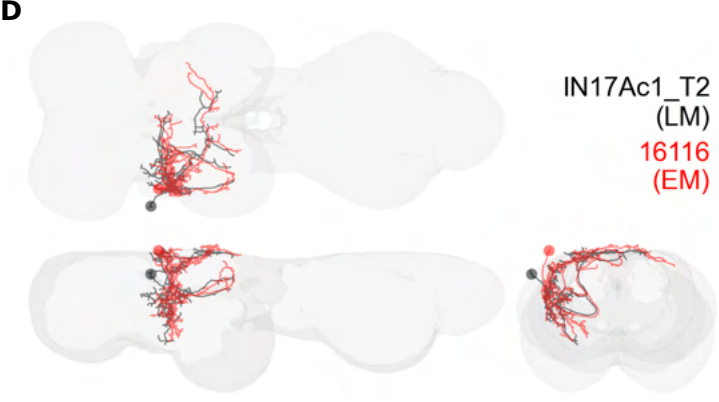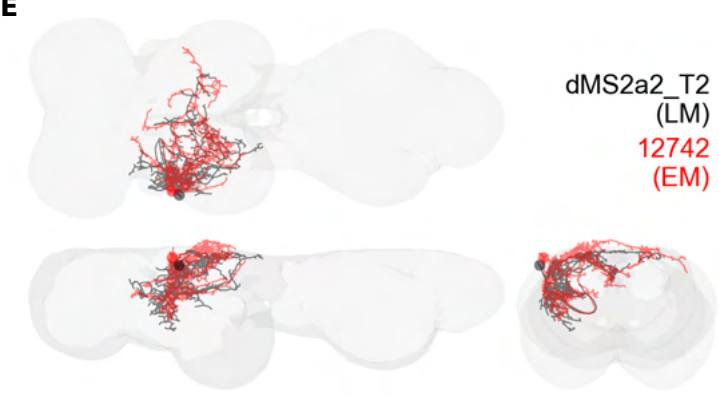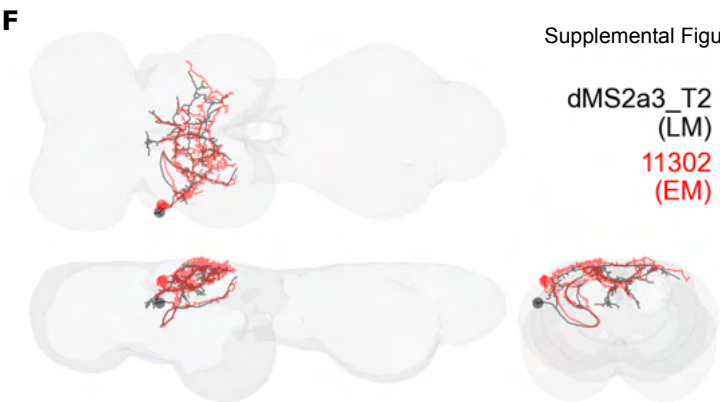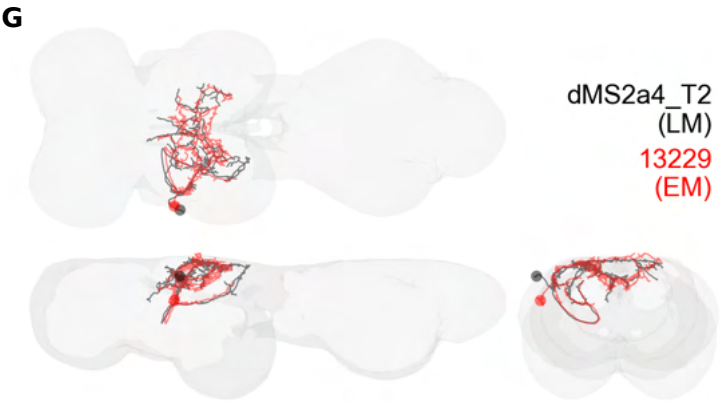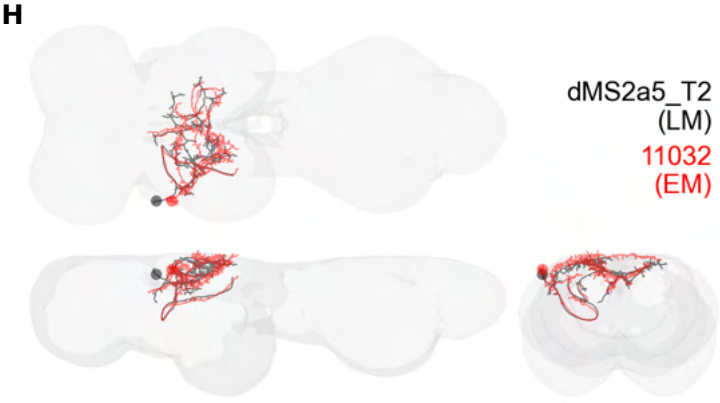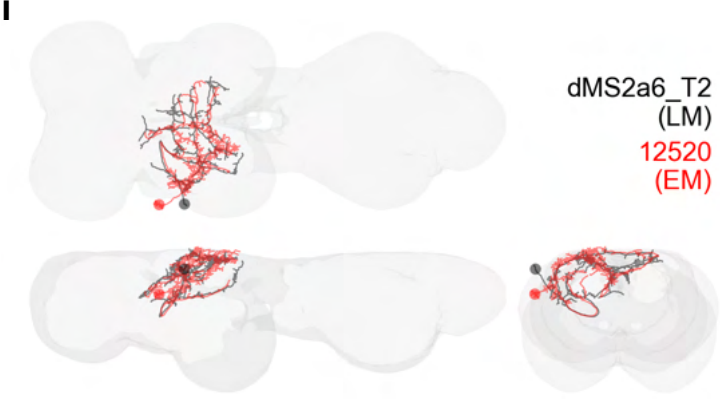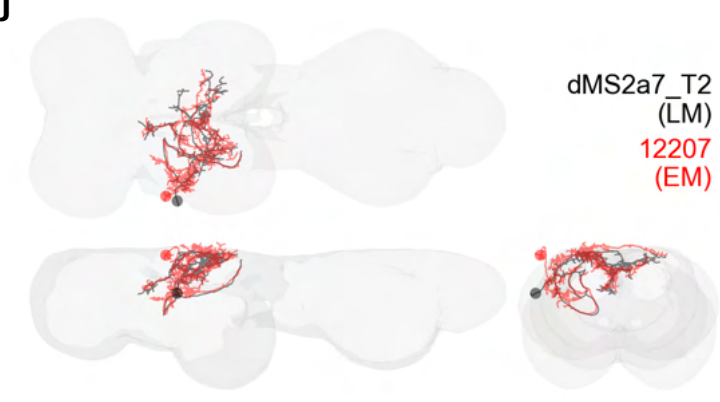

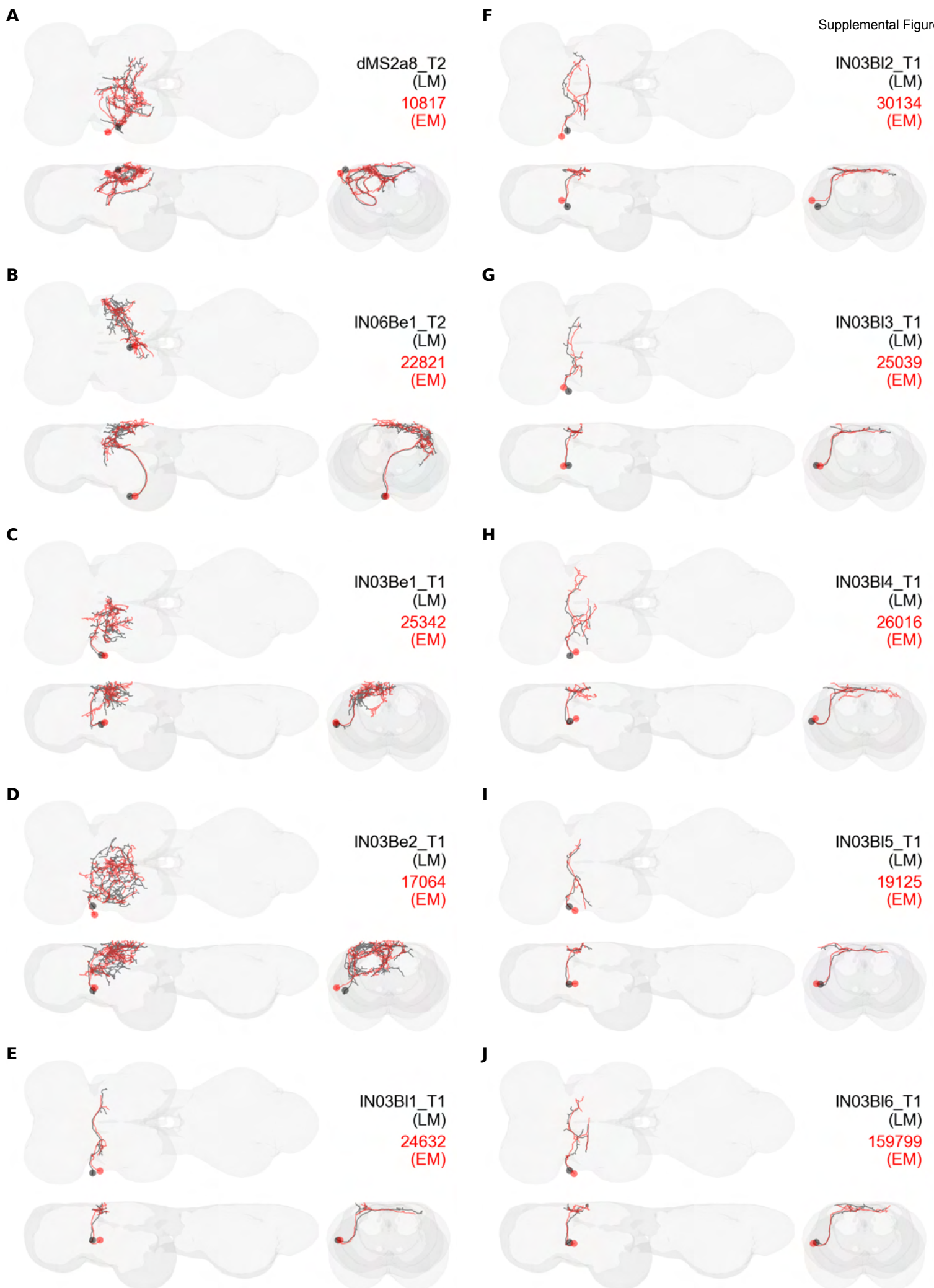

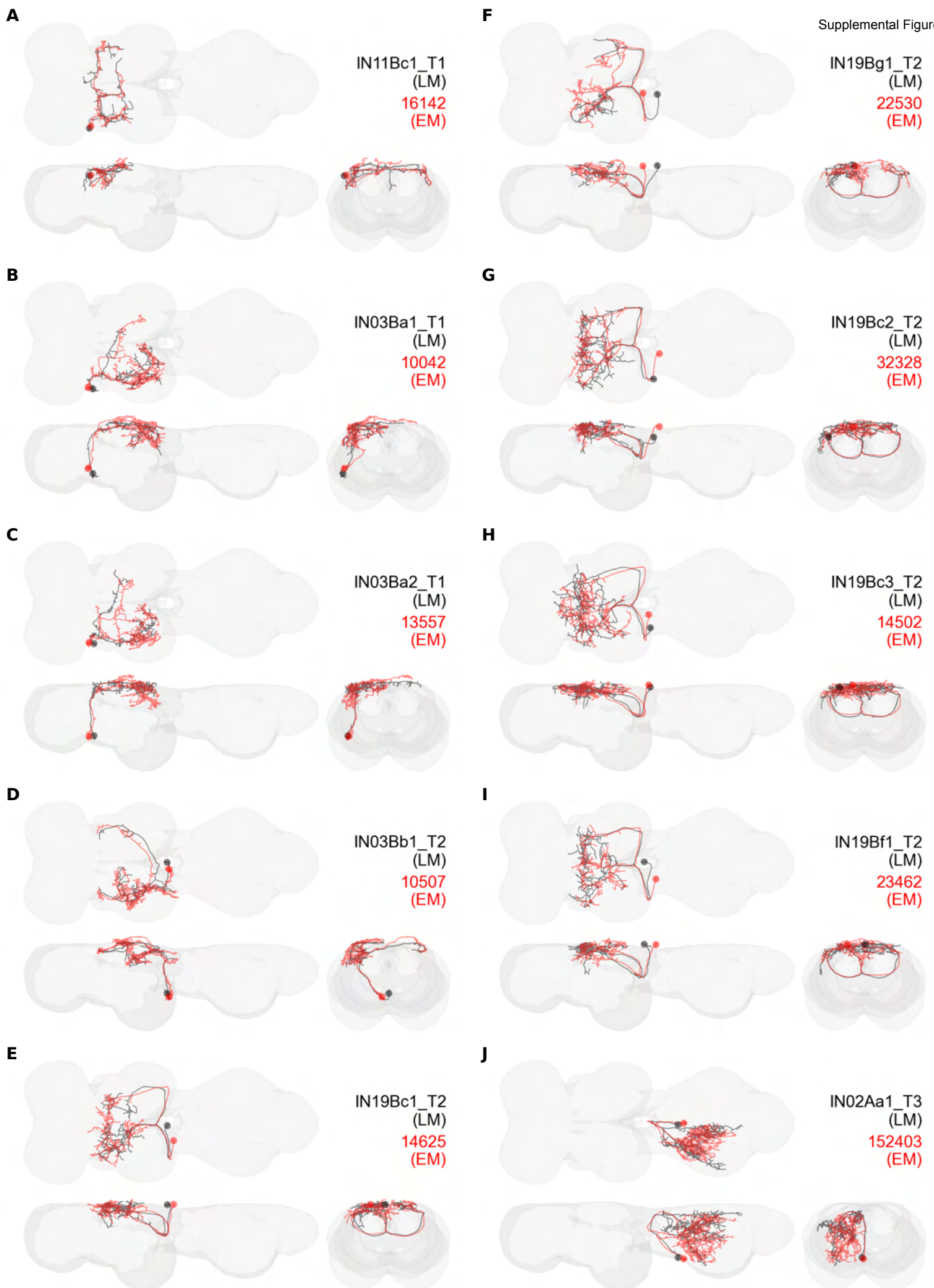

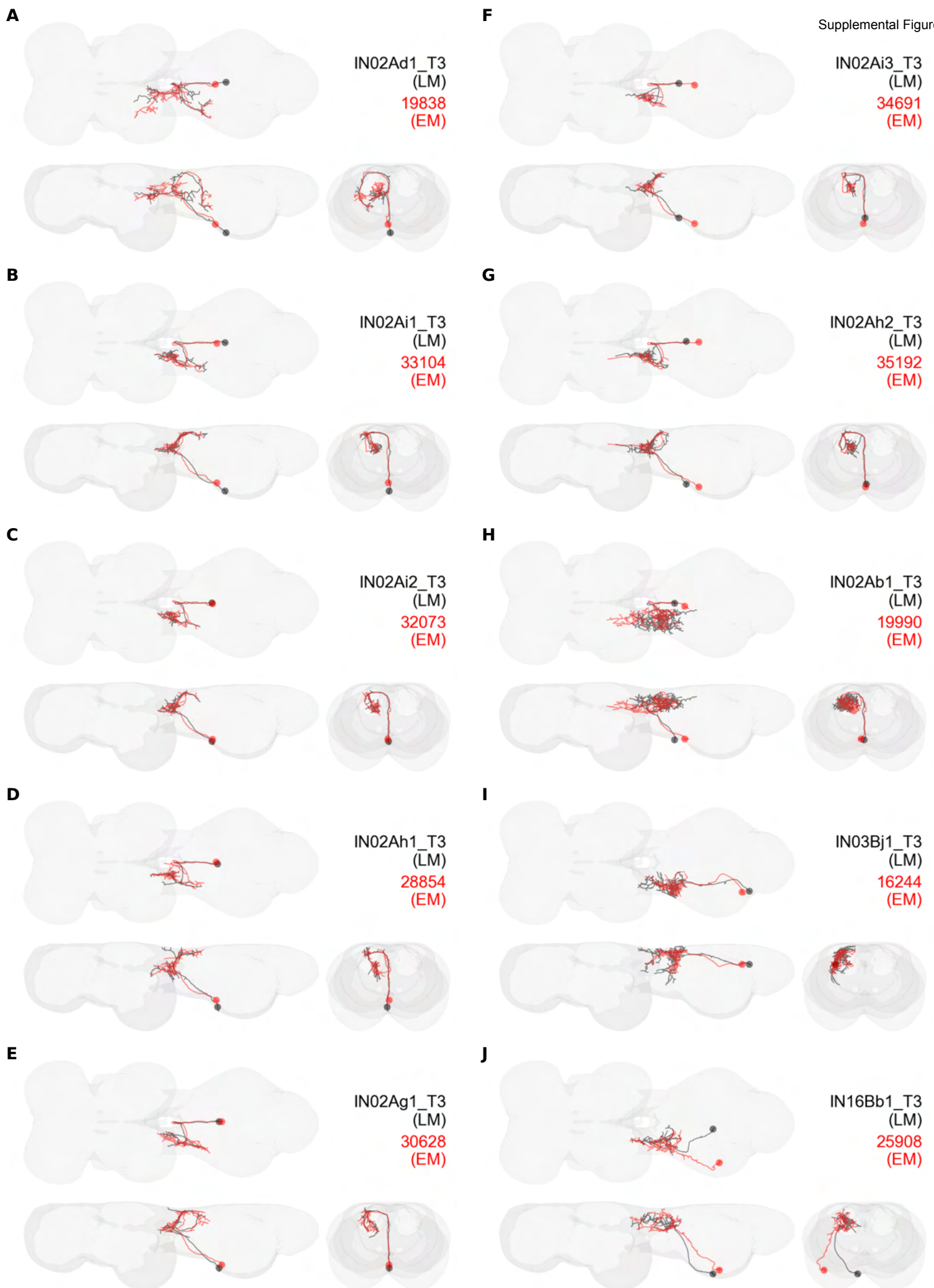

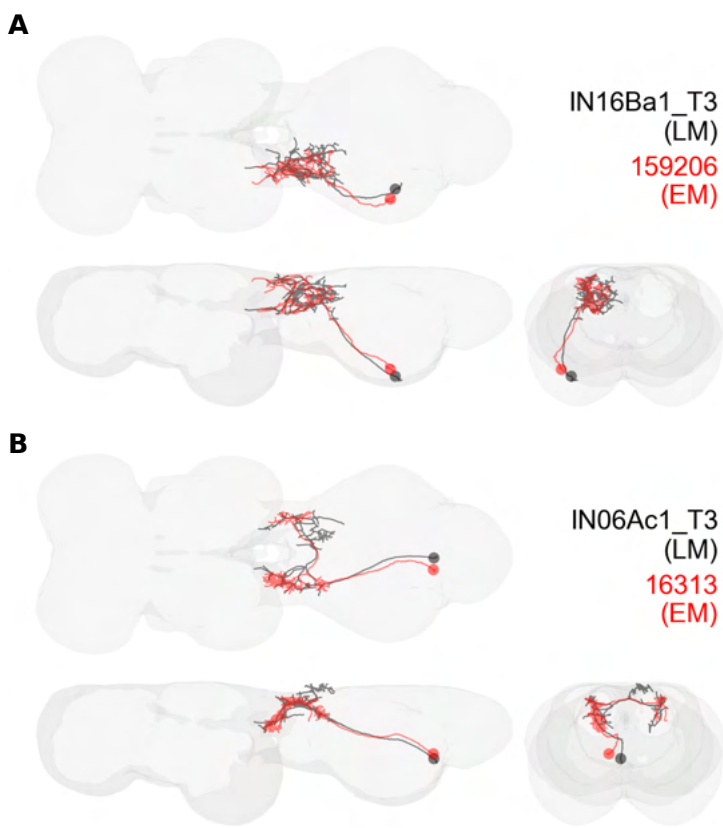

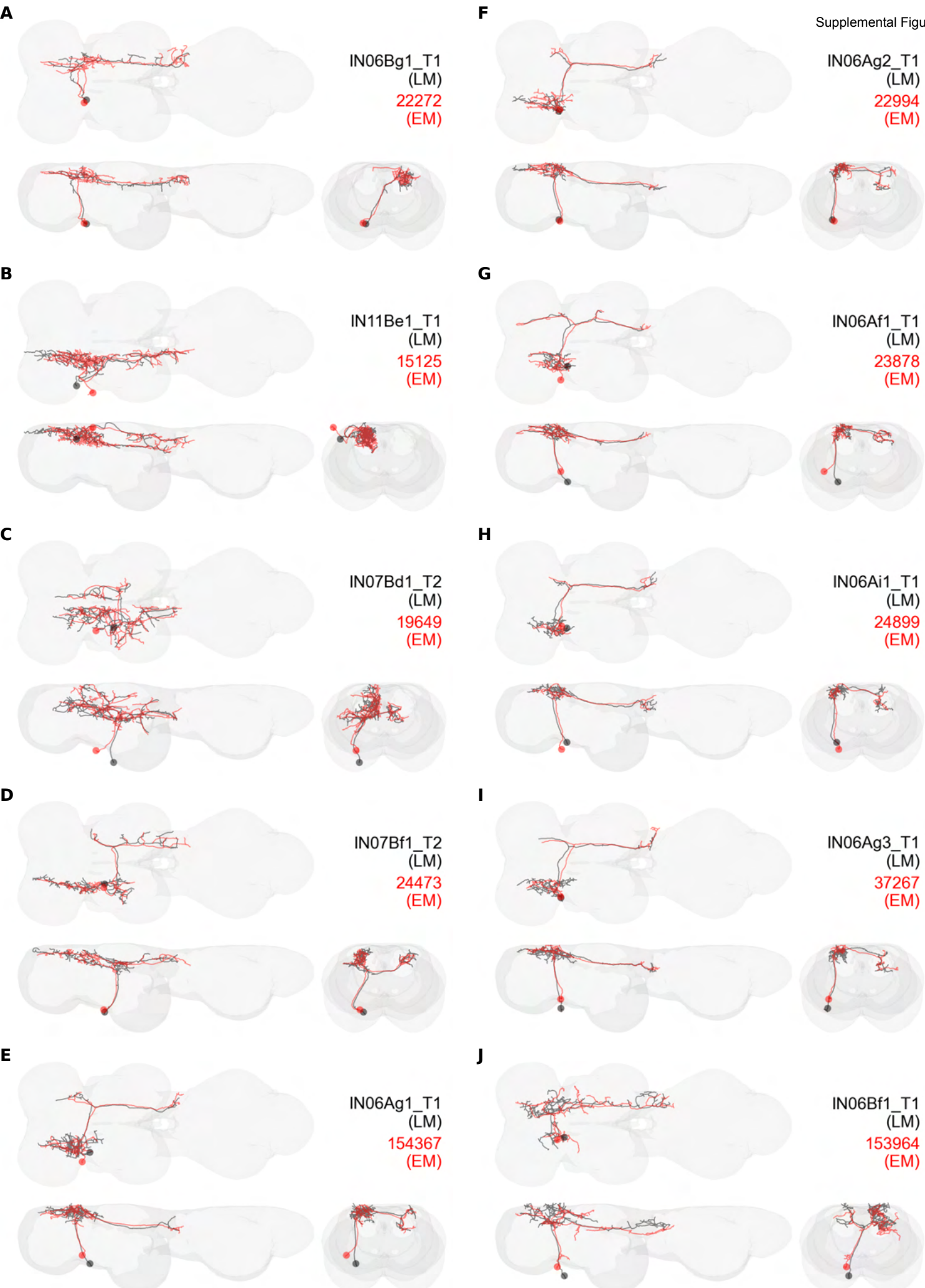

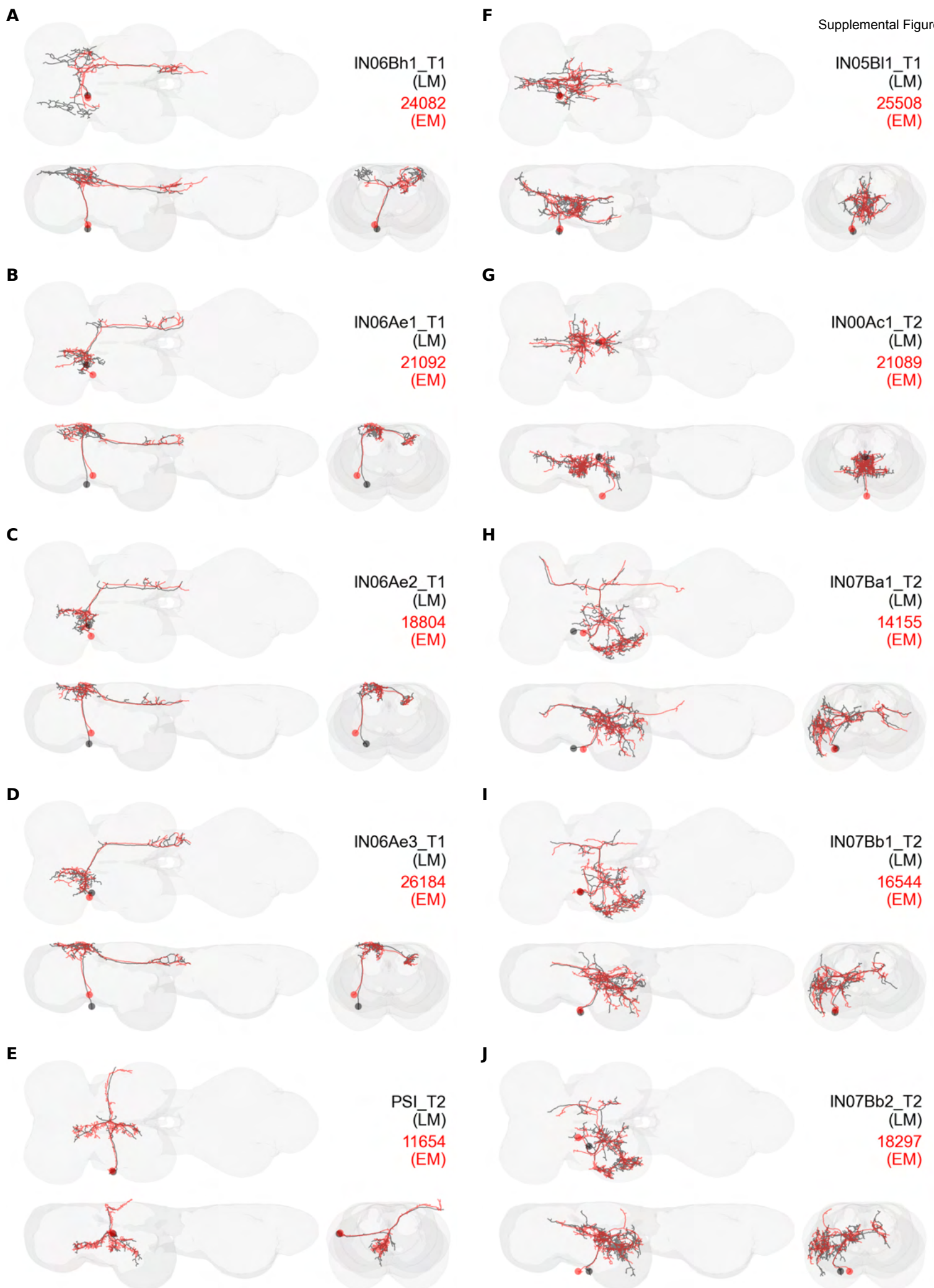

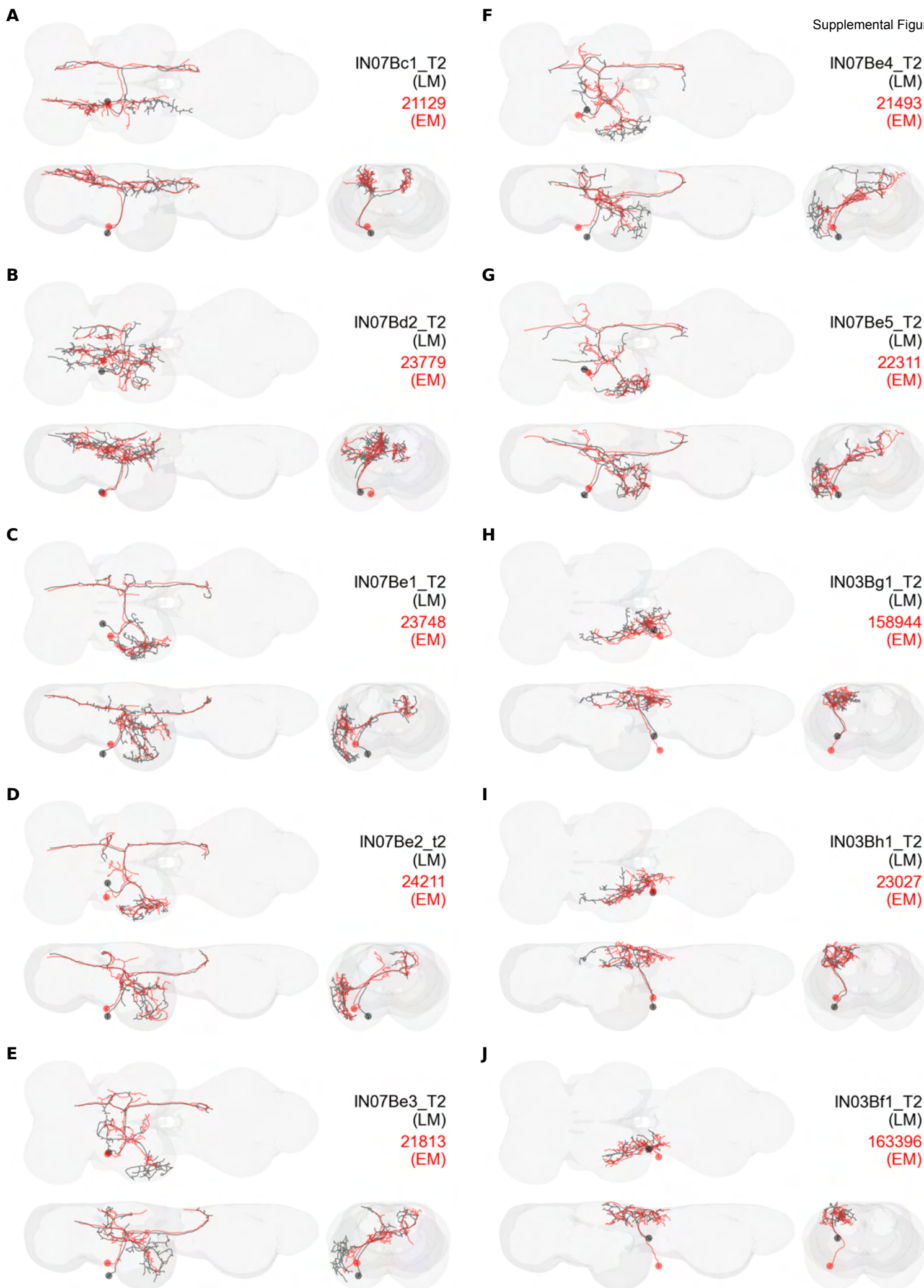

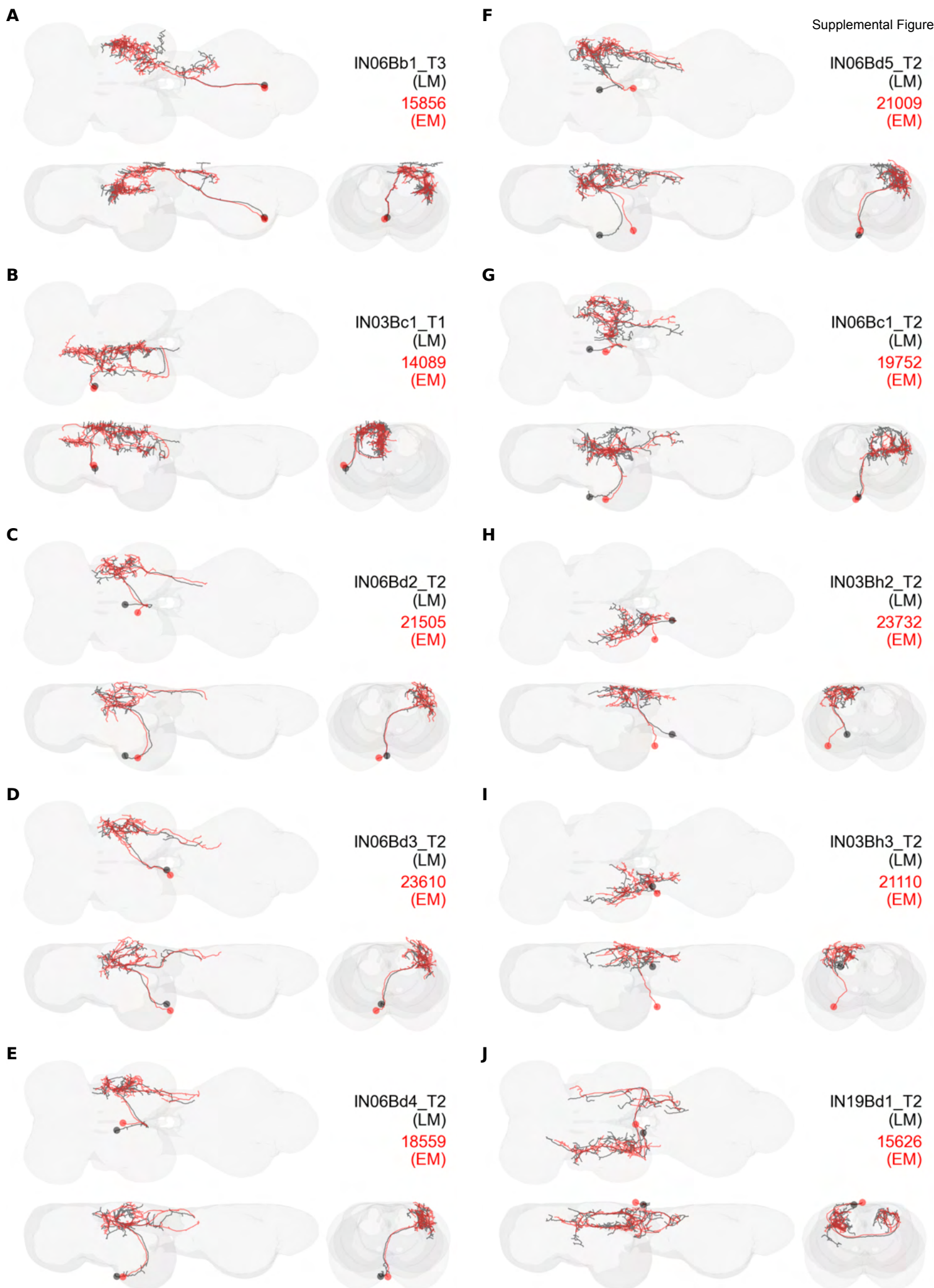

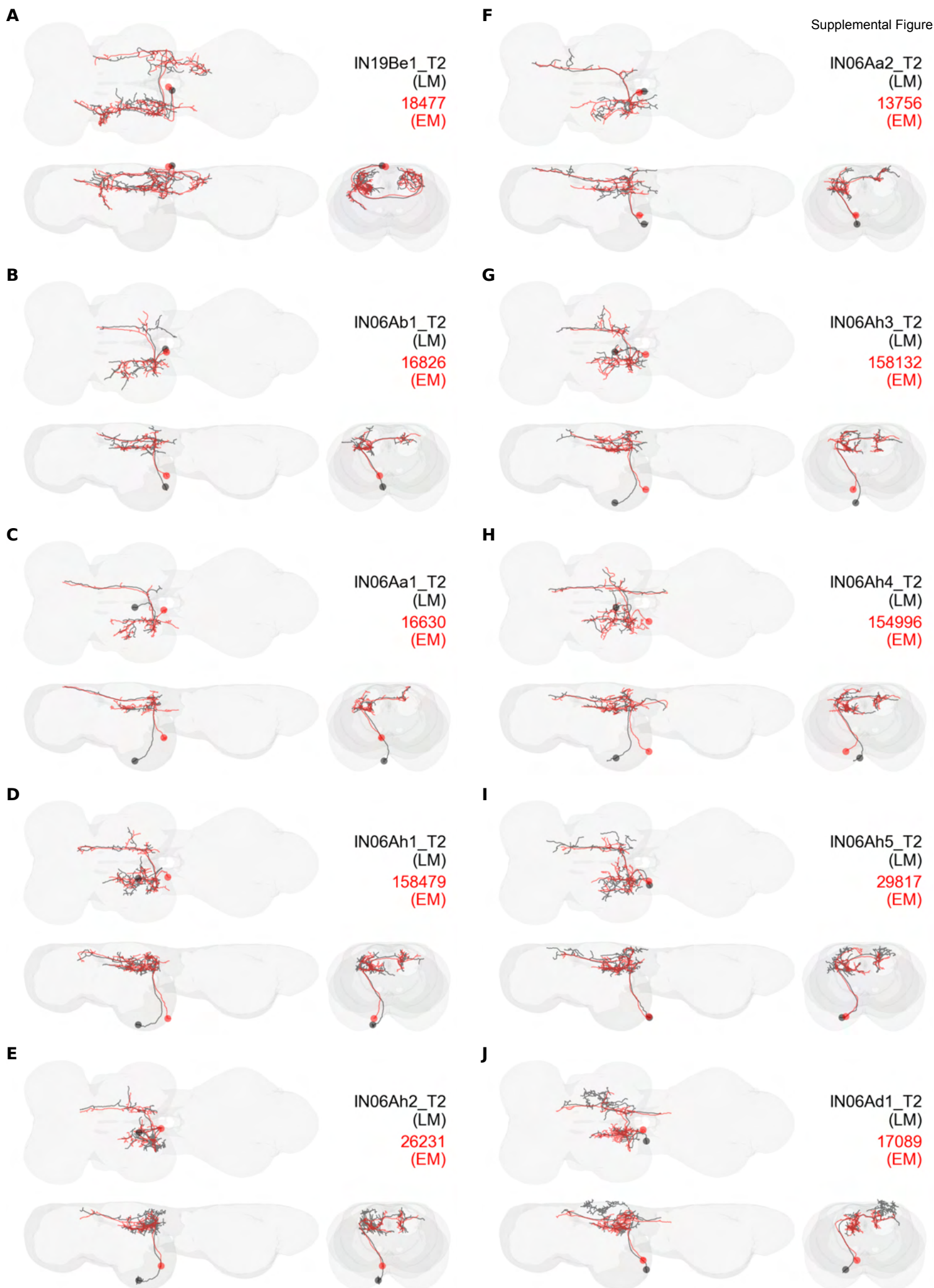

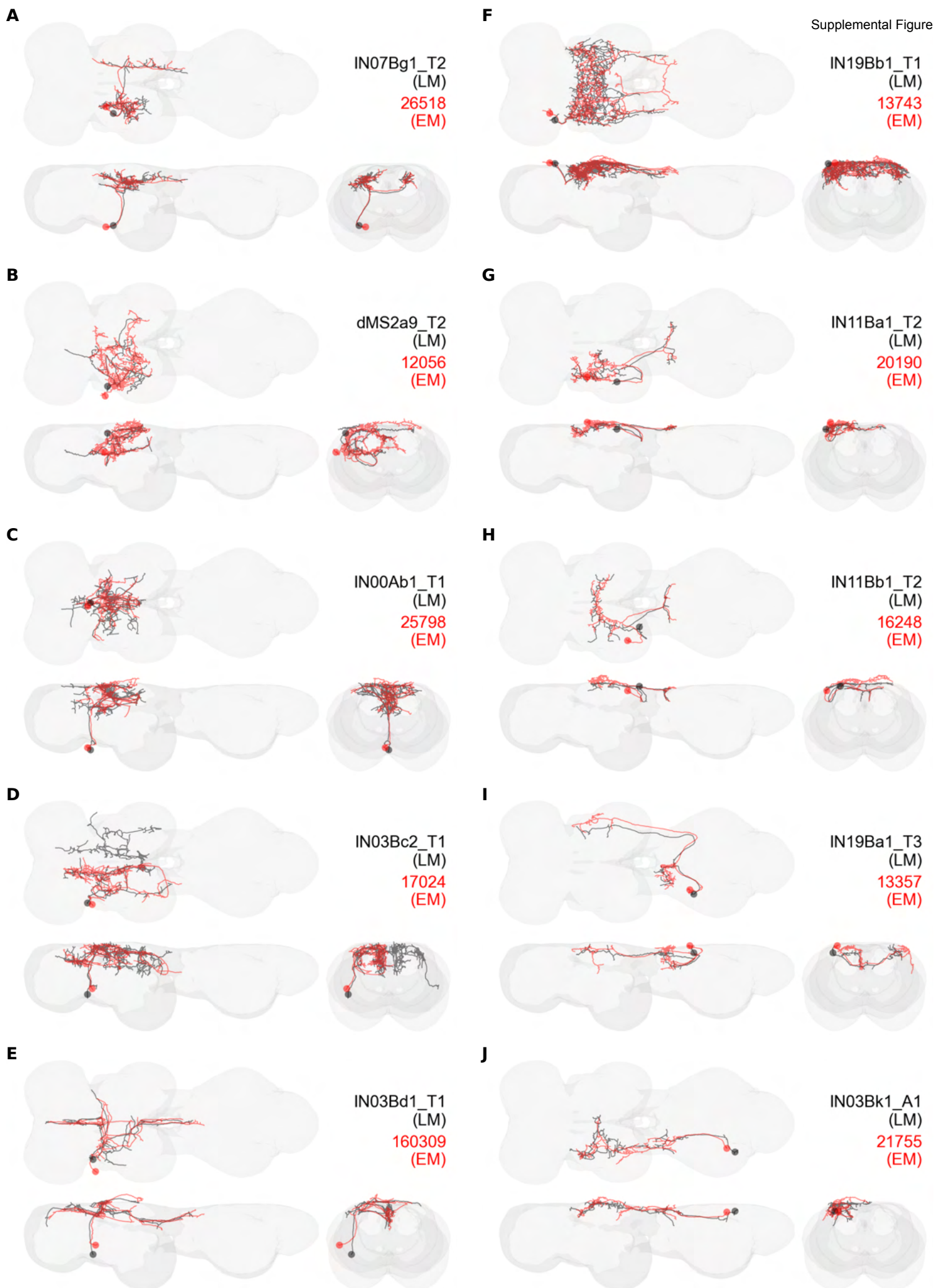

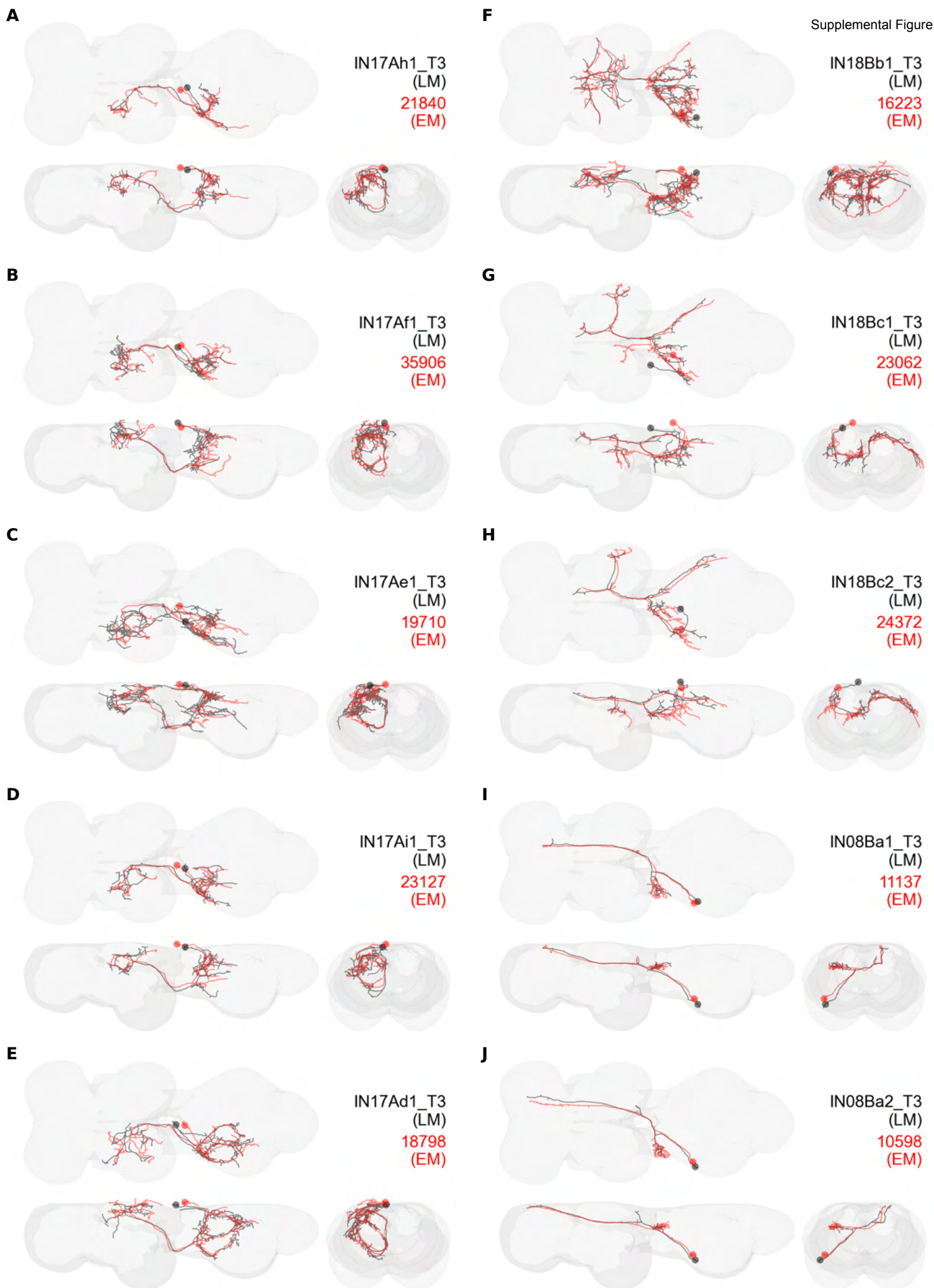

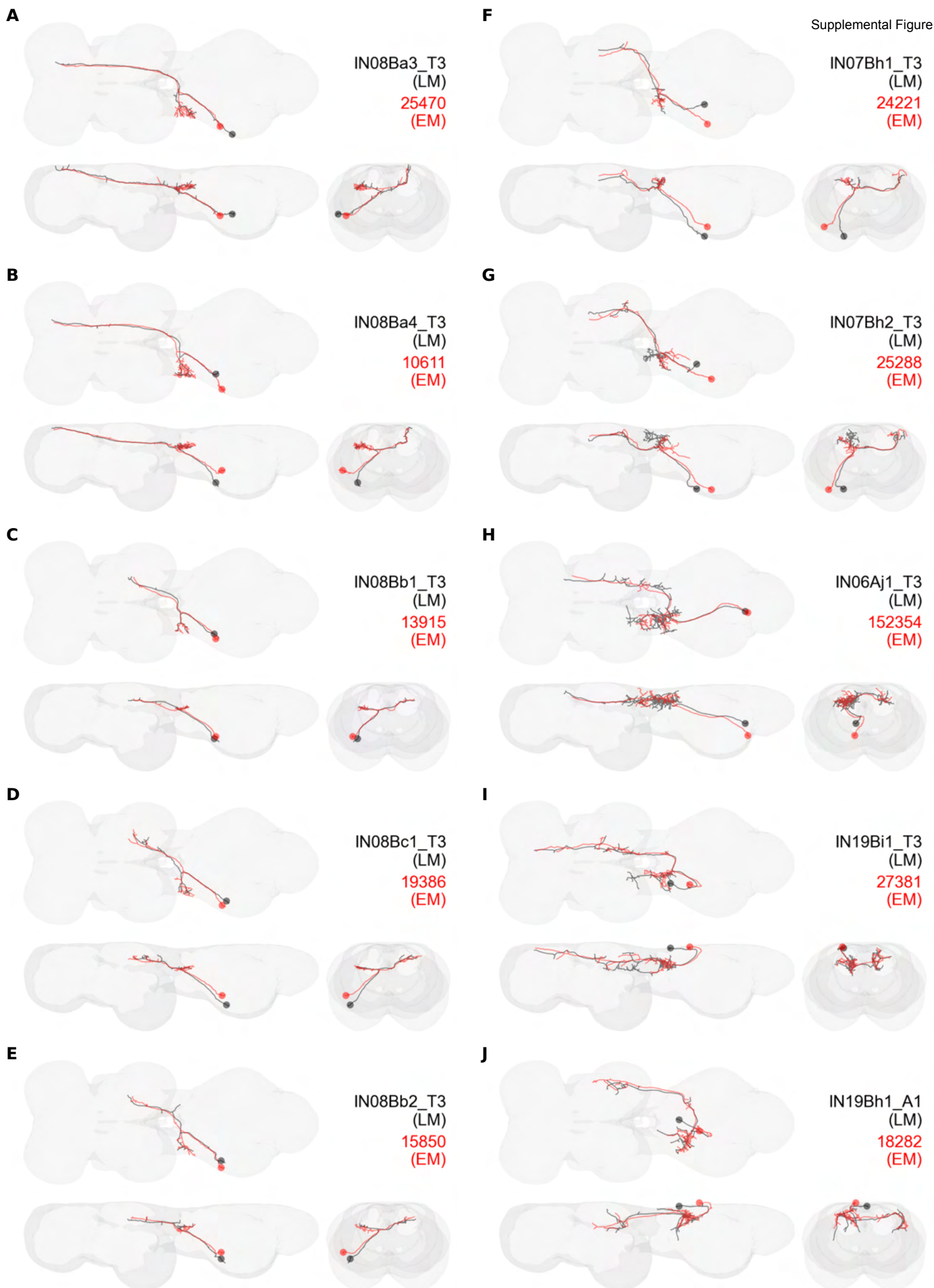

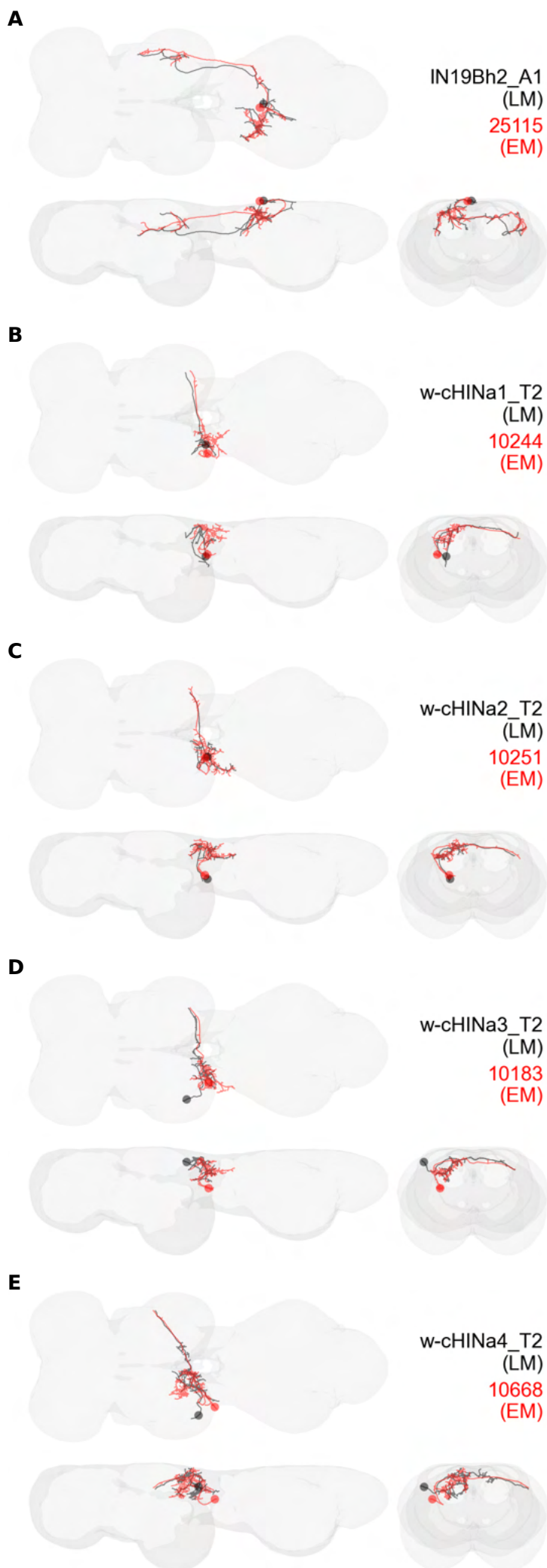

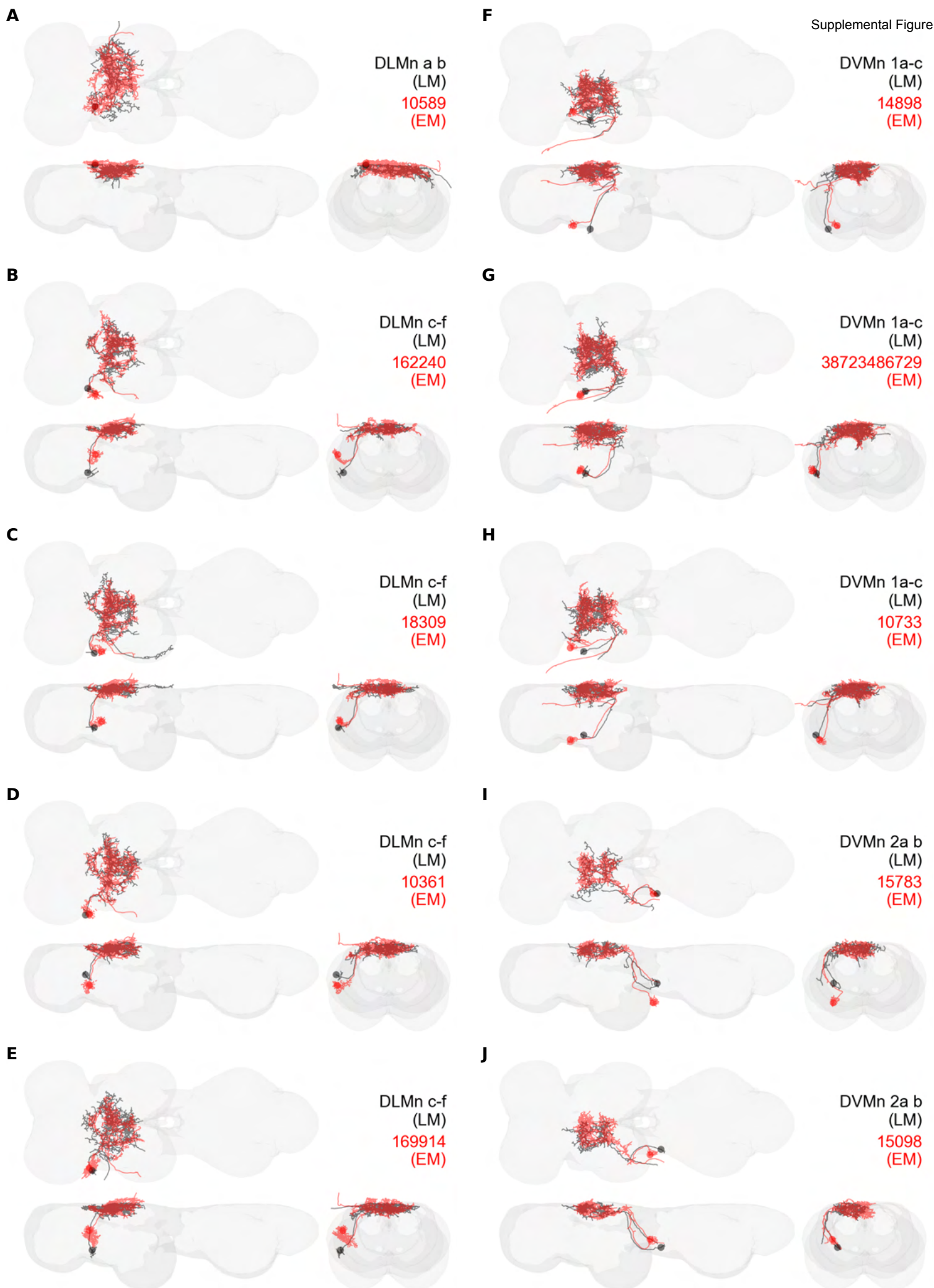

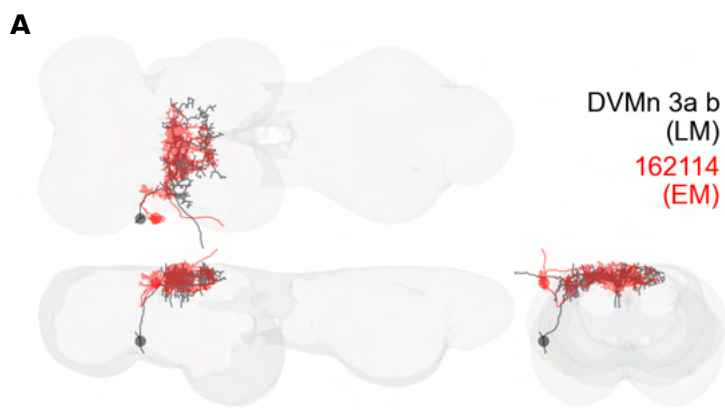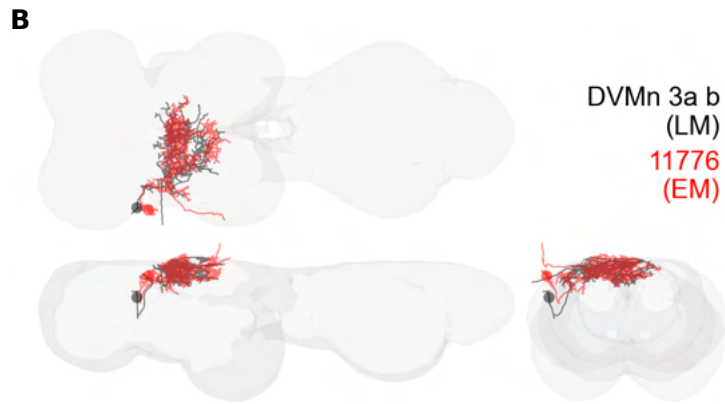

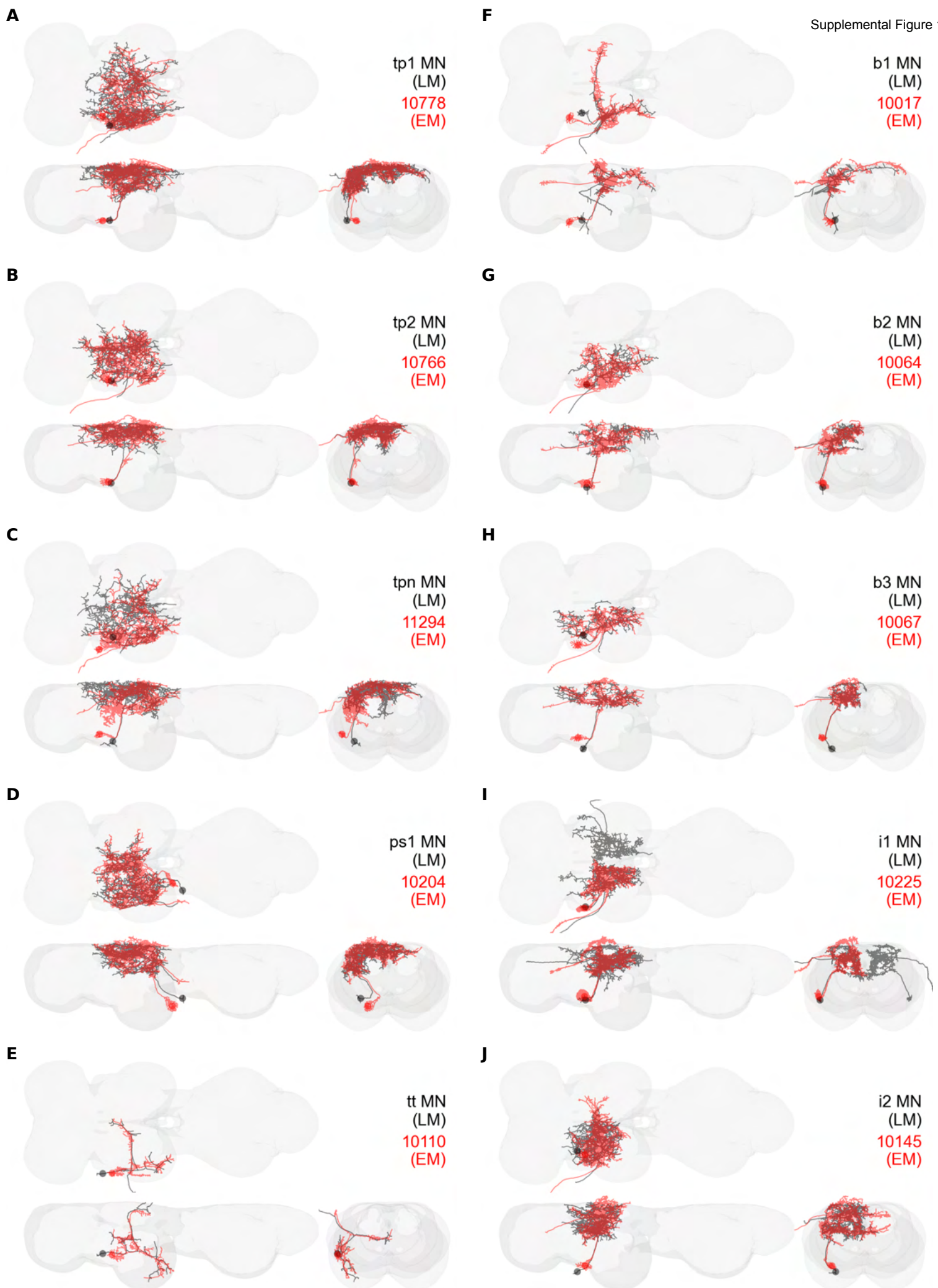

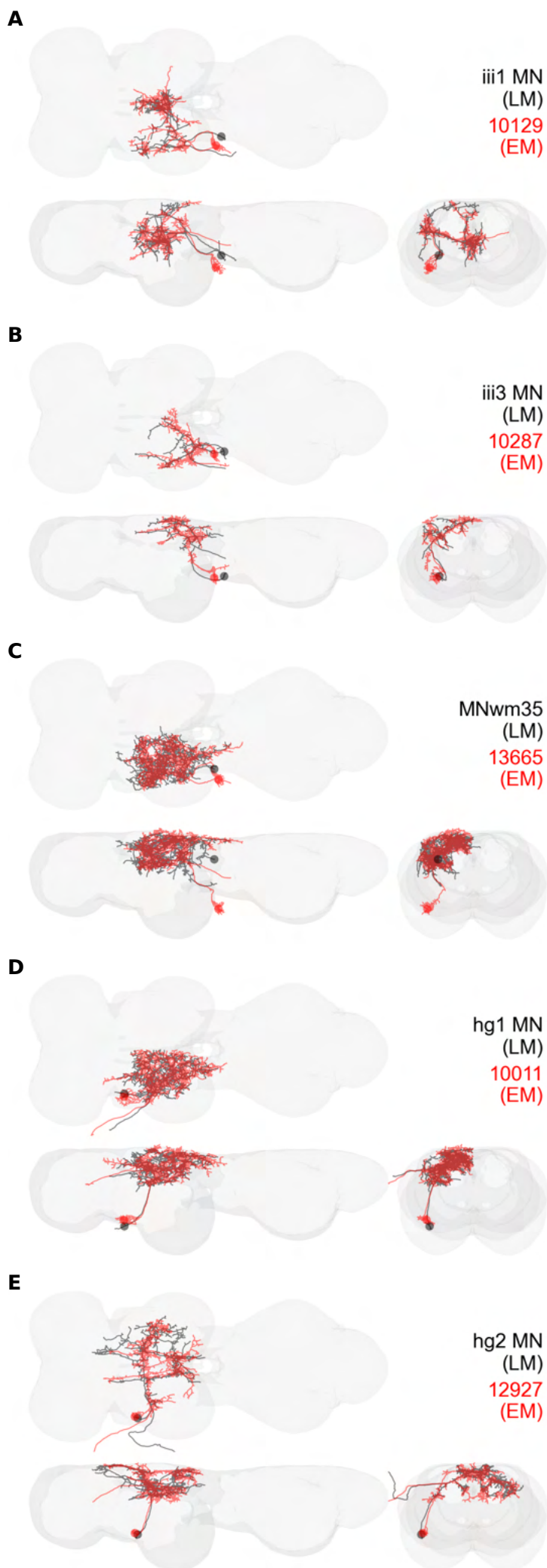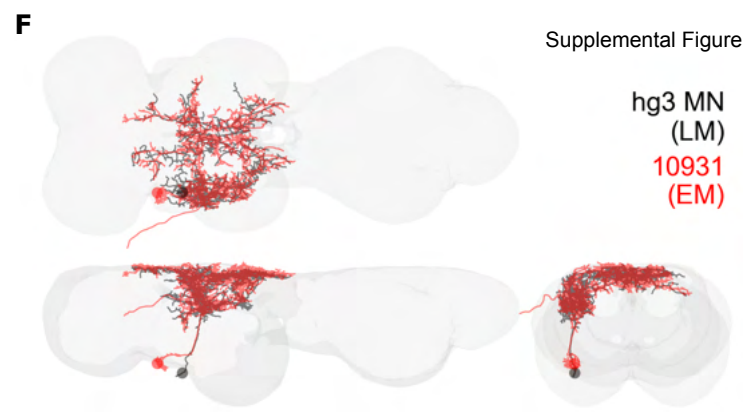

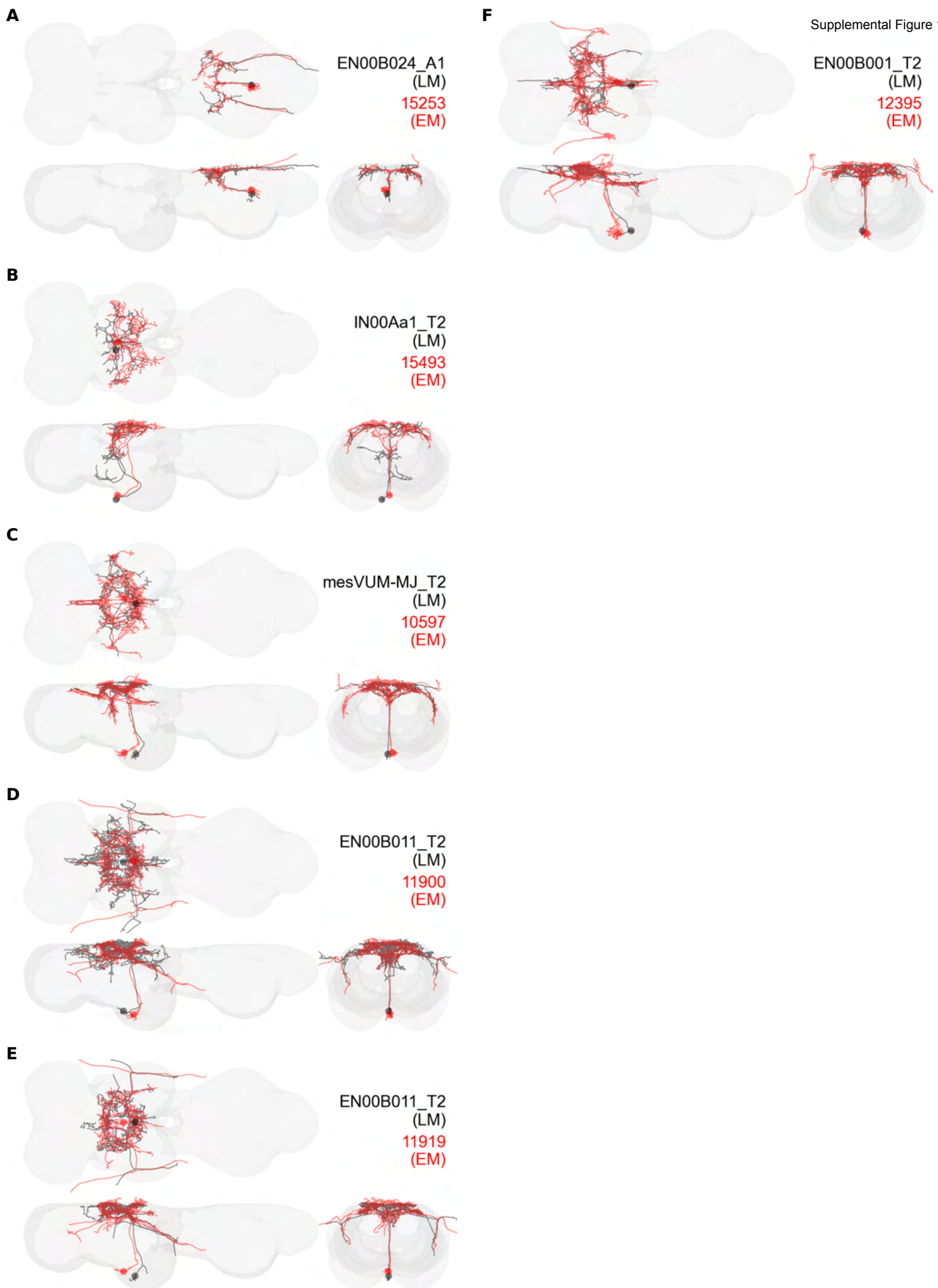

Supplement: Supplement 6 [file media-6.pdf]
