# Supplementary material for: Single-cell type analysis of wing premotor circuits in the ventral nerve cord of Drosophila melanogaster
Source: bioRxiv. 2023 Jun 1:2023.05.31.542897. Preprint. [Version 2] doi: 10.1101/2023.05.31.542897 (PMC10312520; doi:10.1101/2023.05.31.542897)

bioRxiv preprint doi: https://doi.org/10.1101/2023.05.31.542897; this version posted June 1, 2023. The copyright holder for this preprint (which was not certified by peer review) is the author/funder, who has granted bioRxiv a license to display the preprint in perpetuity. It is made available under aCC-BY 4.0 International license.

Supplemental Figure 1

Figure S1: VNC expression patterns of split-GAL4 driver lines stabilized in this project. *Each split line was crossed with pJFRC51-3xUAS-Syt::smGFP-HA in su(Hw)attP1; pJFRC225-5xUAS-IVS-myr::smGFP-FLAG in VK00005. This figure shows the male expression pattern, except in lines which had no male expression, such as SS51528. Each VNC stack was aligned to the JRC 2018 VNC Unisex template. These maximum intensity projections are color coded to indicate the depth of each pixel. Blue represents the ventral layers of the VNC while red represents the dorsal layers. Panels are arranged by split number, except that splits targeting leg neurons which were serendipitously produced in this project are arranged in the last 18 panels.*

Supplemental Figure 2

Figure 29: Morphology of identified intrasegmental interneurons. *MCFO was used to separate interneurons. Each neuron is shown from three views—horizontal (top left), sagittal (bottom left), and transverse (bottom right)—along with neuron name and hemilineage identity (top right). Images taken from preparations of male flies, segmented to isolate individual neurons, and aligned to the JRC 2018 Unisex VNC template. The driver lines respectively are below.*

Page 1 (intrasegmental): SS44314, SS43546, SS36094, SS36094, SS44314, SS31472, SS49042, SS33409, SS49042, SS25511.
Page 2 (intrasegmental): SS42439, SS42442, SS42442, SS42442, SS42438, SS42439, SS48215, SS48215, SS48268, SS47192.
Page 3 (intrasegmental): SS45607, SS45843, SS45843, SS45830, SS45843, SS25482, SS31309, SS31263.
Page 4 (intrasegmental): SS46725, 26A08 (no single-cell image appeared in the MCFO of the split GAL4 line targeting this cell, SS48272, so a generation 1 GAL4 line image was used), SS48272, SS48272, SS49799, SS49766, SS42438, SS40783, SS42442, SS46675.
Page 5 (intrasegmental): SS46675, SS40783, SS42438, SS42447, SS49779, SS51830, SS37306, SS52389, SS52389, SS52389.
Page 6 (intrasegmental): SS52389, SS52389, SS48268, SS44002, SS44003, SS47192, SS37233, SS20796, SS20796, SS49041.
Page 7 (intrasegmental): SS40778, SS54506, SS44314, SS54506, SS54506, SS54506, SS25502, SS54506, SS54506, 50G08 (no single-cell images were produced from the split GAL4 line targeting this cell, SS54506, so a generation 1 GAL4 image was used).
Page 8 (intrasegmental): SS49125, SS31274, SS25478, SS45611.

Supplemental Figure 3

Figure 37: Morphology of identified intersegmental interneurons. *MCFO was used to separate interneurons. Each neuron is shown from three views—horizontal (top left), sagittal (bottom left), and transverse (bottom right)—along with neuron name and hemilineage identity (top right). Images taken from preparations of male flies, segmented to isolate individual neurons, and aligned to the JRC 2018 Unisex VNC template. The driver lines respectively are below.*

bioRxiv preprint doi: https://doi.org/10.1101/2023.05.31.542897; this version posted June 1, 2023. The copyright holder for this preprint (which was not certified by peer review) is the author/funder, who has granted bioRxiv a license to display the preprint in perpetuity. It is made available under aCC-BY 4.0 International license.

Page 9 (intersegmental): SS54495, SS48619, SS60603, SS31899, SS32400, SS54480, SS49807, SS54495, VT014604 (no single cell images were produced from the split GAL4 driver lines targeting this cell, SS31309, SS30330, SS54480, SS54474 or SS54495, so a generation 1 GAL4 image was used), SS25553.
Page 10 (intersegmental): SS25553, SS42079, SS42079, SS42079, SS49809, SS33489, SS42475, SS49784, SS49784, SS49784.
Page 11 (intersegmental): SS28361, SS46735, SS42464, SS42464, SS31289, SS31290, SS3129, SS49800, SS49778.
Page 12 (intersegmental): SS49807, SS29871 SS49779, 22E12 (no single cell images were produced from the split GAL4 driver line targeting this cell, SS53421, so a generation 1 GAL4 image was used), 22E12 (no single cell images were produced from the split GAL4 driver lines targeting this cell, SS53421, SS42447 or SS40456, so a generation 1 GAL4 image was used), SS40456, SS49800, SS49800, SS40764, SS40764.
Page 13 (intersegmental): SS45385, SS45385, SS42446, SS42446, SS42446, SS48204, SS42446, SS48204, SS25553, SS40969.
Page 14 (intersegmental): SS42438, SS46295, SS49779, SS51830, SS40969, SS44005, SS49802, SS40969, SS29600, SS51531.
Page 15 (intersegmental): SS48709, SS51531, SS46260, SS46735, SS49777, 59F02 (no single-cell image appeared in the split GAL4 line targeting this cell, SS49777, so a generation 1 GAL4 image was used), SS42499, SS47214, SS47215.
Page 16 (intersegmental): SS44002, SS47215, SS49853, SS47215, SS49853, SS29535, SS29535. SS44276, SS31246, SS30816.
Page 17 (intersegmental): SS42050, SS49806, SS49806, SS47215, SS29602.

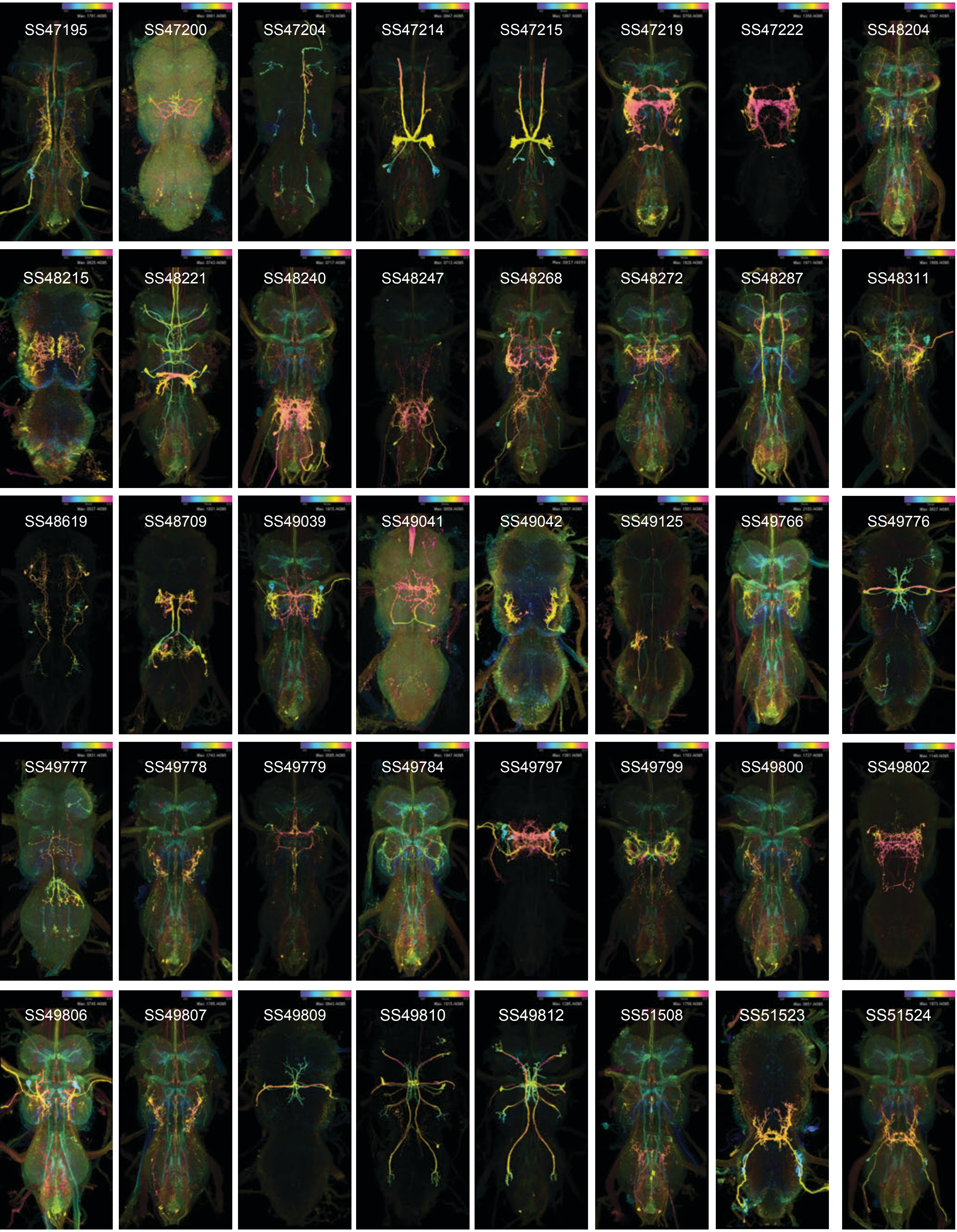

Supplementary Figure 1.1

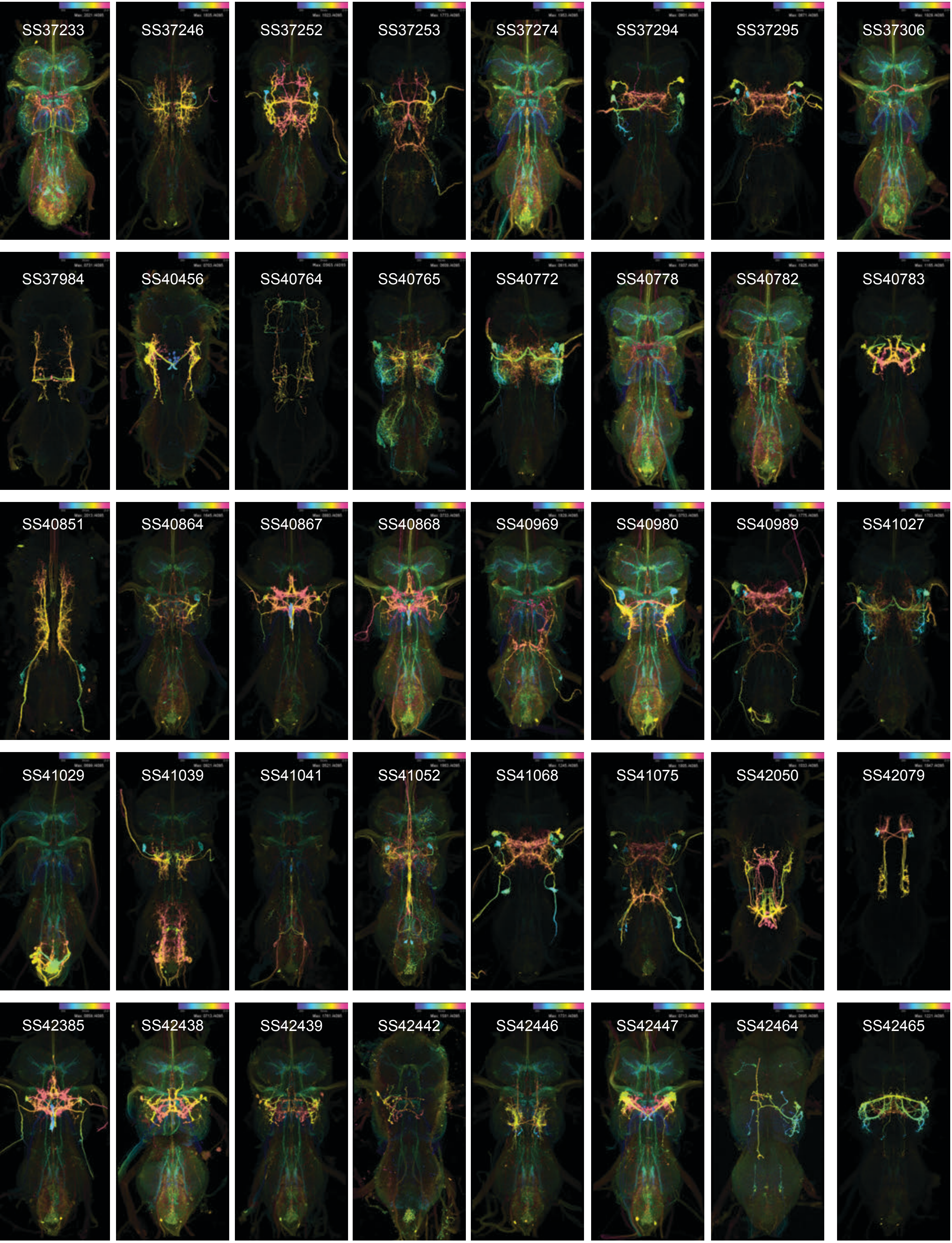

Supplementary Figure 1.2

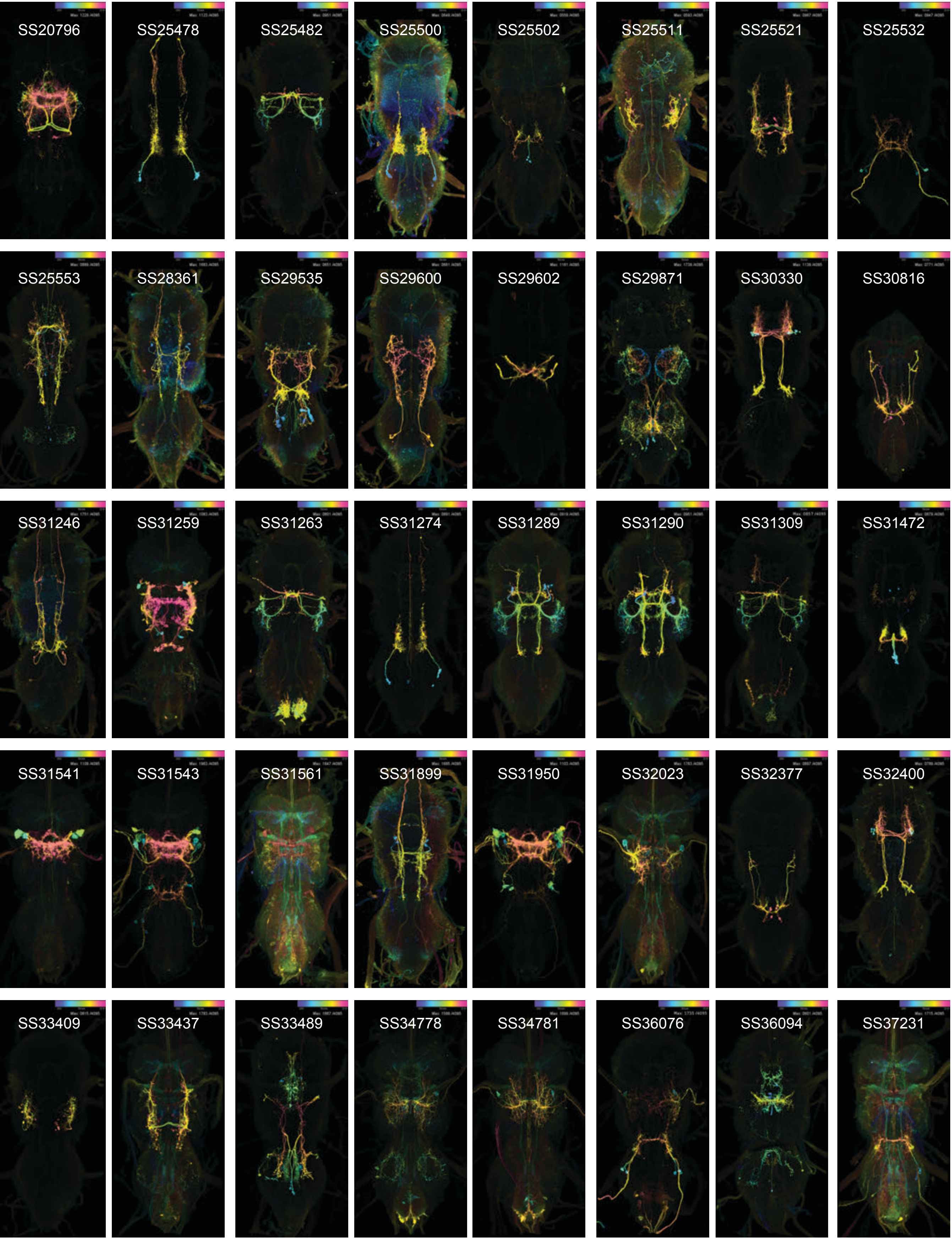

Supplementary Figure 1.3

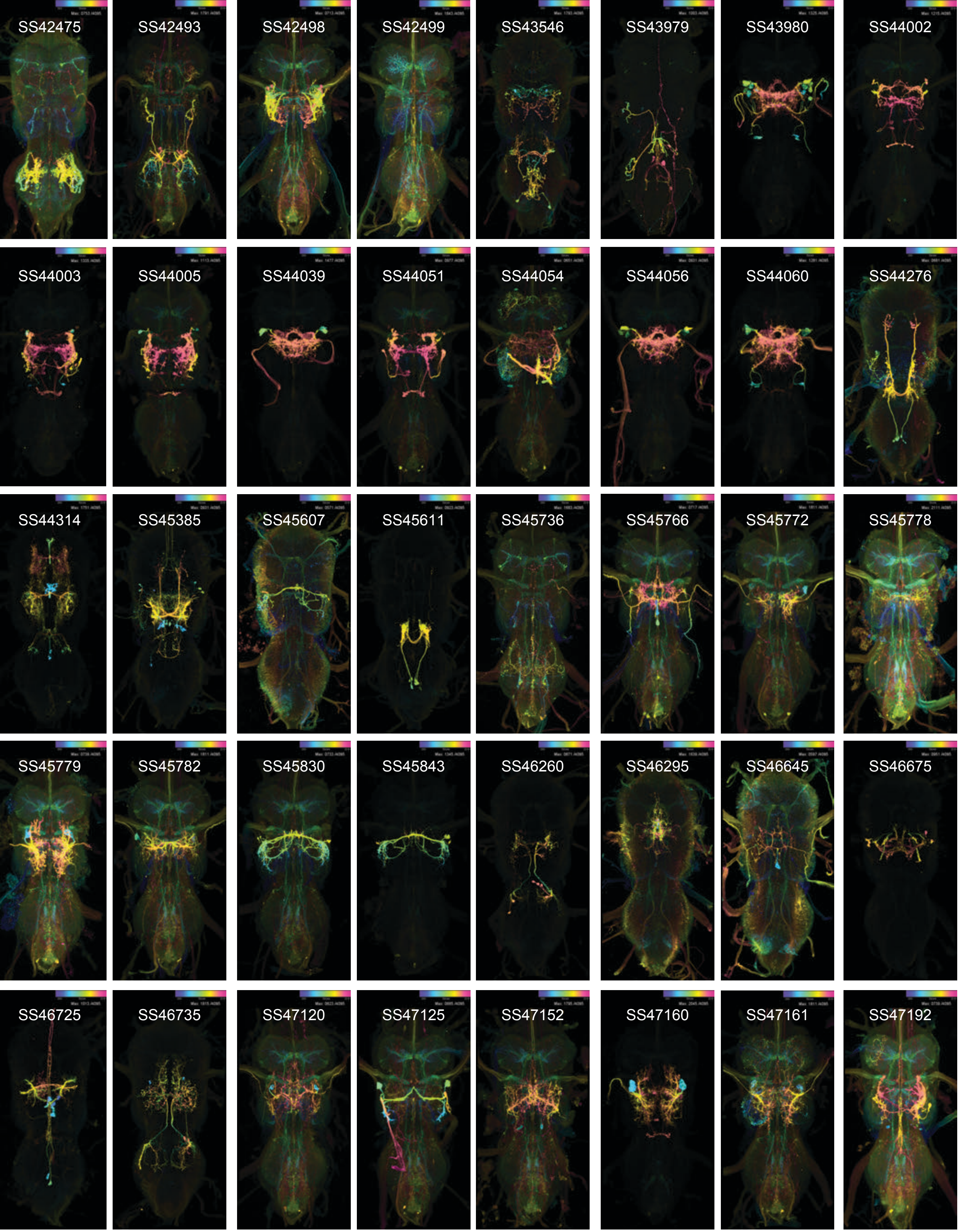

Supplementary Figure 1.4

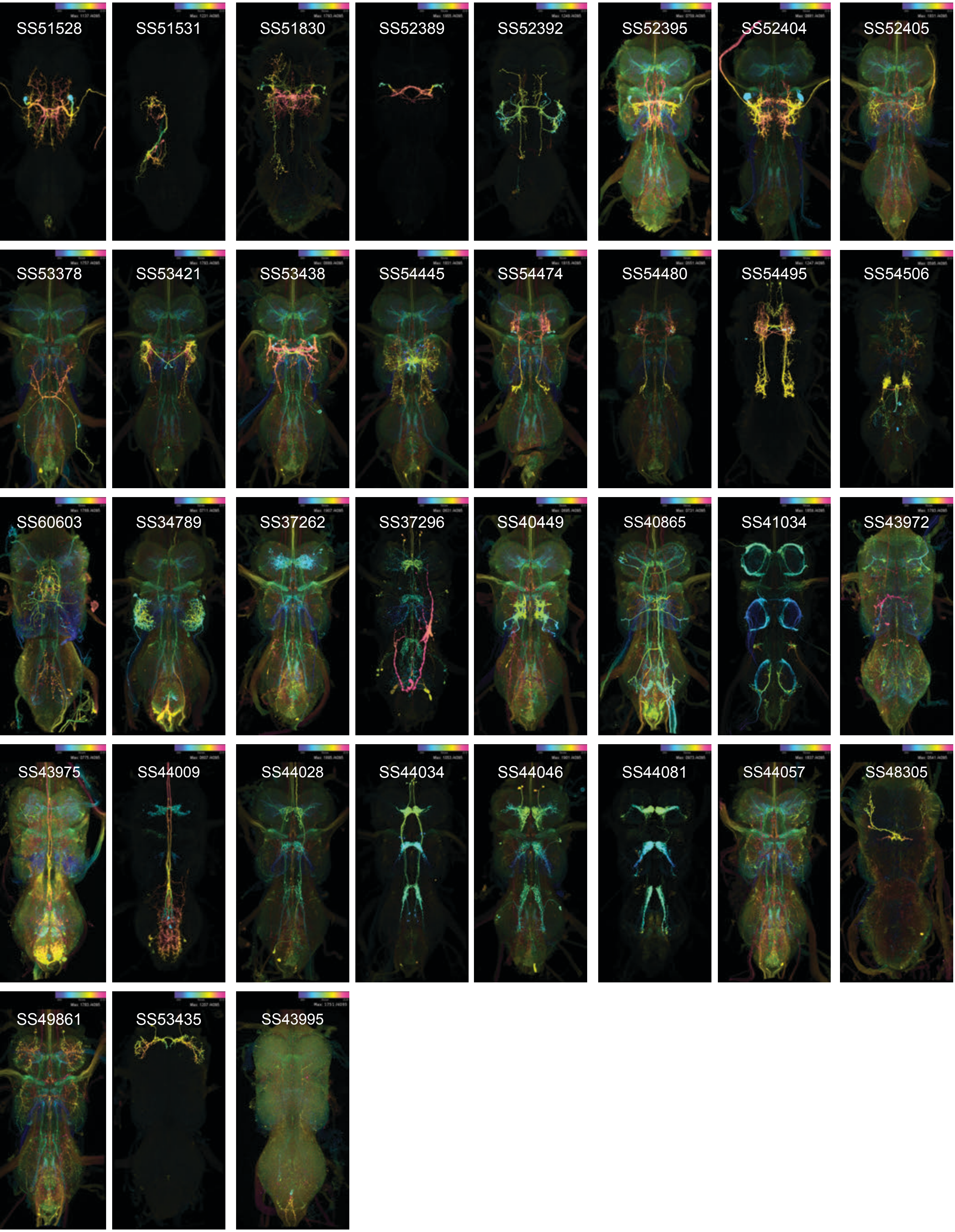

Supplementary Figure 1.5

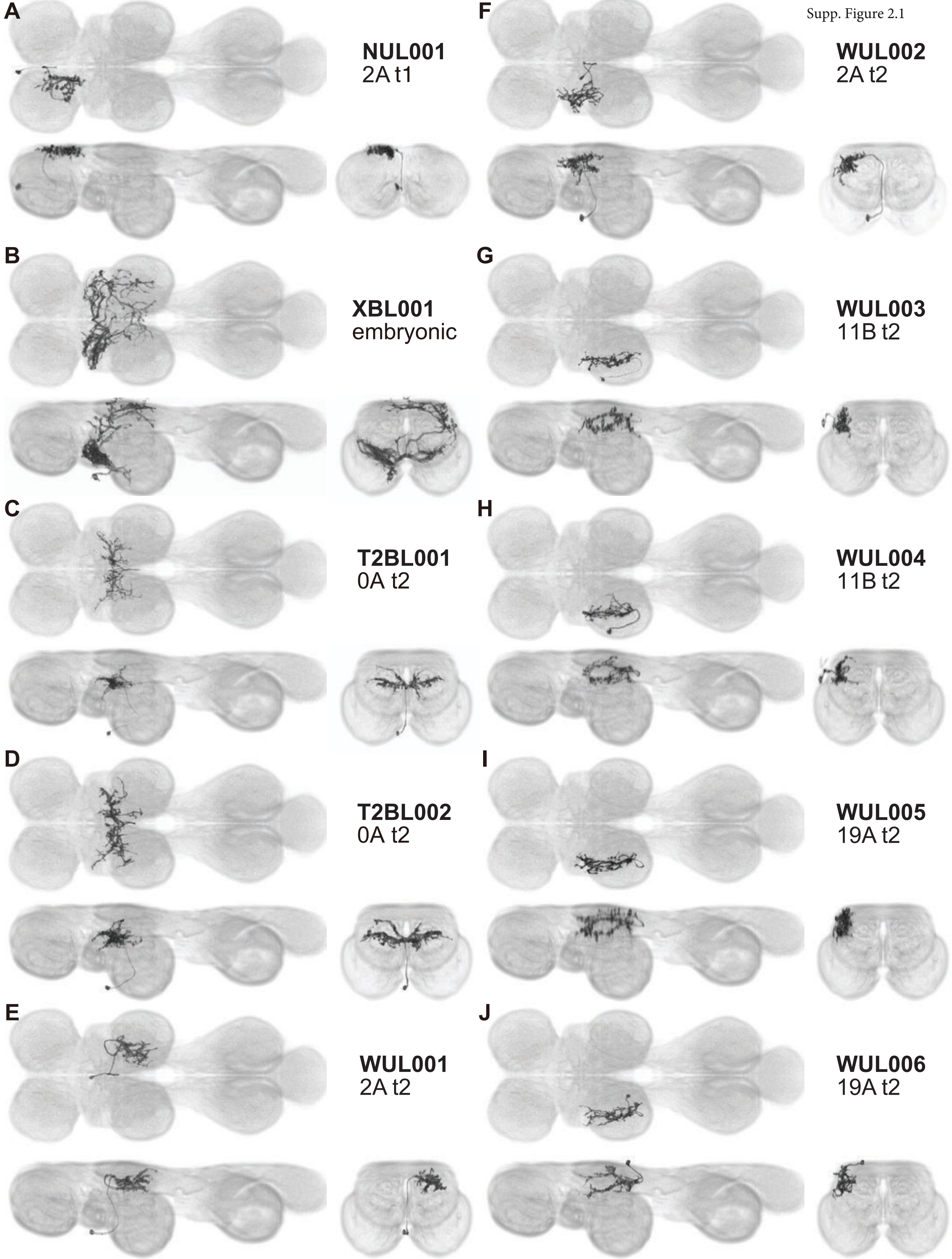
A
NUL001
2A t1
B
XBL001
embryonic
C
T2BL001
0A t2
D
T2BL002
0A t2
E
WUL001
2A t2
F
WUL002
2A t2
G
WUL003
11B t2
H
WUL004
11B t2
I
WUL005
19A t2
J
WUL006
19A t2

Supp. Figure 2.2

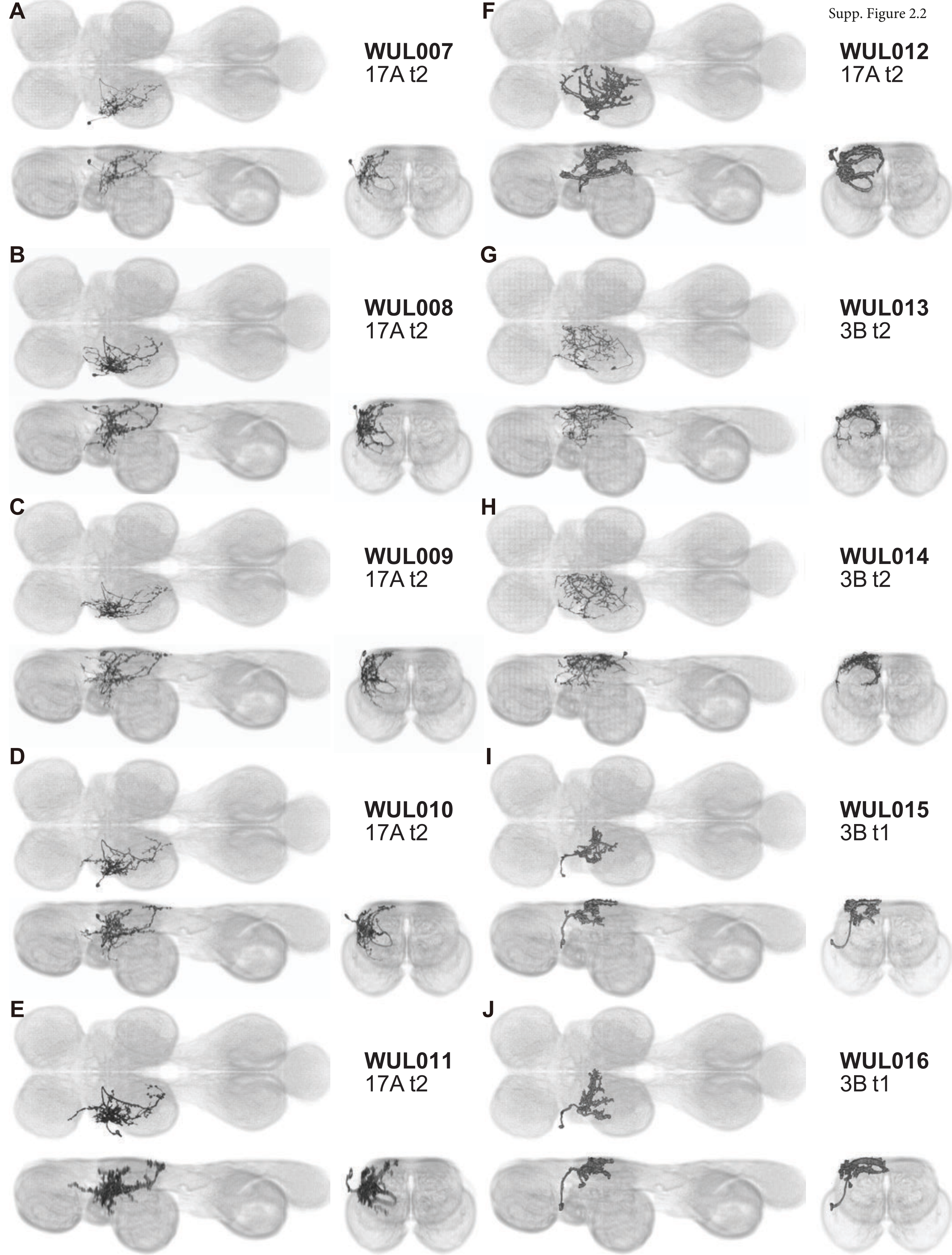

Supp. Figure 2.3

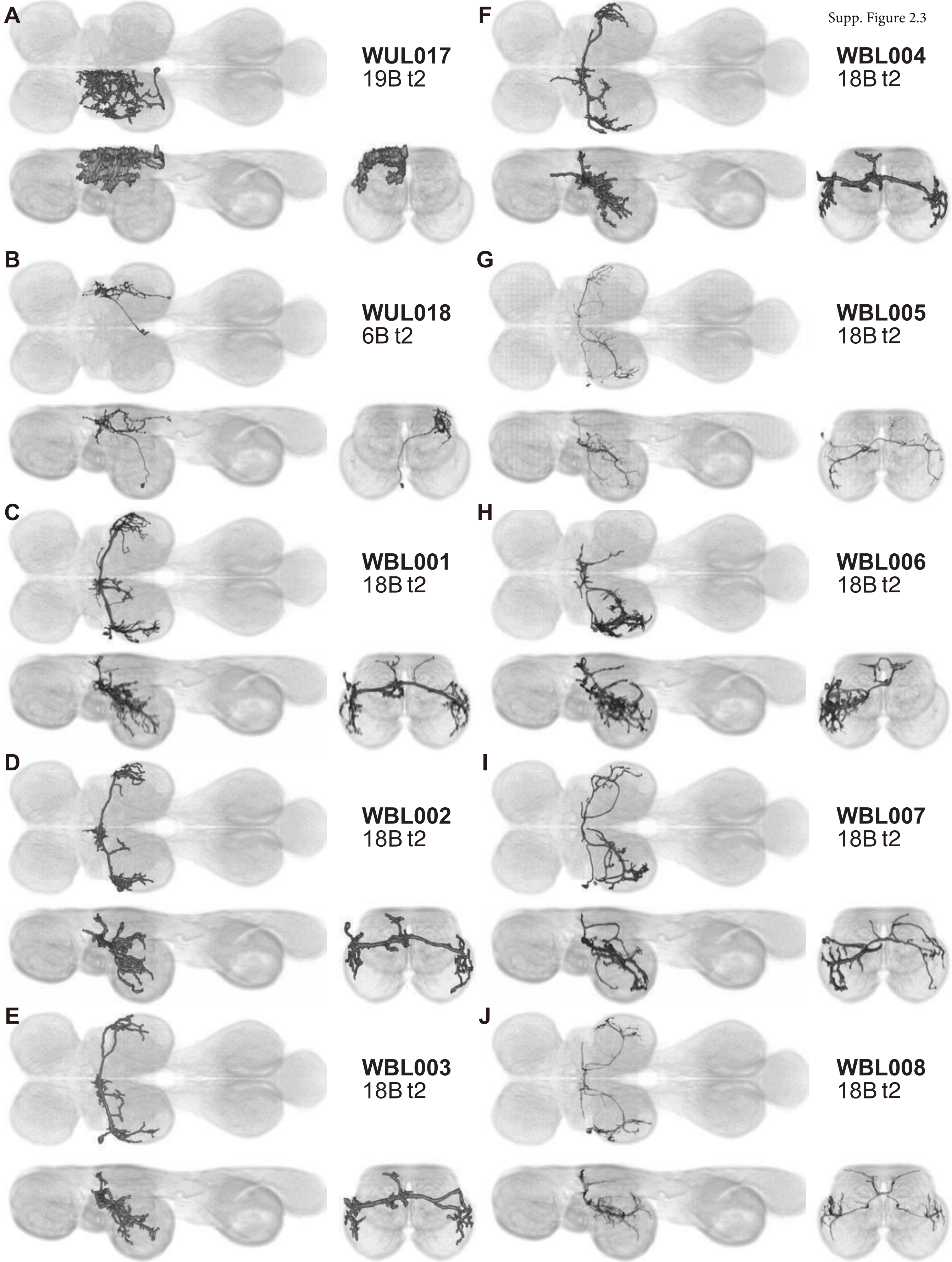

Supp. Figure 2.4

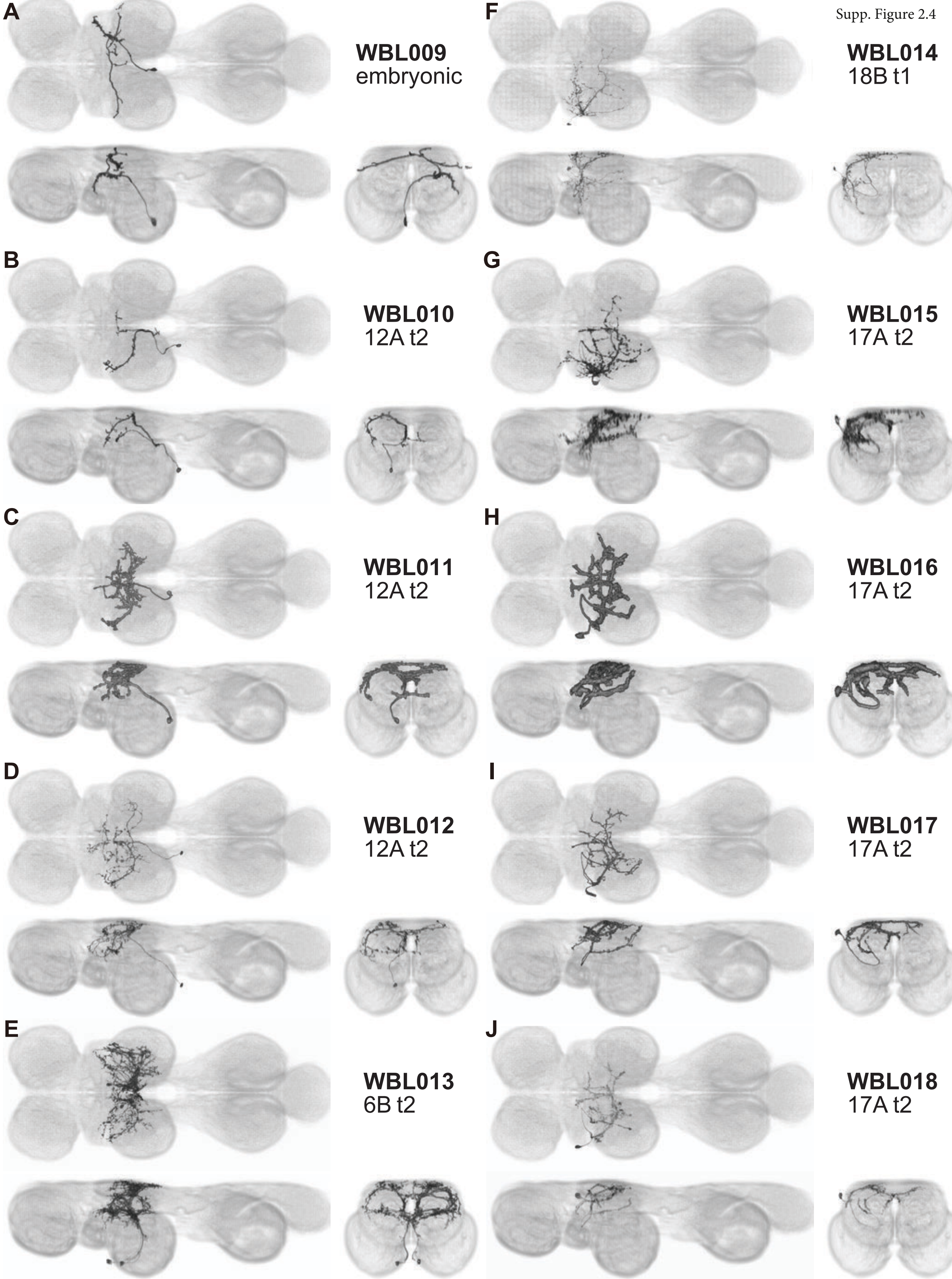

Supp. Figure 2.5

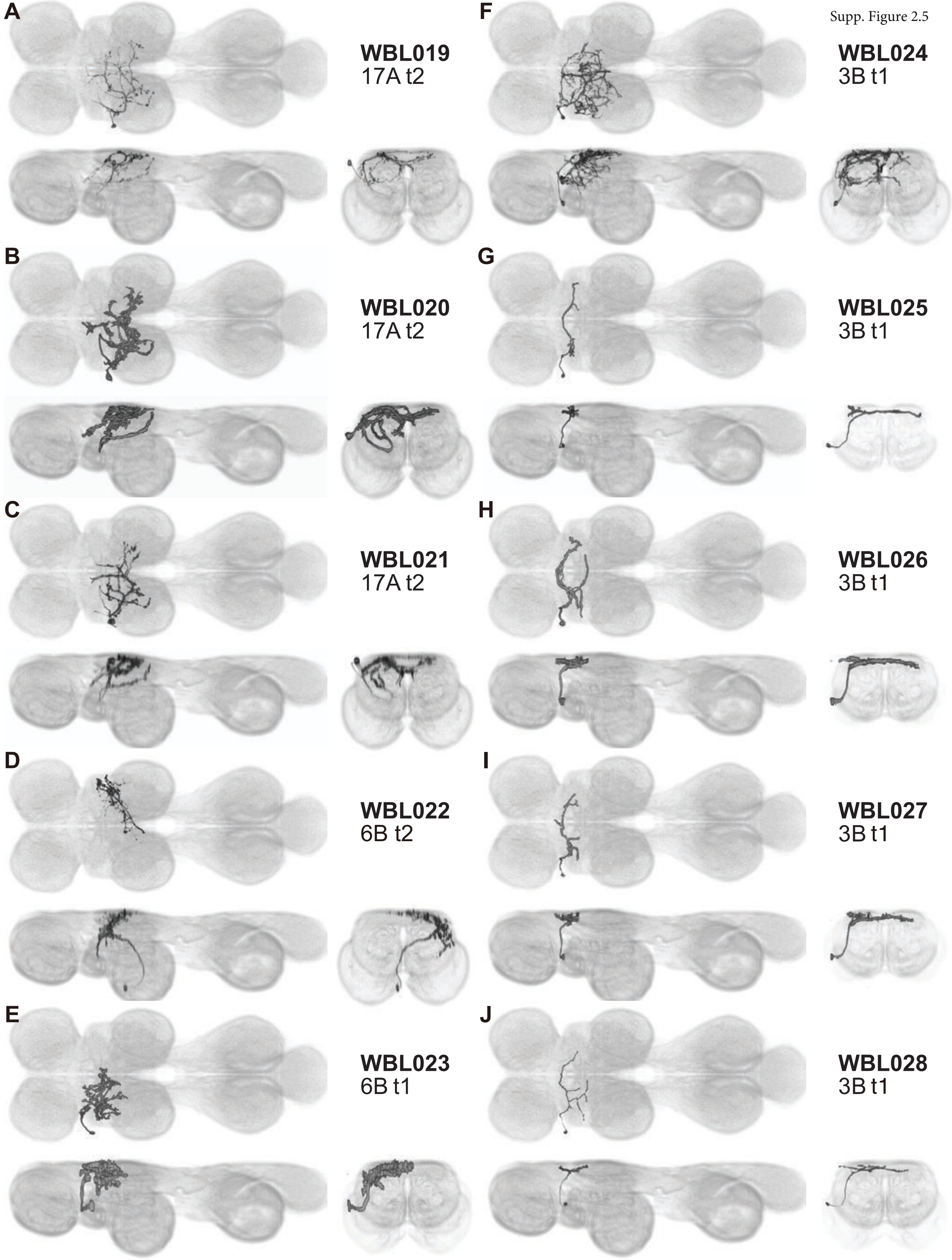

Supp. Figure 2.6

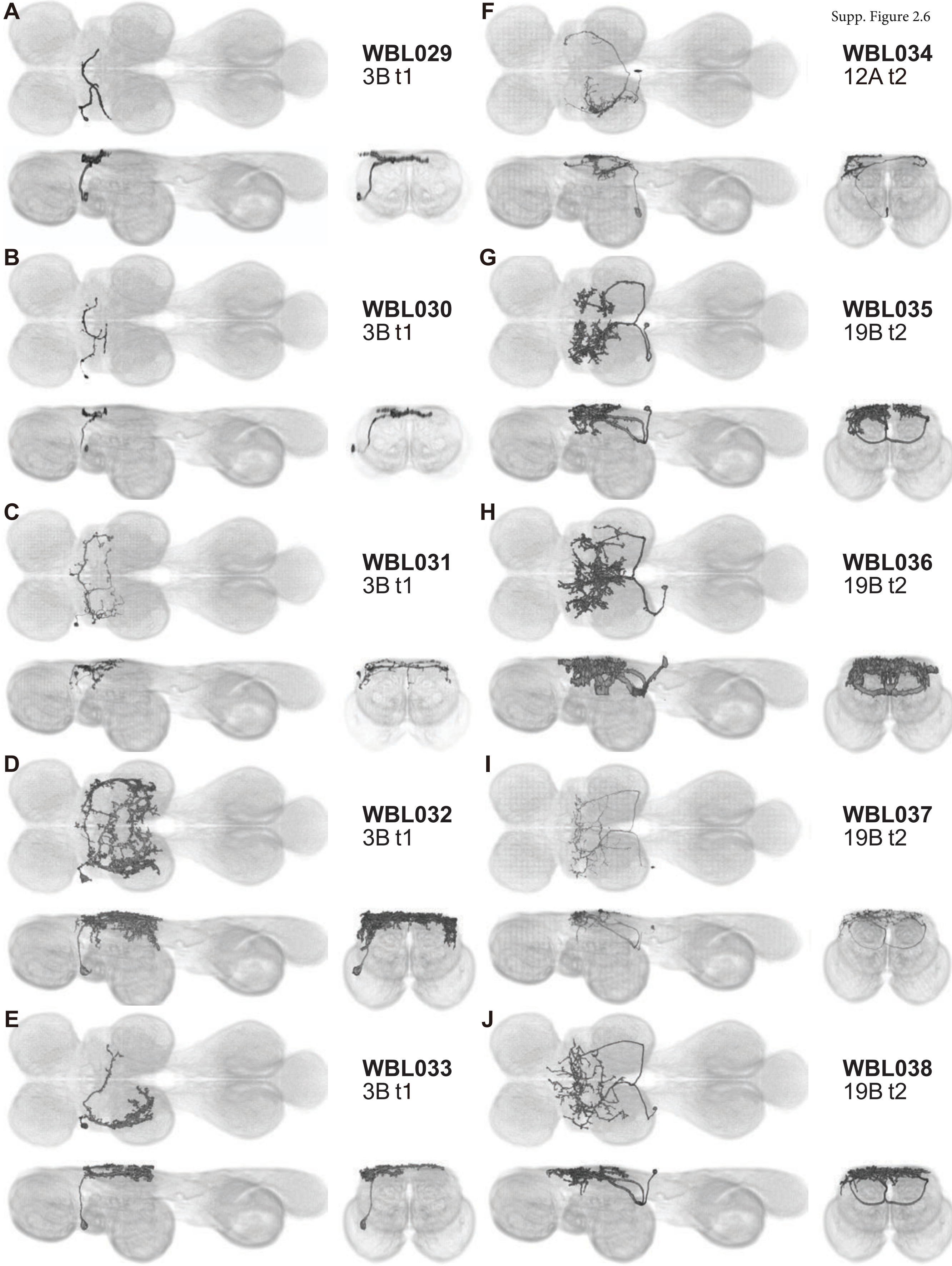

Supp. Figure 2.7

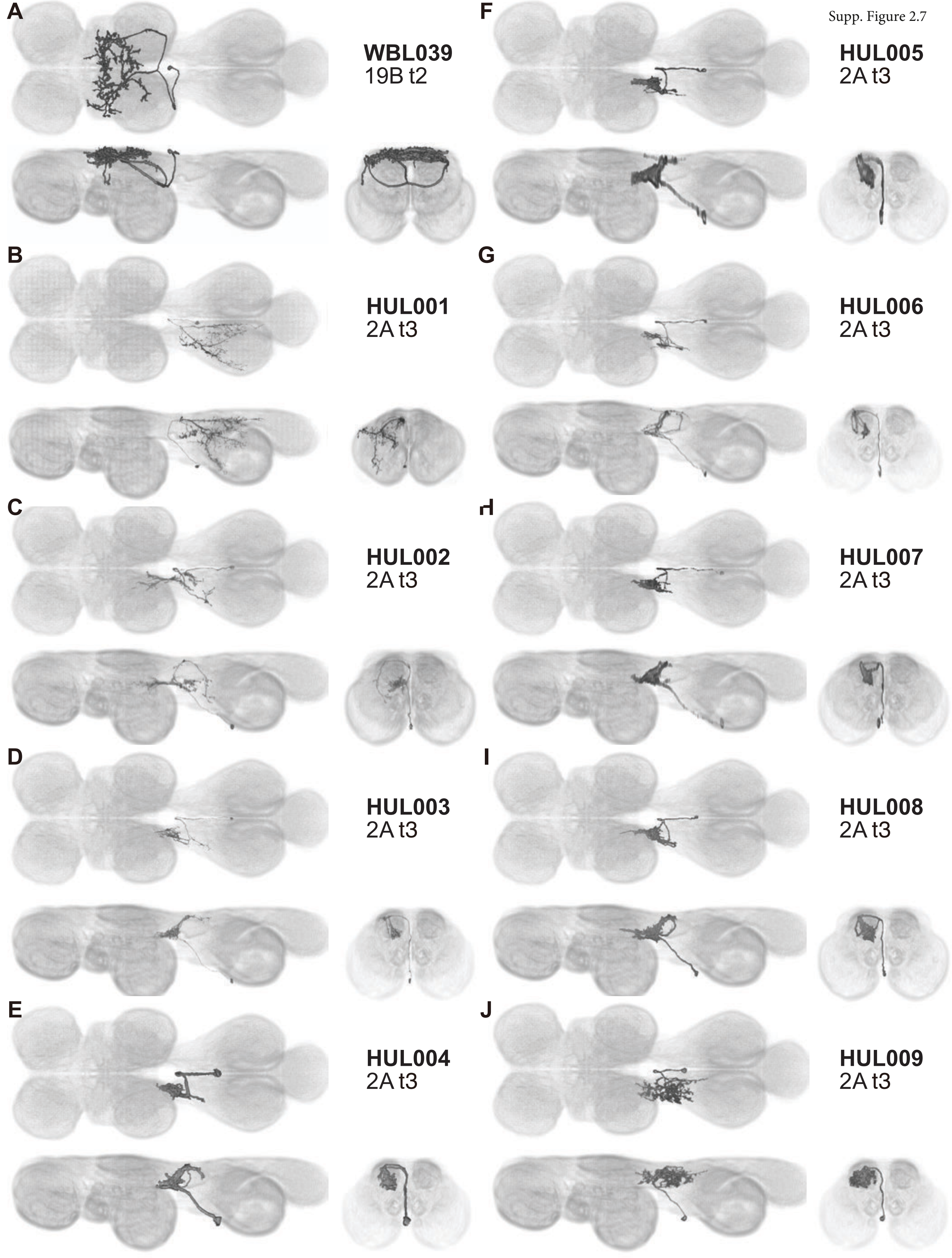

Supp. Figure 2.8

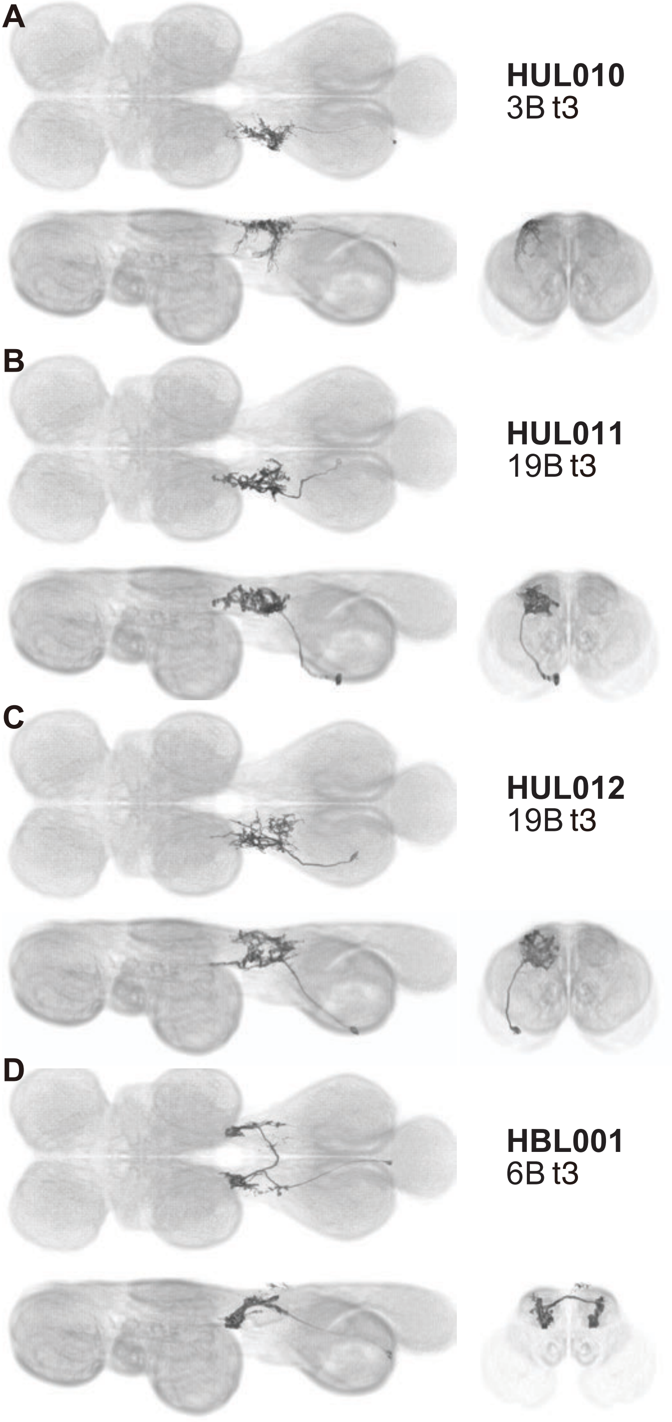

Supp. Figure 3.1

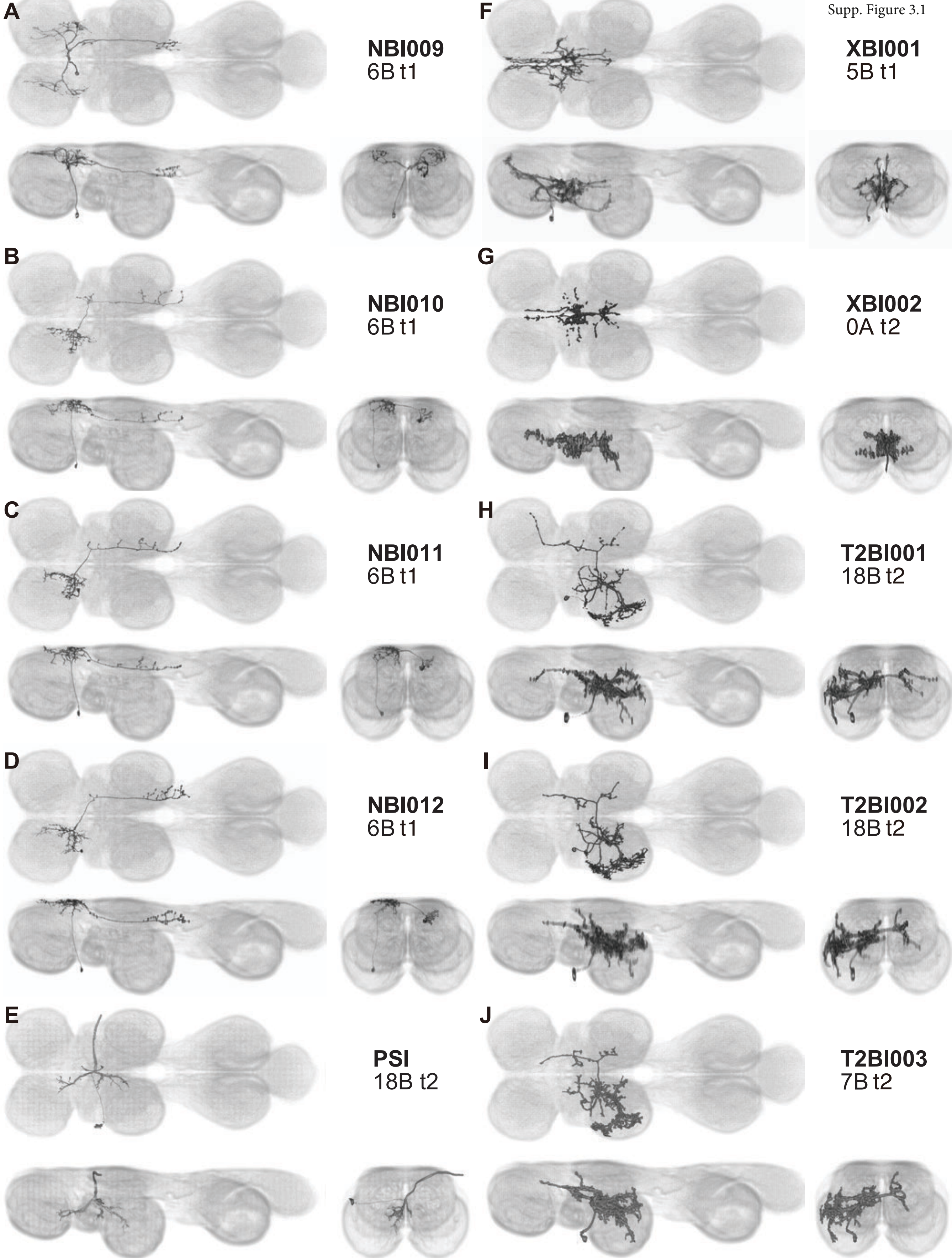

Supp. Figure 3.2

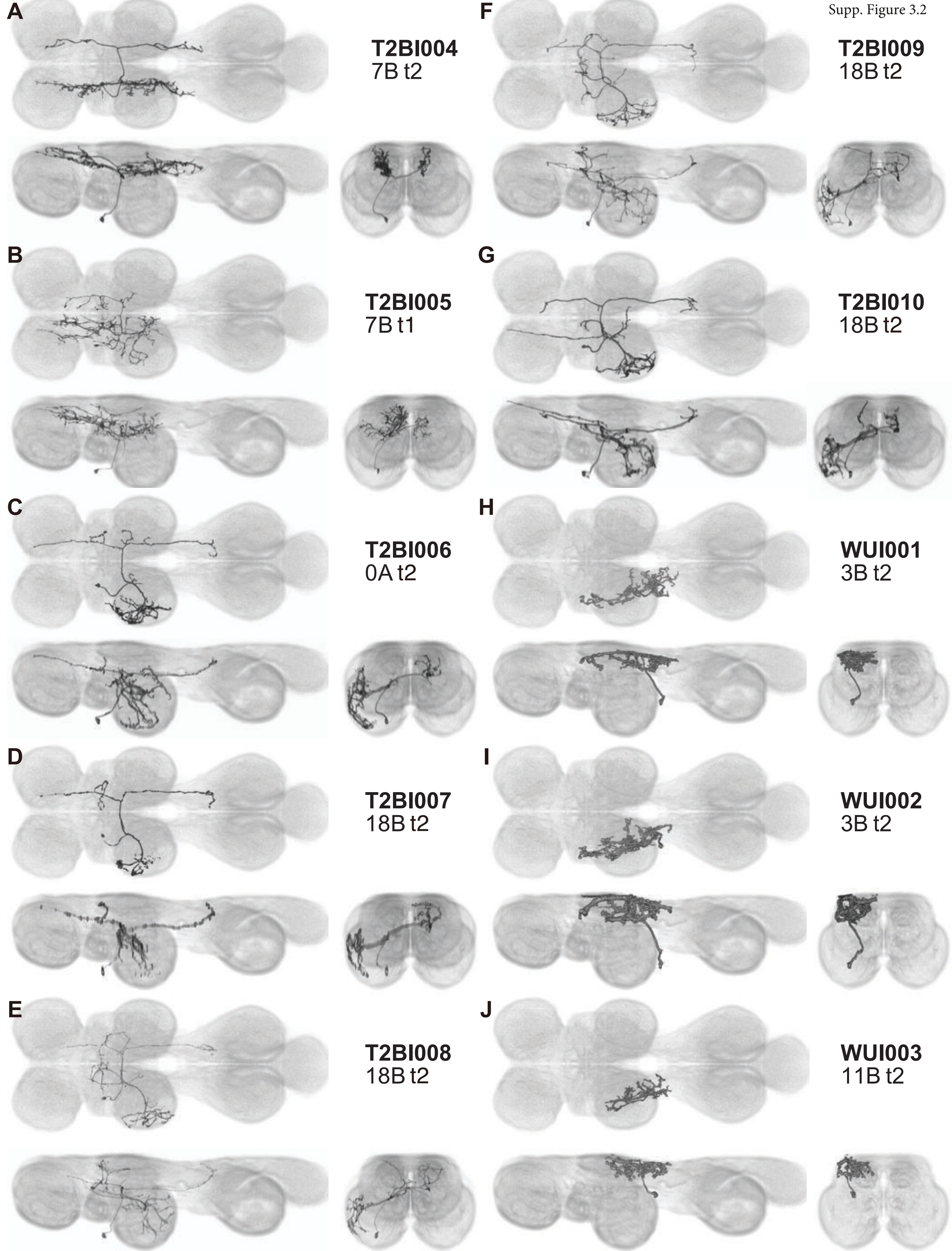

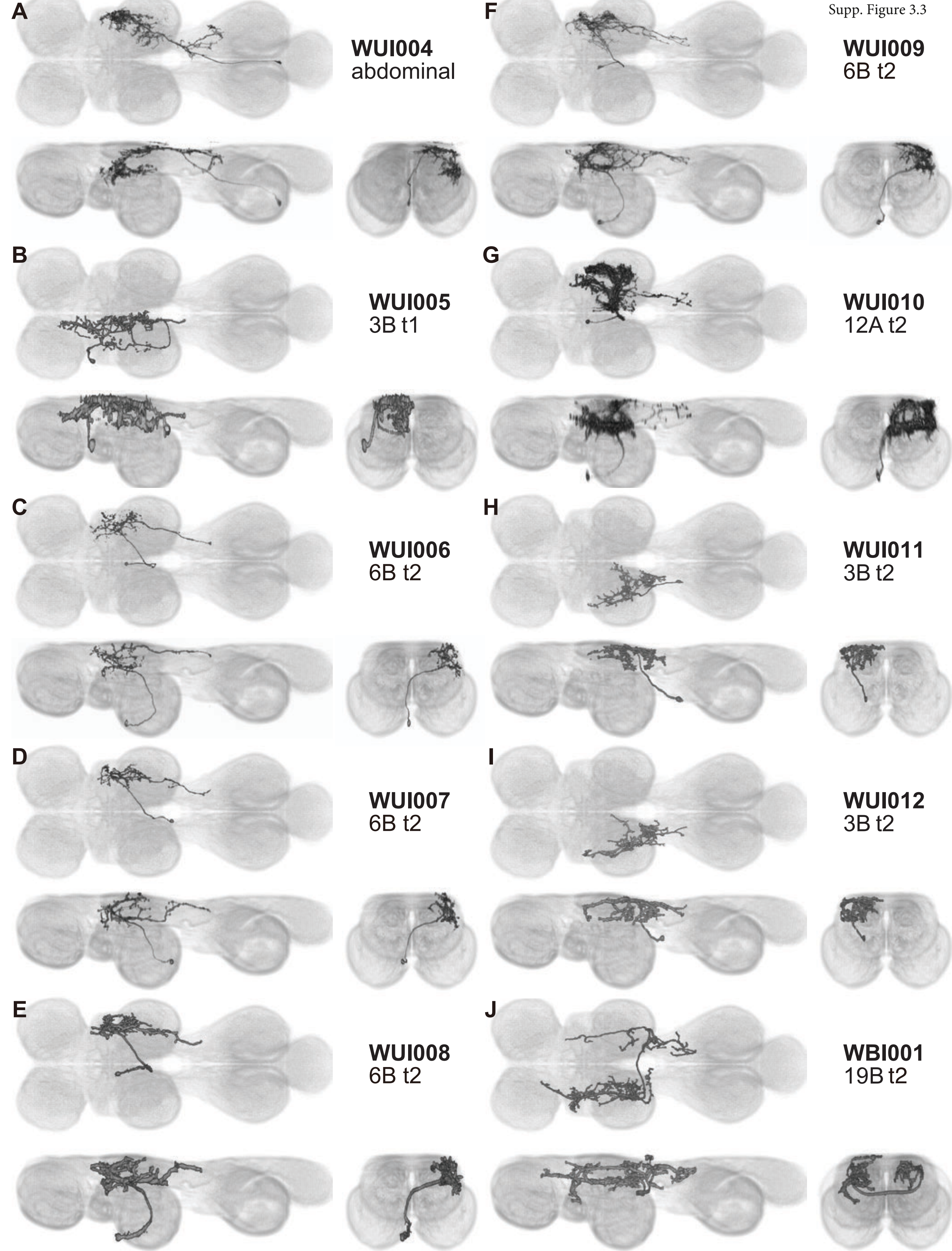
A
WUI004
abdominal
B
WUI005
3B t1
C
WUI006
6B t2
D
WUI007
6B t2
E
WUI008
6B t2
F
WUI009
6B t2
G
WUI010
12A t2
H
WUI011
3B t2
I
WUI012
3B t2
J
WBI001
19B t2

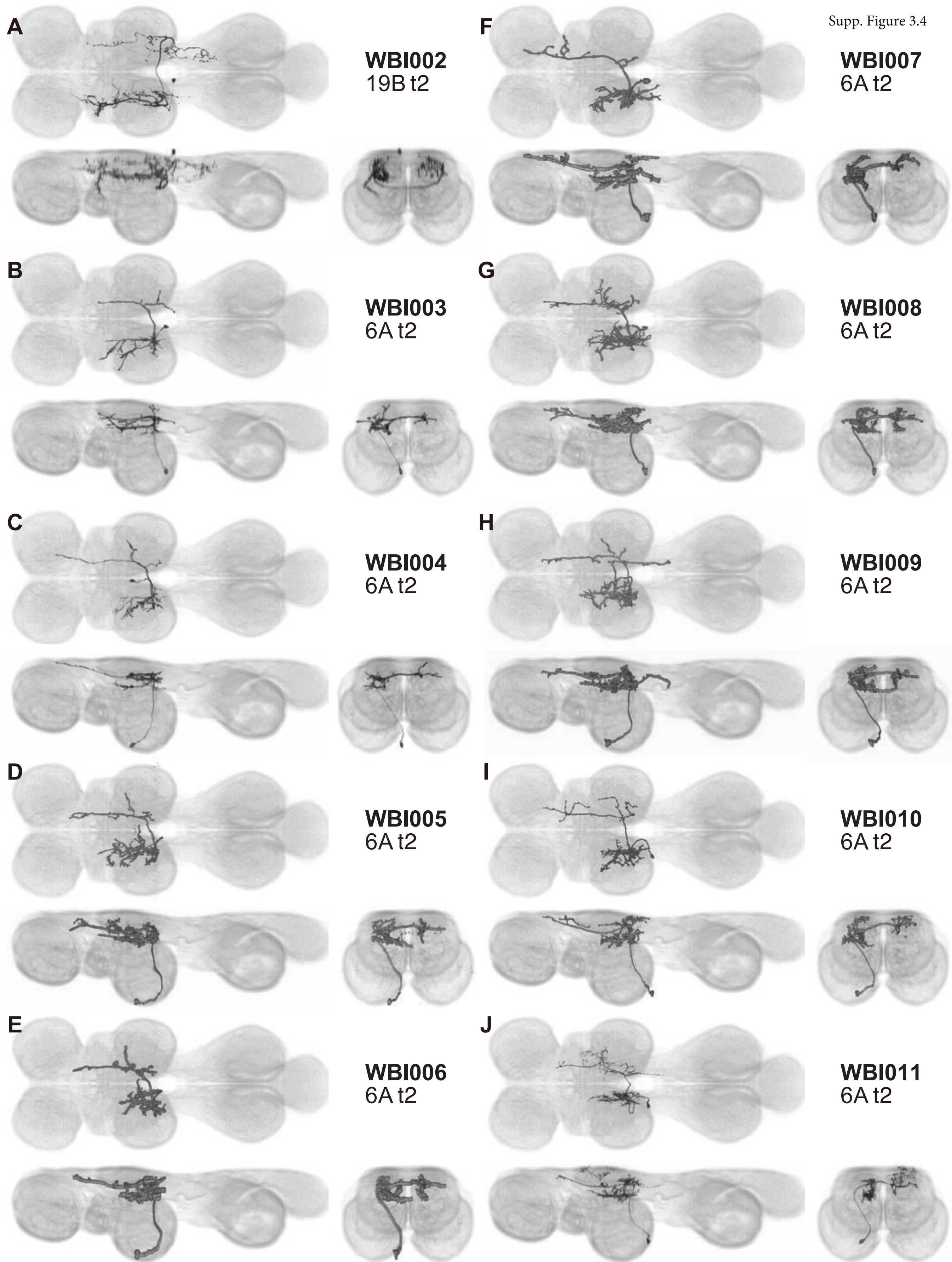
A
WBI002
19B t2
B
WBI003
6A t2
C
WBI004
6A t2
D
WBI005
6A t2
E
WBI006
6A t2
F
WBI007
6A t2
G
WBI008
6A t2
H
WBI009
6A t2
I
WBI010
6A t2
J
WBI011
6A t2

Supp. Figure 3.5

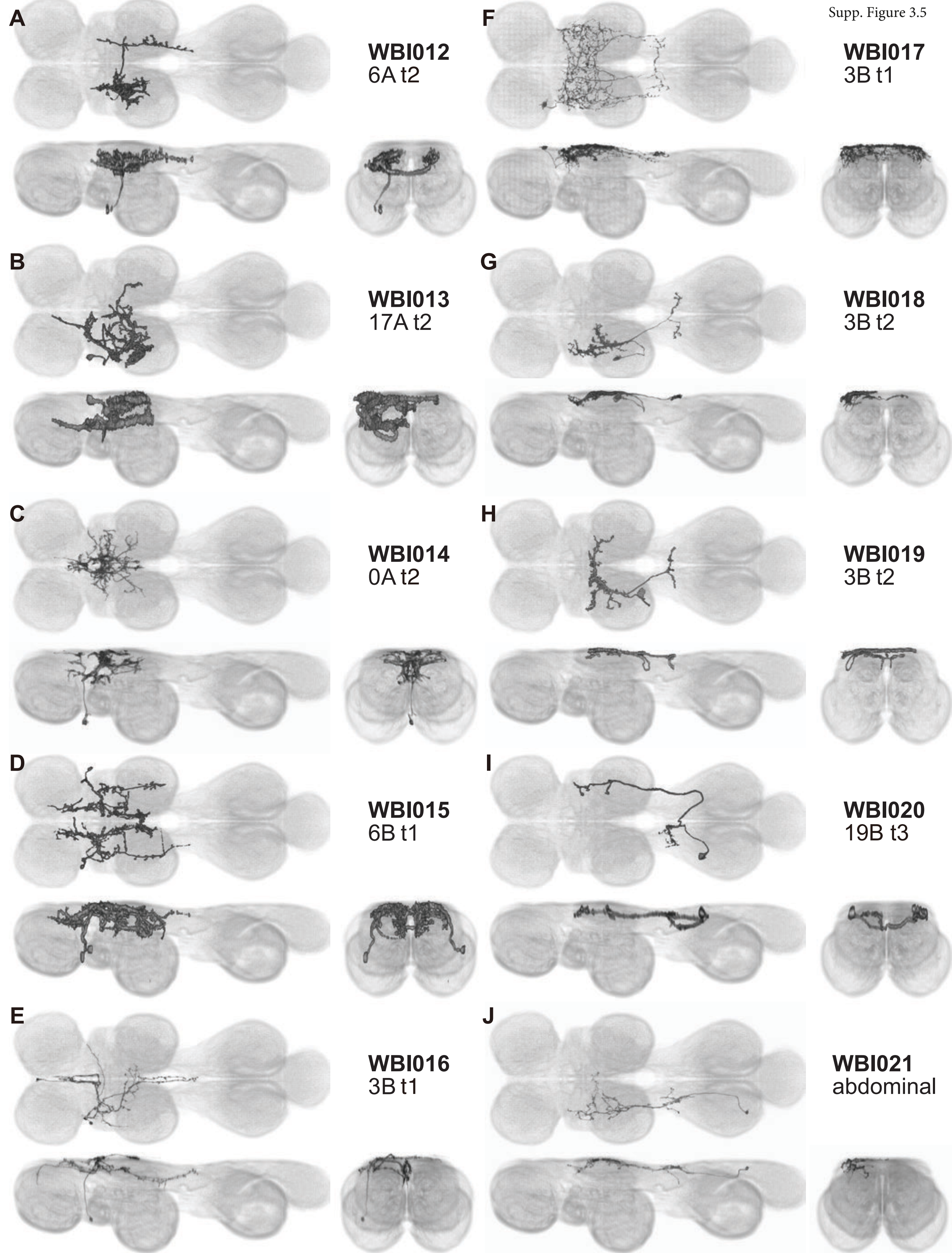

Supp. Figure 3.6

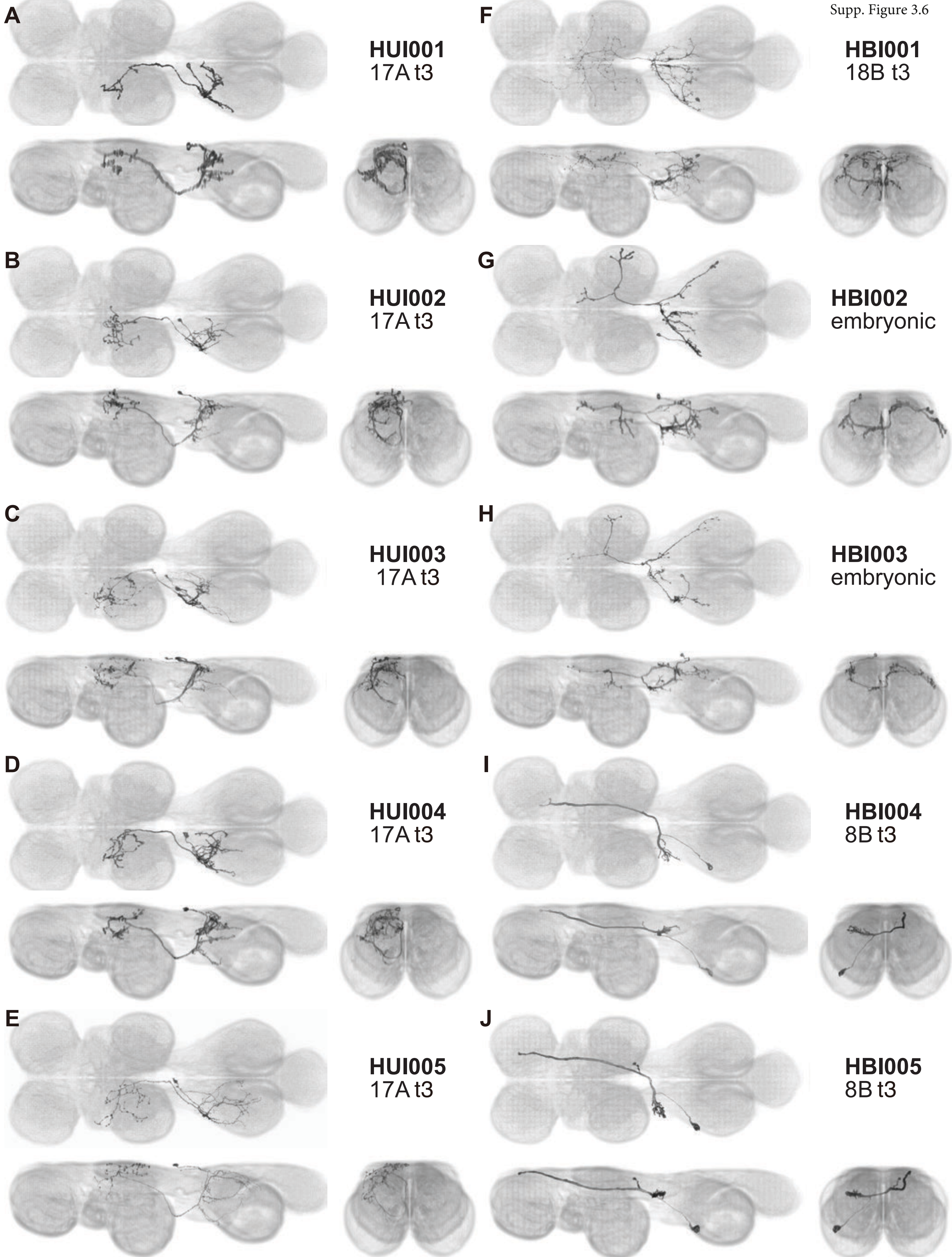

Supp. Figure 3.7

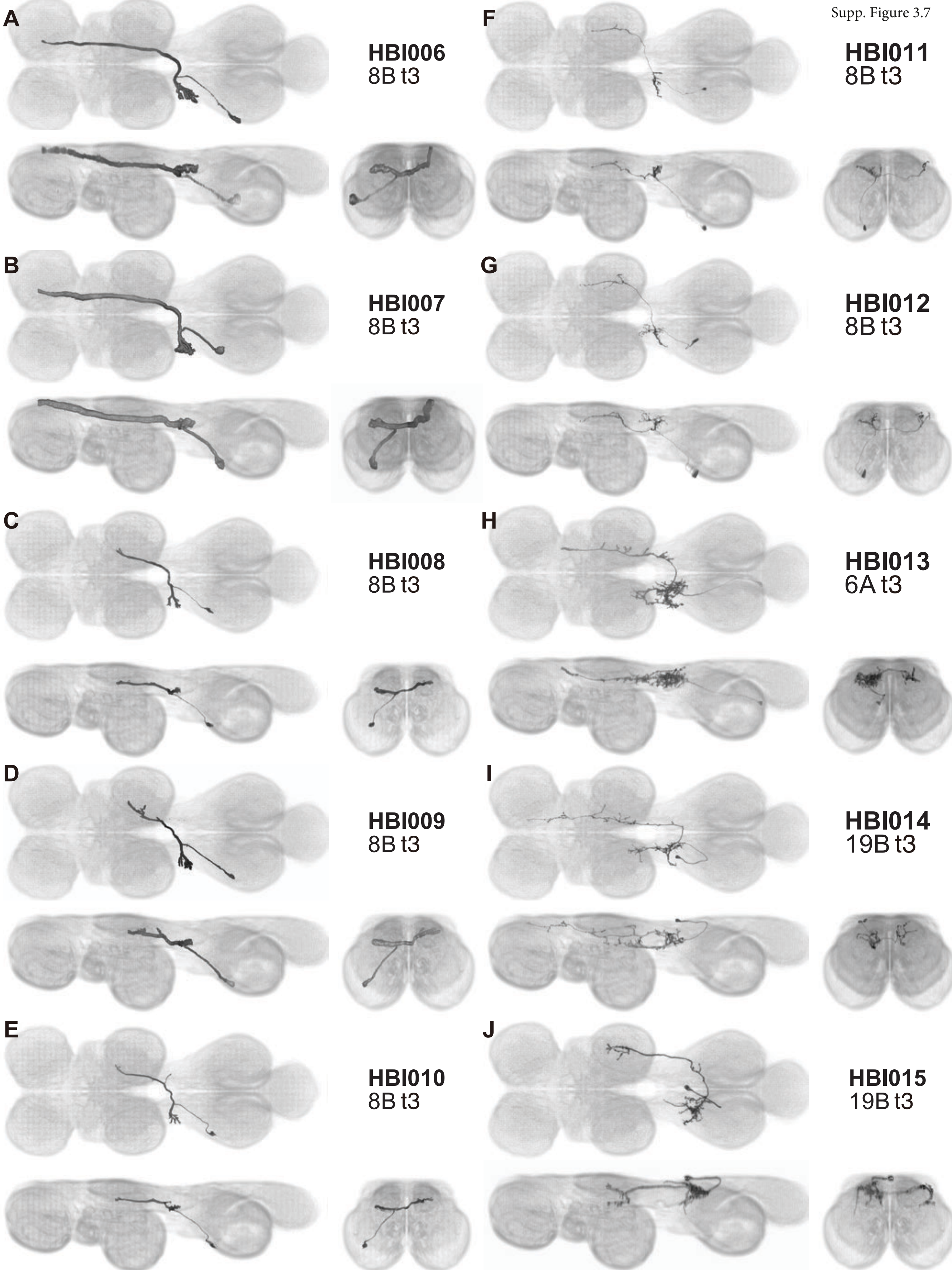

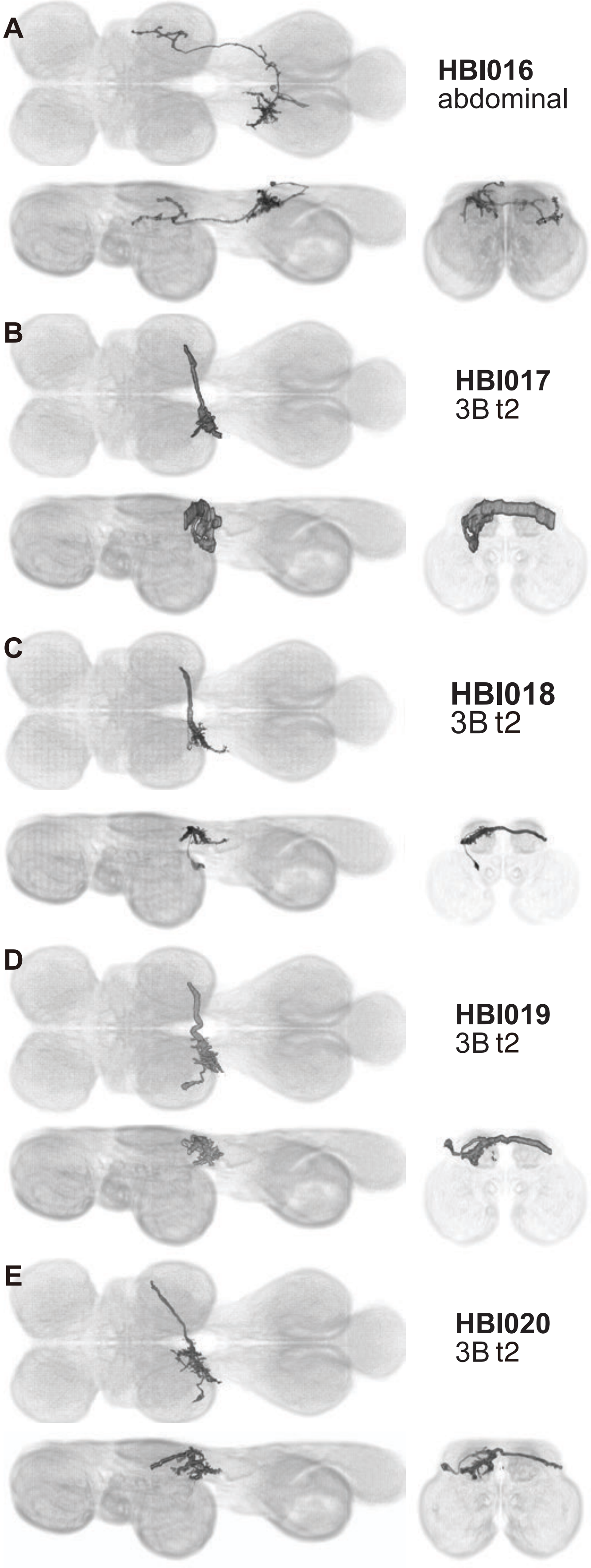
A
HBI016
abdominal
B
HBI017
3B t2
C
HBI018
3B t2
D
HBI019
3B t2
E
HBI020
3B t2

Supp. Figure 3.9

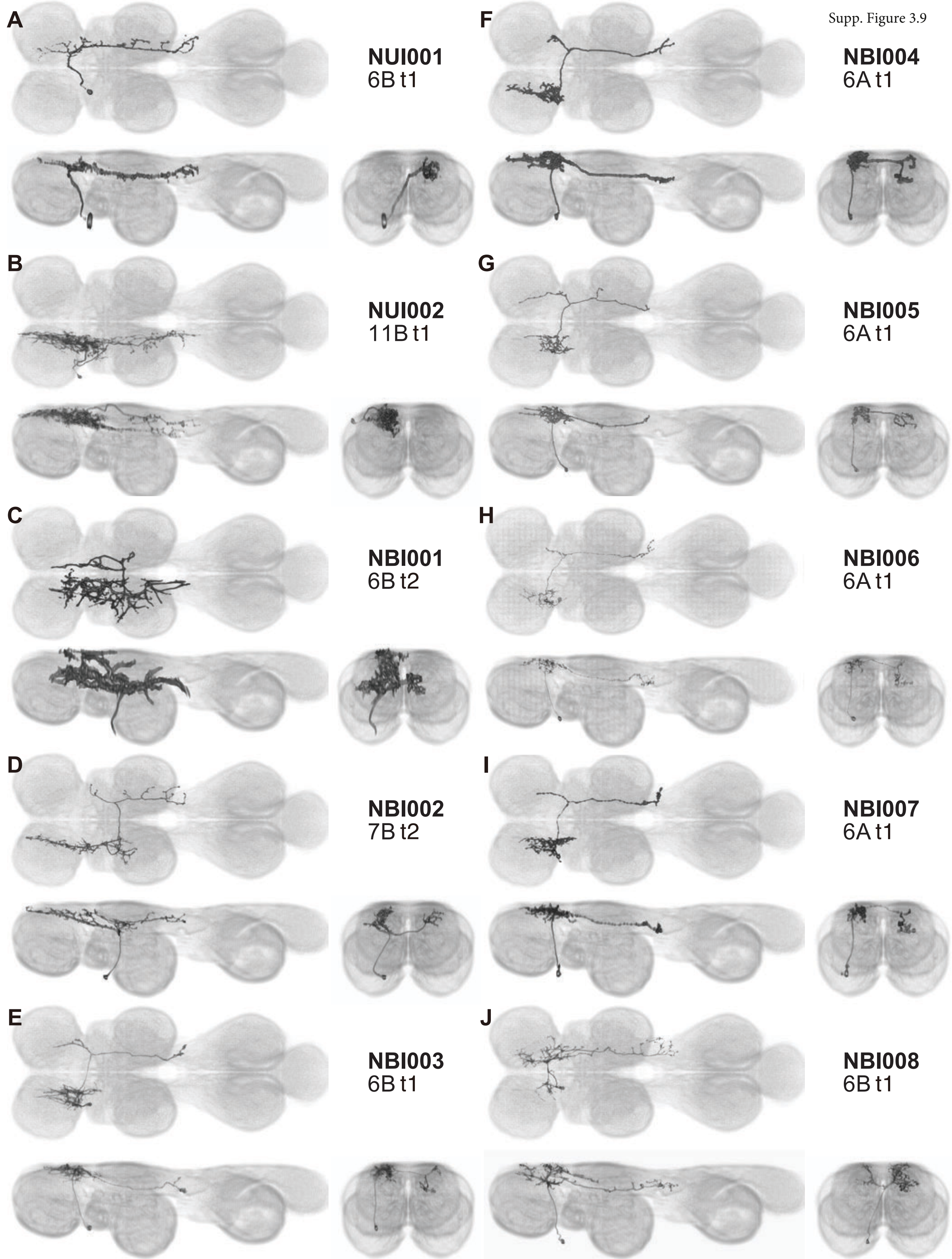

Supplement: Supplementary file 1 [file NIHPP2023.05.31.542897V2-supplement-1.pdf]
